# New sordarialean lineage *Garciamycella chlamydospora* (*Sordariales*, *Schizotheciaceae*) produces rare antifungal papulacandins

**DOI:** 10.3897/imafungus.17.177411

**Published:** 2026-01-14

**Authors:** Manuela Agudelo-Restrepo, Margarita Hernández-Restrepo, Mahmoud A.A. Ibrahim, Esteban Charria-Girón, Sherif S. Ebada, Yasmina Marin-Felix

**Affiliations:** 1 Department of Microbial Drugs, Helmholtz Centre for Infection Research GmbH (HZI), and German Centre for Infection Research Association (DZIF), Inhoffenstraße 7, 38124 Braunschweig, Germany Helmholtz Centre for Infection Research GmbH (HZI), and German Centre for Infection Research Association (DZIF) Braunschweig Germany; 2 Institute of Microbiology, Technische Universität Braunschweig, Spielmannstraße 7, 38106 Braunschweig, Germany Technische Universität Braunschweig Braunschweig Germany; 3 Westerdijk Fungal Biodiversity Institute, Uppsalalaan 8, 3584CT, Utrecht, Netherlands Westerdijk Fungal Biodiversity Institute Utrecht Netherlands; 4 Computational Chemistry Laboratory, Chemistry Department, Faculty of Science, Minia University, Minia 61519, Egypt Minia University Minia Egypt; 5 Department of Engineering, College of Engineering and Technology, University of Technology and Applied Sciences, Nizwa 611, Oman University of Technology and Applied Sciences Nizwa Oman; 6 School of Health Sciences, University of KwaZulu-Natal, Westville Campus, Durban 4000, South Africa University of KwaZulu-Natal Durban South Africa; 7 Bioformatics Group, Wageningen University & Research, Droevendaalsesteeg 1, 6708 PB Wageningen, Netherlands Wageningen University & Research Wageningen Netherlands; 8 Department of Pharmacognosy, Faculty of Pharmacy, Ain Shams University, 11566 Cairo, Egypt Ain Shams University Cairo Egypt

**Keywords:** Antimicrobials, *
Schizotheciaceae
*, secondary metabolites, *
Sordariales
*

## Abstract

The new genus *Garciamycella* is here erected to accommodate the soil-borne fungus *G. chlamydospora*, as well as *G. cyclaminis* and *G. fici*, based on a phylogenetic study using sequences of ITS, LSU, *rpb*2, and *tub*2. The establishment of *Garciamycella* has important taxonomic implications, as it helps to resolve a phylogenetically distinct lineage in the order *Sordariales*, a group in which the placement of numerous taxa remains uncertain. The new species *G. chlamydospora* was investigated for its secondary metabolite production, affording one previously undescribed papulacandin derivative (**1**), together with two known compounds from the same family, Mer-WF3010 (**2**) and papulacandin D (**3**). In addition, two previously undescribed metabolites, penazaphilone M (**4**) and cremenoic acid (**5**), were isolated alongside the known derivatives cremenolide (**6**) and aspinolide B (**7**). All compounds were isolated using preparative high-performance liquid chromatography (HPLC), and their chemical structures were elucidated through comprehensive 1D and 2D NMR spectroscopic analyses, in addition to high-resolution mass spectrometry (HR-MS). Antimicrobial and cytotoxic activities were assessed for all metabolites, and compounds (**1–3**) revealed potent antifungal activity. This research highlights how exploring novel fungal taxa can lead to the discovery of structurally unique metabolites with significant antifungal properties. It further confirms the potential of the order *Sordariales* as prolific producers of bioactive compounds with potential applications in the development of new antifungal agents.

## Introduction

Fungi are well established as prolific producers of secondary metabolites with unique and complex structures (Schrey et al. 2025). This remarkable diversity is attributed to their rich repertoire of biosynthetic genes and gene clusters, whose expression is often regulated by environmental and chemical factors and is closely linked to the developmental and morphological stages of the producing organism (Bills and Gloer 2016). The broad spectrum of biological activities exhibited by these metabolites underscores the pharmaceutical potential of fungi as a source of novel drug leads. This is particularly relevant in the fight against antimicrobial resistance, one of the most pressing challenges in global health (Hyde et al. 2024). In this context, the fungal order *Sordariales* stands out as a rich source of bioactive metabolites, with numerous structurally diverse compounds already reported (Charria-Girón et al. 2022). The antifungal sordarins exemplify the potential of members of this order to produce molecules with unique modes of action and promising clinical applications (Shao et al. 2022). Despite their remarkable chemical diversity and biotechnological potential, research has primarily focused on a few families within the order. For instance, data from the Natural Products Atlas (NPAtlas 3.0) indicate that approximately 75% of the reported metabolites belong to the single family *Chaetomiaceae*, emphasizing the disproportionate attention it has received (Ibrahim et al. 2021; Poynton et al. 2024). In contrast, most genera and even complete families within *Sordariales* remain largely unexplored (Charria-Girón et al. 2022).

The order *Sordariales* comprises nine families: *Chaetomiaceae*, *Podosporaceae*, *Sordariaceae*, *Lasiosphaeriaceae*, *Lasiosphaeridaceae*, *Schizotheciaceae*, *Naviculisporiaceae*, *Bombardiaceae*, and *Diplogelasinosporaceae*, delimited on the basis of multilocus phylogenies and morphology (Thiyagaraja et al. 2025). Among these, *Schizotheciaceae* stands as one of the largest families. It was introduced based on morphological data and phylogenetic analyses using sequences of the internal transcribed spacer region (ITS), the nuclear rDNA large subunit (LSU), and fragments of RNA polymerase II subunit 2 (*rpb*2) and β-tubulin (*tub*2) genes. This polyphasic approach is considered more accurate for identifying taxa within the *Sordariales* (Marin-Felix et al. 2020). Despite its taxonomic importance, the metabolome of its members remains poorly studied (Charria-Girón et al. 2022).

Phylogenetic gaps within *Sordariales* restrict natural product discovery, as poorly resolved or unsampled lineages tend to be overlooked in targeted bioprospection campaigns. In recent years, a few studies have begun to address this gap, demonstrating the promising bioactive potential of unexplored taxa. For instance, seven novel xanthone–anthraquinone heterodimers, xanthoquinodin A1 and xanthoquinodins B10–B15, were isolated from *Jugulospora vestita* (CBS 135.91), all exhibiting inhibitory activity against Gram-positive bacteria and cytotoxicity toward mammalian cell lines (Shao et al. 2020). Similarly, the depsipeptide morinagadepsin, isolated from *Morinagamyces vermicularis* (CBS 303.81), showed cytotoxic activity against mammalian cell lines (Harms et al. 2021b). Furthermore, four previously undescribed polythiodiketopiperazine derivatives were reported from the same strain using MS/MS-metabolomics workflows. Three of these compounds were evaluated for antimicrobial and cytotoxic activity, revealing weak to moderate activity against Gram-positive bacteria and fungi. Notably, botryosulfuranol D exhibited the strongest antifungal activity, particularly against *Mucor hiemalis* (Harms et al. 2024).

To the best of our knowledge, these reports underscore the importance of expanding research on novel fungal compounds beyond well-studied taxa. In this context, the ethyl acetate (EtOAc) extract of *Garciamycella chlamydospora*, a new sordarialean lineage within the family *Schizotheciaceae*, was explored and afforded seven compounds, including three assigned as previously undescribed. All isolated compounds were assessed for their antimicrobial and cytotoxic activities. Herein, we introduce *G. chlamydospora*, and further describe the chemical and biological characterization of the secondary metabolites from its crude extract.

## Materials and methods

### Fungal isolation

Soil samples were collected during a citizen science project conducted by the Westerdijk Fungal Biodiversity Institute, Utrecht, The Netherlands (WI) in 2017. Isolation of axenic cultures was carried out following the serial dilution methodology according to Groenewald et al. (2018) and Giraldo et al. (2019). Other reference strains were retrieved from the CBS culture collection housed at WI. The living strain and dry material were deposited in the CBS Culture Collection and the Fungarium of the WI, respectively.

### Morphological characterization

Cultural characteristics were described from colonies grown on malt extract agar (MEA) and potato–carrot agar (PCA) (both from HiMedia, Mumbai, India), oatmeal agar (OA; Sigma–Aldrich, St. Louis, MO, USA), and potato dextrose agar (PDA; Pronadisa, Madrid, Spain) at 25 °C for 14 days. Colony colors were assessed following the color chart of the Royal Horticultural Society (1996). Micromorphological descriptions and measurements of 30 replicates of reproductive structures and relevant features were carried out in 90% lactic acid. Photomicrographs were taken using a Zeiss Axioskop 2 plus and a Nikon Eclipse Ni compound microscope, both equipped with a DS-Ri2 camera (Nikon, Tokyo, Japan) and NIS-Elements imaging software v. 5.20.

### DNA isolation, amplification, and phylogenetic study

Genomic DNA was extracted using the EZ-10 SPIN Column Fungal Genomic DNA Minipreps Kit (NBS Biologicals, Cambridgeshire, UK), following the manufacturer’s instructions. Amplification of the internal transcribed spacer (ITS) regions and the large subunit (LSU) of the nuclear ribosomal RNA (rRNA) gene complex, as well as partial fragments of RNA polymerase II subunit 2 (*rpb*2) and β-tubulin (*tub*2) genes, was performed according to White et al. (1990) (ITS; primers ITS5 and ITS4), Vilgalys and Hester (1990) (LSU; primers LR0R and LR7), Liu et al. (1999) (*rpb*2; primers RPB2-5F and RPB2-7cR), and Miller and Huhndorf (2005) (*tub*2; primers BT1819R and BT2916). PCR reactions were performed using JumpStart™ Taq ReadyMix™ (Sigma–Aldrich, St. Louis, MO, USA), and the products were sequenced using the Sanger cycle sequencing method at Microsynth Seqlab GmbH (Göttingen, Germany). Consensus sequences were assembled using Geneious® 7.1.9 (Kearse et al. 2012).

Phylogenetic analyses were carried out based on the combined dataset of four loci from the isolate obtained in this study and those of type and reference strains belonging to the *Sordariales* (Table 1). Each locus was aligned separately using MAFFT v. 7 (Katoh et al. 2013) and manually optimized in MEGA v. 10.2.4 (Kumar et al. 2018). In the ITS alignment, a poorly aligned and ambiguous region within ITS1 was manually removed. Maximum likelihood (ML) analyses of single-locus datasets were performed on the CIPRES portal (www.phylo.org) using RAxML-HPC BlackBox v. 8.2.12 with default parameters (Stamatakis 2014). The four loci were subsequently concatenated after confirming the absence of topological conflicts, using SequenceMatrix (Vaidya 2011). ML analysis of the concatenated dataset was conducted using the same settings as those for the single-locus analyses, and Bayesian inference (BI) was carried out in MrBayes v. 3.2.1 (Ronquist et al. 2012), employing the same parameters as described by Harms et al. (2021a). Bootstrap support (BS) values ≥ 70% and posterior probability (PP) values ≥ 0.95 were considered significant (Alfaro et al. 2003). Sequences generated in this study have been deposited in GenBank (Table 1), and the final alignment is provided in the Suppl. material 1.

**Table 1.** Our isolated and selected strains belonging to the order *Sordariales* included in the phylogenetic study, with their accession numbers.

Taxa	Strain	GenBank accession numbers				References
		LSU	ITS	*rpb2*	*tub2*	
*Amesia atrobrunnea*	CBS 379.66^T^	MH870470	MH858833	KX976798	–	Wang et al. (2016a), Vu et al. (2019)
Anopodium ampullaceum*	MJR 40/07	KF557662	–	–	KF557701	Kruys et al. (2015)
	E00218015	KF557663	–	–	KF557702	Kruys et al. (2015)
Apiosordaria microcarpa*	CBS 692.82^T^	MK926841	MK926841	MK876803	–	Wang et al. (2019)
*Apodospora gotlandica*	E00204952	KF557664	–	–	KF557703	Kruys et al. (2015)
*Apodospora peruviana*	CBS 118394	KF557665	EU573703	–	–	Kruys et al. (2015), Debuchy et al. (unpubl. data)
*Apodospora simulans*	Kruys 701	KF557666	–	–	KF557704	Kruys et al. (2015)
*Apodus deciduus*	CBS 506.70^T^	AY681165	AY681199			Cai et al. (2006)
Arnium cirriferum*	CBS 120041	KF557673	–	–	KF557709	Kruys et al. (2015)
Bellojisia rhynchostoma*	CBS 118484	EU999217	–	–	–	Réblová (2008)
*Bombardia bombarda*	SMH 3391	AY346263	–	AY780153	AY780090	Huhndorf et al. (2004), Miller and Huhndorf (2005)
Bombardioidea anartia*	HHB99-1	AY346264	–	AY780155	AY780092	Huhndorf et al. (2004), Miller and Huhndorf (2005)
Cercophora newfieldiana*	SMH 3303	AY780062	–	AY780167	AY780106	Miller and Huhndorf (2005)
Cercophora scortea*	GJS L556	AY780063	–	AY780168	AY780107	Miller and Huhndorf (2005)
Cercophora sparsa*	JF 00229	AY587937	AY587912	–	AY600253	Miller and Huhndorf (2004b)
Cercophora sulphurella*	SMH 2531	AY587938	AY587913	AY600276	AY600254	Miller and Huhndorf (2004b)
Cercophora thailandica*	MFLUCC 12-0845^T^	KU863127	KU940139	KU940176	–	Dai et al. (2017)
*Chaetomium globosum*	CBS 160.62^T^	MH869713	KT214565	KT214666	–	Vu et al. (2019), Wang et al. (2016b)
Corylomyces selenosporus*	CBS 113930^T^	DQ327607	MT784130	KP981612	KP981557	Stchigel et al. (2006), Marin-Felix et al. (2020)
*Corynascus sepedonium*	CBS 111.69^T^	MH871003	MH859271	FJ666394	–	Vu et al. (2019), Greif et al. (2009)
*Echria gigantospora*	F77-1	KF557674	–	–	KF557710	Kruys et al. (2015)
*Echria macrotheca*	Lundqvist 2311	KF557684	–	–	KF557715	Kruys et al. (2015)
*Episternus onthophagi*	KRAM F58223^T^	KP903375	KP903374	–	–	Górz and Boroń (2018)
*Fimetariella rabenhorstii*	Lundqvist 20410-c	KF557694	–	–	KF557721	Kruys et al. (2015)
**Garciamycella chlamydospora**	**CBS 150388^T^**	**PX270288**	**PX270286**	**PX275625**	**PX275627**	Present study
**Garciamycella cyclaminis**	CBS 166.42^T^	MH867608	HQ713776	**PX275626**	–	Grünig et al. (2011), Vu et al. (2019), present study
	CBS 120402	KP981429	**PX270287**	KP981611	KP981556	Marin-Felix et al. (2020), present study
**Garciamycella fici**	MFLUCC 20-0160^T^	MW114439	MW114387	–	–	Tennakoon et al. (2021)
*Immersiella caudata*	SMH 3298	AY436407	–	AY780161	AY780101	Miller and Huhndorf (2004a, 2005)
*Immersiella hirta*	E00204950	KF557675	–	–	KF557711	Kruys et al. (2015)
	E00204487	KF557676	–	–	KF557712	Kruys et al. (2015)
*Immersiella immersa*	SMH 4104	AY436409	–	AY780181	AY780123	Miller and Huhndorf (2004a, 2005)
	SMH 2589	AY436408	–	–	–	Miller and Huhndorf (2004a)
*Jugulospora antarctica*	IMI 381338^T^	KP981433	–	KP981616	KP981561	Marin-Felix et al. (2020)
*Jugulospora carbonaria*	ATCC 34567	AY346302	–	AY780196	AY780141	Huhndorf et al. (2004), Miller and Huhndorf (2005)
*Jugulospora rotula*	FMR 12690	KP981437	MT784133	KP981620	KP981565	Marin-Felix et al. (2020)
*Jugulospora vestita*	CBS 135.91^T^	MT785872	MT784135	MT783824	MT783825	Marin-Felix et al. (2020)
*Lasiosphaeria lanuginosa*	SMH 3819	AY436412	AY587921	AY600283	AY600262	Miller and Huhndorf (2004a, b)
*Lasiosphaeria miniovina*	SMH 2392^T^	MH700179	MH700179	–	–	Crous et al. (2018)
*Lasiosphaeria ovina*	SMH 1538	AF064643	AY587926	AY600287	AF466046	Fernandez et al. (1999, 2006), Miller and Huhndorf (2004b)
*Lasiosphaeria rugulosa*	SMH 1518	AY436414	AY587933	AY600294	AY600272	Miller and Huhndorf (2004a, b)
*Lasiosphaeria similisorbina*	AR 1884^T^	MF806376	MF806376	–	–	Crous et al. (2017)
*Lasiosphaeria sorbina*	CBS 885.85	AY436416	AY587935	AY600296	AY600274	Miller and Huhndorf (2004a, b)
*Lasiosphaeris arenicola*	ANM 1080	JN673037	JN673037	–	–	Raja et al. (2011)
*Lasiosphaeris hirsuta*	SMH 1543	AY436417	–	AY780179	AY780121	Miller and Huhndorf (2004a, 2005)
	JF 02183	AY436418	–	–	–	Miller and Huhndorf (2004a)
*Lasiosphaeris hispida*	SMH 3336	AY436419	–	AY780180	AY780122	Miller and Huhndorf (2004a, 2005)
	CBS 955.72	MH872327	AY681203	–	–	Cai et al. (2006), Vu et al. (2019)
*Lundqvistomyces karachiensis*	CBS 657.74	KP981447	MK926850	KP981630	KP981478	Wang et al. (2019), Marin-Felix et al. (2020)
*Lundqvistomyces tanzaniensis*	TRTC 51981^T^	AY780081	MH862260	AY780197	AY780143	Miller and Huhndorf (2005), Vu et al. (2019)
*Mammaria echinobotryoides*	CBS 277.63	MH869889	MH858283	–	–	Vu et al. (2019)
	CBS 458.65	MH870308	MH858668	–	–	Vu et al. (2019)
	ANM 734	KX171943	KX171948			Miller (unpubl. data)
*Morinagamyces vermicularis*	CBS 303.81^T^	KP981427	MT904879	KP981609	KP981554	Harms et al. (2021b)
Podospora appendiculata*	CBS 212.97	AY780071	MH862644	AY780186	AY780129	Miller and Huhndorf (2005), Vu et al. (2019)
Podospora bullata*	CBS 115576^T^	MH874548	DQ166960	–	–	Bell et al. (2016), Vu et al. (2019)
Podospora didyma*	CBS 232.78	AY999100	AY999127	–	–	Cai et al. (2005)
Podospora fabiformis*	CBS 112043^T^	MK926843	MK926843	MK876805	–	Wang et al. (2019)
Podospora fibrinocaudata*	CBS 315.91^T^	MK926844	MK926844	MK876806	–	Wang et al. (2019)
Podospora serotina*	CBS 252.71	MH871878	MH860102			Vu et al. (2019)
*Pseudoechria curvicolla*	CBS 259.69	MH871036	MH859302	–	–	Vu et al. (2019)
*Pseudoechria decidua*	CBS 254.71^T^	MK926842	MK926842	MK876804	–	Wang et al. (2019)
*Pseudoechria longicollis*	CBS 368.52^T^	MK926847	MK926847	MK876809	–	Wang et al. (2019)
*Pseudoechria. prolifica*	CBS 250.71^T^	MK926848	MK926848	MK876810	–	Wang et al. (2019)
*Pseudoschizothecium atropurpureum*	SMH 2961	AY780056	–	–	AY780099	Miller and Huhndorf (2005)
	SMH 3073	AY780057	–	AY780160	AY780100	Miller and Huhndorf (2005)
Ramophialophora chlamydospora*	AUMC 11013^T^	KX446768	–	–	–	Moubasher et al. (2019)
Ramophialophora globispora*	CGMCC 3.17939^T^	KU746745	KU746699	KY883251	–	Zhang et al. (2017, 2018)
Ramophialophora humicola*	FMR 9523^T^	FR692337	FM955449	–	–	Madrid et al. (2010, 2011)
Ramophialophora petraea*	CGMCC 3.17953	KU746747	KU746701	KY883254		Zhang et al. (2017, 2018)
*Ramophialophora vesiculosa*	CBS 110629^T^	MH874452	MH862866	–	–	Vu et al. (2019)
*Rinaldiella pentagonospora*	CBS 132344^T^	KP981442	MH866007	KP981625	KP981570	Vu et al. (2019), Marin-Felix et al. (2020)
*Schizochlamydosporiella marina*	FMR 20114^T^	PP342613	PP273927	PP412577	–	Guerra-Mateo et al. (2024)
*Schizothecium aloides*	CBS 879.72	AY999097	AY999120	–	–	Cai et al. (2005)
*Schizothecium carpinicola*	CBS 228.87^T^	AY999095	AY999118	–	–	Cai et al. (2005)
*Schizothecium conicum*	CBS 434.50	MH868218	MH856702	–	–	Vu et al. (2019)
*Schizothecium curvisporum*	ATCC 36709	AY346300	–	AY780192	AY780136	Huhndorf et al. (2004), Miller and Huhndorf (2005)
*Schizothecium fimbriatum*	CBS 144.54	AY780075	AY999115	AY780189	AY780132	Cai et al. (2005), Miller and Huhndorf (2005)
*Schizothecium glutinans*	CBS 134.83	AY999093	AY999116	–	–	Cai et al. (2005)
*Schizothecium inaequale*	CBS 356.49^T^	MK926846	MK926846	MK876808	–	Wang et al. (2019)
*Schizothecium minicauda*	CBS 227.87	MH873757	MH862068	–	–	Vu et al. (2019)
*Schizothecium selenosporum*	CBS 109403^T^	MK926849	MK926849	MK876811	–	Wang et al. (2019)
*Schizothecium tetrasporum*	CBS 394.87	MH873776	MH862087			Vu et al. (2019)
Zopfiella attenuata*	CBS 266.77^T^	KP981445	MH861060	KP981628	KP981572	Vu et al. (2019), Marin-Felix et al. (2020)
Zopfiella erostrata*	CBS 255.71	AY999110	AY999133	–	–	Cai et al. (2005)
Zopfiella pleuropora*	CBS 518.70^T^	KP981450	MT784145	KP981633	KP981476	Marin-Felix et al. (2020)
*Zopfiella tabulata*	CBS 230.78	MK926854	MK926854	MK876816	–	Wang et al. (2019)
Zopfiella tardifaciens*	CBS 670.82^T^	MK926855	MK926855	MK876817	–	Wang et al. (2019)
*Zygopleurage zygospora*	SMH 4219	AY346306	–	–	AY780147	Huhndorf et al. (2004), Miller and Huhndorf (2005)
*Zygospermella insignis*	Lundqvist 2444	KF557698	–	–	KF557722	Kruys et al. (2015)
*Zygospermella insignis*	E00204312	KF557699	–	–	KF557723	Kruys et al. (2015)

1ATCC: American Type Culture Collection, Virginia, USA; AUMC: culture collection of the Assiut University Mycological Centre, Assiut, Egypt; CBS: Westerdijk Fungal Biodiversity Institute, Utrecht, the Netherlands; CGMCC: Chinese General Microbiological Culture Collection Center, Beijing, China; FMR: Facultat de Medicina, Reus, Spain; IMI: International Mycological Institute, CABI-Bioscience, Egham, UK; KRAM: Herbarium of the W. Szafer Institute of Botany, Polish Academy of Sciences, Kraków, Poland; MFLUCC: Mae Fah Luang University Culture Collection, Chiang Rai, Thailand; TRTC: Royal Ontario Museum, Toronto, Canada; ANM, AR, GJS, JF, HHB, Kruys, Lundqvist, MJR, Santesson, SMH: personal collections of Andrew N. Miller, Amy Rossman, Gary J. Samuels, Jacques Fournier, Harold H. Burdsal, Åsa Kruys, Nils Lundqvist, Michael J. Richardson, Sweden R. Santesson, and Sabine M. Huhndorf, respectively. T indicates ex-type strains. *Taxa with generic names applied in the broad sense (sensu lato), not necessarily reflecting molecular phylogenetic relationships.

### Fermentation and extraction

The fungal strain was grown on YM agar at 23 °C. Then, five pieces of the colonies were cut using a cork borer (1 cm diam.) and transferred into 200 mL of semi-viscous seed medium (SMYA, maltose 40 g/L, yeast extract 10 g/L, meat pep­tone 10 g/L, agar 4 g/L) contained in 500-mL Erlenmeyer flasks. The seed culture was incubated for 7 days at 23 °C under shaking conditions at 210 rpm. After incubation, a 2-mL aliquot was transferred into 40 Erlenmeyer flasks, each containing solid rice medium (BRFT, brown rice 28 g and 100 mL of base liquid [yeast extract 1 g/L, disodium tartrate dihydrate 0.5 g/L, KH2PO4 0.5 g/L] per flask). The cultures were incubated for 14 days at 23 °C in darkness. After incubation, the rice mycelia were covered with acetone and sonicated in an ultrasonic bath at 40 °C for 30 min. The acetone extract was separated from the mycelia by filtration through cellulose filter paper (MN 615 1/4 Ø 185 mm, Macherey-Nagel GmbH & Co. KG, Düren, Germany). The mycelia were extracted and filtered two additional times using the same procedure. The acetone extracts were combined, and the solvent was evaporated under reduced pressure at 40 °C using a rotary evaporator (Heidolph Instruments GmbH & Co. KG, Germany) connected to a vacuum pump (Vacuubrand GmbH & Co. KG, Wertheim am Main, Germany), resulting in an aqueous residue. This residue was extracted three times with an equal volume of ethyl acetate using a separatory funnel. The combined ethyl acetate extract was dried under vacuum at 40 °C and then dissolved in methanol for partitioning with an equal volume of heptane. Finally, the methanol phase was separated and dried under vacuum to afford the crude extract (1.4 g).

### Isolation of secondary metabolites

The crude extract was pre-fractionated using flash chromatography (Grace Reveleris^®^, Columbia, MD, USA) equipped with a 24-g silica cartridge. The mobile phase consisted of solvent A (DCM), solvent B (MeOH), and solvent C (a mixture of DCM/MeOH, 9:1). The gradient was set up as follows: 100% solvent A for 8 min, increasing to 25% solvent B over 20 min, maintained under isocratic conditions for 2 min, increased to 100% B over 10 min, followed by an increase to 10% solvent C in 2 min and held at 100% solvent C for 10 min. Eleven fractions (F1–F11) were collected.

Consequently, compounds **1**–**7** were separated using a PLC 2250 preparative HPLC system (Gilson, Middleton, WI, USA) with a Gemini^®^ 10-µm C18 110Å column (250 × 50 mm, 10 μm; Phenomenex^®^, Torrance, CA, USA) as the stationary phase.

Fraction F1 (260 mg) was purified under the following mobile conditions: solvent A (deionized H_2_O + 0.1% formic acid); solvent B (acetonitrile (MeCN) + 0.1% formic acid); flow rate: 40 mL/min; gradient as follows: isocratic at 5% B for 10 min, increase to 30% B over 30 min, then from 30% to 85% B over 10 min, and finally 100% B in 10 min to obtain **6** (6.93 mg, *t*_R_ = 48.0–49.0 min).

Fraction F5 (66 mg) was separated using the following conditions: solvent A (deionized H_2_O + 0.1% formic acid); solvent B (acetonitrile (MeCN) + 0.1% formic acid); flow rate: 10 mL/min; gradient, from 5 to 15% B over 10 min, then holding isocratically for 5 min, increased from 15 to 40% B over 40 min, held again at the condition for 5 min, followed by a final increase to 100% B over 5 min, and held at 100% for an additional 5 min to obtain **7** (1.78 mg, *t*_R_ = 54.4–55.0 min).

Fraction F7 (159 mg) was purified using the following conditions: solvent A (deionized H_2_O + 0.1% formic acid); solvent B (MeCN + 0.1% formic acid); flow rate: 40 mL/min; gradient, from 5 to 15% B over 10 min, followed by an increase from 15 to 40% B over 60 min, and finally reaching 100% B in 10 min to afford **4** (3.77 mg, *t*_R_ = 34.0–37.3 min) and **5** (0.88 mg, *t*_R_ = 41.0–44.0 min).

Fraction F8 (128 mg) was separated by the following conditions: solvent A (deionized H_2_O + 0.1% formic acid); solvent B (MeCN + 0.1% formic acid); flow rate: 50 mL/min; gradient: from 5 to 15% B over 10 min, then an increase from 15 to 70% B in 60 min, ending with 100% B for 10 min to obtain **1** (0.53 mg, *t*_R_ = 75.0–76.0 min), **2** (3.3 mg, *t*_R_ = 70.0–72.0 min), and **3** (1.04 mg, *t*_R_ = 67.3–68.0 min).

Papulacandin G (**1**): Light-brown amorphous solid; [α]_D_^20^ +670.0 (*c* 0.1, MeOH); UV/Vis (MeOH): *λ*_max_ = 230, 258 nm; NMR data (^1^H NMR: 700 MHz, ^13^C NMR: 175 MHz, methanol-*d*_4_) see Table 2; HR-(+)ESI-MS: *m/z* 845.4183 [M – H_2_O + H]^+^ (calcd. 845.4318 for C_45_H_65_O_15_^+^), 863.4286 [M + H]^+^ (calcd. 863.4424 for C_45_H_67_O_16_^+^), 885.4099 [M + Na]^+^ (calcd. 885.4243 for C_45_H_66_NaO_16_^+^); *t*_R_ = 12.02 min (HR-ESI-MS).

**Table 2.** ^1^H and ^13^C NMR data of **1**.

pos.	δ_C_,^a,b^ type	δ_H_,^a^ (*J*in Hz)	pos.	δ_C_,^a,e^ type	δ_H_,^b,e^ (*J*in Hz)
1	111.7, C		3‘‘	146.0, CH	7.26 dd (15.4, 11.0)
2	71.5, CH	4.37 d (10.0)	4‘‘	131.3, CH	6.31 ddt (15.0, 11.0)
3	76.1, CH	5.41 dd (10.0, 9.1)	5‘‘	141.3, CH	6.12 dd (15.0, 7.2)
4	77.3, CH	3.96 m	6‘‘	39.8, CH_2_	2.40 br t (6.6)
5	74.5, CH	3.99 m	7‘‘	77.4, CH	4.07 dd (6.6)
6	61.5, CH_2_	3.73 m	8‘‘	137.4, C	
7	73.6, CH_2_	*α* 5.00 d (12.5) *β* 5.05 d (12.5)	9‘‘	126.78, CH	5.99 d (10.8)
			10‘‘	126.81, CH	6.26 ddt (15.0, 10.8, 1.5)
8	145.3, C		11‘‘	135.9, CH	5.67 dd (15.0, 7.1)
9	116.3, C		12‘‘	31.3, CH_2_	2.12 m
10	161.4, C		13‘‘	37.3, CH_2_	*α* 1.21 m; *β* 1.42 m
11	99.7, CH	6.20 dd (2.0, 1.0)	14‘‘	35.0, CH	1.35 m
12	154.3, C		15‘‘	30.2, CH_2_	*α* 1.17 m; *β* 1.30 m
13	102.7, CH	6.22 d (1.9)	16‘‘	11.4, CH_3_	0.88 t (7.3)
1‘	105.1, CH	4.33 m	17‘‘	11.9, CH_3_	1.73 d (1.3)
2‘	72.5, CH	3.45 br d (4.5)	18‘‘	19.1, CH_3_	0.89 d (6.7)
3‘	74.4, CH	3.45 br d (4.5)	1‘‘‘	175.0, CO	
4‘	69.8, CH	3.73 m	2‘‘‘	34.8, CH_2_	2.38 t (7.6)
5‘	72.2, CH	3.51 m	3‘‘‘	25.7, CH_2_	1.62 m
6‘	64.1, CH_2_	*α* 4.11 dd (11.5, 7.0) *β* 4.14 dd (11.5, 6.3)	4‘‘‘-6‘‘‘	30.0-30.5, CH_2_	1.32-1.34 m
1‘‘	168.8, CO		7‘‘‘	23.4, CH_2_	1.33 m
2‘‘	121.4, CH	5.90 d (15.4)	8‘‘‘	14.1, CH_3_	0.91 t (7.0)

^a^ Measured in methanol-*d*_4_ at 700 MHz ^b^ Assignment confirmed by HMBC and HSQC spectra.

Mer-WF3010 (**2**): Light-brown amorphous solid; [α]_D_^20^ +36.2 (*c* 0.1, MeOH); UV/Vis (MeOH): *λ*_max_ = 230, 272, 310 nm; NMR data (^1^H NMR: 600 MHz, ^13^C NMR: 150 MHz, methanol-*d*_4_) comparable to those reported in the literature (Kaneto et al. 1993; Chiba et al. 1993); HR-(+)ESI-MS: *m/z* 839.3704 [M – H_2_O + H]^+^ (calcd. 839.3834 for C_45_H_59_O_15_^+^), 857.3804 [M + H]^+^ (calcd. 857.3954 for C_45_H_61_O_16_^+^), 879.3619 [M + Na]^+^ (calcd. 879.3774 for C_45_H_60_NaO_16_^+^); *t*_R_ = 11.04 min (HR-ESI-MS).

Papulacandin D (**3**): Light-brown amorphous solid; UV/Vis (MeOH): *λ*_max_ = 230, 259 nm; NMR data (^1^H NMR: 700 MHz, ^13^C NMR: 175 MHz, methanol-*d*_4_) comparable to those reported in the literature (Traxler et al. 1980); HR-(+)ESI-MS: *m/z* 557.2681 [M – H_2_O + H]^+^ (calcd. 557.2745 for C_31_H_41_O_9_^+^), 575.2781 [M + H]^+^ (calcd. 575.2851 for C_31_H_43_O_10_^+^), 597.2594 [M + Na]^+^ (calcd. 597.2670 for C_31_H_42_NaO_10_^+^); *t*_R_ = 9.56 min (HR-ESI-MS).

Penazaphilone M (**4**): Dark-yellow amorphous solid; [α]_D_^20^ +283.0 (*c* 1.0, MeOH); UV/Vis (MeOH): *λ*_max_ = 228, 342, 418 nm; NMR data (^1^H NMR: 500 MHz, ^13^C NMR: 125 MHz, methanol-*d*_4_) see Table 3; HR-(+)ESI-MS: *m/z* 418.1252 [M + H]^+^ (calcd. 418.1263 for C_18_H_25_ClNO_8_^+^), 440.1066 [M + Na]^+^ (calcd. 440.1083 for C_18_H_24_ClNNaO_8_^+^); *t*_R_ = 3.10 min (HR-ESI-MS).

**Table 3.** ^1^H and ^13^C NMR data of **4** and **5**.

pos.	4		5	
	δ_C_,^a^ type	δ_H_,^a^ (*J* in Hz)	δ_C_,^b^ type	δ_H_,^b^ (*J* in Hz)
1	151.7, CH	7.90 s	177.9, CO	
2	–	–	31.7, CH_2_	2.28 t (7.8)
3	90.1, C		26.6, CH_2_	α 1.85 m
				β 1.98 m
4	77.8, CH	4.66 s	76.5, CH	4.86 m (overlapped)
4a	146.9, C		–	–
5	101.1, C		73.6, CH	4.13 t (6.0)
6	191.1, CO		134.6, CH	5.74 ddd (15.6, 6.0, 0.8)
7	86.4, C		128.9, CH	5.80 ddd (15.6, 6.0, 0.9)
8	191.2, CO		78.8, CH	5.19 dd (6.0, 4.0)
8a	117.7, C		–	–
9	23.9, CH_3_	1.51 s	69.7, CH	3.87 dq (6.5, 4.0)
10	33.0, CH_2_	α 1.64 m	18.5, CH_3_	1.16 d (6.5)
		β 1.67 m		
11	27.5, CH_2_	α 1.62 m β 1.77 m	172.6, CO	
12	62.6, CH_2_	3.53 td (6.2, 1.4)	21.0, CH_3_	2.02 s
13	171.5, CO		–	–
14	20.1, CH_3_	2.12 s	–	–
15	58.6, CH_3_	3.52 s	–	–
1‘	52.3, CH_2_	α 3.50 overlapped β 3.87 ddd (14.0, 5.3, 4.0)	167.2, CO	
2‘	62.1, CH_2_	α 3.71 ddd (11.5, 7.4, 3.9) β 3.78 ddd (11.5, 5.3, 4.2)	123.6, CH	5.91 dq (15.5, 1.7)
3‘	–	–	146.7, CH	7.03 dq (15.5, 6.9)
4‘	–	–	18.1, CH_3_	1.90 dd (6.9, 1.7)

^a^ Measured in methanol-*d*_4_ at 125 MHz for ^13^C and 500 MHz for ^1^H. ^b^ Measured in methanol-*d*_4_ at 150 MHz for ^13^C and 600 MHz for ^1^H.

Cremenoic acid (**5**): Dark-yellow amorphous solid; [α]_D_^20^ +90.0 (*c* 0.1, MeOH); UV/Vis (MeOH): *λ*_max_ = 226, 260, 300, 345, 410 nm; NMR data (^1^H NMR: 600 MHz, ^13^C NMR: 150 MHz, methanol-*d*_4_) see Table 4; HR-(+)ESI-MS: *m/z* 327.1434 [M – H_2_O + H]^+^ (calcd. 327.1438 for C_16_H_23_O_7_^+^), 345.1537 [M + H]^+^ (calcd. 345.1544 for C_16_H_25_O_8_^+^), 367.1353 [M + Na]^+^ (calcd. 367.1363 for C_16_H_24_NaO_8_^+^); *t*_R_ = 3.82 min (HR-ESI-MS).

**Table 4.** Cytotoxicity and antimicrobial activity of compounds **1**–**7**.

Test cell line	IC_50_ (µM)							Positive control
	1	2	3	4	5	6	7	Epothilone B (µg/mL)
Mouse fibroblast (L929)	–	–	–	–	–	–	–	0.00098
Human endocervival adenocarcinoma (KB3.1)	–	–	–	26	–	–	–	0.000028
**Test microorganism**	**MIC (µg/mL)**							**Positive control (µg/mL)**
*Acinetobacter baumannii* (DSM 30008)	–	–	–	–	–	–	–	0.53 ^C^
*Bacillus subtilis* (DSM 10)	33.3	8.3	–	–	–	–	–	16.6 ^O^
*Candida albicans* (DSM 1665)	4.1	0.2	–	–	–	–	–	2.1 ^N^
*Chromobacterium violaceum* (DSM 30191)	–	–	–	–	–	–	–	0.83 ^G^
*Escherichia coli* (DSM 116)	–	–	–	–	–	–	–	0.42 ^G^
*Mucor hiemalis* (DSM 2656)	–	–	–	–	–	–	–	2.1 ^N^
*Mycolicibacterium smegmatis* (ATCC 700084)	–	–	–	–	–	–	–	0.1 ^K^
*Pseudomonas aeruginosa* (PA 14)	–	–	–	–	–	–	–	0.21 ^G^
*Rhodotorula glutinis* (DSM 10134)	–	–	–	–	–	–	–	1.0 ^N^
*Schizosaccharomyces pombe* (DSM 70572)	8.3	2.1	–	–	–	–	–	4.2 ^N^
*Staphylococcus aureus* (DSM 346)	66.6	16.6	–	–	–	–	–	0.42 ^G^
*Wickerhamomyces anomalus* (DSM 6766)	–	33.3	–	–	–	–	–	4.2 ^N^

IC_50_: half-maximal inhibitory concentration, µg/mL. MIC: minimum inhibitory concentration in µg/mL. “-“: No activity under test conditions (MIC > 66.6 µg/mL, IC_50_ > 37). C: Ciprofloxacin; G: Gentamicin; K: Kanamycin, N: Nystatin; O: Oxytetracycline.

Cremenolide (**6**): Light-yellow amorphous solid; [α]_D_^20^ +46.0 (*c* 0.18, CHCl_3_); UV/Vis (MeOH): *λ*_max_ = 226 nm; NMR data (^1^H NMR: 500 MHz, ^13^C NMR: 125 MHz, methanol-*d*_4_) comparable to those reported in the literature (Vinale et al. 2016); HR-(+)ESI-MS: *m/z* 309.1330 [M – H_2_O + H]^+^ (calcd. 309.1333 for C_16_H_21_O_6_^+^), 327.1433 [M + H]^+^ (calcd. 327.1438 for C_16_H_23_O_7_^+^), 349.1250 [M + Na]^+^ (calcd. 349.1258 for C_16_H_22_NaO_7_^+^); *t*_R_ = 6.24 min (HR-ESI-MS).

Aspinolide B (**7**): Light-yellow amorphous solid; [α]_D_^20^ -44.0 (*c* 0.1, MeOH); UV/Vis (MeOH): *λ*_max_ = 226 nm; NMR data (^1^H NMR: 600 MHz, ^13^C NMR: 150 MHz, methanol-*d*_4_) comparable to those reported in the literature (Fuchser and Zeeck 1997; Pilli et al. 2000); HR-(+)ESI-MS: *m/z* 267.1232 [M – H_2_O + H]^+^ (calcd. 267.1227 for C_14_H_19_O_5_^+^), 285.1333 [M + H]^+^ (calcd. 285.1334 for C_14_H_21_O_6_^+^), 307.1150 [M + Na]^+^ (calcd. 307.1152 for C_14_H_20_NaO_6_^+^); *t*_R_ = 3.52 min (HR-ESI-MS).

### Chromatography and spectral methods

Electrospray ionization mass spectra (ESI-MS) were measured using an UltiMate 3000 Series uHPLC (Thermo Fisher Scientific, Waltham, MA, USA) equipped with a C18 column (Acquity UPLC BEH, 1.7 μm, 2.1 × 50 mm; Waters, Milford, MO, USA) and connected to an amaZon Speed ESI ion trap MS (Bruker Daltonics, Bremen, Germany). The column temperature was maintained at 25 °C, and UV/Vis data were recorded with a diode-array detector (DAD) over a range of 190–600 nm. Crude extracts and pure compounds were dissolved at concentrations of 4.5 and 1.0 mg/mL, respectively, in a solution of acetone and methanol (1:1), with a sample injection volume of 2 μL. The solvents used were A (deionized H_2_O + 0.1% formic acid) and B (MeCN with 0.1% formic acid), with a constant flow rate of 0.6 mL/min. The mobile-phase gradient was as follows: 5% B for 0.5 min, increasing to 100% B over 20 min, followed by 10 min at 100% B.

Optical rotations were obtained using an MCP 150 circular polarimeter (Anton Paar, Seelze, Germany) at 20 °C, and UV/Vis spectra were recorded with a UV-2450 spectrophotometer (Shimadzu, Kyoto, Japan). Both measurements were performed using compounds dissolved in methanol.

All 1D and 2D nuclear magnetic resonance (NMR) spectra were acquired using an Avance III 700 spectrometer equipped with a 5-mm TCI cryoprobe (Bruker, ^1^H NMR: 700 MHz, ^13^C: 175 MHz, Billerica, MA, USA), an Avance III 600 spectrometer (Bruker, ^1^H NMR: 600 MHz, ^13^C: 150 MHz, Billerica, MA, USA), and an Avance III 500 spectrometer (Bruker, ^1^H NMR: 500 MHz, ^13^C: 125 MHz, Billerica, MA, USA). The chemical shifts (*δ*) were referenced to the solvent methanol-*d*_4_.

### Biological activity testing

The antimicrobial activity of all isolated metabolites was evaluated by determining the minimum inhibitory concentration (MIC), following the experimental procedures described by Charria-Girón et al. (2023). The evaluation included a panel of Gram-positive bacteria, including *Bacillus subtilis* (DSM 10), *Mycolicibacterium smegmatis* (ATCC 700084), and *Staphylococcus aureus* (DSM 346), as well as Gram-negative bacteria, such as *Acinetobacter baumannii* (DSM 30008), *Chromobacterium violaceum* (DSM 30191), *Escherichia coli* (DSM 1116), and *Pseudomonas aeruginosa* PA14 (DSM 19882). Additionally, five fungi were tested, including one filamentous strain, *Mucor hiemalis* (DSM 2656), and four yeasts, *Candida albicans* (DSM 1665), *Rhodotorula glutinis* (DSM 10134), *Schizosaccharomyces pombe* (DSM 70572), and *Wickerhamomyces anomalus* (DSM 6766). As a negative control, MeOH was used, while different positive controls were employed depending on the test organism. Nystatin (1 mg/mL) was used for the fungi tested. For *A. baumannii*, *M. smegmatis*, and *P. aeruginosa*, ciprofloxacin (0.25 mg/mL), kanamycin (0.1 mg/mL), and gentamicin (0.1 mg/mL) were used, respectively. Oxytetracycline was employed as a positive control at 0.1 mg/mL against the remaining bacterial strains, except for *B. subtilis*, for which a concentration of 1 mg/mL was used.

The cytotoxicity of all isolated metabolites was evaluated against two mammalian cell lines, namely human endocervical adenocarcinoma (KB 3.1) and mouse fibroblasts (L929), in 96-well plates following established protocols (Charria-Girón et al. 2023). After 5 days of incubation, the cells were stained with 3-(4,5-dimethyl-2-thiazolyl)-2,5-diphenyl-2*H*-tetrazolium bromide (MTT; M2128, Sigma-Aldrich, Deisenhofen, Germany). Viable cells converted this dye into a purple derivative, the intensity of which was quantified relative to untreated control cells (100% viability) for each tested compound concentration. For quantification, a microplate reader set at 595 nm was used to measure and calculate the percentage of cell viability. From these data, the half-maximum inhibitory concentration (IC_50_), expressed in μM, was determined.

### TDDFT calculations

Conformational sampling for compound **4** was performed using the default iMTD-GC protocol implemented in the Conformer–Rotamer Ensemble Sampling Tool (CREST) (Pracht et al. 2024). The resulting ensemble of conformers was clustered to determine the most representative geometries. Each selected structure underwent geometry optimization and vibrational frequency analysis at the B3LYP/6-31G* level of theory. Based on these optimized geometries, time-dependent density functional theory (TDDFT) calculations were performed using the CAM-B3LYP/TZVP functional to explore the lowest 50 excited states. The generated electronic circular dichroism (ECD) spectra of the conformers were averaged according to Boltzmann populations and visualized using SpecDis 1.71, employing Gaussian band shapes with a sigma value of 0.20–0.30 eV (Bruhn et al. 2013). All quantum chemical computations, including geometry optimization and TDDFT simulations, were performed in methanol using the integral equation formalism polarizable continuum model (IEFPCM) (Miertuš and Tomasi 1981; Tomasi and Persico 1994), as implemented in Gaussian09 software (Frisch et al. 2009).

## Results and discussion

### Phylogenetic analysis

**Figure 1.**
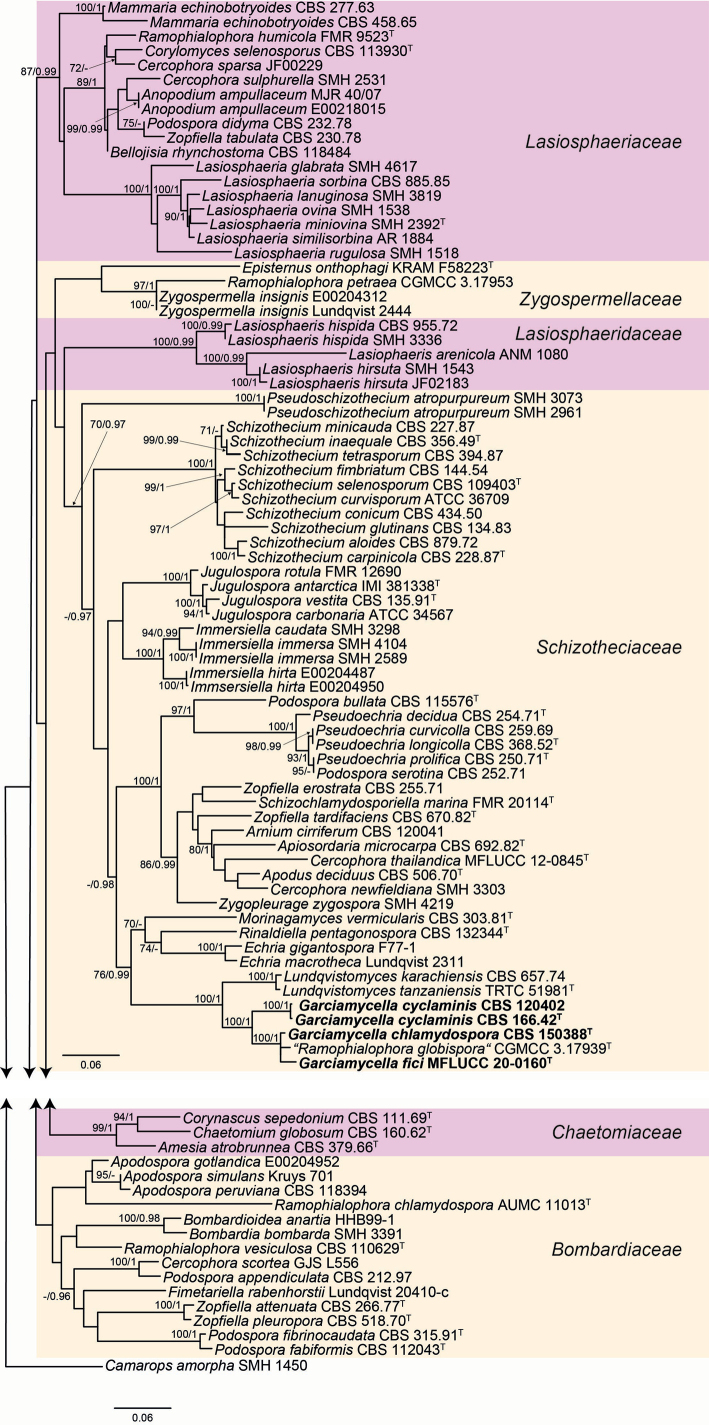
Randomized accelerated maximum likelihood (RAxML) phylogram obtained from the combined sequences of the internal transcribed spacer region (ITS), the nuclear rDNA large subunit (LSU), and fragments of RNA polymerase II subunit 2 (*rpb*2) and β-tubulin (*tub*2) genes of selected strains belonging to the order *Sordariales*. Bootstrap support values ≥ 70 and Bayesian posterior probability values ≥ 0.95 are indicated along branches. Branch lengths are proportional to distance. Novel taxa proposed in the present study are bold. Ex-type strains of the different species are indicated with ^T^.

The lengths of the individual alignments used in the combined dataset were 503 bp (ITS), 886 bp (LSU), 978 bp (*rpb*2), and 618 bp (*tub*2), and the final total alignment comprised 2,985 bp. The phylogenetic tree obtained from the RAxML analysis of the combined dataset, including bootstrap support and Bayesian posterior probability values at the nodes, is shown in Fig. 1. The RAxML tree agreed with the topology of the tree generated by the Bayesian analysis. Our strain was located in a well-supported clade (70 bs/0.97 pp) within the family *Schizotheciaceae*, together with the type strains of “*Cercophora fici*” (MFLUCC 20-0160), “*Ramophialophora globispora*” (CGMCC 3.17939), *Phialophora cyclaminis* (CBS 166.42), and “*Cercophora mirabilis*” (CBS 120402). However, CBS 120402 is not morphologically similar to the latter species (see detailed discussion in the Taxonomy section). Since the type species of *Ramophialophora*, *R. vesiculosa*, belongs to the family *Bombardiaceae*, the new genus *Garciamycella* is here erected to accommodate this monophyletic lineage. Moreover, the new combinations *G. fici* and *G. cyclaminis* are proposed, with amended morphological descriptions, and the new species *G. chlamydospora* is introduced based on our soil-borne fungus. *Ramophialophora globispora* is excluded from the new genus *Garciamycella* and from *Ramophialophora* due to the incongruences discussed in detail in the following section. Furthermore, none of the other species previously described in *Ramophialophora* clustered with the type species, indicating that these taxa should be reassigned to other existing or newly established genera within different families of the order *Sordariales*.

### Taxonomy

#### Garciamycella

Taxon classificationAnimaliaSordariales Schizotheciaceae

Y. Marín & Hern.-Restr. gen. nov.

FD2F4DCA-91F2-5C4B-BF62-5A75463482BA

861139

##### Type species.

*Garciamycella chlamydospora* Y. Marín & Hern.-Restr.

##### Etymology.

Named in honor of the mycologist Dania García, an expert on the order *Sordariales*.

##### Description.

**Sexual morph. *Ascomata*** ostiolate, solitary or aggregated, superficial, dark brown to black, globose to pyriform, coriaceous, with papillate neck, wall of *textura angularis*. ***Asci*** cylindrical, stipitate, 8-spored, with an apical ring. Paraphyses filamentous, hyaline. ***Ascospores*** at first one-celled, hyaline, ellipsoidal, becoming transversely septate and two celled; upper cell brown to dark brown, obovoid to globose, smooth-walled, 1–2-septate, less frequently aseptate, truncate at the base, with an apical or lateral germ pore; lower cell hyaline to pale brown, conical, smooth-walled, aseptate or commonly 1–3-septate. **Asexual morph. *Conidiophores*** mostly reduced to conidiogenous cells, irregularly grouped. ***Conidiogenous cells*** phialidic, hyaline to pale brown, cylindrical to lageniform, with a visible collarette. ***Conidia***, sessile, borne singly along the vegetative hyphae, hyaline, spherical to subspherical, or ovate to elongate, smooth-walled. ***Aleuriconidia*** present along the hyphae, hyaline, subglobose to obovoid. ***Chlamydospores*** present or absent.

##### Notes.

*Garciamycella* is introduced here to accommodate a new species collected from the Netherlands, together with *Cercophora fici*, *Phialophora cyclaminis*, and a strain previously identified as *C. mirabilis*. The type strain of *Ramophialophora globispora* is also located within the monophyletic lineage representing this new genus, but it is not transferred due to incongruences in the morphological characterization. The structures described and illustrated by Zhang et al. (2017) more closely resemble penicillate fungi, which have not been previously reported in the order *Sordariales*. Therefore, this species is not transferred to *Garciamycella* until further studies confirm that the described fungus corresponds to the strain from which the sequences were generated.

The new genus is characterized by the production of ostiolate ascomata and 2-celled ascospores, with both the upper and lower cells being septate. Its closest phylogenetic relative is *Lundqvistomyces*, which differs by producing ascospores with aseptate upper and lower cells (Marin-Felix et al. 2020). The asexual morph of *Garciamycella* consists of semi-macronematous conidiophores and conidiogenous cells with conspicuous collarettes that produce globose, hyaline conidia grouped in masses, which additionally form hyaline aleuriconidia along the vegetative hyphae. This latter type of conidia can also be found in other genera of *Schizotheciaceae*, e.g., *Naviculispora* and *Jugulospora*. However, the only genus presenting both conidiogenous cells with conspicuous collarettes and sessile conidia is *Pseudoschizothecium*. Both genera present ascospores with lower cells that are up to 3-septate and upper cells that are 1-septate, but this latter characteristic is more common in *Garciamycella* than in *Pseudoschizothecium*. The main characteristic distinguishing the two genera is the ascomatal neck, which is warty in *Pseudoschizothecium* and papillate in *Garciamycella*. It is of note that ascomatal morphology has been shown to be more informative for phylogenetic inference than ascospore morphology in the *Sordariales* (Miller and Huhndorf 2005).

#### Garciamycella chlamydospora

Taxon classificationAnimaliaSordariales Schizotheciaceae

Hern.-Restr. & Y. Marín sp. nov.

1DB0F4EF-43EA-5261-AA5B-D1E0F133D223

861140

Fig. 2

##### Etymology.

From Greek chlamydos-, cloak, and from Latin – spora, spore.

**Figure 2.**
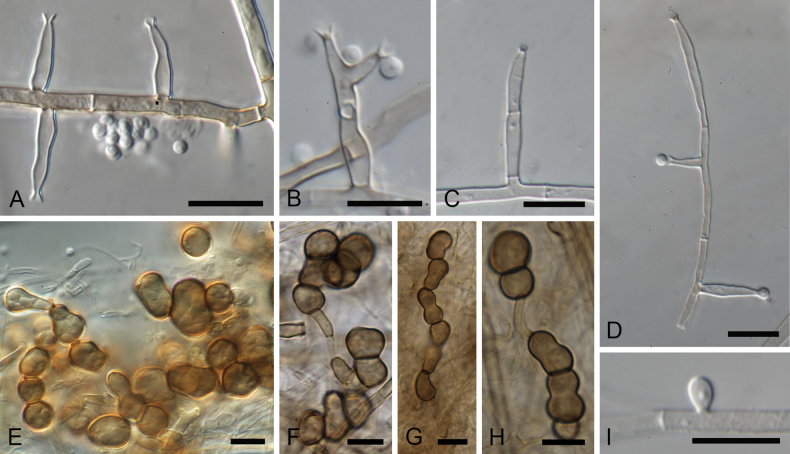
*Garciamycella chlamydospora* (ex-type strain CBS 150388). **A–D** Conidiogenous cells and conidia. **E–H** Chlamydospores. **I** Aleuriconidia. Scale bars: 10 µm.

##### Type material.

The Netherlands, Rotterdam, isolated from garden soil, 2017, col. F., M. & L. Blok, isol. A. Giraldo JW184011 (holotype designated here CBS H-25778, ex-type strain CBS 150388).

##### Description.

***Vegetative hyphae*** septate, branched, hyaline to subhyaline, smooth- and thin-walled, 1.5–3 µm wide. **Asexual morph. *Conidiophores*** semi-macronematous, mostly reduced to conidiogenous cells. ***Conidiogenous cells*** phialidic, intercalary, terminal or lateral, cylindrical, subcylindrical to lageniform, straight to flexuous, subhyaline or pale brown, 8.5–25 µm long, 2–3 wide at the base, 0.5–1 µm wide at the apex, bearing conspicuous collarettes. ***Conidia*** one-celled, smooth- and thin-walled, globose to subglobose, 2–2.5 µm diam., guttulate. ***Aleurioconidia*** borne singly along the vegetative hyphae, hyaline, subglobose to ovoid, smooth-walled, 3.5–5 × 2.5–3 µm. ***Chlamydospores*** multicellular, starting in chains, later grouping irregularly, composed of globose, ellipsoidal, or irregular shape, sometimes constricted, brown to dark brown cells, up to 17 µm diam. **Sexual morph** not observed.

##### Cultural characterization.

Colonies on OA attaining 17–23 mm at 25 °C in 14 d, velvety to slightly cottony, umbonate, margins regular, greyed-orange (165A) with white center; reverse grey (201A). Colonies on PCA reaching 48–71 mm at 25 °C in 14 d, velvety to cottony, umbonate, margins fimbriate, grey (201A–B) with greyed-orange (165A–D) margins; reverse brown (200A–D). Colonies on PDA attaining 17–22 mm at 25 °C in 14 d, velvety to slightly cottony, umbonate, margins fringed, white to greyed-white (156A–D) with grey (201A) center; reverse greyed-orange (165C–D) with darker greyed-orange (165A) center. Colonies on MEA attaining 14–22 mm at 25 °C in 14 d, velvety to slightly cottony, umbonate, margins fringed, greyed-yellow (156A–C) to greyed-yellow (161A–D); reverse greyed-yellow (161A–D) with greyed-orange (165A) center.

##### Notes.

This new species, isolated from soil in the Netherlands, is characterized by the production of an exclusively asexual state. Similar to *G. cyclaminis*, it forms conidia singly along the vegetative hyphae as well as on semi-macronematous conidiophores, with phialides bearing conspicuous collarettes. Both species can be distinguished by the production of brown, thick-walled chlamydospores in *G. chlamydospora*, which are absent in *G. cyclaminis*, as well as by the smaller aleuriconidia in *G. chlamydospora*. Moreover, *G. cyclaminis* produces a sexual morph. Both type strains of *G. cyclaminis* and *G. chlamydospora* were isolated from the Netherlands; however, *G. cyclaminis* was isolated from plant material, with an additional strain reported from herbivorous animal dung in Australia.

#### Garciamycella cyclaminis

Taxon classificationAnimaliaSordariales Schizotheciaceae

(J.F.H. Beyma) Hern.-Restr. & Y. Marín comb. nov.

35E8D489-8311-547F-AFBD-13A1EE187824

861148

Fig. 3

##### Basionym.

*Phialophora cyclaminis* J.F.H. Beyma, Antonie van Leeuwenhoek 8: 115. 1942.

**Figure 3.**
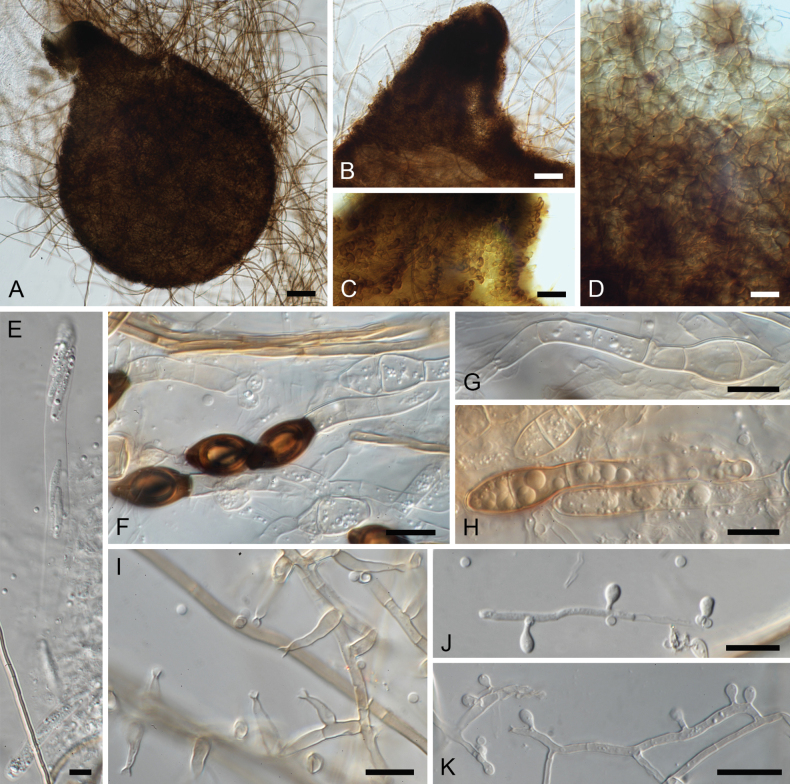
*Garciamycella cyclaminis* (CBS 120402). **A** Ascoma. **B, C** Papillate neck. **D** Ascomatal wall of textura angularis. **E** Asci. **F–H** Ascospores. **I** Conidiogenous cells and conidia. **J, K** Sessile conidia. Scale bars: 10 µm.

##### Type material.

The Netherlands, Aalsmeer, from *Cyclamen persicum*, Oct. 1942, J.W. Roodenburg (holotype CBS H-7579, ex-type strain CBS 166.42).

##### Additional material examined.

Australia, Victoria, Eucalyptus forest near Healesville, Wallaby dung, 24 Jul. 1998, A. Bell, CBS 120402.

##### Description.

***Vegetative hyphae*** composed of hyaline to pale brown, smooth, septate hyphae 2–3.5 µm diam. **Sexual morph. *Ascomata*** 176.5–407.5 × 150.5–382 µm, ostiolate, solitary or aggregated, superficial, dark brown to black, globose to subglobose, membranaceous, with papillate neck up to 237.5 µm, wall of *textura angularis*. ***Asci*** cylindrical, stipitate, 8-spored, with an apical ring, scarce, fast evanescent. ***Paraphyses*** filamentous, hyaline. ***Ascospores*** at first one-celled, hyaline, ellipsoidal, becoming transversely septate and two celled; upper cell brown to dark brown, obovoid, smooth-walled, 1–2-septate, less frequently aseptate, truncate at the base, with an apical to lateral germ pore, 15–21 × 7–9.5 µm; lower cell hyaline to pale brown, conical, smooth-walled, aseptate or commonly 1–2(–3)-septate, 26.5–40.5 × 4–6 µm. **Asexual morph. *Conidiophores*** semi-macronematous, often reduced to conidiogenous cells, or septate, branched, subhyaline to pale brown, smooth-walled, with cell walls usually thicker than those of the vegetative hyphae. ***Conidiogenous cells*** phialidic, terminal, intercalary or lateral, mostly lageniform, sometimes subcylindrical, 10–32 µm long, 2–3 µm wide at the base, 0.5–1 µm wide at the apex, subhyaline to pale brown, bearing conspicuous collarettes. ***Conidia*** one-celled, hyaline, smooth- and thin-walled, globose to subglobose, 2–2.5(–3) µm diam., guttulate. ***Aleuriconidia*** borne singly along the vegetative hyphae, hyaline, subglobose to obovoid or ellipsoidal, smooth-walled, 3.5–8 × 2–3 µm. ***Chlamydospores*** not observed.

##### Cultural characterization.

Colonies on OA attaining 40–45 mm at 25 °C in 14 d, velvety to cottony, slightly raised to convex with papillate surface, grey (201A–D), margin regular and darker (greyed orange (177A)) or irregular to fimbriate; reverse greyed-green (197A–B to 195A–B), margin brown (200C–D) regular or crenate. Colonies on PCA reaching 38–48 mm at 25 °C in 14 d, flat with moderate amount of aerial mycelium restricted to the center, other mycelium submerged, greyed orange (177A) to grey (201C), margin irregular, fimbriate; reverse greyed-orange (165B to 177A). Colonies on PDA attaining 40–45 mm at 25 °C in 14 d, velvety to slightly cottony, slightly raised to convex with papillate surface, greyed orange (177A), brown (200A–B) to the periphery, margin regular, fimbriate to irregular and lobate; reverse brown (200A–B). Colonies on MEA attaining 35–45 mm at 25 °C in 14 d, cottony, slightly raised to convex with papillate surface, greyed orange (177A) to grey (201C), margins regular, fimbriate to crenate, greyed-orange (165B) to brown (200B); reverse brown (200A–B).

##### Notes.

The type strain of *Phialophora cyclaminis* was located within the monophyletic lineage representing *Garciamycella* and is therefore here transferred to this new genus. Moreover, it clustered without phylogenetic distance from CBS 120402, which was previously considered a reference strain for *C. mirabilis*, delimiting the genus to the lineage including this strain, as this is the type species of *Cercophora* (Huang et al. 2021). However, as previously discussed, this strain did not sporulate and was never examined morphologically (Marin-Felix and Miller 2022). In the present study, the fungus sporulated, allowing a detailed morphological analysis. Our observations revealed that the strain produces ascospores with septate upper and lower cells, a character not reported in *C. mirabilis* (Fuckel 1870). Therefore, this strain is not suitable as a reference strain for the type species of *Cercophora*.

The anamorph of *G. cyclaminis* is similar to that of *G. chlamydospora* (see morphological comparison above). In contrast, the teleomorph is similar to that observed in *G. fici*. The two species can be differentiated by the size of the ascomata (400–600 × 350–500 µm in *G. fici* vs. 176.5–407.5 × 150.5–382 µm in *G. cyclaminis*). Moreover, an anamorph has not been observed in *G. fici*.

#### Garciamycella fici

Taxon classificationAnimaliaSordariales Schizotheciaceae

(Tennakoon, C.H. Kuo & K.D. Hyde) Y. Marín & Hern.-Restr. comb. nov.

C3596473-CDBC-5F47-A427-E01D2E371A17

861234

##### Basionym.

*Cercophora fici* Tennakoon, C.H. Kuo & K.D. Hyde, Fungal Diversity 108: 186. 2021.

##### Notes.

This species produces a teleomorph stage similar to that of *G. cyclaminis*, consisting of ostiolate ascomata and ascospores with septate upper and lower cells. Although this septation is not mentioned in the original description of the taxon, it can be readily observed in the illustrations (Tennakoon et al. 2021). See the morphological differences discussed in the Notes section for *G. cyclaminis*. The upper cell is described and illustrated as hyaline; however, this may be a consequence of immaturity, as pigmentation develops at later stages.

### Isolation and structure elucidation of secondary metabolites

**Figure 4.**
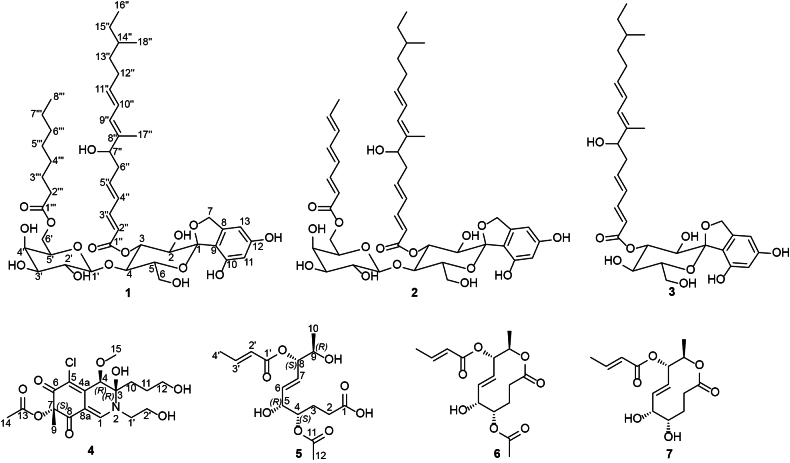
Chemical structures of **1**–**7**.

**Figure 5.**
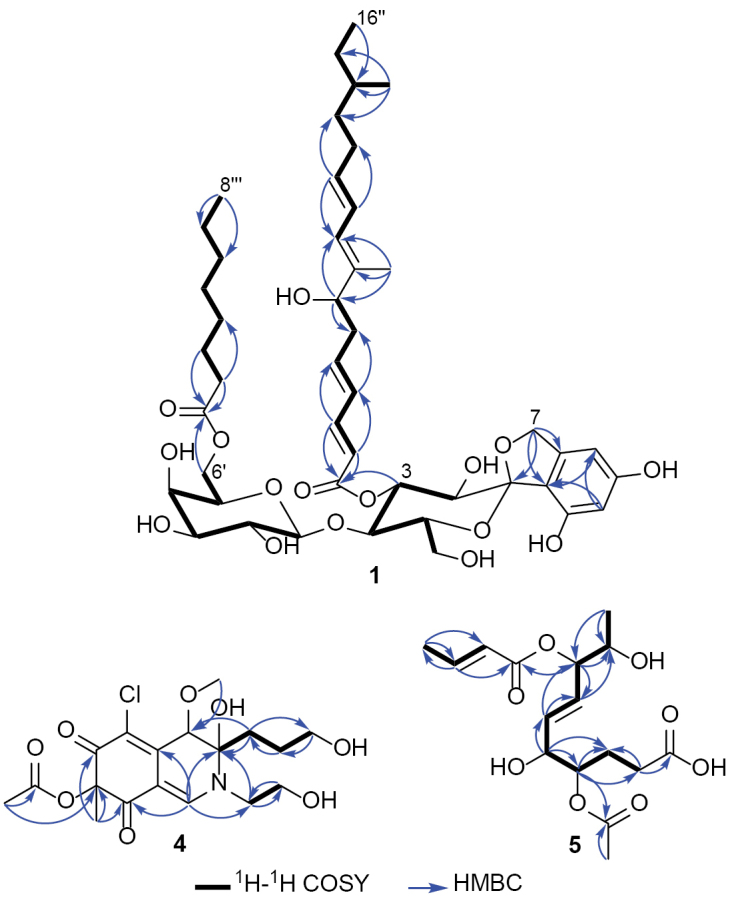
Key ^1^H–^1^H COSY and HMBC correlations of **1**, **4**, and **5**.

Compound **1** (Fig. 4) was isolated as a light-brown amorphous solid. Its molecular formula was established as C_45_H_66_O_16_ based on the HR-ESI-MS spectrum (Suppl. material 1: fig. S2) that revealed a protonated molecular ion peak at *m/z* 863.4286 [M + H]^+^ (calculated 863.4424) and a sodium adduct at *m/z* 885.4099 [M + Na]^+^ (calculated 885.4243), thus indicating thirteen degrees of unsaturation. The ^1^H and ^13^C NMR spectral data of **1** (Table 2) revealed two different sets of aliphatic methines and methylenes that were directly correlated via the ^1^H–^1^H COSY spectrum (Fig. 5, Suppl. material 1: fig. S4), indicating the presence of two sugar residues in **1**. In addition, the ^1^H–^1^H COSY spectrum of **1** also revealed two spin systems as follows: 1) four conjugated olefinic protons H-2’’/H-3’’/H-4’’/H-5’’ then to an aliphatic methylene (H_2_-6’’) and an oxygenated methine (H-7’’); 2) three conjugated olefinic protons H-9’’/H-10’’/H-11’’ extending over two methylenes (H_2_-12’’ and H_2_-13’’), an aliphatic methine (H-14’’), a methylene (H_2_-15’’), and a terminal triplet methyl group (H_3_-16’’).

A literature search of **1** revealed its close structural similarity to a family of potent spirocyclic diglycoside antifungal metabolites comprising papulacandins A–D, chaetiacandin, L-687,781, and Mer-WF3010, formerly reported from *Apiospora sphaerosperma* (syn. *Papularia sphaerosperma*) (Traxler et al. 1977, 1980), *Monochaetia dimorphospora* (Komori et al. 1985; Komori and Itoh 1985), *Tainosphaeria simplex* (syn. *Dictyochaeta simplex*) (VanMiddlesworth et al. 1991a, 1991b), and *Phialophora cyclaminis* (Kaneto et al. 1993; Chiba et al. 1993), which is here transferred to *Garciamycella*. A detailed comparison of 1D (^1^H and ^13^C) and 2D (^1^H–^1^H COSY, HMBC, HSQC, and ROESY) NMR spectral data of **1** (Table 2; Figs 4, 5Suppl. material 1: fig. S3–S7) with the measured and reported data of Mer-WF3010 (**2**) (Chiba et al. 1993) revealed a close coherence of the chemical shift values, apart from the absence of a conjugated three–double bond spin system extending from H-2’’’ to H-7’’’.

This sole difference explains the higher molecular weight of **1** compared with **2** by 6 Da and, therefore, indicates the presence of one polyunsaturated and one saturated fatty acid residues. The close similarity of the ¹H and ¹³C NMR data of the sugar moieties in **1** to those reported for papulacandins (Traxler et al. 1980) and Mer-WF3010 (**2**) (Chiba et al. 1993), together with their presumed common biosynthetic origin, suggests that the disaccharide unit in **1** is composed of glucose and galactose residues linked via a *β-trans-*1,4 linkage. The HMBC spectrum of **1** (Fig. 5, Suppl. material 1: fig. S5) confirmed the positions of the two fatty acyl moieties in its structure through key correlations from H-3 at δ_H_ 5.41 (dd, *J* = 10.0, 9.1 Hz) to C-1’’ (δ_C_ 168.8) and from H_2_-6’ at δ_H_ 4.11/4.14 to C-1’’’ (δ_C_ 175.0). Accordingly, the two fatty acyl moieties in **1** are positioned at C-3 and C-6’ of the glucosyl and galactosyl moieties, respectively. Based on these results, compound **1** was identified as a previously undescribed spirocyclic diglycoside derivative, named papulacandin G.

Compounds **2** and **3** were obtained as light-brown amorphous solids, with their molecular formulas determined as C_45_H_60_O_16_ and C_31_H_42_O_10_, respectively, based on their HR-ESI-MS data (Suppl. material 1: figs S9, S17). Their chemical structures were elucidated through comprehensive 1D and 2D NMR spectral analyses (Suppl. material 1: figs S10–S15, S18–S23) and by comparison with the reported literature, which ultimately identified them as Mer-WF3010 (**2**) (Kaneto et al. 1993; Chiba et al. 1993) and papulacandin D (**3**) (Traxler et al. 1977, 1980), respectively.

**Figure 6.**
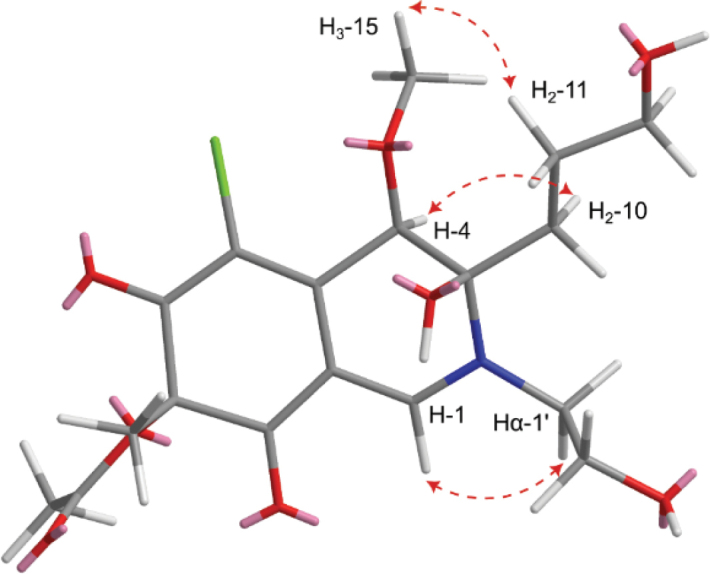
Key ROESY correlations of **4**.

**Figure 7.**
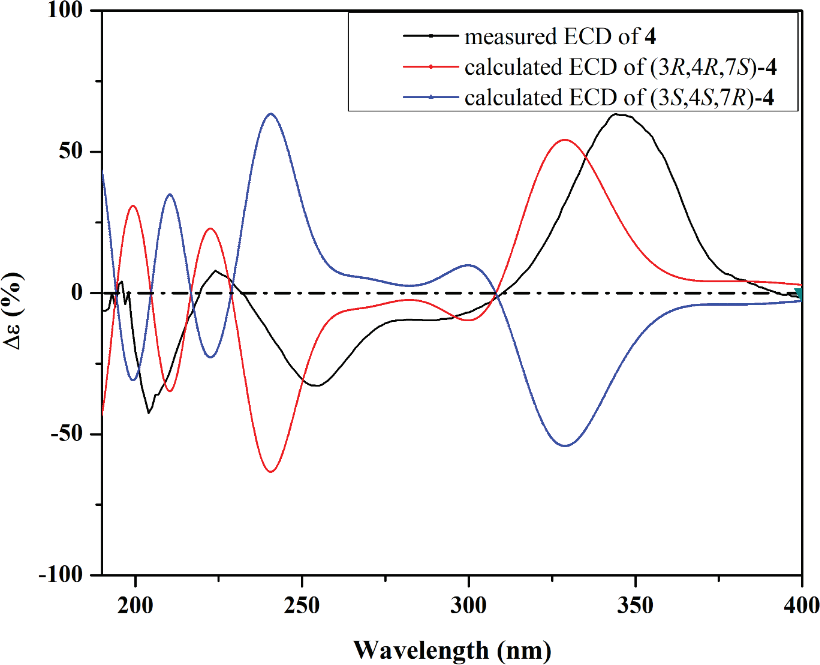
Measured and calculated ECD spectra of **4**.

Compound **4** was purified as a dark-yellow amorphous solid. The HR-ESI-MS spectrum (Suppl. material 1: fig. S25) revealed a protonated molecular ion peak at *m/z* 418.1252 [M + H]^+^ (calculated 418.1263) and a sodium adduct at *m/z* 440.1066 [M + Na]^+^ (calculated 440.1083). It also revealed a cluster of two protonated isotopic molecular ion peaks at *m/z* 418.1252 and 420.1224 in a 3:1 ratio that suggested the presence of one chlorine atom in its structure. These results determined its molecular formula as C_18_H_24_ClNO_8,_ indicating seven degrees of unsaturation. The ^13^C NMR and HSQC spectra of **4** (Table 3, Suppl. material 1: figs S27, S30) revealed 18 carbon resonances distinguished into eight unprotonated, including three carbonyl groups (δ_C_ 191.2, 191.1, 171.5, 146.9, 117.7, 101.1, 90.1, 86.4), two methines (δ_C_ 151.7, 77.8), five methylenes (δ_C_ 62.6, 62.1, 52.3, 33.0, 27.5), and three methyls, including one oxygenated methyl group (δ_C_ 58.6, 23.9, 20.1). The obtained results interpreted five degrees of unsaturation and thus indicated that compound **4** is a bicyclic compound. The ^1^H NMR, ^1^H–^1^H COSY, and HSQC spectra of **4** (Table 3, Fig. 5, Suppl. material 1: fig. S28, S30) revealed two spin systems: 1) between two diastereotopic deshielded methylene groups at δ_H_ 3.50/3.87 (δ_C_ 52.3; H_2_-1’) and 3.71/3.78 (δ_C_ 62.1; H_2_-2’); 2) extending over three methylene groups recognized into two diastereotopic at δ_H_ 1.64/1.67 (δ_C_ 33.0; H_2_-10) and 1.62/1.77 (δ_C_ 27.5; H_2_-11) ending at a deshielded oxygenated methylene group at δ_H_ 3.53 (δ_C_ 62.6; H_2_-12). In addition, the ^1^H NMR and HSQC spectra of **4** (Table 3, Fig. 5, Suppl. material 1: fig. S30) also revealed two deshielded olefinic and aliphatic protons at δ_H_ 7.90 and 4.66 that were directly correlated to carbon resonances at δ_C_ 151.7 (C-1) and 77.8 (C-4), respectively. Based on the obtained results of **4** and the literature search, it was suggested to belong to the chloroazaphilone class of metabolites resembling isochromophilones (Matsuzaki et al. 1995; Michael et al. 2003; Kanokmedhakul et al. 2006; McMullin et al. 2013; Jansen et al. 2013; Hematsin et al. 2016; Lu et al. 2018), penazaphilones (Tang et al. 2019; Zhang et al. 2023), and eupenicilazaphilones (Gu et al. 2018; Wang et al. 2021). The depicted structure of **4** (Fig. 4) was further confirmed via the HMBC spectrum (Fig. 5, Suppl. material 1: fig. S29) that revealed key correlations from two singlet methyl groups at δ_H_ 1.51 (H_3_-9) and 2.12 (H_3_-14) to two carbon atoms at δ_C_ 86.4 (C-7) and 171.5 (C-13), respectively. Moreover, the HMBC (Fig. 5, Suppl. material 1: fig. S29) revealed key correlations from a methoxy group at δ_H_ 3.52 (H_3_-15) and H_2_-10 to C-4 (δ_C_ 77.8) together with those from H-1 to C-3 (δ_C_ 90.1), C-4a (δ_C_ 146.9), C-8 (δ_C_ 191.2), and C-1’ (δ_C_ 52.3) confirming the depicted structure of 4 (Fig. 4).. The relative configuration of **4** was determined through acquiring its ROESY spectrum (Fig. 6, Suppl. material 1: fig. S31) that revealed key ROE correlations between H-4 and H_2_-10, suggesting their existence toward the same face of the molecule, while the H_3_-15 and 3-OH are supposedly directed toward the opposite face of the molecule. The absolute configuration of **4** was determined through the comparison of its measured and calculated TDDFT-ECD spectra (Fig. 7), which revealed a close coherence along the whole range with the calculated spectrum of the (3*R*,4*R*,7*S*) configuration.

Compound **5** was obtained as a dark-yellow amorphous solid. Its molecular formula was determined as C_16_H_24_O_8,_ indicating five degrees of unsaturation based on the HR-ESI-MS results (Suppl. material 1: fig. S33), revealing a protonated molecular ion peak and a sodium adduct at *m/z* 345.1537 [M + H]^+^ (calculated 345.1544) and 367.1353 [M + Na]^+^ (calculated 367.1363), respectively. The ^13^C and ^1^H NMR spectral data of **5** (Table 3) revealed characteristic signals ascribed for a crotonyl group as follows: δ_C_ 167.2 (C-1’), 123.6 (C-2’), 146.7 (C-3’), 18.1 (C-4’), δ_H_ 5.91 (dq, *J* = 15.5, 1.7 Hz, H-2’), 7.03 (dq, *J* = 15.5, 6.9 Hz, H-3’), 1.90 (dd, *J* = 6.9, 1.7 Hz, H-4’) (Malmierca et al. 2015). In addition, the ^13^C and ^1^H NMR spectral data of **5** (Table 3) revealed the presence of an *E*-configured olefinic bond: δ_C_ 134.6 (C-6), 128.9 (C-7), δ_H_ 5.74 (ddd, *J* = 15.6, 6.0, 0.8 Hz, H-6), and 5.80 (ddd, *J* = 15.6, 6.0, 0.9 Hz, H-7) that revealed COSY correlations (Fig. 5, Suppl. material 1: fig. S36) to two oxygenated methine protons at δ_H_ 4.13 (t, *J* = 6.0 Hz, H-5) and 5.19 (dd, *J* = 6.0, 4.0 Hz, H-8), respectively. A literature search of **5** supported by the obtained results suggested its chemical structure to be similar to 10-membered lactone derivatives such as aspinolides (Fuchser and Zeek 1997; Malmierca et al. 2015). A detailed comparison of its ^1^H and ^13^C NMR data with the reported literature revealed a close coherence to the respective data reported for cremenolide (**6**) (Vinale et al. 2016) that was also isolated and identified in this study. Further confirmation of the depicted structure of **5** was provided by acquiring its HMBC (Fig. 5, Suppl. material 1: fig. S37), which revealed the presence of an acetyl group via the key correlation from a singlet methyl group at δ_H_ 2.02 (s, H_3_-12; δ_C_ 21.0) to a carboxyl carbon at δ_C_ 172.6 (C-11) and in turn was correlated to a methine proton at δ_H_ 4.86 (H-4). The HMBC spectrum of **5** (Fig. 5, Suppl. material 1: fig. S37) also revealed a key correlation from H-8 to C-1’, indicating the binding of the crotonyl group at C-8. Based on the different molecular weights and molecular formulas, **5** was concluded to represent an opened-ring derivative of **6**, featuring a terminal free carboxylic acid moiety at δC 177.9 (C-1). The opened-ring structure of **5** hindered the observation of key ROE correlations required to assign the relative configurations of the four chiral carbon atoms (C-4, C-5, C-8, and C-9). A similar limitation was encountered when attempting to determine the absolute configuration of **5** through comparison of its experimental and calculated TDDFT-ECD spectra. However, the close structural resemblance between **5** and other 10-membered lactones, such as cremenolide (**6**) (Vinale et al. 2016) and aspinolides, including aspinolide B (**7**) (Fuchser and Zeek 1997), suggests that these compounds are produced through the same biosynthetic pathway and likely adopt a similar absolute configuration (4*S*,5*R*,8*S*,9*R*). Accordingly, compound **5** was identified as a previously undescribed opened-ring cremenolide derivative, named cremenoic acid.

### Antimicrobial and cytotoxic activities of compounds 1–7

The isolated compounds were tested against a broad panel of microorganisms and mammalian cell lines (Table 4). Among the seven isolated compounds, three exhibited antimicrobial activity in our assays.

In the present study, papulacandin G (**1**) exhibited activity against the fungal strains *S. pombe* and *C. albicans*, with MIC values of 8.3 and 4.1 μg/mL, respectively. Additionally, it displayed weak antibacterial activity against the Gram-positive bacteria *B. subtilis* (MIC = 33.3 µg/mL) and *S. aureus* (MIC = 66.6 µg/mL). Papulacandins A–E were originally isolated from cultures of the sordariomycete *Apiospora sphaerosperma* (Van der Kaaden et al. 2012). Papulacandins constitute a family of natural products characterized by a benzannulated spiroketal moiety, a structural motif frequently found in bioactive secondary metabolites. Members of this compound family display potent antifungal activity by targeting (1,3)-*β*-D-glucan synthase, thereby disrupting cell wall formation through reduced incorporation of the key polysaccharide *β*-glucan (Sperry et al. 2010).

In contrast, papulacandin D (**3**) did not exhibit biological activity against the tested strains. In the literature, papulacandins A–D have been evaluated against *C. albicans*, exhibiting MIC values of 0.2, 0.1, 0.4, and 1–2 µg/mL, respectively (Breta-Oliveiras et al. 2020). Notably, papulacandin D has been described as the least potent derivative among the papulacandins.

Mer-WF3010 (**2**), another papulacandin, exhibited activity against *S. pombe* (MIC = 2.1 µg/mL), *W. anomalus* (MIC = 33.3 μg/mL), *B. subtilis* (MIC = 8.3 μg/mL), and *S. aureus* (MIC = 16.6 μg/mL). It also showed strong antifungal activity against *C. albicans* (MIC = 0.2 μg/mL), which is higher than the previously reported activity of the same compound isolated from *G. cyclaminis* (MIC = 0.63 μg/mL) (Kaneto et al. 1993). Since Mer-WF3010 has been reported exclusively from two species belonging to this newly described genus (*G. cyclaminis* and *G. chlamydospora*), and not from any other taxa, it represents a potential chemotaxonomic marker. Chemotaxonomy can contribute to a more natural classification of sordarialean fungi. For example, the new genus *Pseudorhypophila* was established by Harms et al. (2021a), supported by the production of zopfinol in combination with phylogenetic evidence.

The structures of papulacandins vary primarily in the two partially unsaturated acyl chains attached to the sugar moieties (Van Der Kaaden et al. 2012). Comparison of activity against *C. albicans* among different papulacandin derivatives, particularly between papulacandin G (**1**) and Mer-WF3010 (**2**), supports the notion that minor modifications in these fatty acid tails result in only small changes in biological activity. However, more drastic alterations of these chains, such as full saturation, de‑acylation, or the absence of one of the acyl chains, lead to a marked reduction in activity compared with the most potent member of this family, papulacandin B. This trend is supported by the loss of activity observed for papulacandin D (**3**), which differs from other papulacandins by lacking one of the long fatty acid residues (Bretas et al. 2020).

Overall, these antifungal metabolites stand out as promising leads for the development of antifungal drugs. This is particularly important given the global rise in fungal infections and antifungal resistance. This growing concern arises not only from inappropriate antifungal use in human health and agriculture but also from the limited number of available therapeutic options and the general lack of attention given to fungal diseases (Vitiello et al. 2023). Furthermore, a considerable proportion of pathogens listed in the WHO Fungal Priority Pathogens List exhibit intrinsic resistance to current systemic antifungal agents, while others rapidly acquire resistance following exposure in environmental or clinical settings (Hui et al. 2024).

Penazaphilone M (**4**) did not exhibit antimicrobial activity but showed mild cytotoxicity against the mouse fibroblast L929 cell line (IC_50_ = 26 µg/mL). Penazaphilone M belongs to the azaphilone class of fungal polyketide pigments, which are characterized by a highly oxygenated pyranoquinone bicyclic core. This class encompasses a wide range of biologically active compounds with antimicrobial, antiviral, cytotoxic, anticancer, and anti-inflammatory properties (Gao et al. 2013; Kuhnert et al. 2014; Surup et al. 2013). Penazaphilones were previously isolated from *Penicillium sclerotiorum*, among which penazaphilones A, E–H and L exhibited significant anti-inflammatory activity by suppressing nitric oxide production in lipopolysaccharide-stimulated RAW 264.7 cells (Tang et al. 2019; Zhang et al. 2023). The reported bioactivity profiles of related analogs suggest that further evaluation of the anti-inflammatory potential of penazaphilone M (**4**) is warranted.

The final group of isolated compounds comprised 10-membered lactones: cremenolide (**6**), aspinolide B (**7**), and the previously undescribed opened-ring derivative cremenoic acid (**5**). Cremenolide was first isolated from *Trichoderma cremeum* and reported to inhibit the mycelial growth of *Fusarium oxysporum*, *Botrytis cinerea*, and *Rhizoctonia solani* (Vinale et al. 2016). Aspinolide B was initially identified during screening of *Aspergillus ochraceus* (Fuchser and Zeek 1997) and later from *Trichoderma arundinaceum*, where it was shown to possess plant growth-regulating properties (Malmierca et al. 2015). Although aspinolide B has not been reported to exhibit antimicrobial activity, 10-membered lactones are known to display diverse biological activities, including antibacterial, antimalarial, cytotoxic, antitumor, antifungal, phytotoxic, and antifeedant effects (Chang et al. 2019). In the present study, none of the compounds in this group showed bioactivity against the tested cell lines or microbial pathogens.

## Conclusion

The investigation of the previously undescribed genus *Garciamycella* led to the isolation of seven compounds, three of which are novel derivatives belonging to the papulacandins, penazaphilones, and 10-membered macrolide families. In addition, the production of Mer-WF3010 (**2**) by two species of this genus suggests its potential as a chemotaxonomic marker and supports the introduction of the new genus based on morphological and molecular data.

Beyond the taxonomic implications, the newly discovered metabolites and their structural diversity emphasize the untapped biosynthetic and pharmaceutical potential of the order *Sordariales* and underscore the importance of exploring neglected or taxonomically novel taxa. Moreover, given the potent antifungal activities observed, these compounds represent promising leads for antifungal drug discovery at a time of increasing resistance to existing therapies. Further investigations across related *Sordariales* may uncover additional chemical diversity.

## Supplementary Material

XML Treatment for Garciamycella

XML Treatment for Garciamycella chlamydospora

XML Treatment for Garciamycella cyclaminis

XML Treatment for Garciamycella fici

## Additional information

### Use of AI

No use of AI was reported.

### Adherence to national and international regulations

All the fungal strains used in this study have been legally obtained, respecting the Convention on Biological Diversity (Rio Convention).

### Author ORCIDs

Manuela Agudelo-Restrepo https://orcid.org/0009-0003-5842-202X

Margarita Hernández-Restrepo https://orcid.org/0000-0001-5157-3392

Mahmoud A. A. Ibrahim https://orcid.org/0000-0003-4819-2040

Esteban Charria-Girón https://orcid.org/0000-0002-3823-1416

Sherif S. Ebada https://orcid.org/0000-0002-2753-0031

Yasmina Marin-Felix https://orcid.org/0000-0001-8045-4798

### Data availability

The DNA sequences are deposited in GenBank (https://www.ncbi.nlm.nih.gov/genbank/).

## Supplementary materials

Supplementary material 1 Data related to structure elucidation, and phylogenetic analysis https://doi.org/10.3897/imafungus.17.177411.suppl1 Data type docx https://binary.pensoft.net/file/1511913This dataset is made available under the Open Database License (http://opendatacommons.org/licenses/odbl/1.0/). The Open Database License (ODbL) is a license agreement intended to allow users to freely share, modify, and use this Dataset while maintaining this same freedom for others, provided that the original source and author(s) are credited. Manuela Agudelo-Restrepo, Margarita Hernández-Restrepo, Mahmoud A. A. Ibrahim, Esteban Charria-Girón, Sherif S. Ebada, Yasmina Marin-Felix

## References

[B1] Alfaro ME, Zoller S, Lutzoni F (2003) Bayes or bootstrap? A simulation study comparing the performance of Bayesian Markov chainMonte Carlo sampling and bootstrapping in assessing phylogenetic confidence. Molecular Biology and Evolution 20(2): 255–266. 10.1093/molbev/msg028 12598693

[B2] Bell A, Mahoney DP, Debuchy R (2016) *Podospora bullata*, a new homothallic ascomycete from kangaroo dung in Australia. Ascomycete.org 8: 111–118. 10.25664/art-0180

[B3] Bills GF, Gloer JB (2016) Biologically active secondary metabolites from the fungi. Microbiology Spectrum 4(6). 10.1128/microbiolspec.FUNK-0009-2016 27809954

[B4] Bretas ACO, Souza TBD, Borelli B et al. (2020) Synthesis of novel papulacandin D analogs and evaluation of their antifungal potential. Brazilian Journal of Pharmaceutical Sciences, 56. 10.1590/s2175-97902019000417652

[B5] Bruhn T, Schaumloffel A, Hemberger Y et al. (2013) SpecDis: Quantifying the comparison of calculated and experimental electronic circular dichroism spectra. Chirality 25: 243–249. 10.1002/chir.22138 23532998

[B6] Cai L, Jeewon R, Hyde KD (2005) Phylogenetic evaluation and taxonomic revision of *Schizothecium* based on ribosomal DNA and protein coding genes. Fungal Diversity 19: 1-21.

[B7] Cai L, Jeewon R, Hyde KD (2006) Phylogenetic investigations of *Sordariaceae* based on multiple gene sequences and morphology. Mycological Research 110(2): 137–150. 10.1016/j.mycres.2005.09.014 16378718

[B8] Chang C, Geng J, Du Y et al. (2019) Divergent total synthesis of aspinolides B, E and J. Tetrahedron 75(29): 3933–3938. 10.1016/j.tet.2019.06.015

[B9] Charria-Girón E, Stchigel AM, Čmoková A et al. (2023) *Amesia hispanica* sp. nov., Producer of the antifungal class of antibiotics dactylfungins. Journal of Fungi 9(4): 463. 10.3390/jof9040463 PMC10141101 37108917

[B10] Charria-Girón E, Surup F, Marin-Felix Y (2022) Diversity of biologically active secondary metabolites in the ascomycete order *Sordariales*. Mycological Progress 21(4): 43. 10.1007/s11557-022-01775-3

[B11] Chiba H, Kaneto R, Agematu H et al. (1993) Mer-WF3010, a new member of the papulacandin family. II. Structure determination. Journal of Antibiotics 46(2): 356–358. 10.7164/antibiotics.46.356 8468253

[B12] Crous PW, Wingfield MJ, Burgess TI et al. (2017) Fungal Planet description sheets: 625–715. Persoonia 39: 270–274. 10.3767/persoonia.2017.39.11 PMC5832955 29503478

[B13] Crous PW, Luangsa-ard JJ, Wingfield MJ et al. (2018) Fungal Planet description sheets: 785–867. Persoonia 41: 238–417. 10.3767/persoonia.2018.41.12 PMC6344811 30728607

[B14] Dai DQ, Phookamsak R, Wijayawardene NN et al. (2017) Bambusicolous fungi. Fungal Diversity 82: 1–105. 10.1007/s13225-016-0367-8

[B15] Fernández FA, Lutzoni FM, Huhndorf SM (1999) Teleomorph-anamorph connections: the new pyrenomycetous genus *Carpoligna* and its *Pleurothecium* anamorph. Mycologia 91(2):251– 262. 10.1080/00275514.1999.12061015

[B16] Fernández FA, Miller AN, Huhndorf SM et al. (2006) Systematics of the genus *Chaetosphaeria* and its allied genera: morphological and phylogenetic diversity in north temperate and neotropical taxa. Mycologia 98: 121–130. 10.1080/15572536.2006.11832718 16800310

[B17] Frisch MJ, Trucks GW, Schlegel HB et al. (2009) Gaussian 09, Revision E01; Gaussian Inc.: Wallingford CT, USA.

[B18] Fuchser J, Zeeck A (1997) Aspinolides and aspinonene/aspyrone co-metabolites, new pentaketides produced by *Aspergillus ochraceus*. Liebigs Ann. Recueil 1997(1): 87–95. 10.1002/jlac.199719970114

[B19] Fuckel L (1870) Symbolae mycologicae. Beiträge zur Kenntniss der Rheinischen Pilze. Jahrbücher des Nassauischen Vereins für Naturkunde 23-24: 1–459. 10.5962/bhl.title.47117

[B20] Gao JM, Yang SX, Qin JC (2013) Azaphilones: Chemistry and Biology. Chemical Reviews 113(7): 4755–4811. 10.1021/cr300402y 23581812

[B21] Giraldo A, Hernández-Restrepo M, Crous PW (2019) New plectosphaerellaceous species from Dutch garden soil. Mycological Progress 18(9): 1135–1154. 10.1007/s11557-019-01511-4

[B22] Gu BB, Wu Y, Tang J et al. (2018) Azaphilone and isocoumarin derivatives from the sponge-derived fungus *Eupenicillium* sp. 6A-9. Tetrahedron Letters 59: 3345–3348. 10.1016/j.tetlet.2018.06.057

[B23] Guerra-Mateo D, Cano-Lira JF, Fernández-Bravo A et al. (2024) Sunken riches: ascomycete diversity in the Western Mediterranean coast through direct plating and flocculation, and description of four new taxa. Journal of Fungi 10(4): 281. 10.3390/jof10040281 PMC11051201 38667952

[B24] Górz A, Boroń P (2018) *Episternus onthophagi*: a new monotypic genus of epizoic fungus found on *Onthophagus* beetles (*Scarabaeoidea*). Phytotaxa 376(1): 43–59. 10.11646/phytotaxa.376.1.5

[B25] Greif MD, Stchigel AM, Miller AN et al. (2009) A re-evaluation of genus *Chaetomidium* based on molecular and morphological characters. Mycologia 101(4): 554–564. 10.3852/08-200 19623937

[B26] Groenewald M, Lombard L, De Vries M et al. (2018) Diversity of yeast species from Dutch garden soil and the description of six novel Ascomycetes. FEMS Yeast Research 18(7). 10.1093/femsyr/foy076 30016423

[B27] Grünig CR, Queloz V, Sieber TN (2011) Structure of diversity in dark septate endophytes: From species to genes. Endophytes of forest trees: Biology and applications. Dordrecht: Springer Netherlands, 3-30. 10.1007/978-94-007-1599-8_1

[B28] Harms K, Milic A, Stchigel AM et al. (2021a) Three new derivatives of zopfinol from *Pseudorhypophila mangenotii* gen. et comb. nov. Journal of Fungi 7(3): 181. 10.3390/jof7030181 PMC8000789 33802411

[B29] Harms K, Surup F, Stadler M et al. (2021b) Morinagadepsin, a depsipeptide from the fungus *Morinagamyces vermicularis* gen. et comb. nov. Microorganisms 9(6): 1191. 10.3390/microorganisms9061191 PMC8230337 34073017

[B30] Harms K, Charria-Girón E, Stchigel AM et al. (2024) Reaping the chemical diversity of *Morinagamyces vermicularis* using feature-based molecular networking. Journal of Natural Products 87(9): 2335–2342. 10.1021/acs.jnatprod.4c00654 PMC11443486 39279157

[B31] Hemtasin C, Kanokmedhakul S, Moosophon P et al. (2016) Bioactive azaphilones from the fungus *Penicillium multicolor* CM01. Phytochemistry Letters 16: 56–60. 10.1016/j.phytol.2016.03.004

[B32] Huhndorf SM, Miller AN, Fernández FA (2004) Molecular systematics of the *Sordariales*: the order and the family *Lasiosphaeriaceae* redefined. Mycologia 96: 368–387. 10.1080/15572536.2005.11832982 21148859

[B33] Hui ST, Gifford H, Rhodes J (2024) Emerging antifungal resistance in fungal pathogens. Current Clinical Microbiology Reports 11(2): 43–50. 10.1007/s40588-024-00219-8 PMC11076205 38725545

[B34] Hyde KD, Baldrian P, Chen Y et al. (2024) Current trends, limitations and future research in the fungi? Fungal Diversity 125(1): 1–71. 10.1007/s13225-023-00532-5

[B35] Ibrahim SRM, Mohamed SGA, Sindi IA et al. (2021) Biologically active secondary metabolites and biotechnological applications of species of the family Chaetomiaceae (Sordariales): An updated review from 2016 to 2021. Mycological Progress 20(5): 595–639. 10.1007/s11557-021-01704-w

[B36] Jansen N, Ohlendorf B, Erhard A et al. (2013) Helicusin E, isochromophilone X and isochromophilone XI: New chloroazaphilones produced by the fungus *Bartalinia robillardoides* strain LF550. Marine Drugs 11(3): 800–816. 10.3390/md11030800 PMC3705371 23481677

[B37] Kaneto R, Chiba H, Agematu H et al. (1993) Mer-WF3010, a new member of the papulacandin family. I. Fermentation, isolation and characterization. Journal of Antibiotics 46(2): 247–250. 10.7164/antibiotics.46.247 8468238

[B38] Kanokmedhakul S, Kanokmedhakul K, Nasomjai P et al. (2006) Antifungal azaphilones from the fungus *Chaetomium cupreum* CC3003. Journal of Natural Products 69: 891–895. 10.1021/np060051v 16792406

[B39] Katoh K, Standley DM (2013) MAFFT Multiple Sequence Alignment Software Version 7: improvements in performance and usability. Molecular Biology and Evolution 30: 772–780. 10.1093/molbev/mst010 PMC3603318 23329690

[B40] Kearse M, Moir R, Wilson A et al. (2012) Geneious Basic: an integrated and extendable desktop software platform for the organization and analysis of sequence data. Bioinformatics 28: 1647–1649. 10.1093/bioinformatics/bts199 PMC3371832 22543367

[B41] Komori T, Itoh Y (1985) Chaetiacandin, a novel papulacandin. II. Structure determination. Journal of Antibiotics 38(4): 544–546. 10.7164/antibiotics.38.544 3839234

[B42] Komori T, Yamashita M, Tsurumi Y et al. (1985) Chaetiacandin, a novel papulacandin. I. Fermentation, isolation and characterization. Journal of Antibiotics 38(4): 455–459. 10.7164/antibiotics.38.455 3839230

[B43] Kruys Å, Huhndorf SM, Miller AN (2015) Coprophilous contributions to the phylogeny of *Lasiosphaeriaceae* and allied taxa within *Sordariales* (*Ascomycota*, Fungi). Fungal Diversity 70(1): 101–113. 10.1007/s13225-014-0296-3

[B44] Kumar S, Stecher G, Li M et al. (2018) MEGA X: Molecular Evolutionary Genetics Analysis across Computing Platforms. Molecular Biology and Evolution 35(6): 1547–1549. 10.1093/molbev/msy096 PMC5967553 29722887

[B45] Kuhnert E, Heitkämper S, Fournier J et al. (2014) Hypoxyvermelhotins A–C, new pigments from *Hypoxylon lechatii* sp. nov. Fungal Biology 118(2): 242–252. 10.1016/j.funbio.2013.12.003 24528645

[B46] Liu YJ, Whelen S, Hall BD (1999) Phylogenetic relationships among ascomycetes: Evidence from an RNA Polymerase II Subunit. Molecular Biology and Evolution 16: 1799–1808. 10.1093/oxfordjournals.molbev.a026092 10605121

[B47] Luo X, Lin X, Tao H et al. (2018) Isochromophilones A–F, cytotoxic chloroazaphilones from the marine mangrove endophytic fungus *Diaporthe* sp. SCSIO 41011. Journal of Natural Products 81(4): 934–941. 10.1021/acs.jnatprod.7b01053 29517908

[B48] Madrid H, Cano J, Stchigel A et al. (2010) *Ramophialophora humicola* and *Fibulochlamys chilensis*, two new microfungi from soil. Mycologia 102: 605–612. 10.3852/09-128 20524593

[B49] Madrid H, Cano J, Gené J et al. (2011) Two new species of *Cladorrhinum*. Mycologia 103: 795–805. 10.3852/10-150 21307165

[B50] Malmierca MG, Barua J, McCormick SP et al. (2015) Novel aspinolide production by *Trichoderma arundinaceum* with a potential role in *Botrytis cinerea* antagonistic activity and plant defence priming. Environmental Microbiology 17(4): 1103–1118. 10.1111/1462-2920.12514 24889745

[B51] Matsuzaki K, Ikeda H, Masuma R et al. (1995) Isochromophilones I and II, novel inhibitors against gp120-CD4 binding produced by *Penicillium multicolor* FO-2338. Journal of Antibiotics 48(7): 703–707. 10.7164/antibiotics.48.703 7649871

[B52] Marin-Felix Y, Miller AN, Cano-Lira JF et al. (2020) Re-evaluation of the order *Sordariales*: delimitation of *Lasiosphaeriaceae* s. str., and introduction of the new families *Diplogelasinosporaceae*, *Naviculisporaceae*, and *Schizotheciaceae*. Microorganisms 8(9):1430. 10.3390/microorganisms8091430 PMC7565071 32957559

[B53] McMullin DR, Sumarah MW, Blackwell BA et al. (2013) New azaphilones from *Chaetomium globosum* isolated from the built environment. Tetrahedron Letters 54: 568–572. 10.1016/j.tetlet.2012.11.084

[B54] Michael AP, Grace EJ, Kotiw M et al. (2003) Isochromophilone IX, a novel GABA-containing metabolite isolated from a cultured fungus, *Penicillium* sp. Australian journal of chemistry 56(1): 13–16. 10.1002/chin.200331240

[B55] Miertuš S, Scrocco E, Tomasi J (1981) Electrostatic interaction of a solute with a continuum. A direct utilizaion of ab initio molecular potentials for the prevision of solvent effects. Chemical Physics 55(1): 117–129. 10.1016/0301-0104(81)85090-2

[B56] Miller AN, Huhndorf SM (2004a) A natural classification of *Lasiosphaeria* based on nuclear LSU rDNA sequences. Mycological Research 108: 26–34. 10.1017/S0953756203008864 15035502

[B57] Miller AN, Huhndorf SM (2004b) Using phylogenetic species recognition to delimit species boundaries within *Lasiosphaeria*. Mycologia 96(5): 1106–1127. 10.1080/15572536.2005.11832909 21148930

[B58] Miller AN, Huhndorf S (2005) Multi-gene phylogenies indicate ascomal wall morphology is a better predictor of phylogenetic relationships than ascospore morphology in the *Sordariales* (*Ascomycota*, Fungi). Molecular Phylogenetics and Evolution 35(1): 60–75. 10.1016/j.ympev.2005.01.007 15737582

[B59] Moubasher AH, Ismail MA, Al-Bedak OA et al. (2019) *Ramophialophora chlamydospora*, a new species from an alkaline lake of Wadi-El-Natron, Egypt. Asian Journal of Mycology 2(1): 110–117. 10.5943/ajom/2/1/5

[B60] Pilli RA, Victor MM, De Meijere A (2000) First total synthesis of aspinolide B, a new pentaketide produced by *Aspergillus ochraceus*. The Journal of Organic Chemistry 65(19): 5910–5916. 10.1021/jo000327i 10987921

[B61] Poynton EF, van Santen JA, Pin M et al. (2024) The Natural Products Atlas 3.0: Extend­ing the database of microbially-derived natural products. Nucleic Acids Researchg 50(D1): D1317–D1323. 10.1093/nar/gkab941 PMC8728154 34718710

[B62] Pracht P, Grimme S, Bannwarth C et al. (2024) CREST-A program for the exploration of low-energy molecular chemical space. The Journal of Chemical Physics 160(11): 114110. 10.1063/5.0197592 38511658

[B63] Raja HA, Schoch CL, Hustad VP et al. (2011) Testing the phylogenetic utility of MCM7 in the Ascomycota. MycoKeys 1: 63–94. 10.3897/mycokeys.1.1966

[B64] Réblová M (2008) *Bellojisia*, a new sordariaceous genus for *Jobellisia rhynchostoma* and a description of *Jobellisiaceae* fam. nov. Mycologia 100(6): 893–901. 10.3852/08-068 19202843

[B65] Ronquist F, Teslenko M, van der Mark P et al. (2012) MrBayes 3.2: Efficient Bayesian phylogenetic inference and model choice across a large model space. Systematic Biology 61(3): 539–542. 10.1093/sysbio/sys029 PMC3329765 22357727

[B66] Royal Horticultural Society (1996) RHS Colour Chart. Royal Horticultural Society, London, UK.

[B67] Schrey H, Lambert C, Stadler M (2025) Fungi: Pioneers of chemical creativity – Techniques and strategies to uncover fungal chemistry. IMA Fungus 16: e142462. 10.3897/imafungus.16.142462 PMC11909596 40093757

[B68] Shao L, Marin-Felix Y, Surup F et al. (2020) Seven new cytotoxic and antimicrobial xanthoquinodins from *Jugulospora vestita*. Journal of Fungi 6(4): 188. 10.3390/jof6040188 PMC7712541 32992954

[B69] Shao Y, Molestak E, Su W et al. (2022) Sordarin—An anti‐fungal antibiotic with a unique modus operandi. British Journal of Pharmacology 179(6): 1125–1145. 10.1111/bph.15724 34767248

[B70] Sperry J, Wilson ZE, Rathwell DCK et al. (2010) Isolation, biological activity and synthesis of benzannulated spiroketal natural products. Natural Product Reports 27(8): 1117. 10.1039/b911514p 20648380

[B71] Stamatakis A (2014) RAxML version 8: A tool for phylogenetic analysis and post-analysis of large phylogenies. Bioinformatics (30): 1312–1313. 10.3897/mycokeys.1.1966 PMC3998144 24451623

[B72] Stchigel AM, Cano J, Miller AN et al. (2006) *Corylomyces*: a new genus of *Sordariales* from plant debris in France. Mycological Research 110(11): 1361–1368. 10.1016/j.mycres.2006.08.003 17071066

[B73] Surup F, Mohr KI, Jansen R et al. (2013) Cohaerins G–K, azaphilone pigments from *Annulohypoxylon cohaerens* and absolute stereochemistry of cohaerins C–K. Phytochemistry 95: 252–258. 10.1016/j.phytochem.2013.07.027 23969107

[B74] Tang J, Zhou Z, Yang T et al. (2019) Azaphilone alkaloids with anti-inflammatory activity from fungus *Penicillium sclerotiorum* cib-411. Journal of Agricultural and Food Chemistry 67(8): 2175–2182. 10.1021/acs.jafc.8b05628 30702881

[B75] Tennakoon DS, Kuo CH, Maharachchikumbura SS et al. (2021) Taxonomic and phylogenetic contributions to *Celtis formosana*, *Ficus ampelas*, *F. septica*, *Macaranga tanarius* and *Morus australis* leaf litter inhabiting microfungi. Fungal Diversity 108: 1–215. 10.1007/s13225-021-00474-w

[B76] Thiyagaraja V, Hyde KD, Piepenbring M et al. (2025) Orders of Ascomycota. Mycosphere 16(1): 536–1411. 10.5943/mycosphere/16/1/8

[B77] Tomasi J, Persico M (1994) Molecular interactions in solution: An overview of methods based on continuous distributions of the solvent. Chemical Reviews 94(7): 2027–2094. 10.1021/cr00031a013

[B78] Traxler P, Gruner J, Auden JAL (1977) Papulacandins, a new family of antibiotics with antifungal activity. I. Fermentation, isolation, chemical and biological characterization of papulacandins A, B, C, D and E. Journal of Antibiotics 30(4): 289–296. 10.7164/antibiotics.30.289 324958

[B79] Traxler P, Fritz H, Fuhrer H et al. (1980) Papulacandins, a new family of antibiotics with antifungal activity. Structures of papulacandins A, B, C and D. Journal of Antibiotics 33(9): 967–978. 10.7164/antibiotics.33.967 7440418

[B80] Vaidya G, Lohman DJ, Meier R (2011) SequenceMatrix: concatenation software for the fast assembly of multi-gene datasets with character set and codon information. Cladistics 27: 171–180. 10.1111/j.1096-0031.2010.00329.x 34875773

[B81] Vanmiddlesworth F, Omstead MN, Schmatz D et al. (1991a) L-687,781, a new member of the papulacandin family of *β*-1,3-D-glucan synthesis inhibitors. I. Fermentation, isolation and biological activity. Journal of Antibiotics 44(1): 45–51. 10.7164/antibiotics.44.45 2001985

[B82] VanMiddlesworth F, Dufresne C, Smith J et al. (1991b) Structure elucidation of L-687,781, a new *β*-1,3-D-glucan synthesis inhibitor. Tetrahedron 47(36): 7563–7568. 10.1016/S0040-4020(01)88280-6

[B83] Van Der Kaaden M, Breukink E, Pieters RJ (2012) Synthesis and antifungal properties of papulacandin derivatives. Beilstein Journal of Organic Chemistry 8: 732–737. 10.3762/bjoc.8.82 PMC3388860 23015820

[B84] Vilgalys R, Hester M (1990) Rapid genetic identification and mapping of enzymatically amplified ribosomal DNA from several *Cryptococcus* species. Journal of Bacteriology 172(8): 4238–4246. 10.1128/jb.172.8.4238-4246.1990 PMC213247 2376561

[B85] Vinale F, Strakowska J, Mazzei P et al. (2016) Cremenolide, a new antifungal, 10-member lactone from *Trichoderma cremeum* with plant growth promotion activity. Natural Product Research 30(22): 2575–2581. 10.1080/14786419.2015.1131985 26728227

[B86] Vitiello A, Ferrara F, Boccellino M et al. (2023) Antifungal drug resistance: An emergent health threat. Biomedicines 11(4): 1063. 10.3390/biomedicines11041063 PMC10135621 37189681

[B87] Vu D, Groenewald M, de Vries M et al. (2019) Large-scale generation and analysis of filamentous fungal DNA barcodes boosts coverage for kingdom Fungi and reveals thresholds for fungal species and higher taxon delimitation. Studies in Mycology 92(1): 135–154. 10.1016/j.simyco.2018.05.001 PMC6020082 29955203

[B88] Wang XW, Bai FY, Bensch K et al. (2019) Phylogenetic re-evaluation of *Thielavia* with the introduction of a new family *Podosporaceae*. Studies in Mycology 93(1): 155–252. 10.1016/j.simyco.2019.08.002 PMC6816082 31824584

[B89] Wang XW, Houbraken J, Groenewald JZ et al. (2016a) Diversity and taxonomy of *Chaetomium* and chaetomium-like fungi from indoor environments. Studies in Mycology 84(1): 145–224. 10.1016/j.simyco.2016.11.005 PMC5226397 28082757

[B90] Wang XW, Lombard L, Groenewald JZ et al. (2016b) Phylogenetic reassessment of the *Chaetomium globosum* species complex. Persoonia 36(1): 83–133. 10.3767/003158516x689657 PMC4988377 27616789

[B91] Wang HC, Ke TY, Ko YC et al. (2021) Anti-Inflammatory azaphilones from the edible alga-derived fungus *Penicillium sclerotiorum*. Marine Drugs 19(10): 529. 10.3390/md19100529 PMC8537458 34677428

[B92] White TJ, Bruns TD, Lee S et al. (1990) Amplification and direct sequencing of fungal ribosomal genes for phylogenetics. In PCR Protocols: A Guide to Methods and Applications; Gelfand, M., Sninsky, J.I., White, T.J., Eds.; Academic Press: New York, NY, USA, 1990, 315–322. 10.1016/B978-0-12-372180-8.50042-1

[B93] Zhang ZF, Liu F, Zhou X et al. (2017) Culturable mycobiota from Karst caves in China, with descriptions of 20 new species. Persoonia-Molecular Phylogeny and Evolution of Fungi 39(1): 1–31. 10.3767/persoonia.2017.39.01 PMC5832949 29503468

[B94] Zhang ZF, Zhao P, Cai L (2018) Origin of cave fungi. Frontiers in Microbiology 9: 1407. 10.3389/fmicb.2018.01407 PMC6036247 30013527

[B95] Zhang X, Hu Y, Yang T et al. (2023) Penazaphilones J–L, three new hydrophilic azaphilone pigments from *Penicillium sclerotiorum* cib-411 and their anti-inflammatory activity. Molecules 28(7): 3146. 10.3390/molecules28073146 PMC10095951 37049911

