# Supplementary material for: New sordarialean lineage Garciamycella chlamydospora (Sordariales, Schizotheciaceae) produces rare antifungal papulacandins
Source: IMA Fungus. 2026 Jan 14;17:e177411. doi: 10.3897/imafungus.17.177411 (PMC12824588; doi:10.3897/imafungus.17.177411)
Supplement: Supplementary material 1 — Data related to structure elucidation, and phylogenetic analysis [file imafungus-17-e177411-s001.docx]

Supplementary Materials for:

New sordarialean lineage *Garciamycella chlamydospora* produces rare antifungal papulacandins

Manuela Agudelo-Restrepo^1,2^, Margarita Hernández-Restrepo^3^, Mahmoud A. A. Ibrahim,^4,5,6^ Esteban Charria-Girón^1,2^, Sherif S. Ebada^1,7,^*, Yasmina Marin-Felix^1,2,^*

^1^ Department of Microbial Drugs, Helmholtz Centre for Infection Research, 38124 Braunschweig, Germany;

^2^ Institute of Microbiology, Technische Universität Braunschweig, 38106 Braunschweig, Germany

^3^ Westerdijk Fungal Biodiversity Institute, Uppsalalaan 8, 3584CT, Utrecht, the Netherlands

^4^ Computational Chemistry Laboratory, Chemistry Department, Faculty of Science, Minia University, Minia 61519, Egypt

^5^ Department of Engineering, College of Engineering and Technology, University of Technology and Applied Sciences, Nizwa 611, Sultanate of Oman

^6^ School of Health Sciences, University of KwaZulu-Natal, Westville Campus, Durban 4000, South Africa

^7^ Department of Pharmacognosy, Faculty of Pharmacy, Ain Shams University, 11566 Cairo, Egypt

*Correspondence: [sherif.elsayed@helmholtz-hzi.de](mailto:sherif.elsayed@helmholtz-hzi.de); [sherif_elsayed@pharma.asu.edu.eg](mailto:sherif_elsayed@pharma.asu.edu.eg) (S.S.E.); [yasmina.marinfelix@helmholtz-hzi.de](mailto:yasmina.marinfelix@helmholtz-hzi.de) (Y.M.-F.); Tel.: +49-531-6181-424

Contents of Supporting Information

| # | Contents | Page |
| --- | --- | --- |
| 1 | Figure S1. LR-ESI-MS of **1**. | S4 |
| 2 | Figure S2. HR-ESI-MS of **1**. | S5 |
| 3 | Figure S3. ^1^H NMR spectrum of **1** in methanol-*d*_4_ at 700 MHz. | S6 |
| 4 | Figure S4. ^1^H–^1^H COSY spectrum of **1** in methanol-*d*_4_ at 700 MHz. | S7 |
| 5 | Figure S5. HMBC spectrum of **1** in methanol-*d*_4_ at 700 MHz. | S8 |
| 6 | Figure S6. HSQC spectrum of **1** in methanol-*d*_4_ at 700 MHz. | S9 |
| 7 | Figure S7. ROESY spectrum of **1** in methanol-*d*_4_ at 700 MHz. | S10 |
| 8 | Figure S8. LR-ESI-MS of **2**. | S11 |
| 9 | Figure S9. HR-ESI-MS of **2**. | S12 |
| 10 | Figure S10. ^1^H NMR spectrum of **2** in methanol-*d*_4_ at 600 MHz. | S13 |
| 11 | Figure S11. ^13^C NMR spectrum of **2** in methanol-*d*_4_ at 150 MHz. | S14 |
| 12 | Figure S12. ^1^H–^1^H COSY spectrum of **2** in methanol-*d*_4_ at 600 MHz. | S15 |
| 13 | Figure S13. HMBC spectrum of **2** in methanol-*d*_4_ at 600 MHz. | S16 |
| 14 | Figure S14. HSQC spectrum of **2** in methanol-*d*_4_ at 600 MHz. | S17 |
| 15 | Figure S15. ROESY spectrum of **2** in methanol-*d*_4_ at 600 MHz. | S18 |
| 16 | Figure S16. LR-ESI-MS of **3**. | S19 |
| 17 | Figure S17. HR-ESI-MS of **3**. | S20 |
| 18 | Figure S18. ^1^H NMR spectrum of **3** in methanol-*d*_4_ at 700 MHz. | S21 |
| 19 | Figure S19. DEPTQ spectrum of **3** in methanol-*d*_4_ at 175 MHz. | S22 |
| 20 | Figure S20. ^1^H–^1^H COSY spectrum of **3** in methanol-*d*_4_ at 700 MHz. | S23 |
| 21 | Figure S21. HMBC spectrum of **3** in methanol-*d*_4_ at 700 MHz. | S24 |
| 22 | Figure S22. HSQC spectrum of **3** in methanol-*d*_4_ at 700 MHz. | S25 |
| 23 | Figure S23. ROESY spectrum of **3** in methanol-*d*_4_ at 700 MHz. | S26 |
| 24 | Figure S24. LR-ESI-MS of **4**. | S27 |
| 25 | Figure S25. HR-ESI-MS of **4**. | S28 |
| 26 | Figure S26. ^1^H NMR spectrum of **4** in methanol-*d*_4_ at 500 MHz. | S29 |
| 27 | Figure S27. ^13^C NMR spectrum of **4** in methanol-*d*_4_ at 125 MHz. | S30 |
| 28 | Figure S28. ^1^H–^1^H COSY spectrum of **4** in methanol-*d*_4_ at 500 MHz. | S31 |
| 29 | Figure S29. HMBC spectrum of **4** in methanol-*d*_4_ at 500 MHz. | S32 |
| 30 | Figure S30. HSQC spectrum of **4** in methanol-*d*_4_ at 500 MHz. | S33 |
| 31 | Figure S31. ROESY spectrum of **4** in methanol-*d*_4_ at 500 MHz. | S34 |
| 32 | Figure S32. LR-ESI-MS of **5**. | S35 |
| 33 | Figure S33. HR-ESI-MS of **5**. | S36 |
| 34 | Figure S34. ^1^H NMR spectrum of **5** in methanol-*d*_4_ at 600 MHz. | S37 |
| 35 | Figure S35. DEPTQ spectrum of **5** in methanol-*d*_4_ at 150 MHz. | S38 |
| 36 | Figure S36. ^1^H–^1^H COSY spectrum of **5** in methanol-*d*_4_ at 600 MHz. | S39 |
| 37 | Figure S37. HMBC spectrum of **5** in methanol-*d*_4_ at 600 MHz. | S40 |
| 38 | Figure S38. HSQC spectrum of **5** in methanol-*d*_4_ at 600 MHz. | S41 |
| 39 | Figure S39. ROESY spectrum of **5** in methanol-*d*_4_ at 600 MHz. | S42 |
| 40 | Figure S40. LR-ESI-MS of **6**. | S43 |
| 41 | Figure S41. HR-ESI-MS of **6**. | S44 |
| 42 | Figure S42. ^1^H NMR spectrum of **6** in methanol-*d*_4_ at 500 MHz. | S45 |
| 43 | Figure S43. DEPTQ spectrum of **6** in methanol-*d*_4_ at 125 MHz. | S46 |
| 44 | Figure S44. ^1^H–^1^H COSY spectrum of **6** in methanol-*d*_4_ at 500 MHz. | S47 |
| 45 | Figure S45. HMBC spectrum of **6** in methanol-*d*_4_ at 500 MHz. | S48 |
| 46 | Figure S46. HSQC spectrum of **6** in methanol-*d*_4_ at 500 MHz. | S49 |
| 47 | Figure S47. ROESY spectrum of **6** in methanol-*d*_4_ at 500 MHz. | S50 |
| 48 | Figure S48. LR-ESI-MS of **7**. | S51 |
| 49 | Figure S49. HR-ESI-MS of **7**. | S52 |
| 50 | Figure S50. ^1^H NMR spectrum of **7** in DMSO-*d*_6_ at 600 MHz. | S53 |
| 51 | Figure S51. DEPTQ spectrum of **7** in DMSO-*d*_6_ at 150 MHz. | S54 |
| 52 | Figure S52. ^1^H–^1^H COSY spectrum of **7** in DMSO-*d*_6_ at 600 MHz. | S55 |
| 53 | Figure S53. HMBC spectrum of **7** in DMSO-*d*_6_ at 600 MHz. | S56 |
| 54 | Figure S54. HSQC spectrum of **7** in DMSO-*d*_6_ at 600 MHz. | S57 |
| 55 | Figure S55. ROESY spectrum of **7** in DMSO-*d*_6_ at 600 MHz. | S58 |
| 56 | Figure S56. RAxML phylogram obtained from the internal transcribed spacer region (ITS) sequences | S59 |
| 57 | Figure S57. RAxML phylogram obtained from the nuclear rDNA large subunit (LSU) sequences | S60 |
| 58 | Figure S58. RAxML phylogram obtained from the fragment of ribosomal polymerase II subunit 2 (rpb2) sequences | S61 |
| 59 | Figure S59. RAxML phylogram obtained from the fragment of β-tubulin (tub2) sequences | S62 |
| 60 | Table S1. Alignment used in the phylogenetic study. | S63 |


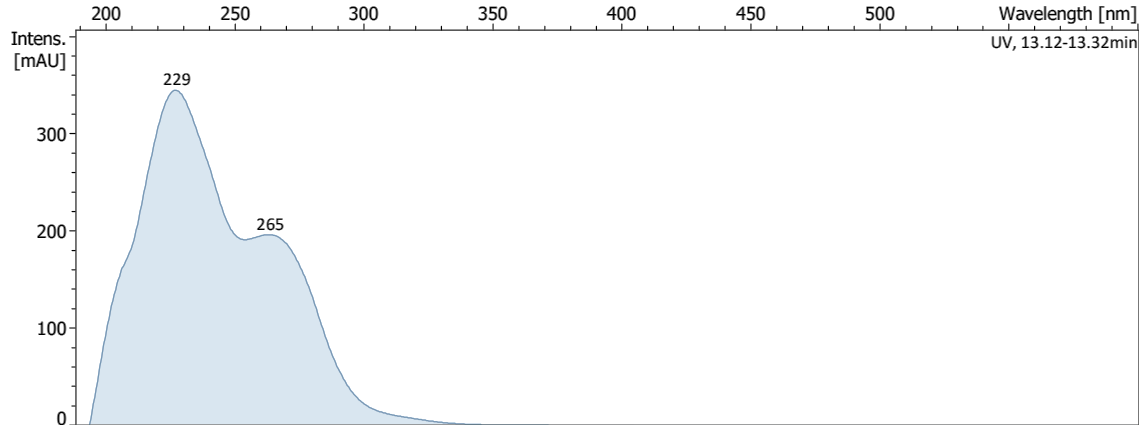

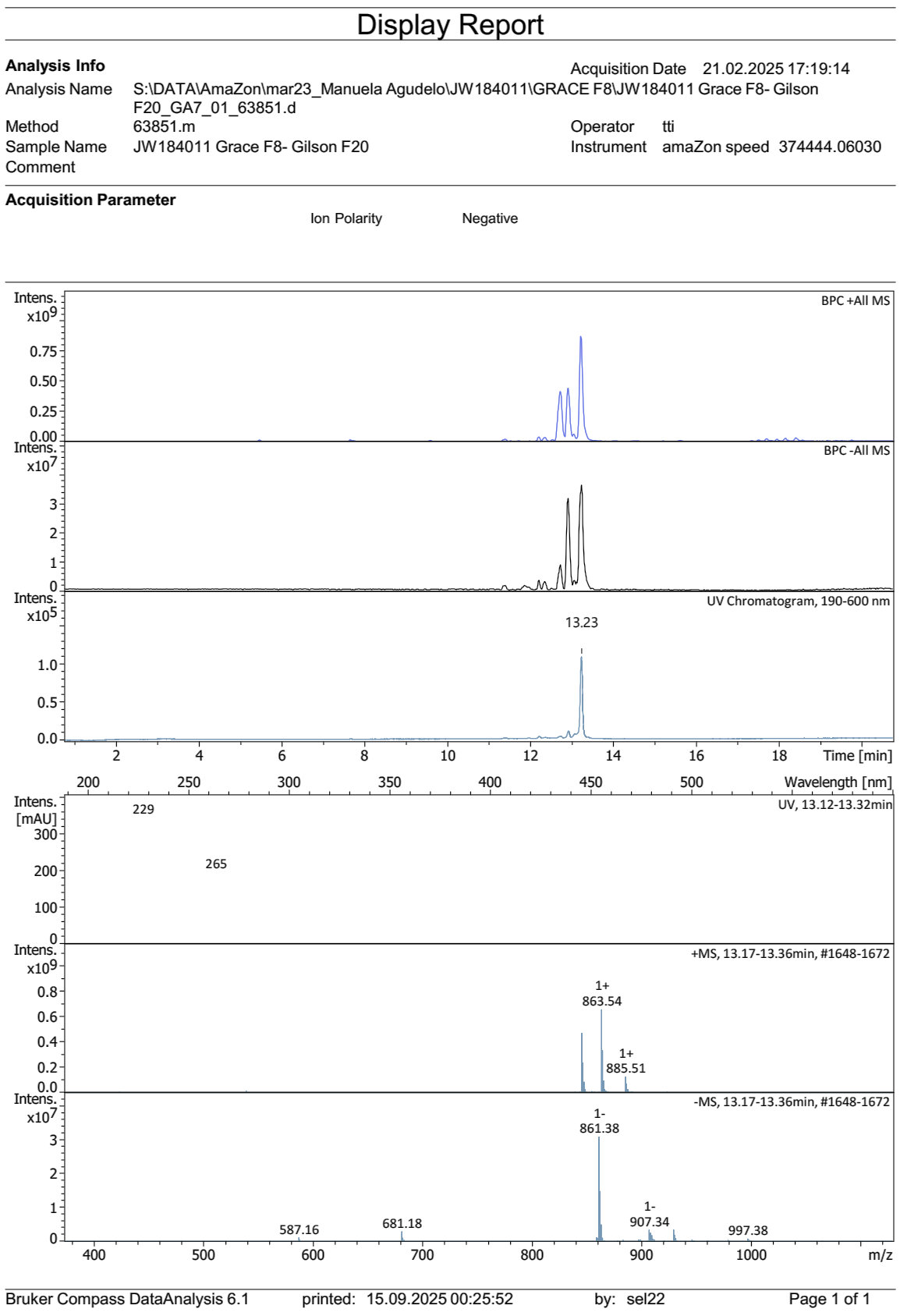


[M-H]^-^

[M+Na]^+^

[M+H]^+^

Figure S1. LR-ESI-MS of **1**.


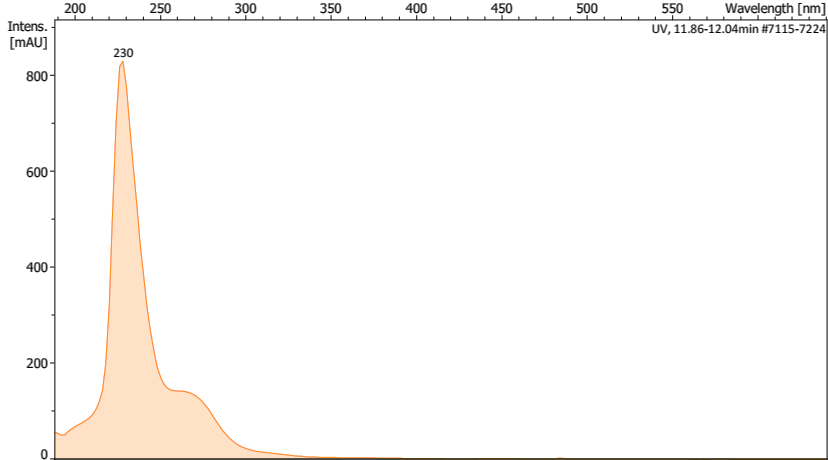

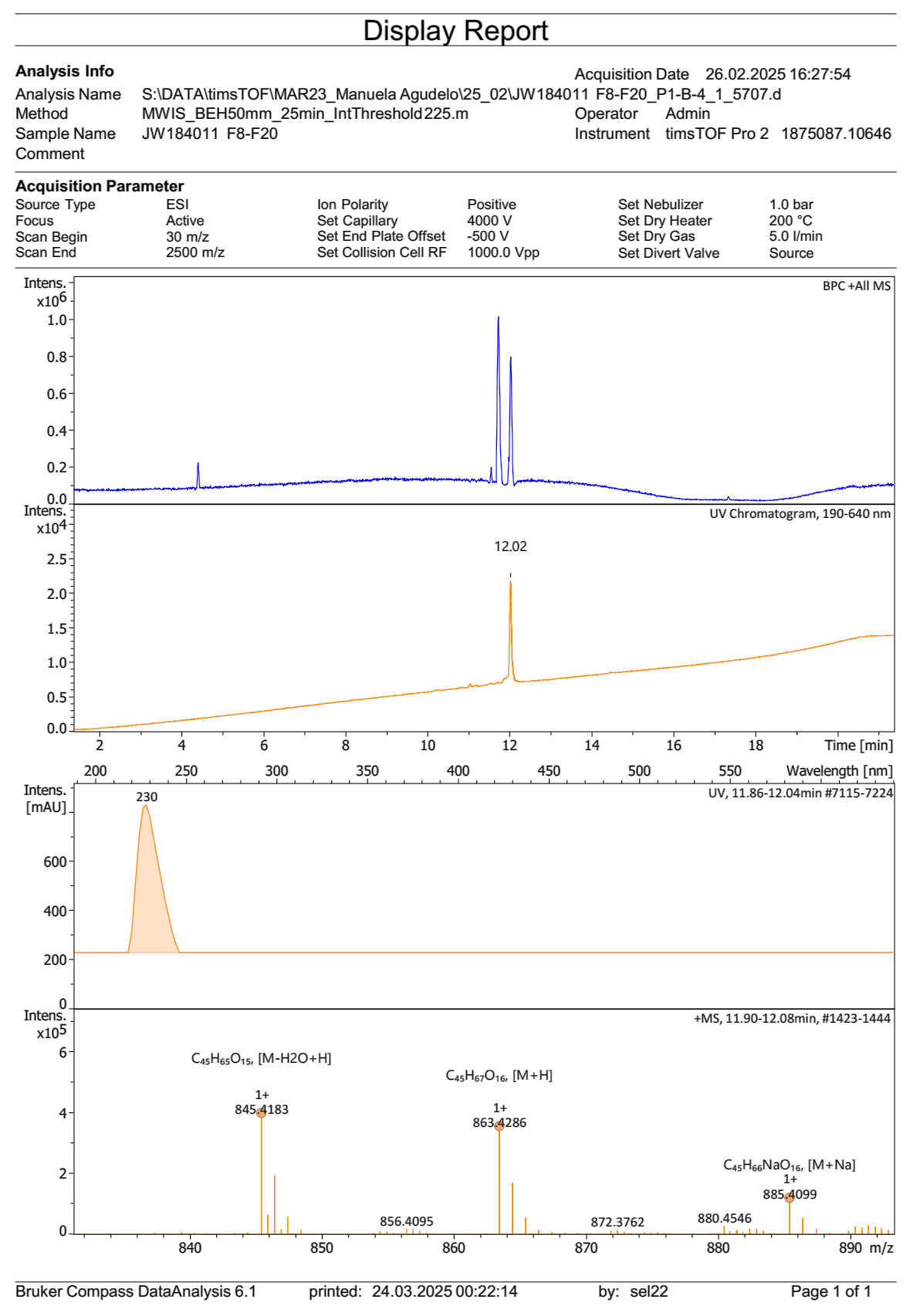


Figure S2. HR-ESI-MS of **1**.

Figure S3. ^1^H NMR spectrum of **1** in methanol-*d*_4_ at 700 MHz.

Figure S4. ^1^H–^1^H COSY spectrum of **1** in methanol-*d*_4_ at 700 MHz.

Figure S5. HMBC spectrum of **1** in methanol-*d*_4_ at 700 MHz.

Figure S6. HSQC spectrum of **1** in methanol-*d*_4_ at 700 MHz.

Figure S7. ROESY spectrum of **1** in methanol-*d*_4_ at 700 MHz.


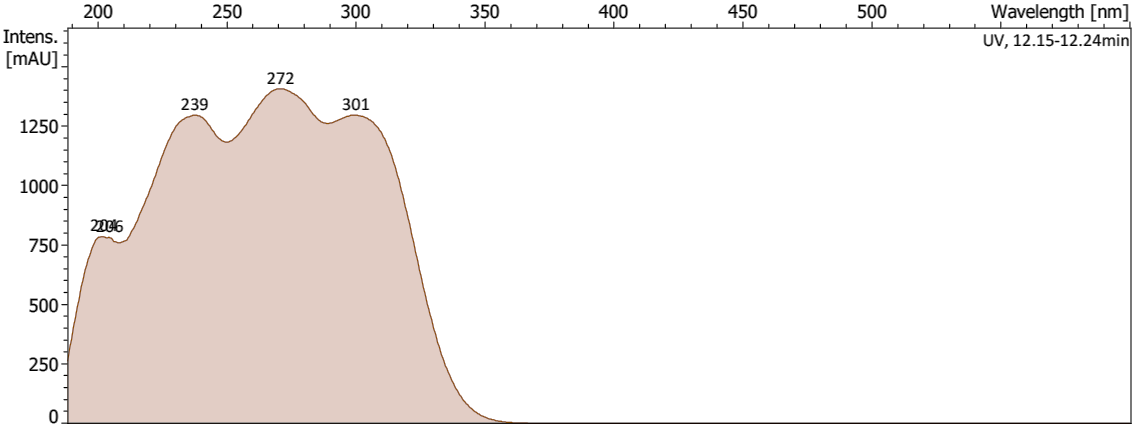

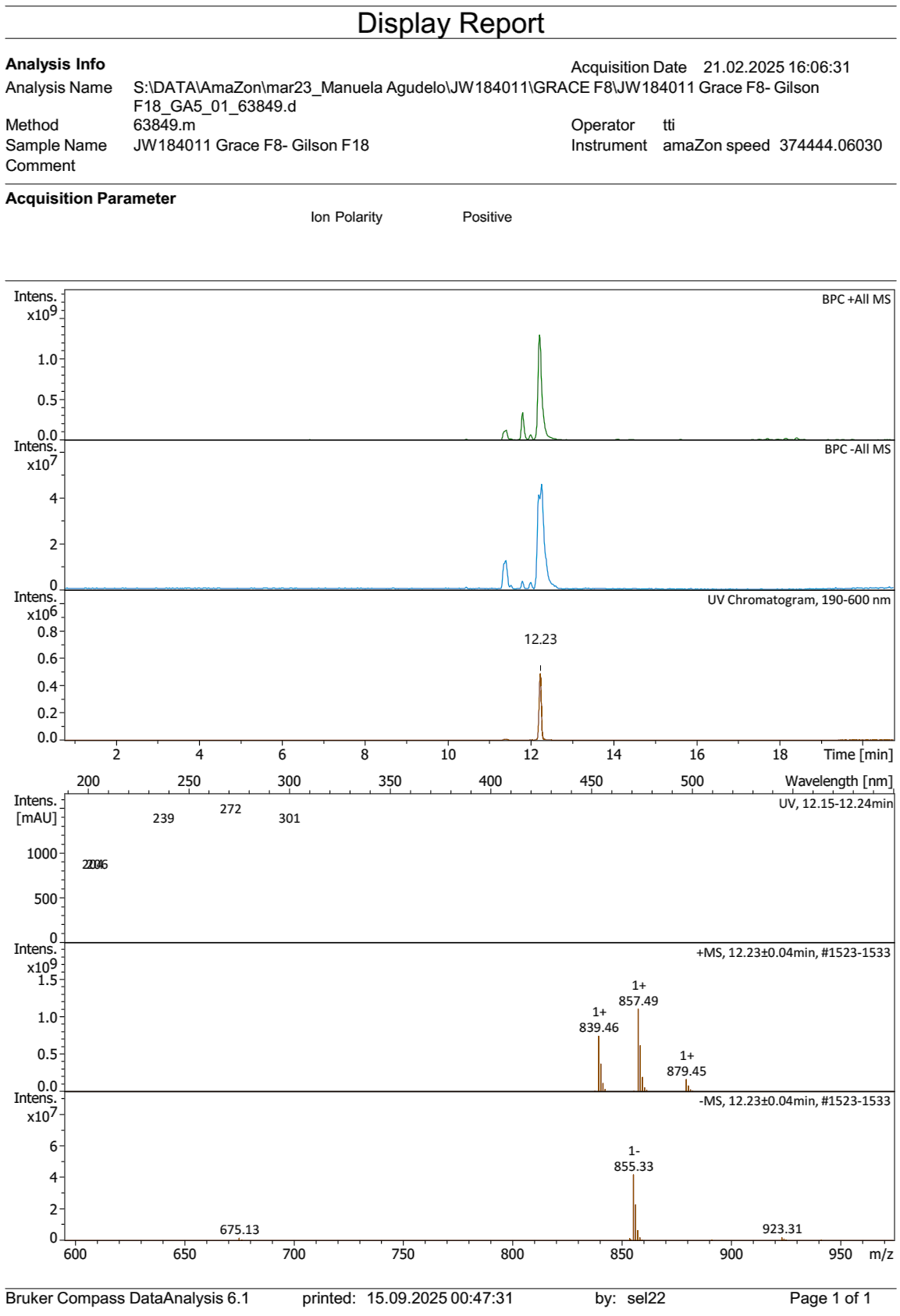


[M-H]^-^

[M+Na]^+^

[M+H]^+^

Figure S8. LR-ESI-MS of **2**.


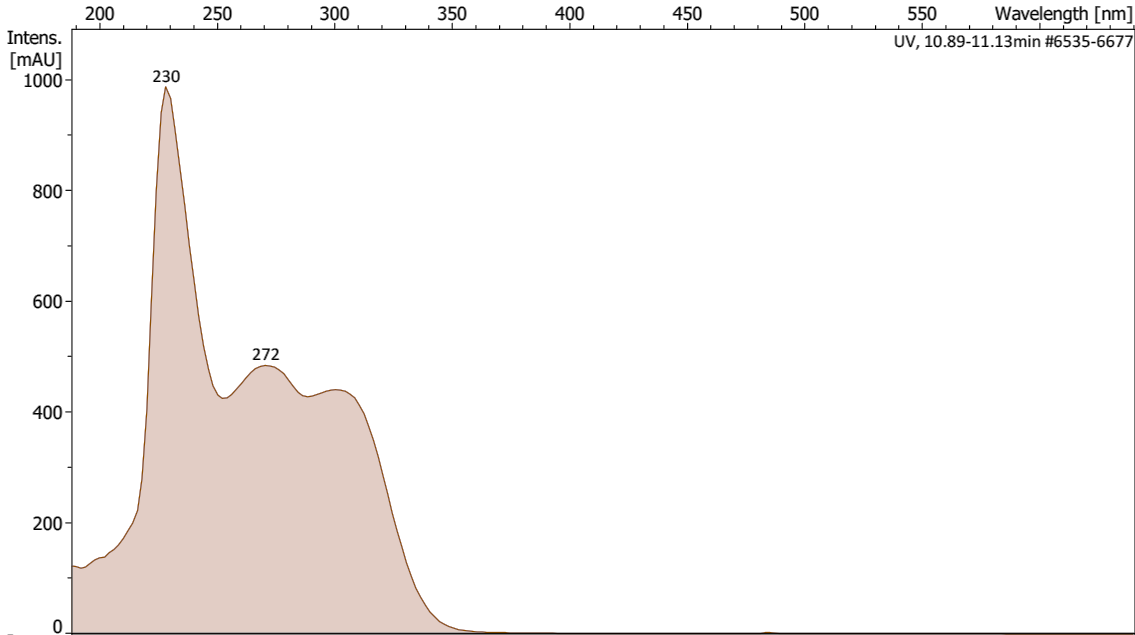

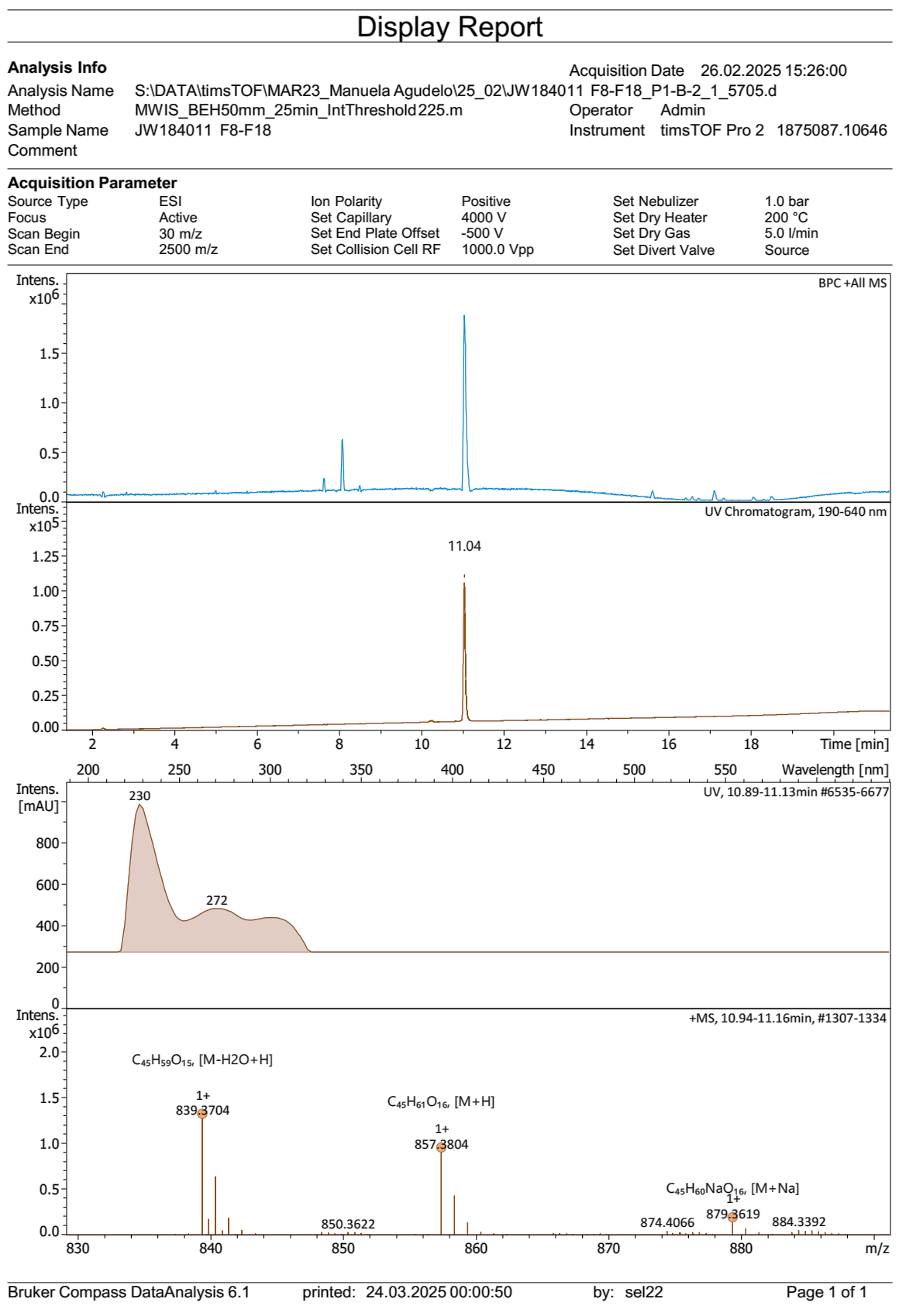


Figure S9. HR-ESI-MS of **2**.

Figure S10. ^1^H NMR spectrum of **2** in methanol-*d*_4_ at 600 MHz.

Figure S11. ^13^C NMR spectrum of **2** in methanol-*d*_4_ at 150 MHz.

Figure S12. ^1^H–^1^H COSY spectrum of **2** in methanol-*d*_4_ at 600 MHz.

Figure S13. HMBC spectrum of **2** in methanol-*d*_4_ at 600 MHz.

Figure S14. HSQC spectrum of **2** in methanol-*d*_4_ at 600 MHz.

Figure S15. ROESY spectrum of **2** in methanol-*d*_4_ at 600 MHz.


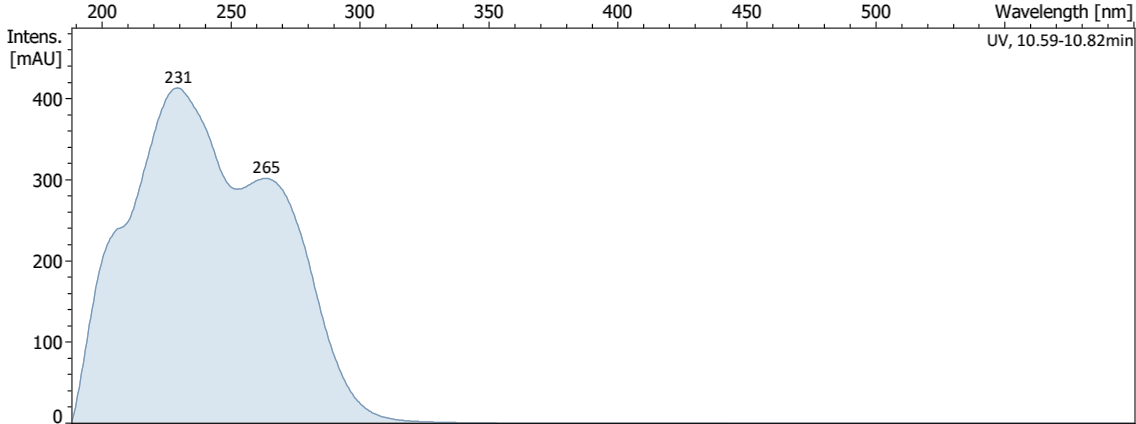

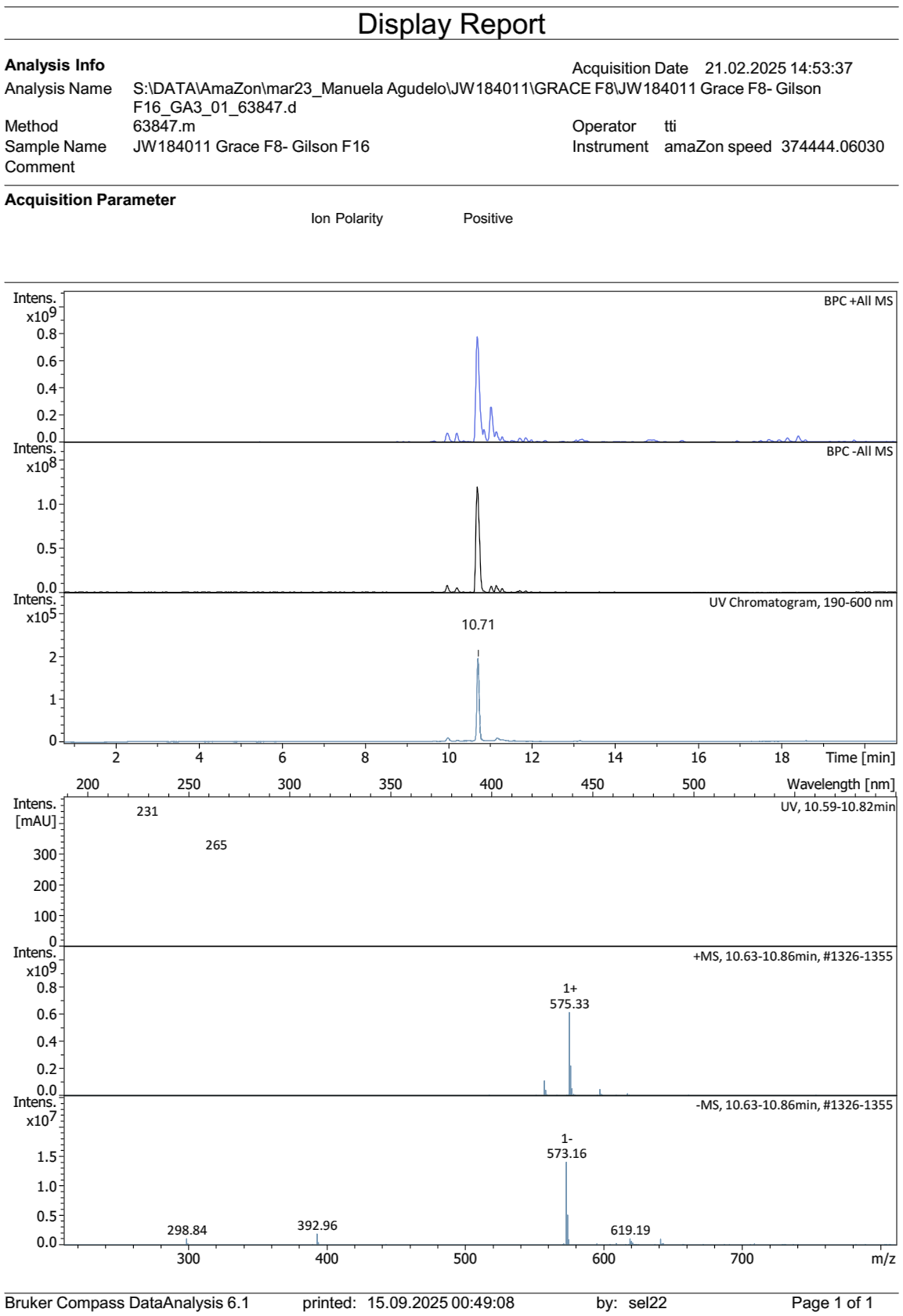


[M-H]^-^

[M+H]^+^

[2M+Na]^+^

Figure S16. LR-ESI-MS of **3**.


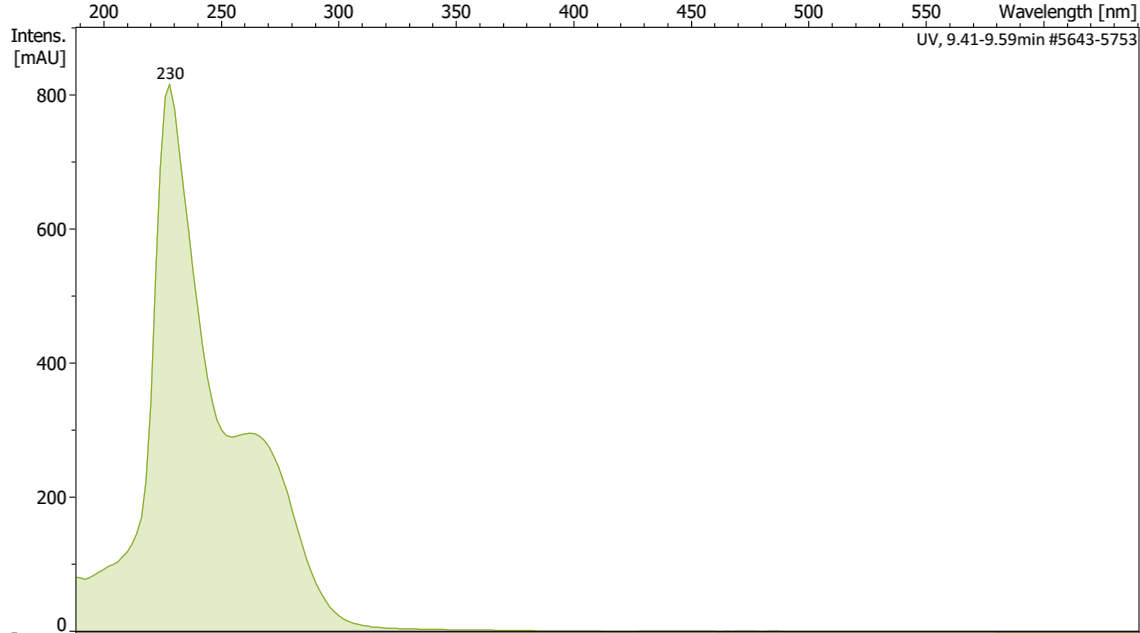

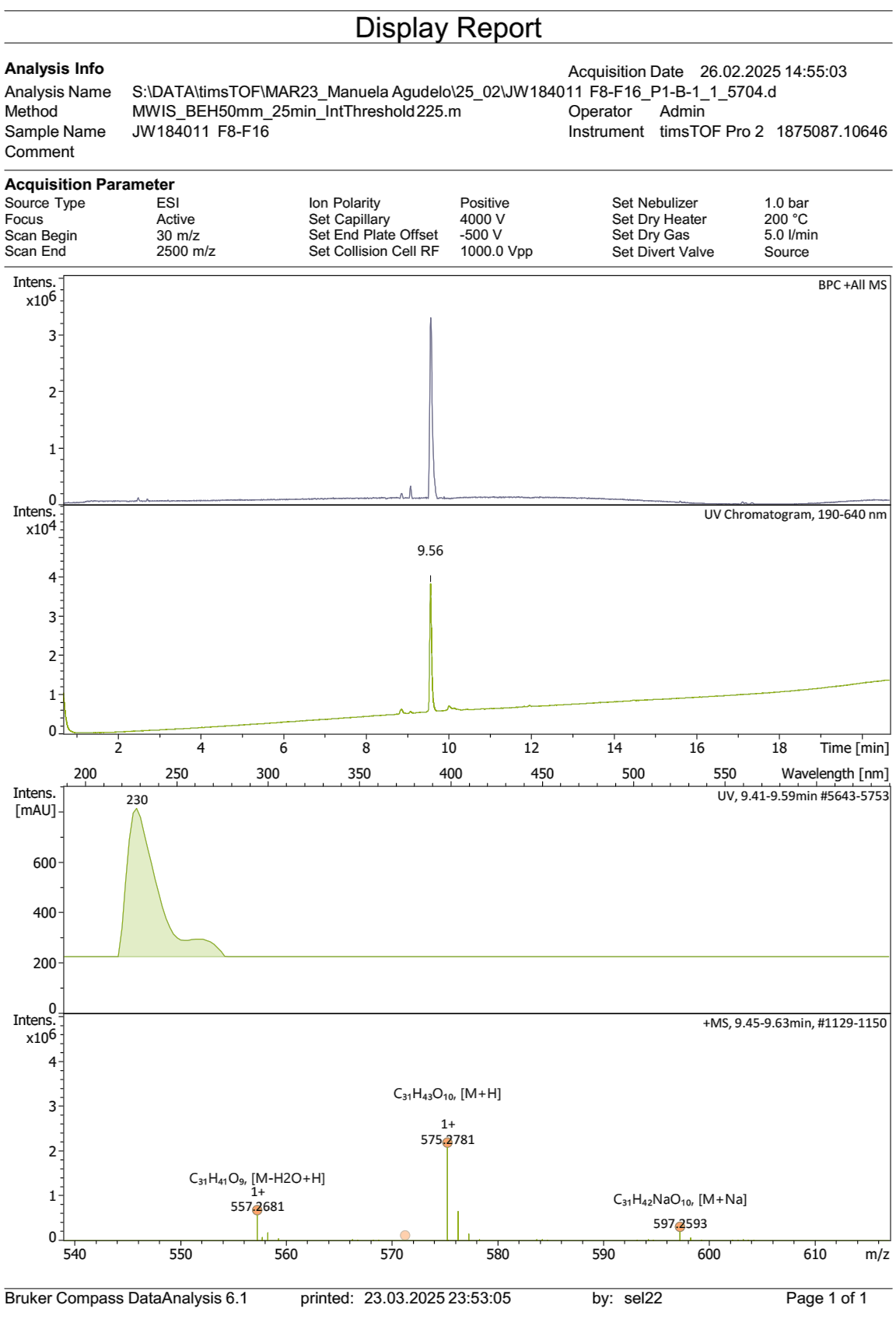


Figure S17. HR-ESI-MS of **3**.

Figure S18. ^1^H NMR spectrum of **3** in methanol-*d*_4_ at 700 MHz.

Figure S19. DEPTQ spectrum of **3** in methanol-*d*_4_ at 175 MHz.

Figure S20. ^1^H–^1^H COSY spectrum of **3** in methanol-*d*_4_ at 700 MHz.

Figure S21. HMBC spectrum of **3** in methanol-*d*_4_ at 700 MHz.

Figure S22. HSQC spectrum of **3** in methanol-*d*_4_ at 700 MHz.

Figure S23. ROESY spectrum of **3** in methanol-*d*_4_ at 700 MHz.


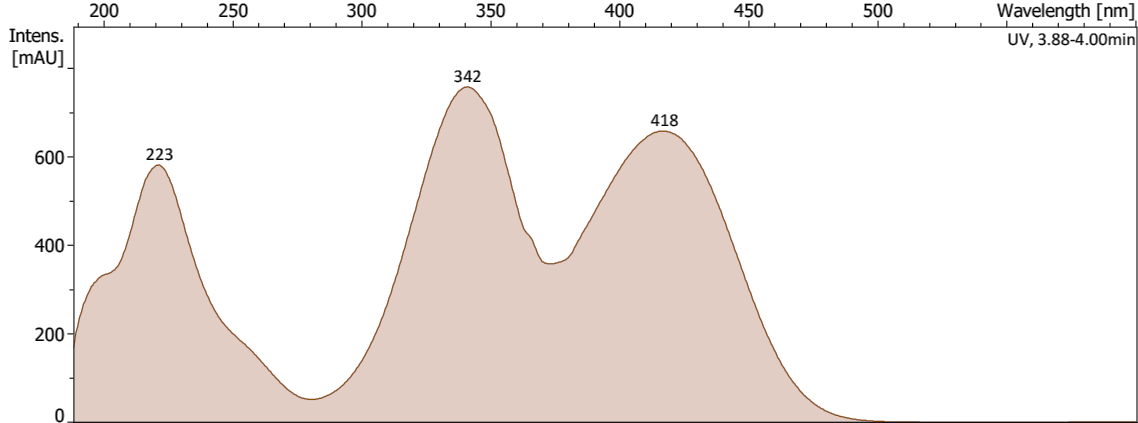

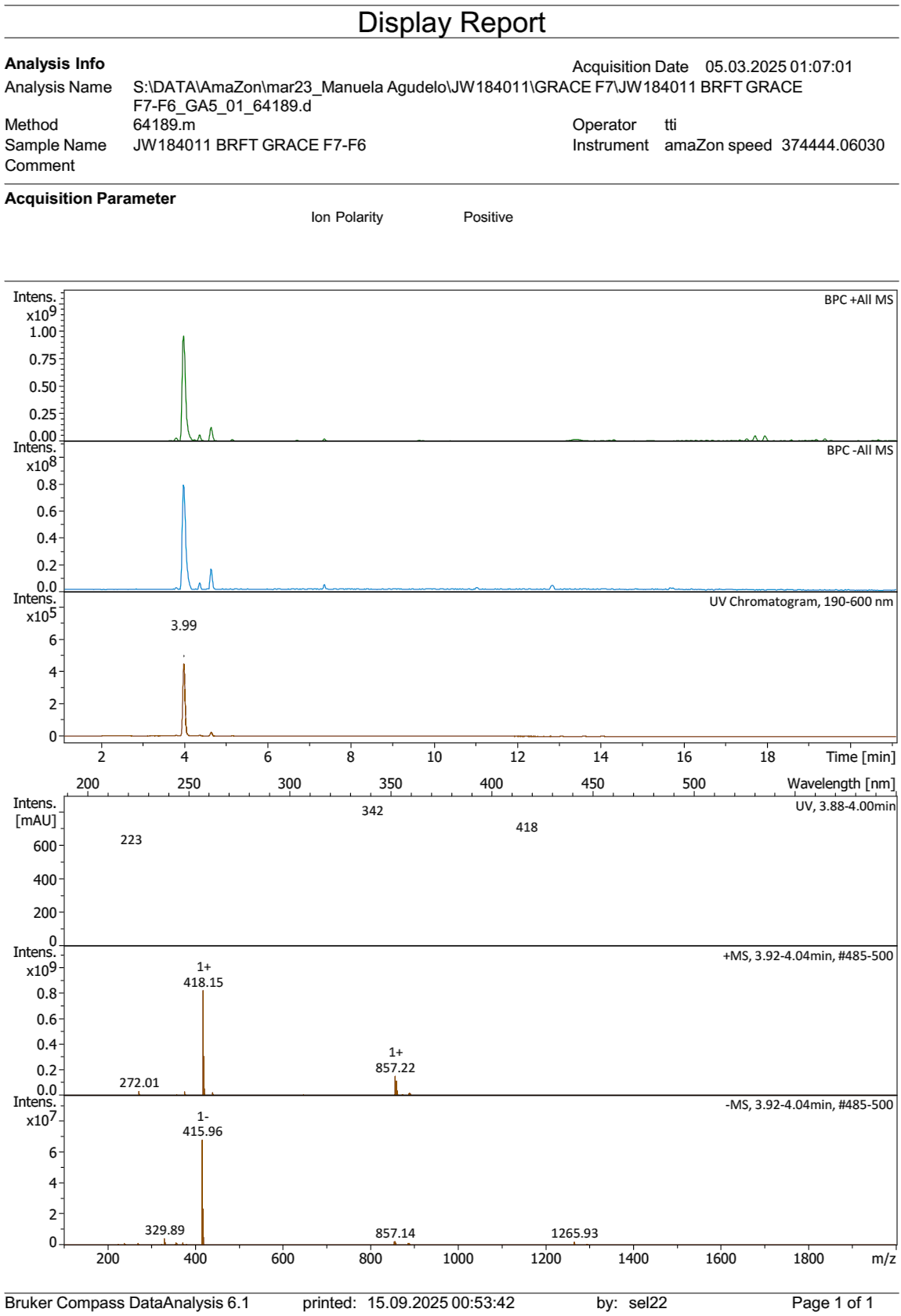


[M-H]^-^

[M+H]^+^

Figure S24. LR-ESI-MS of **4**.


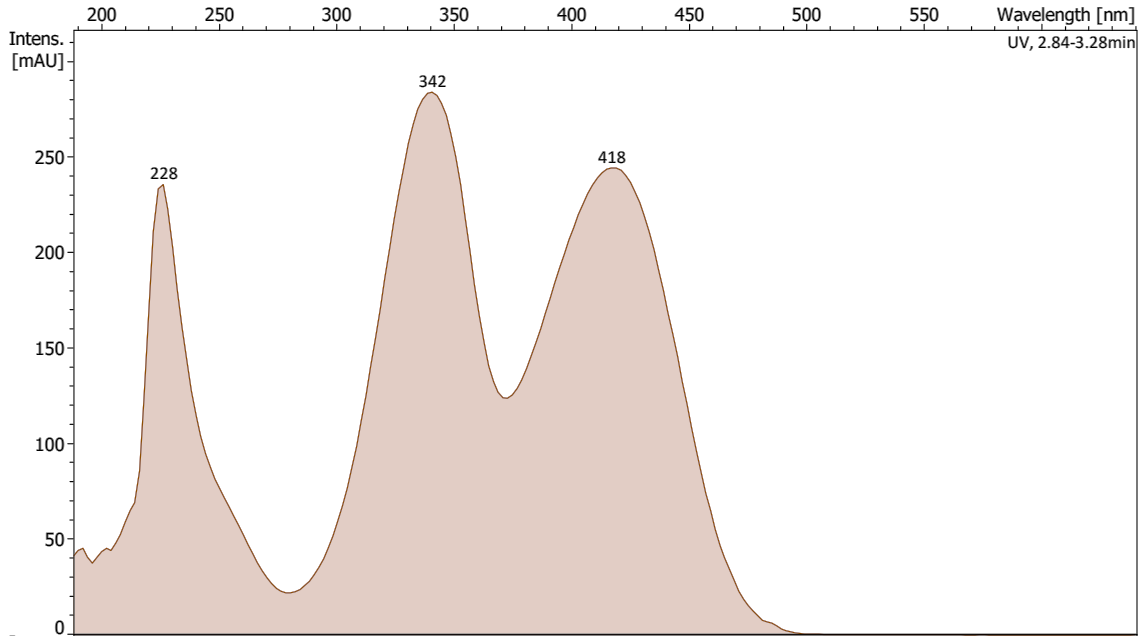

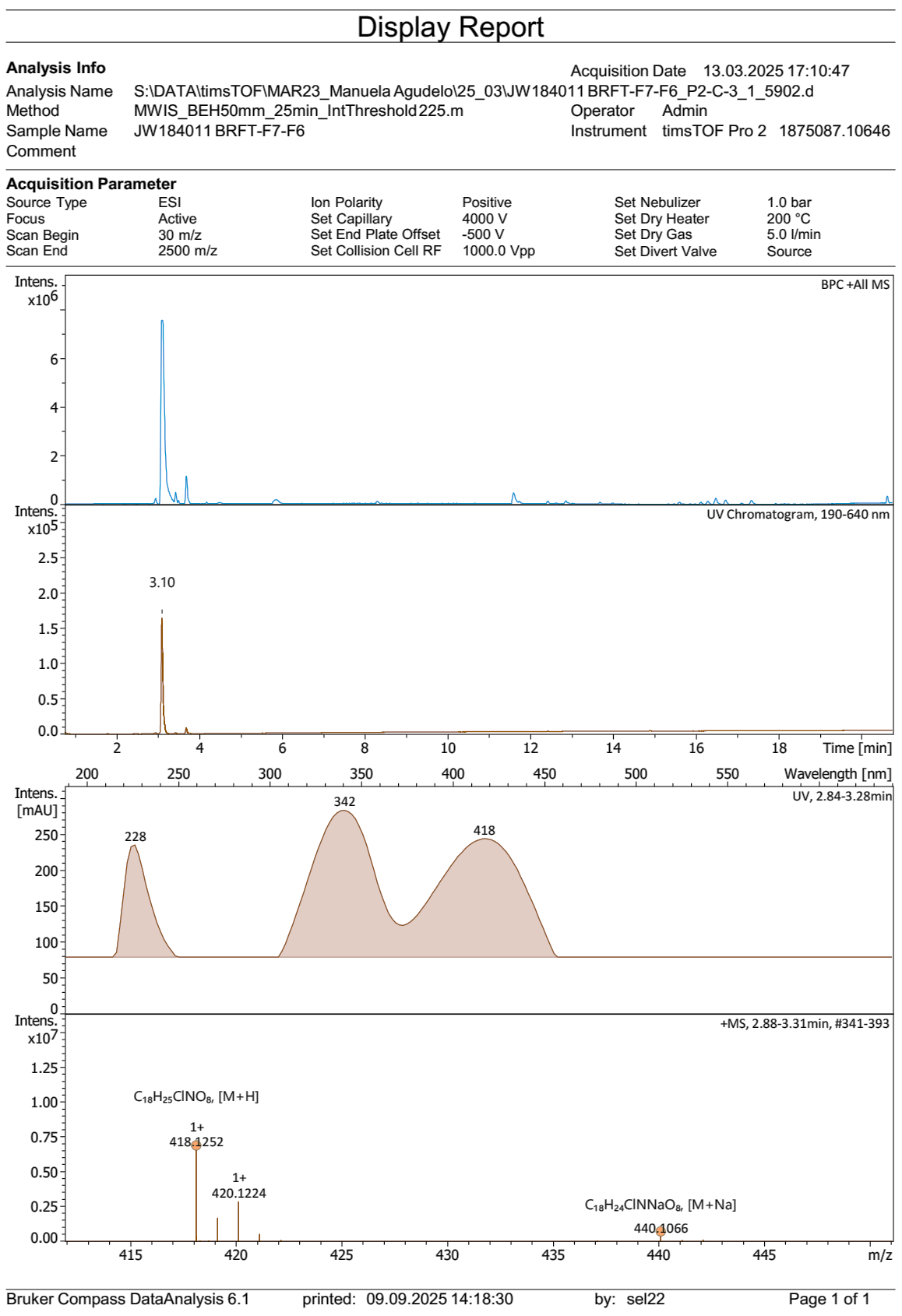


Figure S25. HR-ESI-MS of **4**.

Figure S26. ^1^H NMR spectrum of **4** in methanol-*d*_4_ at 500 MHz.

Figure S27. DEPTQ spectrum of **4** in methanol-*d*_4_ at 125 MHz.

Figure S28. ^1^H–^1^H COSY spectrum of **4** in methanol-*d*_4_ at 500 MHz.

Figure S29. HMBC spectrum of **4** in methanol-*d*_4_ at 500 MHz.

Figure S30. HSQC spectrum of **4** in methanol-*d*_4_ at 500 MHz.

Figure S31. ROESY spectrum of **4** in methanol-*d*_4_ at 500 MHz.


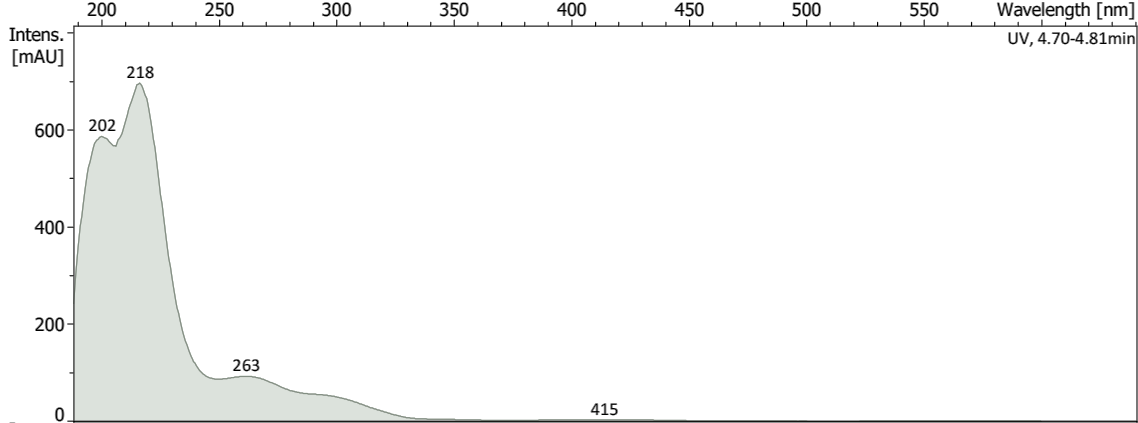

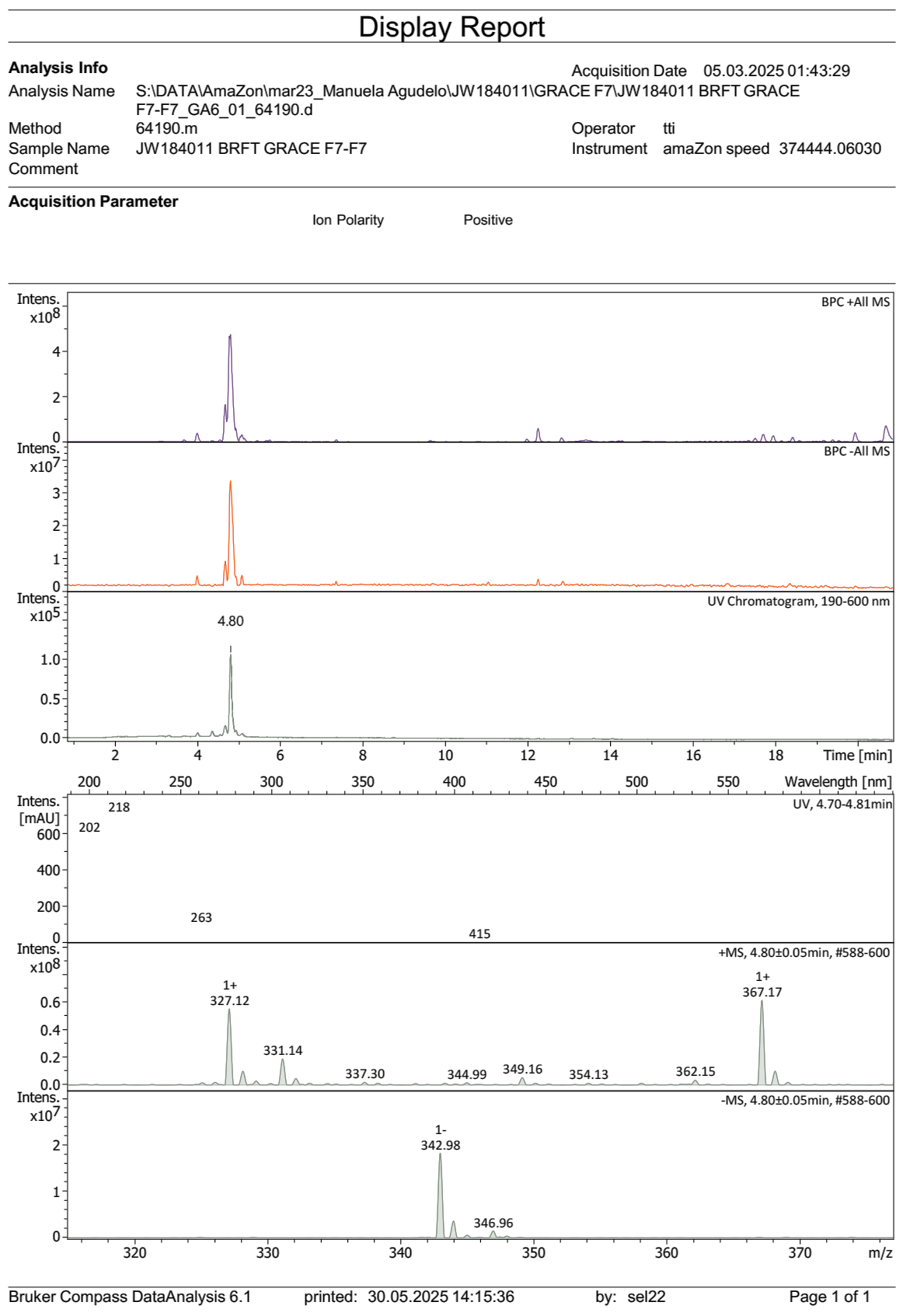


[M-H_2_O+H]^+^

[M+Na]^+^

[M+H]^+^

[M-H]^-^

Figure S32. LR-ESI-MS of **5**.


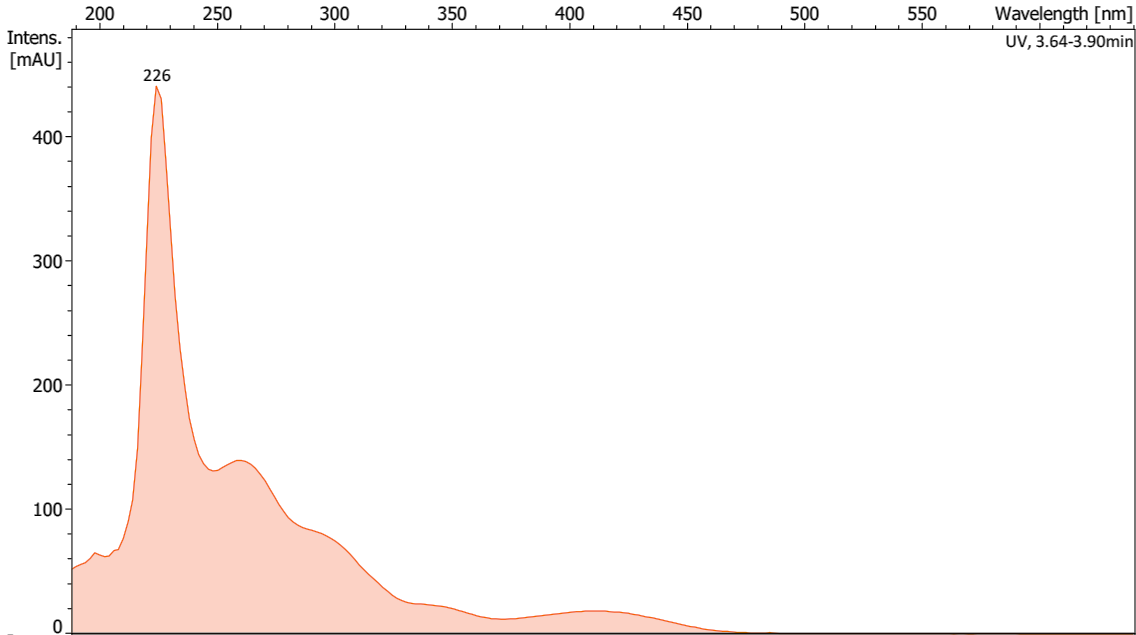

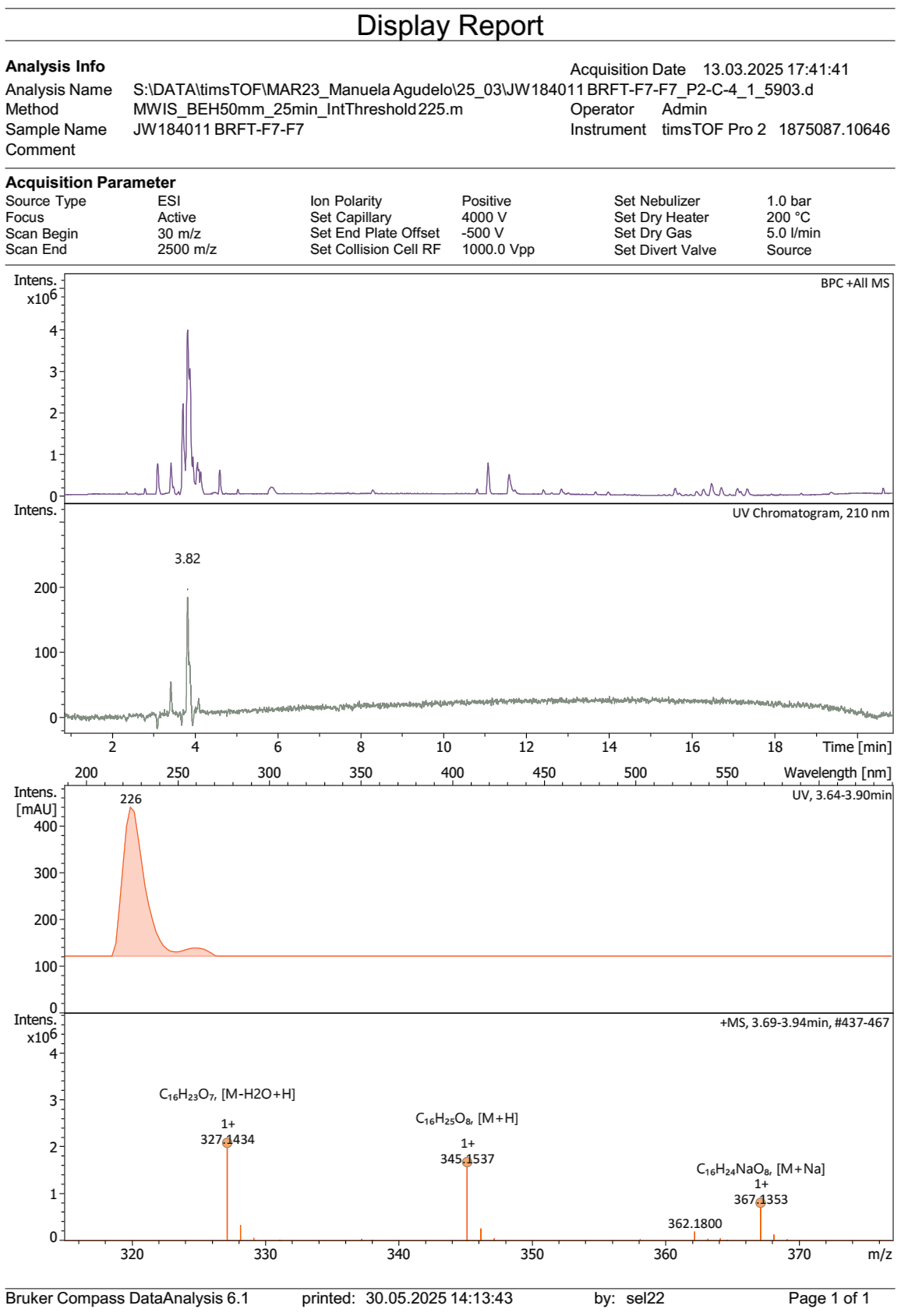


Figure S33. LR-ESI-MS of **5**.

Figure S34. ^1^H NMR spectrum of **5** in methanol-*d*_4_ at 600 MHz.

Figure S35. DEPTQ spectrum of **5** in methanol-*d*_4_ at 150 MHz.

Figure S36. ^1^H–^1^H COSY spectrum of **5** in methanol-*d*_4_ at 600 MHz.

Figure S37. HMBC spectrum of **5** in methanol-*d*_4_ at 600 MHz.

Figure S38. HSQC spectrum of **5** in methanol-*d*_4_ at 600 MHz.

Figure S39. ROESY spectrum of **5** in methanol-*d*_4_ at 600 MHz.


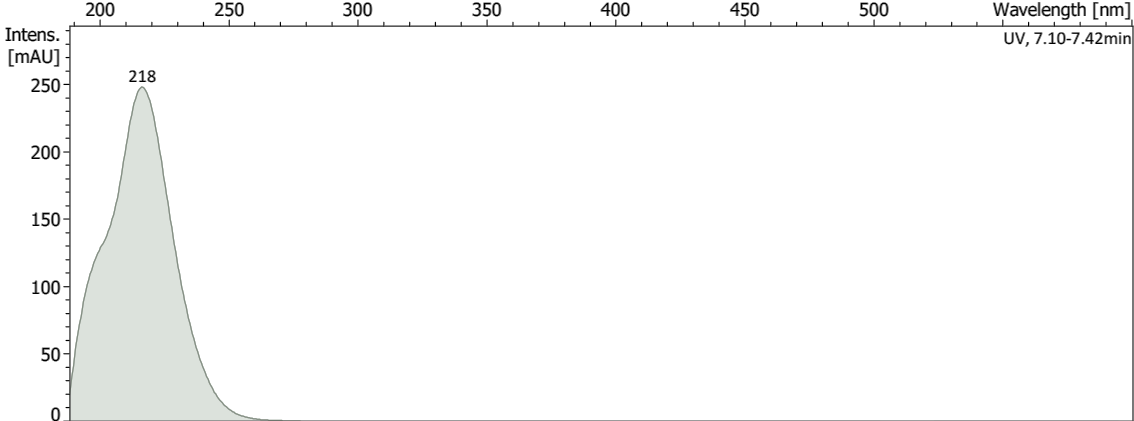

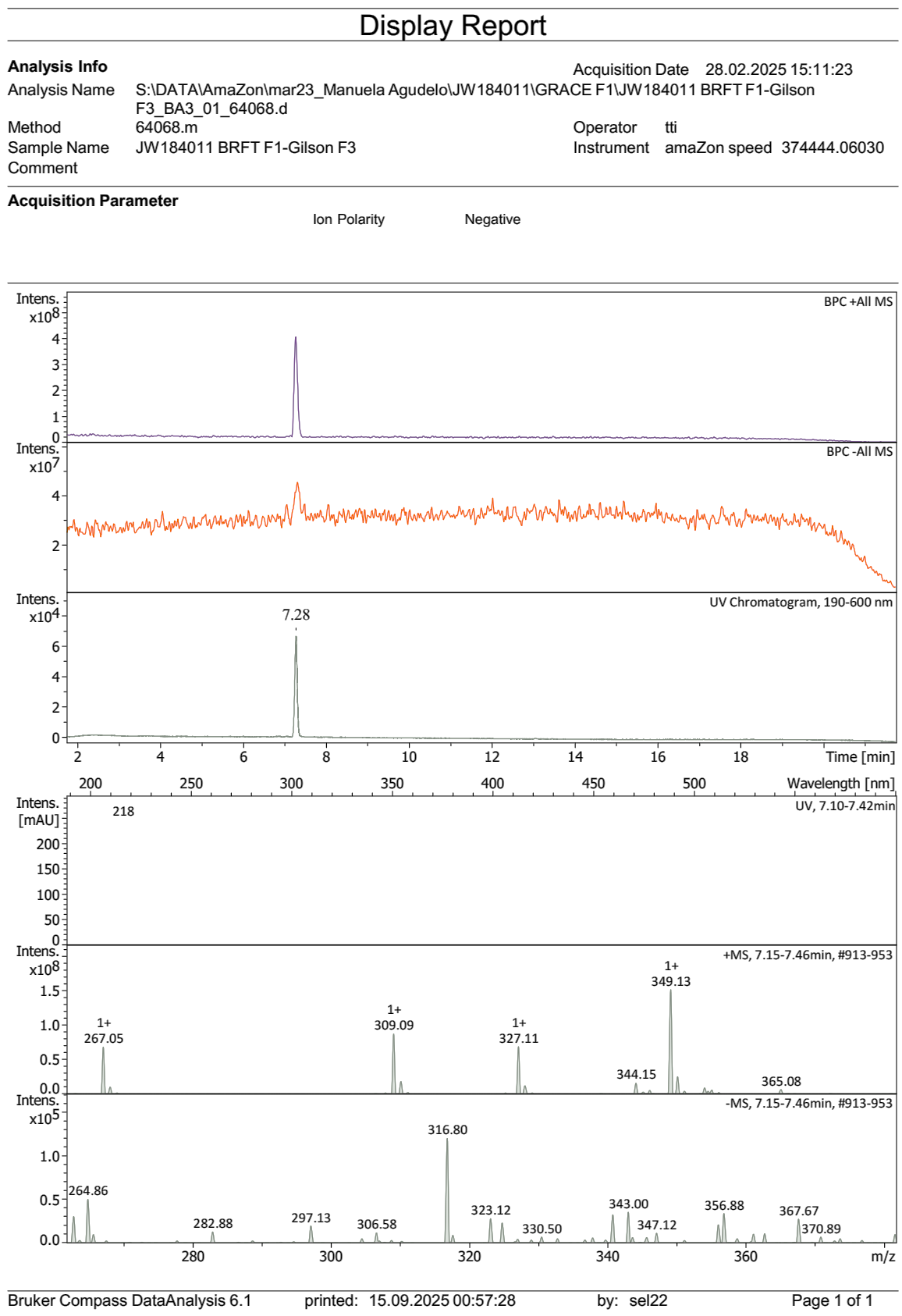


[M-H_2_O+H]^+^

[M+Na]^+^

[M+H]^+^

Figure S40. LR-ESI-MS of **6**.


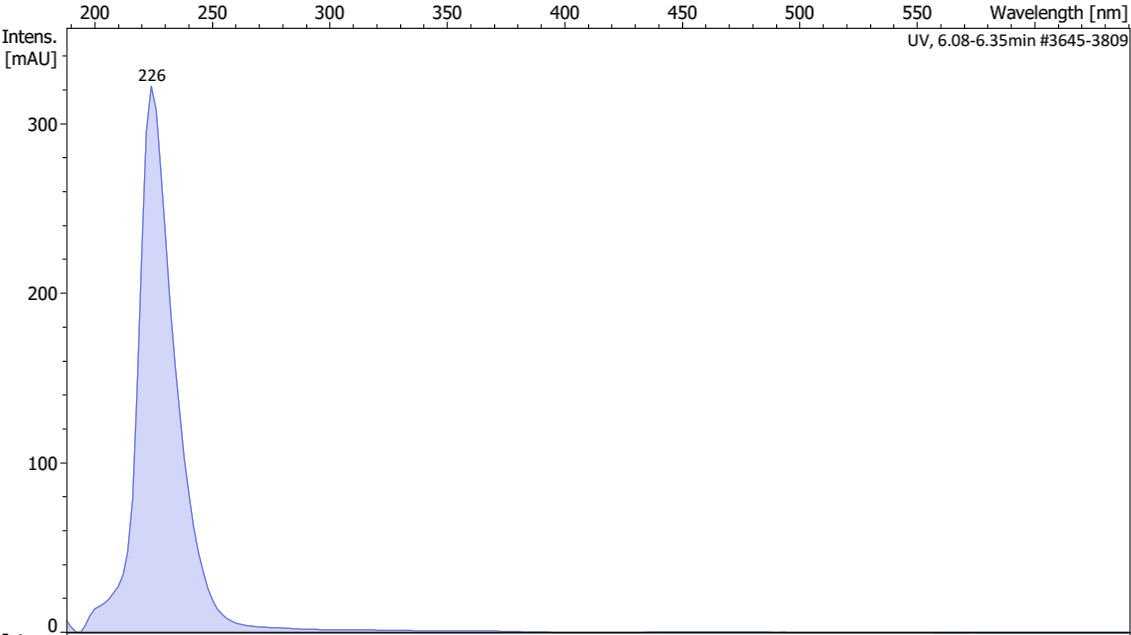

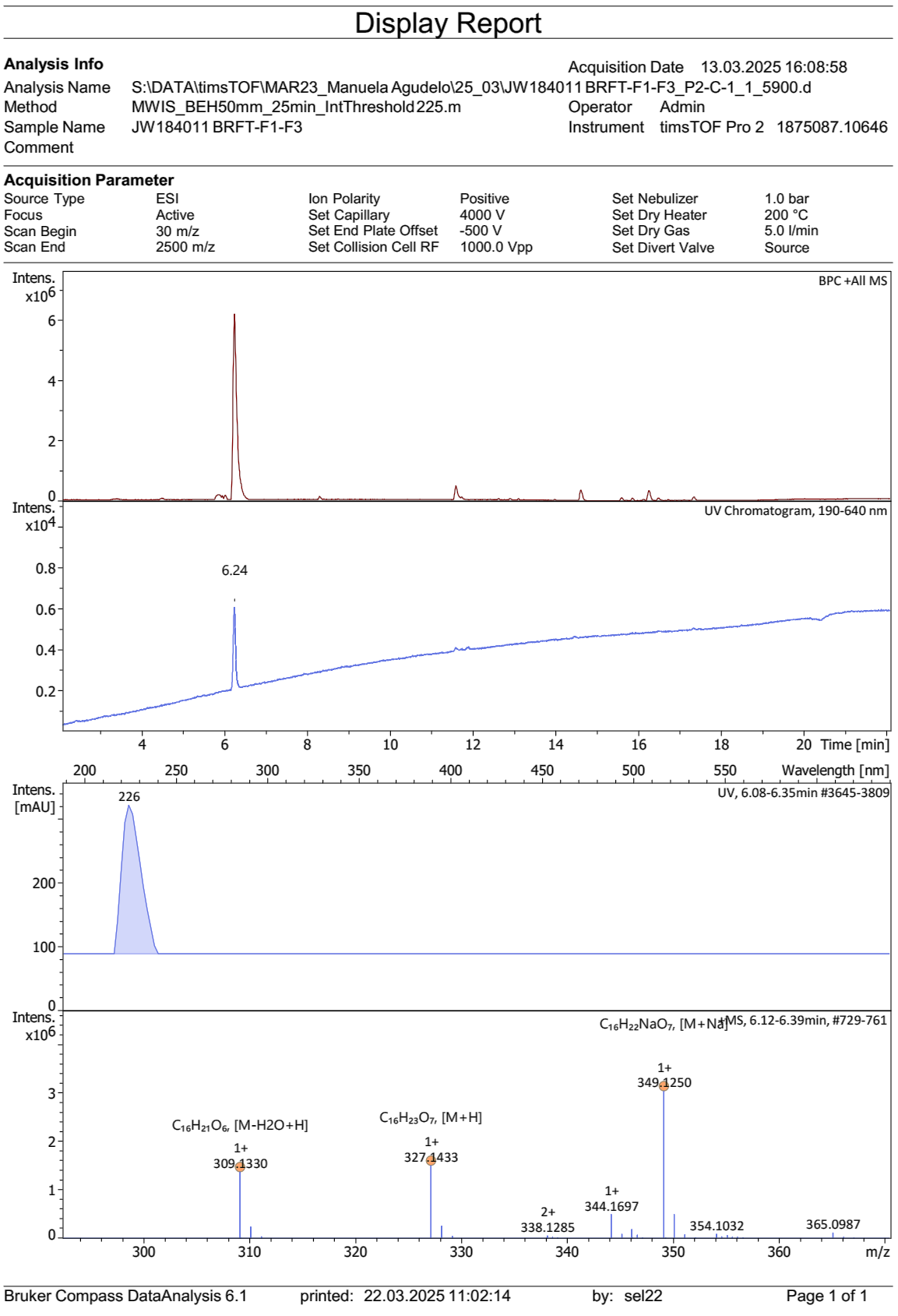


Figure S41. HR-ESI-MS of **6**.

Figure S42. ^1^H NMR spectrum of **6** in methanol-*d*_4_ at 500 MHz.

Figure S43. DEPTQ spectrum of **6** in methanol-*d*_4_ at 125 MHz.

Figure S44. ^1^H–^1^H COSY spectrum of **6** in methanol-*d*_4_ at 500 MHz.

Figure S45. HMBC spectrum of **6** in methanol-*d*_4_ at 500 MHz.

Figure S46. HSQC spectrum of **6** in methanol-*d*_4_ at 500 MHz.

Figure S47. ROESY spectrum of **6** in methanol-*d*_4_ at 500 MHz.


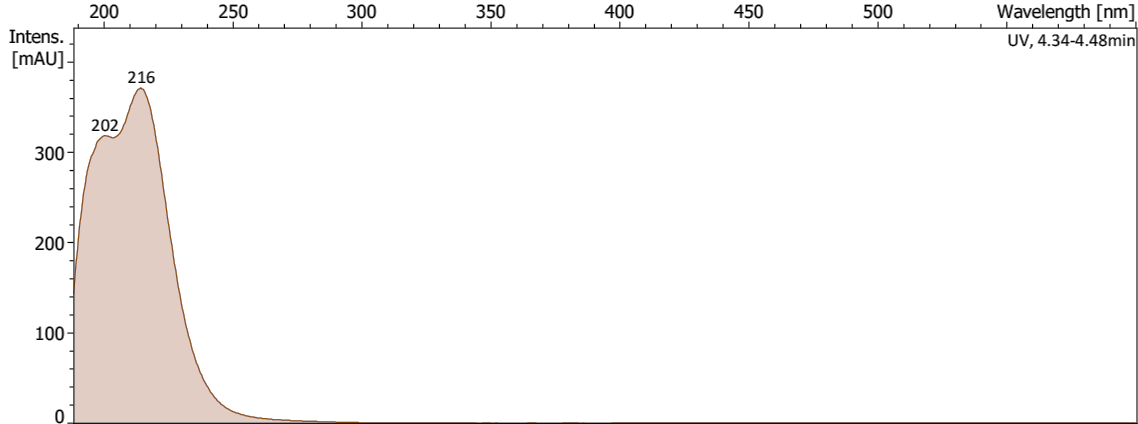

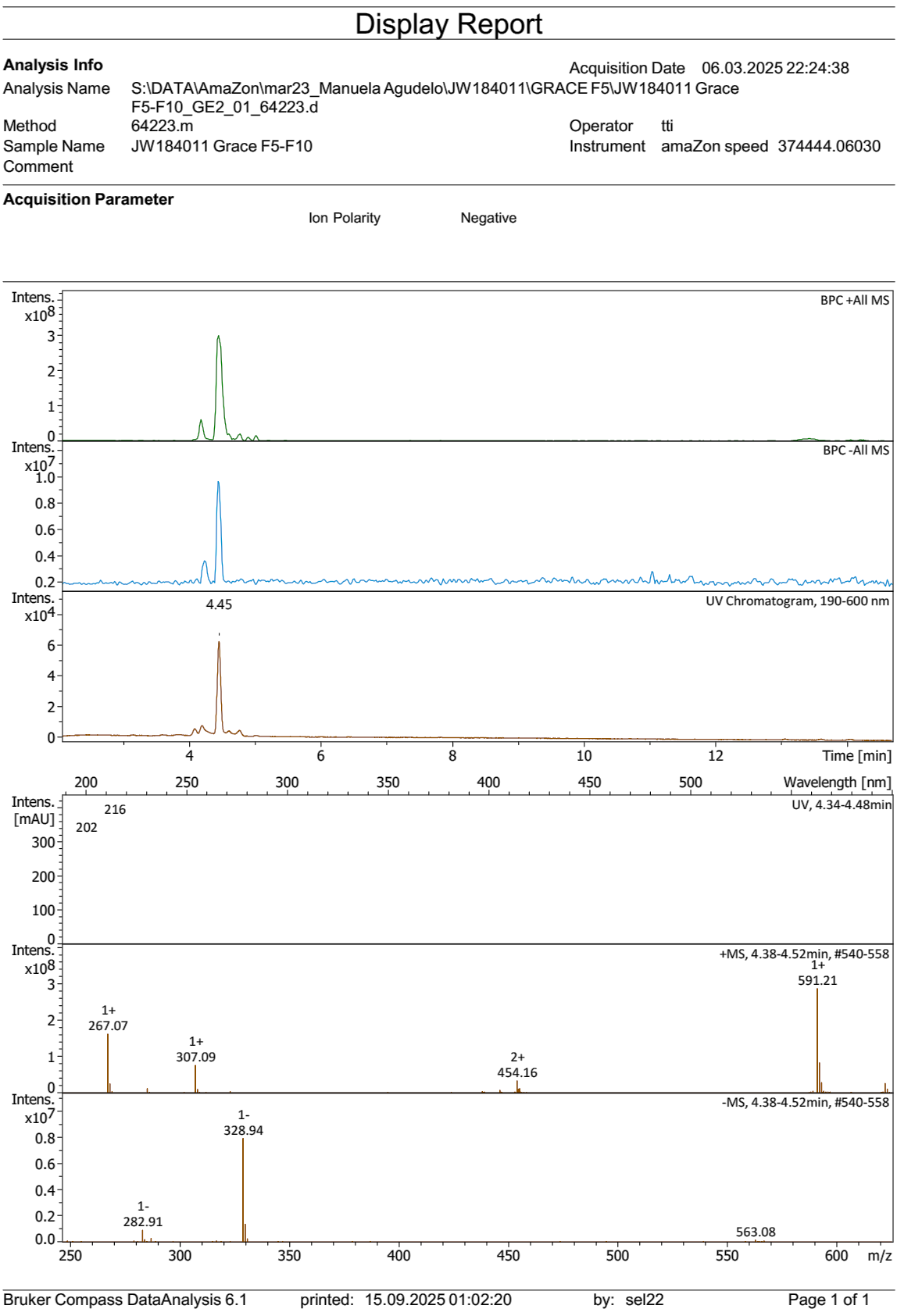


[M+H]^+^

**1+**

**285.08**

[M-H_2_O+H]^+^

[M-H]^-^

[M+Na]^+^

[2M+H]^+^

Figure S48. LR-ESI-MS of **7**.


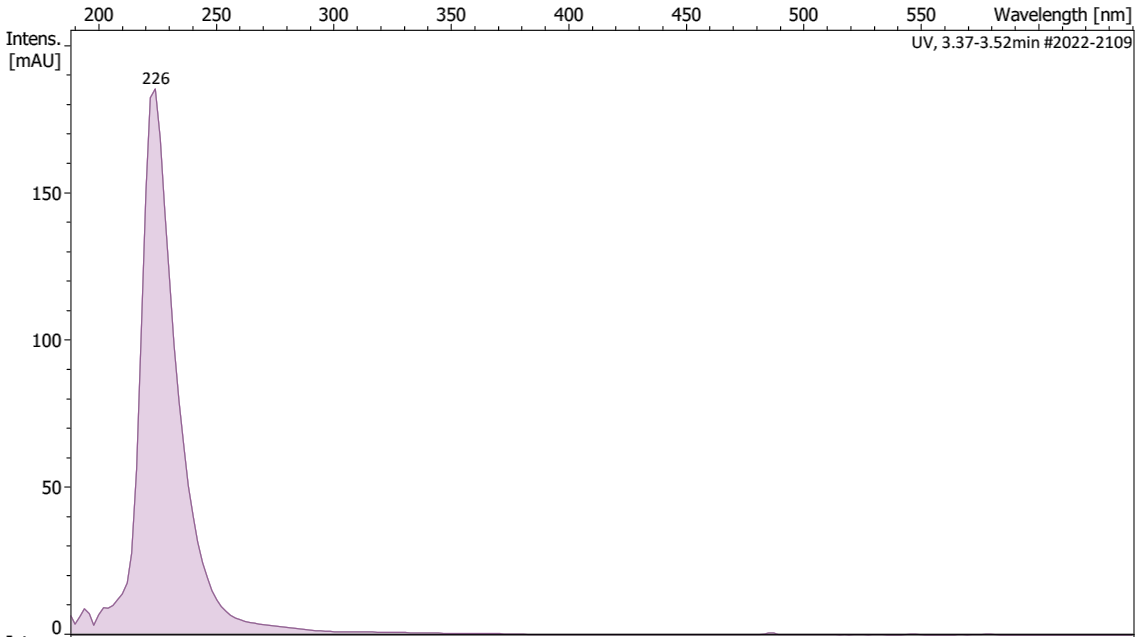

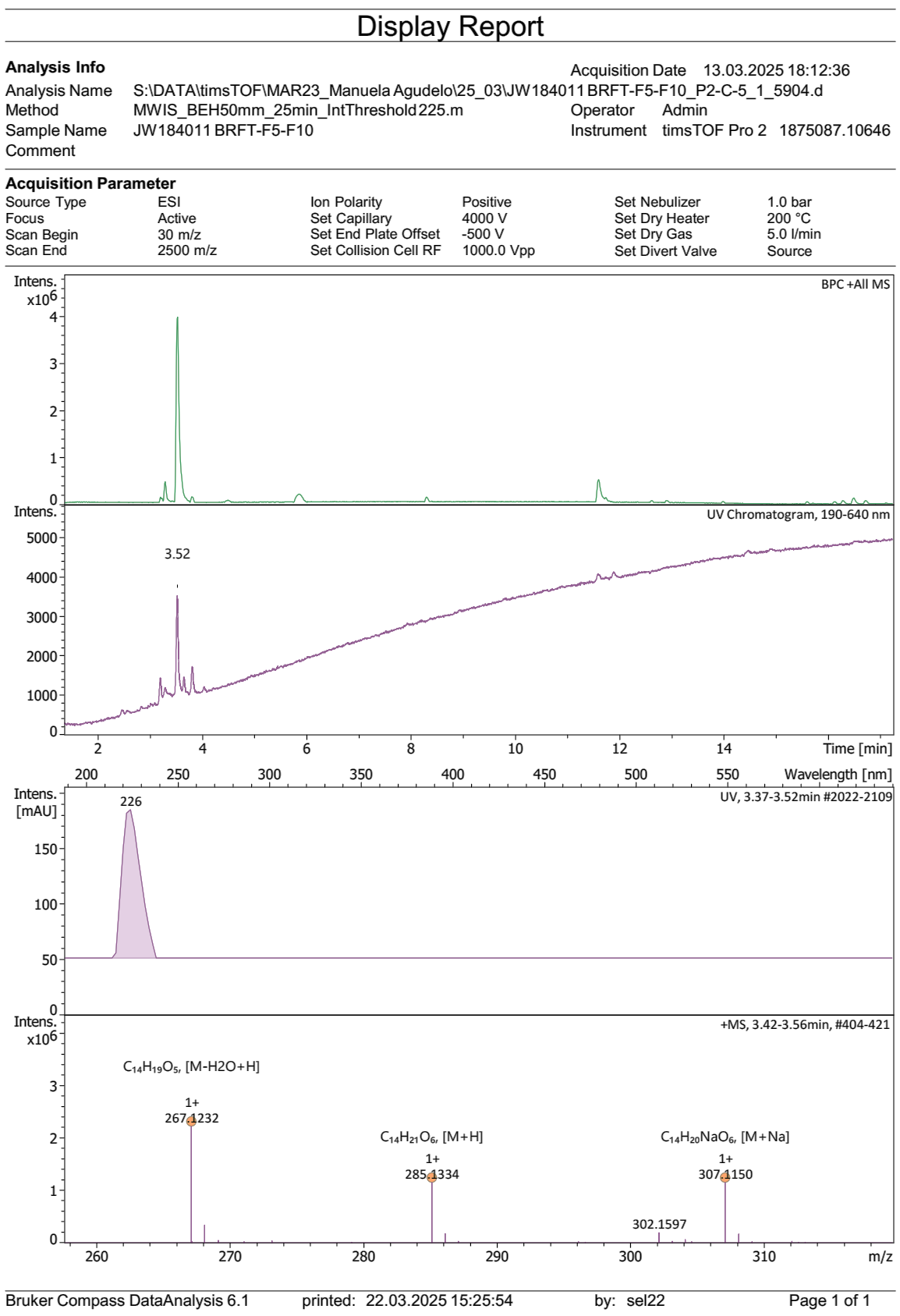


Figure S49. HR-ESI-MS of **7**.

Figure S50. ^1^H NMR spectrum of **7** in methanol-*d*_4_ at 600 MHz.

Figure S51. DEPTQ spectrum of **7** in methanol-*d*_4_ at 150 MHz.

Figure S52. ^1^H–^1^H COSY spectrum of **7** in methanol-*d*_4_ at 600 MHz.

Figure S53. HMBC spectrum of **7** in methanol-*d*_4_ at 600 MHz.

Figure S54. HSQC spectrum of **7** in methanol-*d*_4_ at 600 MHz.

Figure S55. ROESY spectrum of **7** in methanol-*d*_4_ at 600 MHz.


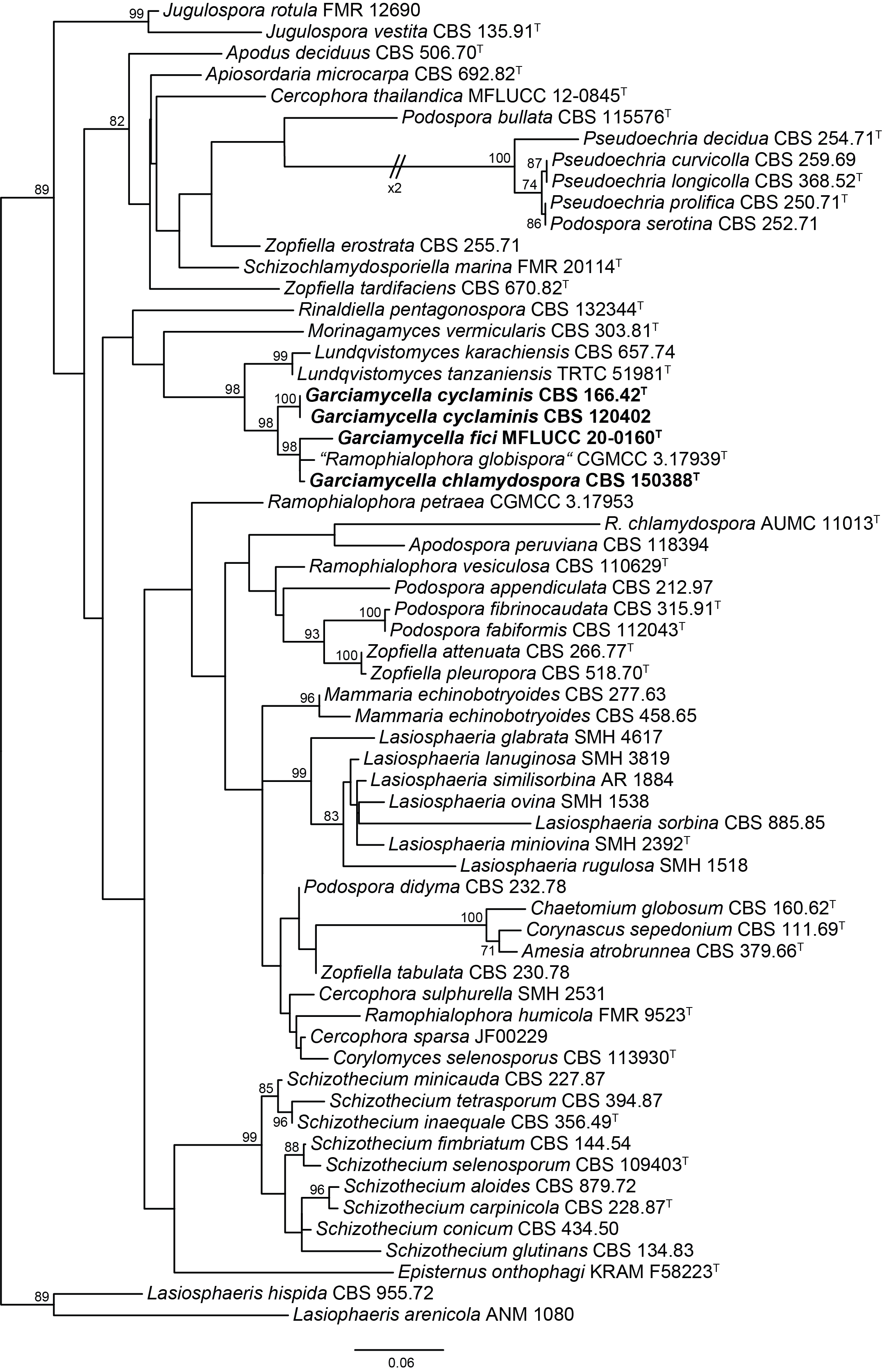


Figure S56. RAxML phylogram obtained from the internal transcribed spacer region (ITS) sequences


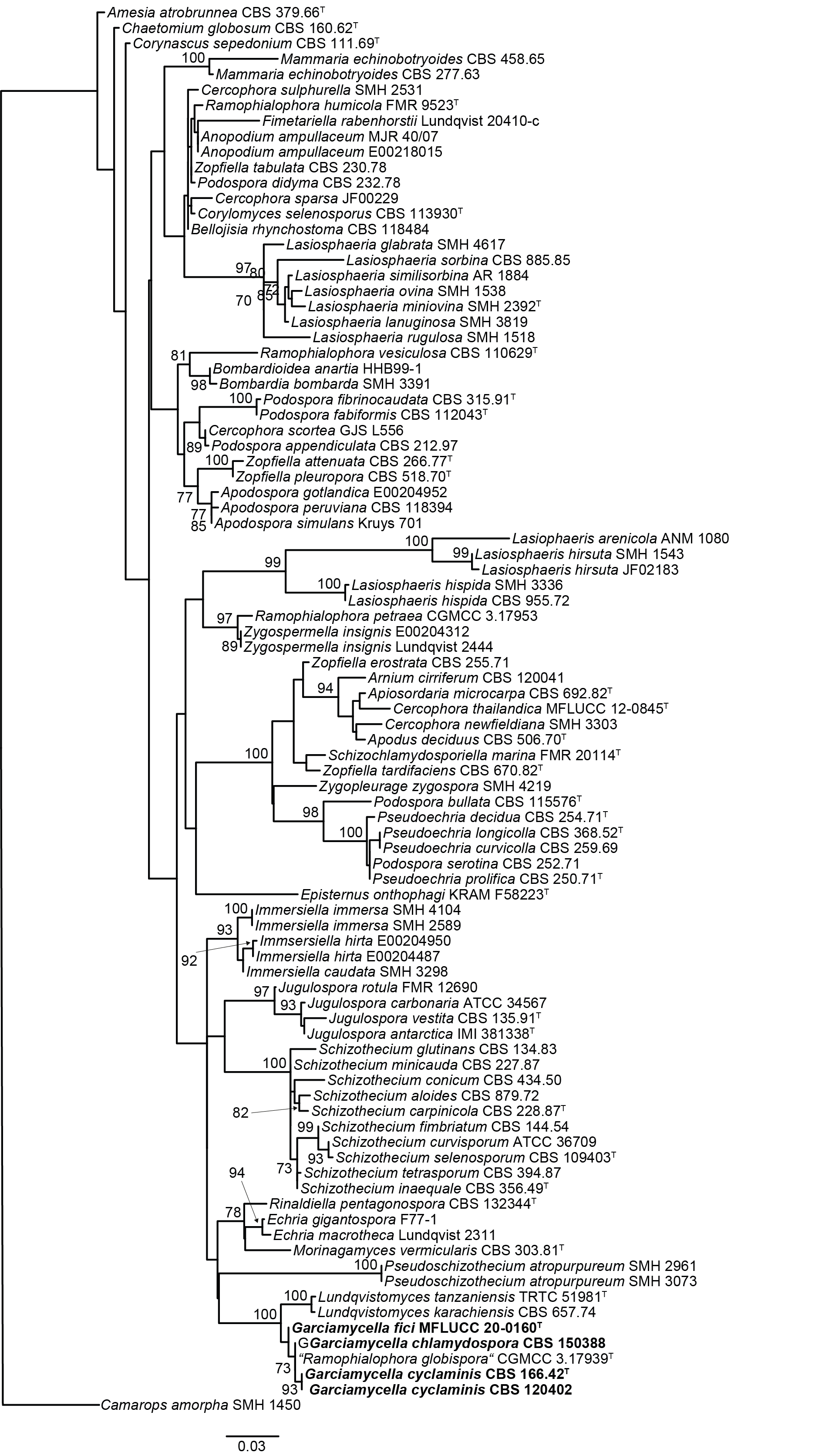


Figure S57. RAxML phylogram obtained from the nuclear rDNA large subunit (LSU) sequences


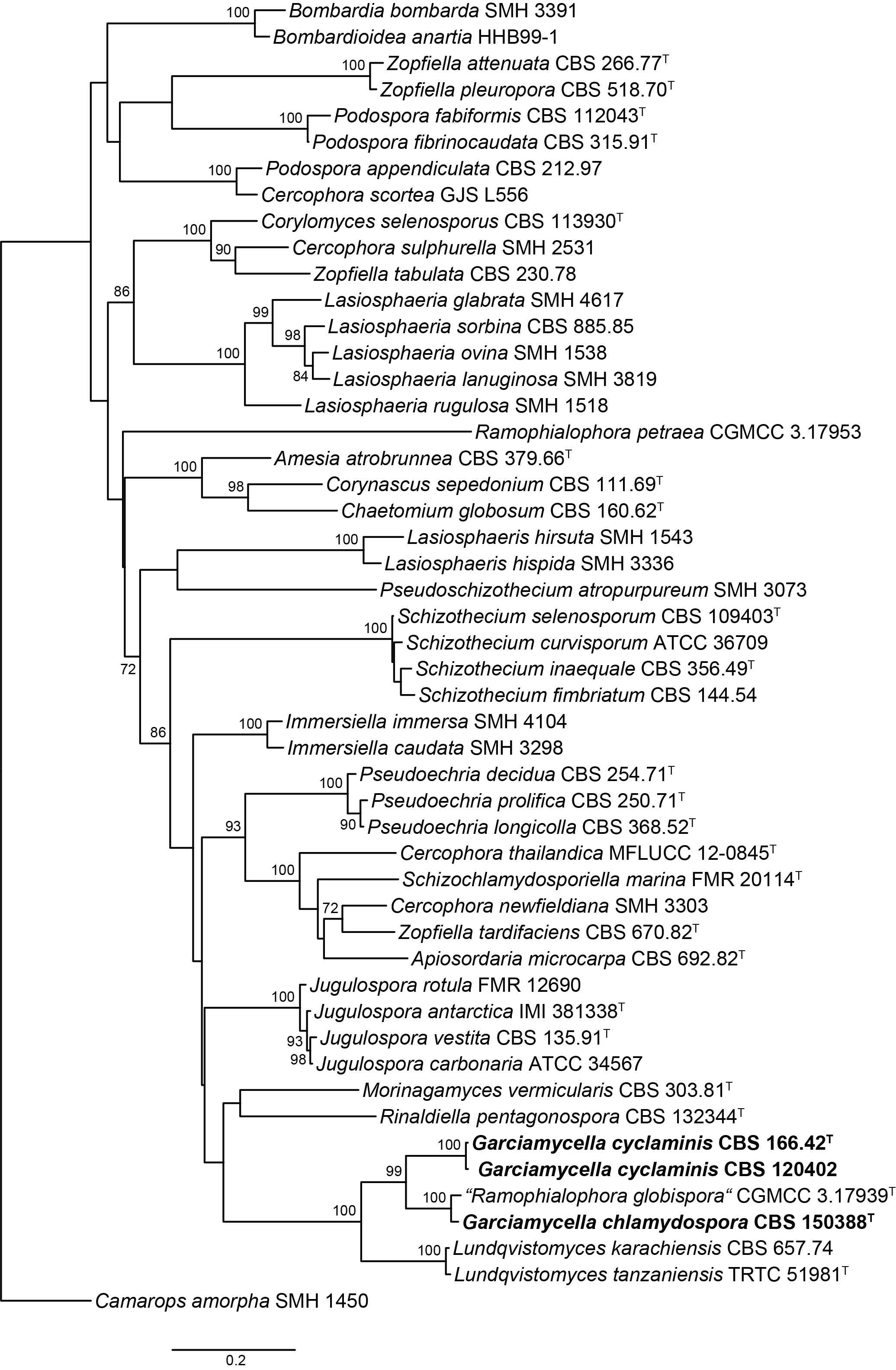


Figure S58. RAxML phylogram obtained from the fragment of ribosomal polymerase II subunit 2 (rpb2) sequences


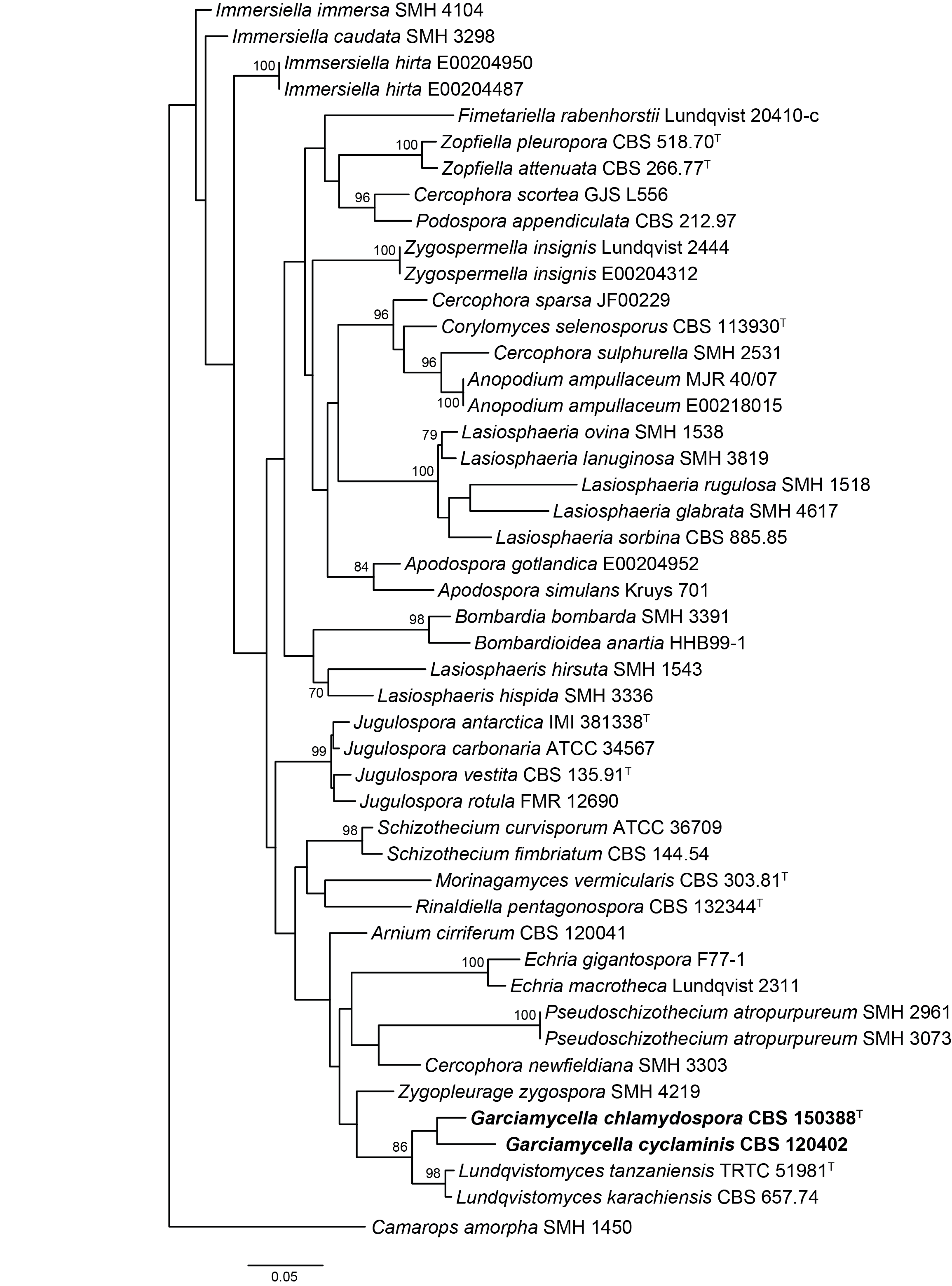


Figure S59. RAxML phylogram obtained from the fragment of β-tubulin (tub2) sequences

Table S1. Alignment used in the phylogenetic study.

>*Ramophialophora_globispora*_LC6218

????????????????????????CTCTCT-AAA-CA-TTGTGA-CG--ACGAAC----CCG-TTGCTTCGGCGTTGTTTCTCTGAGTA------CTTATTCAATAAGTAAAAACTTTCAACAACGGATCTCTTGGTTCTGGCATCGATGAAGAACGCAGCGAAATGCGATAAGTAATGTGAATTGCAGAATTCAGTGAATCATCGAATCTTTGAACGCACATTGCGCCCGCCAGTATTCTGGCGGGCATGCCTGTTCGAGCGTCATTTCAA---CCATCAAG-CCCCA---GGCTTGGTGTTGGGG-GCCTGCA-GCT-GCT--GCAGCCCCC-TAAAAGCAGTGGCGGACTCGCT-GTCAT-TCCGAGCGCAGTAGTT-ACATCTCGCTCTGGGCGTGGCGGCGGG-CACCTGCCGTGAAACACTCTTT--------CTAAGGTTGACCTCGGATCAGGTAGGAATACCCGCTGAACTTAAGCATATCAATAAGCGGAGGAAAAGAAACCAACAGGG-ATTGCCC-TAGTAACGGCG-AGTGAAGCGGCAACAGCTCAAATTTGAAATCTGGCTTC--GG-CCCGAGTTGTAATTTGTAGAGG-AAGCTTCTGGCGCGGTGCCATCCGAGTCCCCTGGAACGGGGCGCCATAGAGGGTGAGAGCCCCGTATGGATGGATGCCTA-GCCTGTGTGAAGCTCCTTCGACGAGTCGAGTAGTTTGGGAATGCTGCTCAAAATGGGAGGTAAATTCCTTCTAAAGCTAAATATTGGCCAGAGACCGATAGCGCACAAGTAGAGTGATCGAAAGATGAAAAGCACTTTGAAAAGAGGGTTAAACAGCACGTGAAATTGTTGAAAGGGAAGCGCTTGTGACCAGACTTGCGCCAGGCTGATCATCCGGTGTTCTCACCGGTGCACTCTGCCTGGCACAGGCCAGCATCAGTTTTGGTGGGGGGATAAAGGGCGCAGGAACGTAGCTCC--TCCGGGAGTG-TTATAGCCTGTGTCGTAATGCCCTCGCCGGGACTGAGGACCGCGCATCTGCAAGGATGCTGGCGTAATGGTCATCAGCGACCCGTCTTGAAACACGGACCAAGGAGTCAAGGTTTTGCGCGAGTGTTTGGGTGTAAAACCCGCACGCGTAATGAAAGTGAACGTAGGTGAGAG--CTTCGGCGCATCATCGACCGATCCTGATGTATTCGGATGGATTTGAGTAAGAGCGTTAAGCCTTGGACCCGAAAGATGGTGAACTATGCTTGGATAGGGTGAAGCCAGAGGAAACTCTGGTGGAGGCTCGCAGCGGTT-CTGACGTGCAAATCGATCGTCAAATCTGAGCAT-GGGGGCGAAAGACTAATCGAACCATCTAGTA?????????????GGTCTCAAATACTCGCTGGCGACGGGCAATTGGGGTGATCAAAAGAAGGCGATGAGCTCGACGGCCGGTGTGTCCCAGGTGTTGAACCGCTACACCTTTGCGTCGACGCTGTCCCATTTGCGTCGTACCAACACTCCAATCGGCCGCGATGGCAAGCTTGCGAAGCCTCGTCAGCTCCACAACACTCATTGGGGCTTGGTTTGCCCTGCCGAAACACCCGAGGGTCAGGCTTGCGGTCTCGTCAAGAACTTGTCGTTGATGTGCTACATCAGCGTTGGTACCAATGCGGAGCCGATTGTTGACTTTATGATAGCAAGAAACATGGAAGTTCTGGAGGAGTACGAACCACTTCGATATCCCAACGCCACCAAGGTCTTTGTGAATGGCACTTGGGTCGGTGTTGTTCAGGACGCCAAGAGCCTGGTTGGTCTTGTCCAGGGCCTGCGACGGTCGAATATTATTTCGTTCGAGGTTTCGCTGGTCAGAGATATCCGAGACAGAGAGTTCAAGATCTTTTCGGATGCCGGTCGTGTCATGCGACCCCTTTTTGTTGTGGAGCAAGAAGACGGCAACACA------GCGAGCGGCGTCGAGCGAGGACAGCTGGTTTTGAGGAAGGAGCATTGCAATAAGTTGGAGCGCGATCGAGAGATTGGTAGATTCG------------ACCCCGATTACTACGGCTGGGACGGCCTGCTCCGGGACGGATGTGTCGAGTATCTCGATGCGGAGGAAGAAGAGACAACCATGATCTGCATGTCTCCCGAAGATCTCGAGATCTTCAGAACGACAAAGAT------CTTGGGTATCCCCCAGGTCGATGTCGAGGACCCTAGTGCGGGTAACAAGCGCATCCCGACAAAACTCAACCCAACAACCCACGCGTACACGCACTGCGAAATTCACCCGAGTATGCTGCTTGGCATCTGCGCAAGCATCATTCCTTCC?????????????????????????????????????????????????????????????????????????????????????????????????????????????????????????????????????????????????????????????????????????????????????????????????????????????????????????????????????????????????????????????????????????????????????????????????????????????????????????????????????????????????????????????????????????????????????????????????????????????????????????????????????????????????????????????????????????????????????????????????????????????????????????????????????????????????????????????????????????????????????????????????????????????????????????????????????????????????????????

>*Garciamycella_cyclaminis*_CBS_166.42

ATTAGCGA----------GTATAACTCTCTAAAACCA-TTGTGAACG-AACAGAC----CGG-TTGCTTCGGCGTTGTCTCTCTGAGTAA-----ATTACTTAATAAGTTAAAACTTTCAACAACGGATCTCTTGGTTCTGGCATCGATGAAGAACGCAGCGAAATGCGATAAGTAATGTGAATTGCAGAATTCAGTGAATCATCGAATCTTTGAACGCACATTGCGCCCGCCAGTATTCTGGCGGGCATGCCTGTTCGAGCGTCATTTCAA---CCATCAAG-CCCCA----GGCTTGTGTTGGGG-GCCTGCA-GCT-GCT--GCAGCCCCC-TAAAAGCAGTGGCGGACTCGCT-GTCAT-TCCGAGCGCAGTAGTT-ACATCTCGCTCTGGACGTGGCGGCGGG-CACCTGCCGTGAAACACTCTTT--------CTAAGGTTGACCTCGGAT??????????????????????????GCATATCAATAAGCGGAGGAAAAGAAACCAACAGGG-ATTGCCCCCAGTAACGGCG-AGTGAAGCGGCAACAGCTCAAATTTGAAATCTGGCTTC--GG-CCCGAGTTGTAATTTGTAGAGG-AAGCTTCTGGCGCGGTGCCATCCGAGTCCCCTGGAACGGGGCGCCATAGAGGGTGAGAGCCCCGTATGGATGGATGCCTA-GCCTGTGTGAAGCTCCTTCGACGAGTCGAGTAGTTTGGGAATGCTGCTCAAAATGGGAGGTAAATTCCTTCTAAAGCTAAATATTGGCCAGAGACCGATAGCGCACAAGTAGAGTGATCGAAAGATGAAAAGCACTTTGAAAAGAGGGTTAAACAGCACGTGAAATTGTTGAAAGGGAAGCGCTTGTGACCAGACTTGCGCCAGGCTGATCATCCGGTGTTCTCACCGGTGCACTCTGCCTGGCACAGGCCAGCATCAGTTTTGGTGGGGGGATAAAGGGCGCAGGAACGTAGCTCC--TCCGGGAGTG-TTATAGCCTGTGTCGTAATGCCCTCGCCGGGACTGAGGACCGCGCATCTGCAAGGATGCTGGCGTAATGGTTATCAGCGACCCGTCTTGAAACACGGACCAAGGAGTCAAGGTTTTGCGCGAGTGTTTGGGTGTAAAACCCGCACGCGTAATGAAAGTGAACGTAGGTGAGAG--CTTCGGCGCATCATCGACCGATCCTGATGTATTCGGATGGATTTGAGTAAGAGCGTTAAGCCTTGGACCCGAAAGATGGTGAACTATGCTTGGATAGGGTGAAGCCAGAGGAAACTCTGGTGGAGGCTCGCAGCGGTT-CTGACGTGCAAATCGATCGTCAAATCTGAGCAT-GGGGGCGAAAGACTAATCGAACCATCTAGTAGCTGGTTACCGCC?????????????????????????????????????????????????????????????????????????????????????????????????????????????????????????????????????????????GGTCGTGATGGCAAACTCGCAAAGCCTCGTCAGCTCCACAACACCCATTGGGGCTTGGTTTGCCCTGCCGAAACACCCGAGGGGCAGGCTTGCGGTCTCGTCAAGAACCTGTCCTTGATGTGTTACATCAGTGTCGGTACCAACGCGGAGCCGATCGTTGACTTTATGATTGCAAGAAACATGGAGGTGCTGGAAGAATACGAGCCGCTCCGGTATCCCAACGCCACCAAGGTCTTTGTGAATGGTACCTGGGTCGGTGTTGTTCAGGACGCCAAAAGTCTGGTTGGTCTTGTCCAGGGGCTGAGACGATCAAATATTATTTCCTTCGAGGTGTCCCTGGTCAGAGATATCCGAGACCGCGAGTTCAAGATCTTTTCAGATGCCGGTCGCGTCATGAGGCCTCTCTTCGTTGTCGAGCAAGAAGATGGCAACCCG------AACACCAAGGTTGAGCGAGGACAGCTGGTTTTGACCAAGGAACACACGGCGAAGTTGGAGCACGACAGGGAGATTGGGAGGTTTG------------ACCCCAATTATTACGGATGGGATGGCCTGCTCCGGGACGGATGTGTCGAGTATCTCGATGCAGAGGAAGAGGAGACAACCATGATCTGCATGTCACCTGAAGATCTCGAACACTTCCGAACGAC?????????????????????????????????????????????????????????????????????????????????????????????????????????????????????????????????????????????????????????????????????????????????????????????????????????????????????????????????????????????????????????????????????????????????????????????????????????????????????????????????????????????????????????????????????????????????????????????????????????????????????????????????????????????????????????????????????????????????????????????????????????????????????????????????????????????????????????????????????????????????????????????????????????????????????????????????????????????????????????????????????????????????????????????????????????????????????????????????????????????????????????????????????????????????????????????????????????????????????????

>*Garciamycella_chlamydospora*_CBS_150388

attagcga----------gtataactctctaaaacca-ttgtgaacg-aacgaac----ccg-ttgcttcggcgttgtttctctgagta------cttattcaataagtaaaaactttcaacaacggatctcttggttctggcatcgatgaagaacgcagcgaaatgcgataagtaatgtgaattgcagaattcagtgaatcatcgaatctttgaacgcacattgcgcccgccagtattctggcgggcatgcctgttcgagcgtcatttcaa---ccatcaag-cccca----ggcttgtgttgggg-gcctgca-gct-gct--gcagccccc-taaaagcagtggcggactcgct-gtcat-tccgagcgcagtagtt-acatctcgctctgggcgtggcggtggg-cacctgccgtgaaacactcttt--------ctaaggttgacctcggatcaggtaggaatacccgctgaacttaagcatatcaataagcggaggaaaagaaaccaacaggg-attgccc-tagtaacggcg-agtgaagcggcaacagctcaaatttgaaatctggcttc--gg-cccgagttgtaatttgtagagg-aagcttctggcgcggtgccatccgagtcccctggaacggggcgccatagagggtgagagccccgtatggatggatgccta-gcctgtgtgaagctccttcgacgagtcgagtagtttgggaatgctgctcaaaatgggaggtaaattccttctaaagctaaatattggccagagaccgatagcgcacaagtagagtgatcgaaagatgaaaagcactttgaaaagagggttaaacagcacgtgaaattgttgaaagggaagcgcttgtgaccagacttgcgccaggctgatcatccggtgttctcaccggtgcactctgcctggcacaggccagcatcagttttggtggggggataaagggcgcaggaacgtagctcc--tccgggagtg-ttatagcctgtgtcgtaatgccctcgccgggactgaggaccgcgcatctgcaaggatgctggcgtaatggtcatcagcgacccgtcttgaaacacggaccaaggagtcaaggttttgcgcgagtgtttgggtgtaaaacccgcacgcgtaatgaaagtgaacgtaggtgagag--cttcggcgcatcatcgaccgatcctgatgtattcggatggatttgagtaagagcgttaagccttggacccgaaagatggtgaactatgcttggatagggtgaagccagaggaaactctggtggaggctcgcagcggtt-ctgacgtgcaaatcgatcgtcaaatctgagcat-gggggcgaaagactaatcgaaccatctagtagctggttaccgcc?????????????????????????????????????????????????????????????????????????????????????????????????ttgcgtcgacactgtcccatttgcgtcgtaccaacactccgatcggccgcgatggcaagcttgcgaagccccgtcagctccacaacactcattggggcttggtttgccctgccgaaacgcccgagggtcaggcttgcggtctcgtcaagaacttgtcgttgatgtgctacatcagcgttggcaccaatgcggagccgattgttgactttatgatagcaaggaacatggaagttctggaggagtacgaaccgcttcgatatcccaacgccaccaaggtctttgtgaatggcacttgggtcggtgttgttcaggacgccaaaagcctggttggtcttgtccagggcctgcgacggtcgaatattatttcgttcgaggtttcactggtcagagatatccgagaccgagagttcaagatcttttcggatgccggtcgtgtcatgcgacccctgtttgttgtggagcaagaagacggcaacaca------acgagcggcgtcgagcgaggacagttggttttgaagaaggagcactgcaataagttggagcgcgatcgagagattggtagattcg------------accccgattactacggctgggacggcctgctccgggacggatgtgtcgagtatctcgatgcggaggaagaagagacaaccatgatctgcatgtctcccgaagatctcgagatcttcagaacgacgaagat------cttgggcattccccaggtcgatgtcgaggaccctagtgcgggtaacaagcgcatcccgacaaaactcaatccaactacccacgcgtacacgcactgcgaaattcacccgagcatgctgctcggcatctgcgcaagcatcattcctttccctgatcacaaccaagcttgttgaccaagttctcgatgtcgtccgtcgtgaggctgagggctgcgactgcctccagggcttccagatcacccactctctcggtggtggtaccggtgccggtatgggtaccctcctcatctccaagatccgcgaggagttccccgaccgcatgatggcgactttctccgtcgtcccctcccctaaggtgtcggataccgttgtcgagccctacaacgccaccctctccgtccaccagcttgttgagaactcggacgagaccttctgcatcgacaacgaggctctctacgacatctgcatgaggacactcaagctctccaacccctcatacggcgaccttaaccacctcgtttcggccgtcatgtccggtgttaccgtctcgctccgtttccccggtcagctcaactcggatctccgcaagctcgccgtgaacatggtgcccttcccccgtctgcacttcttcatggtcggctttgcccctcttactagccgcggcgcccactctttccgtgctgtctcggttcccgagctcacccagcagatgttcgaccccaagaacatgatggctgcctccgacttccgcaacggtcgttacctcacttgctccgccatctt

>*Garciamycella_cyclaminis*_CBS_120402

ATTAGCGA----------GTATAACTCTCTAAAACCA-TTGTGAACG-AACAGAC----CGG-TTGCTTCGGCGTTGTCTCTCTGAGTAA-----ATTACTTAATAAGTTAAAACTTTCAACAACGGATCTCTTGGTTCTGGCATCGATGAAGAACGCAGCGAAATGCGATAAGTAATGTGAATTGCAGAATTCAGTGAATCATCGAATCTTTGAACGCACATTGCGCCCGCCAGTATTCTGGCGGGCATGCCTGTTCGAGCGTCATTTCAA---CCATCAAG-CCCCA----GGCTTGTGTTGGGG-GCCTGCA-GCT-GCT--GCAGCCCCC-TAAAAGCAGTGGCGGACTCGCT-GTCAT-TCCGAGCGCAGTAGTT-ACATCTCGCTCTGGACGTGGCGGCGGG-CACCTGCCGTGAAACACTCTTT--------CTAAGGTTGACCTCGGATCAGGTAGGAATACCCGCTGAACTTAAGCATATCAATAAGCGGAGGAAAAGAAACCAACAGGG-ATTGCCC-CAGTAACGGCG-AGTGAAGCGGCAACAGCTCAAATTTGAAATCTGGCTTC--GG-CCCGAGTTGTAATTTGTAGAGG-AAGCTTCTGGCGCGGTGCCATCCGAGTCCCCTGGAACGGGGCGCCATAGAGGGTGAGAGCCCCGTATGGATGGATGCCTA-GCCTGTGTGAAGCTCCTTCGACGAGTCGAGTAGTTTGGGAATGCTGCTCAAAATGGGAGGTAAATTCCTTCTAAAGCTAAATATTGGCCAGAGACCGATAGCGCACAAGTAGAGTGATCGAAAGATGAAAAGCACTTTGAAAAGAGGGTTAAACAGCACGTGAAATTGTTGAAAGGGAAGCGCTTGTGACCAGACTTGCGCCAGGCTGATCATCCGGTGTTCTCACCGGTGCACTCTGCCTGGCACAGGCCAGCATCAGTTTTGGTGGGGGGATAAAGGGCGCAGGAACGTAGCTCC--TCCGGGAGTG-TTATAGCCTGTGTCGTAATGCCCTCGCCGGGACTGAGGACCGCGCATCTGCAAGGATGCTGGCGTAATGGTTATCAGCGACCCGTCTTGAAACACGGACCAAGGAGTCAAGGTTTTGCGCGAGTGTTTGGGTGTAAAACCCGCACGCGTAATGAAAGTGAACGTAGGTGAGAG--CTTCGGCGCATCATCGACCGATCCTGATGTATTCGGATGGATTTGAGTAAGAGCGTTAAGCCTTGGACCCGAAAGATGGTGAACTATGCTTGGATAGGGTGAAGCCAGAGGAAACTCTGGTGGAGGCTCGCAGCGGTT-CTGACGTGCAAATCGATCGTCAAATCTGAGCAT-GGGGGCGAAAGACTAATCGAACCATCTAGTAGCTGGTTACCGCC???CTCAAGTACTCTTTGGCGACRGGCAATTGGGGCGACCAGAAGAAGGCAATGAGTTCGACGGCTGGTGTGTCGCAGGTGTTGAACAGATACACCTTCGCGTCTACCCTGTCCCATTTGCGTCGCACCAACACCCCGATCGGTCGTGATGGCAAACTCGCAAAGCCTCGTCAGCTCCACAACACCSATTGGGGCTTGGTTTGCCCTGCCGAAACACCCGAGGGGCAGGCTTGCGGTCTCGTCAAGAACCTGTCCTTGATGTGTTACATCAGTGTCGGTACCAACGCGGAGCCGATCGTTGACTTTATGATTGCAAGAAACATGGAGGTGCTGGAAGAATACGAGCCGCTCCGGTATCCCAACGCCACCAAGGTCTTTGTGAATGGTACCTGGGTCGGTGTTGTTCAGGACGCCAAAAGTCTGGTTGGTCTTGTCCAGGGGCTGAGACGATCAAATATTATTTCCTTCGAGGTGTCCCTGGTCAGAGATATCCGAGACCGCGAGTTCAAGATCTTTTCAGATGCCGGTCGCGTCATGAGGCCTCTCTTCGTTGTCGAGCAAGAAGATGGCAACCCG------AACACCAAGGTTGAGCGAGGACAGCTGGTGTTGACGAAGGACCACACGGCGAAGTTGGAGCACGACAGGGAGATTGGGAGGTTTG------------ACCCCAATTATTACGGATGGGATGGCCTGCTCCGGGACGGATGTGTCGAGTATCTCGATGCAGAGGAAGAGGAGACAACCATGATCTGCATGTCGCCTGAAGATCTCGAACACTTCCGAACGACAAAGAT?????????????????????????????????????????????????????????????????????????????????????????????????????????????????????????????????????????????????????????????????????????GCTTGTTGACCAAGTTCTCGATGTTGTTCGTCGCGAGGCTGAGGGCTGCGACTGCCTTCAGGGCTTCCAGATCACCCACTCTCTCGGTGGTGGTACTGGTGCCGGTATGGGTACCCTCCTTATCTCCAAGATTCGTGAGGAGTTCCCCGACCGCATGATGGCGACCTTCTCCGTCGTCCCCTCTCCCAAGGTCTCGGATACCGTTGTCGAGCCCTACAACGCCACCCTCTCCGTCCACCAGCTTGTTGAGAACTCCGACGAGACCTTCTGCATTGACAACGAGGCTCTCTACGACATCTGCATGAGGACACTCAAGCTCTCCAACCCCTCGTACGGCGATCTTAACCACCTCGTTTCCGCCGTCATGTCCGGTGTTACCGTCTCGCTCCGTTTCCCCGGCCAGCTCAACTCGGATCTCCGCAAGCTTGCCGTGAACATGGTGCCTTTCCCCCGTCTGCATTTCTTCATGGTCGGCTTTGCGCCCCTTACCAGCCGCGGCGCCCACTCTTTCCGTGCCGTCTCGGTTCCCGAGCTCACGCAGCAGATGTTCGACCCCAAGAACATGATGGCTGCTTCCGACTTCCGTAACGGTCGCTACCTCACTTGCTCCGCCATCTT

>*Lasiosphaeris_arenicola*_ANM_1080

ATTACCGAGTT------GCCAAGACTCCC--CCACCA-TTGTGAACCCTACCCCG----CAG-TTGCTTCGGCGAGGCCTCTCTGAGTAC---CAAAACAAAAATAAGTCAAAACTTTCAACAACGGATCTCTTGGCTCTGGCATCGATGAAGAACGCAGCGAAATGCGATAAGTAATGTGAATTGCAGAATTCAGTGAATCATCGAATCTTTGAACGCACATTGCGCCCGCCAGCACTCTGGCGGGCATGCCTGTCCGAGCGTCATTTCCA---CCATCAAG-CGCTC---CCGCTTGCGTTGGGGCCTCTGCG-GCT-GCC--GCAGTCCCC-GAAAATCAGTGGCGGGCTCGCT-GTCATCACCGAGTGCAGTAGTT--CTCTATCCTCTCCGCGATGCAGCGC--TCCTTGCCGTAAAACACCCCCA----CCTCTCTAGGTTGACCTCGGATCAGGTAGGAATACCCGCTGAACTTAAGCATATCAATAAGCGGAGGAAAAGAAACCAACAGGG-ATTGCCT-CAGTAACGGCG-AGTGAAGCGGCAACAGCTCAAATTTGAAATCTGGCCTC--GG-CCCGAGTTGTACTTTGCAGAGG-GCGATTCTGGTGAGGCACCTGCTGAGTCCCCTGGAACGGGGCGCCGTAGAGGGTGAGAGCCCCGTAGCGCAGGATGCCGA-CCCGCTGTGAATCCCCTTCGACGAGTCGAGTAGTTTGGGAATGCTGCTCAAAATGGGAGGTAAATTCCTTCTAAAGCTAAATACCGGCCAGAGACCGATAGCGCACAAGTAGAGTGATCGAAAGATGAAAAGCACTTTGAAAAGAGGGTTAAACAGCACGTGAAATTGTTGAAAGGGAAGCGCTCACGACCAGACTTGCGCCGGGGCGATCATCCGCTCTTCTGAGCGGTGCACTCGCCCCGGGGCAGGCCAGCATCGGTTCCGGCGGGGGGACAAAAGCCCAGGGAATGTGGCTCGCCTCGGCGAGTG-TTATAGCCCTGTCCACAATGCCCCCGCGGGGACCGAGGTTCGCGCGTCTGCAAGGATGCTGGCGTAATGGTCATCAGCGACCCGTCTTGAAACACGGACCAAGGAGTCAAGGTTTGGCGCGAGTGTCTGGGTGTCAAACCCGCACGCGTAATGAAAGTGAACGCAGGTGAGAG--CTTCGGCGCATCATCGACCGATCCTGATGTCCTCGGATGGATTTGAGTAGGAGCGTCAAGCCTTGGACCCGAAAGATGGTGAACTATGCTTGGATAGGGTGAAGCCAGAGGAAACTCTGGTGGAGGCTCGCAGCGGTT-CTGACGTGCAAATCGATCGTCAAATCTGAGCAT-GGGGGCGAAAGACTAATCGAACCATCTAGTAGCTGGTTACCGCC????????????????????????????????????????????????????????????????????????????????????????????????????????????????????????????????????????????????????????????????????????????????????????????????????????????????????????????????????????????????????????????????????????????????????????????????????????????????????????????????????????????????????????????????????????????????????????????????????????????????????????????????????????????????????????????????????????????????????????????????????????????????????????????????????????????????????????????????????????????????????????????????????????????????????????????????????????????????????????????????????????????????????????????????????????????????????????????????????????????????????????????????????????????????????????????????????????????????????????????????????????????????????????????????????????????????????????????????????????????????????????????????????????????????????????????????????????????????????????????????????????????????????????????????????????????????????????????????????????????????????????????????????????????????????????????????????????????????????????????????????????????????????????????????????????????????????????????????????????????????????????????????????????????????????????????????????????????????????????????????????????????????????????????????????????????????????????????????????????????????????????????????????????????????????????????????????????????????????????????????????????????????????????????????????????????????????????????????????????????????????????????????????????????????????????????????????????????????????????????

>*Lasiosphaeris_hirsuta*_SMH1543

???????????????????????????????????????????????????????????????????????????????????????????????????????????????????????????????????????????????????????????????????????????????????????????????????????????????????????????????????????????????????????????????????????????????????????????????????????????????????????????????????????????????????????????????????????????????????????????????????????????????????????????????????????????????????????????????????????????????????????????????????????????????????????AAAGAAACCAACAGGG-ATTGCCT-CAGTAACGGCG-AGTGAAGCGGCAACAGCTCAAATTTGAAATCCGGCCTC--GG-CCCGAGTTGTAATTTGCAGAGG-AAGATTCCGGTGAGGTACCTGCTGAGTCTCCTGGAACGGAGCGCCGCAGAGGGTGAGAGCCCCGTATAGCAGGACGCCAA-GCCTTTGTGAATCCCCTTCGACGAGTCGAGTAGTTTGGGAATGCTGCTCAAAATGGGAGGTAAATTCCTTCTAAAGCTAAATACTGGCCAGAGACCGATAGCGCACAAGTAGAGTGATCGAAAGATGAAAAGCACTTTGAAAAGAGGGTTAAACAGCACGTGAAATTGTTGAAAGGGAAGCGCTCGTGACCAGACTTGCGCCGGAGCGATCATCCGCTCTTCTGAGCGGTGCACTCGCCCCGGGGCAGGCCAGCATCGGTTCCGGCGGGGGGACAACAGTTCAGGGAATGTGGCTCGCCTCGGCGAGTG-TTATAGCCCGGTTCACAATGCCCCCGCGGGGACCGAGGTTCGCGCGTCTGCAAGGATGCTGGCGTAATGGTCACCAGCGACCCGTCTTGAAACACGGACCAAGGAGTCAAGGTTTCGCGCGAGTGTCTGGGTGTCAAACCCGCACGCGTAATGAAAGTGAACGTAGGTGAGAG--CTTCGGCGCATCATCGACCGATCCTGATGTCTTCGGATGGATTTGAGTAGGAGCGTCAAGCCTTGGACCCGAAAGATGGTGAACTATGCTTGGATAGGGTGAAGCCAGAGGAAACTCTGGTGGAGGCTCGCAGCGGTT-CTGACGTGCAAATCGATCGTCAAATCTGAGCAT-GGGGGCGAAAGACTAATCGAACCATCTAGTAGCTGGTTACCGCCGGCCTCAAGTACTCGTTGGCAACAGGAAACTGGGGTGATCAGAAGAAGGCTGCGAGCTCCACAGCTGGCGTCTCTCAGGTGTTGAACAGGTACACATTTGCCTCAACCCTTTCGCATTTGCGACGAACCAACACACCTATTGGTCGTGATGGCAAACTTGCCAAACCCCGCCAGCTGCACAACACCCATTGGGGCTTGGTCTGTCCCGCAGAAACGCCAGAAGGACAGGCTTGTGGTTTGGTCAAGAATTTGTCTCTCATGTGCTTTGTGAGTGTGGGCTCTGCGCAGGAACCCATTATAGAATTTATGATCGCTCGAGGCATGGAGGTTCTTGAAGAATACGAGCCCCTGCGGTATCCGAACGCCACCAAGGTTTTCGTCAACGGCACTTGGGTCGGTGTCCACCAGCAACCAAAGGAGCTGGTGACCGACATCCAGGGTCTCCGGAGAAAGGGATCTCTGACAGCTGAAGTCTCGCTTGTTAGAGACATTCGAGACAGAGAGTTTAAGATTTTCACAGACGCCGGCCGTGTCATGAGACCGCTATTTGTCGTTGAGCAGAGCGAAAGCGGTGAA---------TGGGGAGTCGAGAAGGGCCAGCTGATCATACAAAAACGGCACATTGAGAGATTGCAGGAAGACAAGGCCTATGCTGGTATCCCGACTCTCGAGGGAGAGGAAAAGCGAGGCTGGGAGTTTCTGCTGGCAGACGGCTGCATCGAGTATCTCGATGCCGAGGAGGAAGAAACGGCCATGATTTGCATGACTCCCGAAGACTTGGACACGTACCGGCTCAGCAGATT------------GGGTTTCGACGTCCGCGACGAGGAAACGGCCGAAAACAACAAACGTATCAAGACCAAGATGAACCCAACAACGCACATGTATACGCACTGCGAGATTCACCCTAGCATGCTGCTCGGGATCTGCGCCAGCATTATCCCATTCCCCGACCATAACCAAGCTTGTTGACCAAGTTCTCGATGTTGTCCGTCGTGAGGCCGAGGGCTGTGACTGCCTCCAGGGCTTCCAGATTACCCACTCTCTCGGTGGTGGTACCGGTGCCGGTATGGGTACCCTCCTTATCTCCAAGATTCGTGAGGAGTTCCCCGACCGCATGATGGCCACTTTCTCCGTTGTGCCTTCGCCCAAGGTCTCGGATACTGTTGTTGAGCCCTACAACGCCACCCTCTCGGTCCATCAGCTTGTTGAGAACTCCGACGAGACCTTTTGCATCGACAACGAAGCTCTCTACGACATCTGCATGCGCACACTGAAGCTGTCGAATCCCTCATATGGTGATCTCAACCACTTGGTCTCGGCCGTCATGTCCGGTGTCACCGTCTCGCTGCGTTTCCCCGGCCAGCTGAACTCTGATCTCCGCAAGCTTGCTGTCAACATGGTTCCGTTCCCGCGTCTCCATTTCTTCATGGTTGGCTTCGCGCCTCTTACTAGCCGTGGCGCGCACTCTTTCCGTGCCGTCTCGGTTCCAGAGTTGACTCAGCAGATGTTCGACCCCAAGAATATGATGGCTGCTTCCGACTTCCGCAACGGTCGCTACCTGACCTGCTCTGCCATCTT

>*Lasiosphaeris_hispida*_CBS_955.72

ATTACAGAGTT-------GCAAAACTCCC----ACCA-TTGTGAATC-TACCTA-----TAG-TTGCTTCGGCGAGGCCTCTCTGAGTAA------AACAAAAATAAGTTAAAACTTTCAACAACGGATCTCTTGGTTCTGGCATCGATGAAGAACGCAGCGAAATGCGATAAGTAATGTGAATTGCAGAATTCAGTGAATCATCGAATCTTTGAACGCACATTGCGCCCGCTAGTATTCTGGCGGGCATGCCTGTCCGAGCGTCATTTCAA---CCATCAAG-CCCTA----GGCTTGTGTTGGGG-TTCTGCG-GCT-GTC-CGCAGGCCCT-GAAAATCAGTGGCGGGCTCGTT-GTCAA-ACCGAGTGCAGTAGTA--ATATTCTCTCTGGTCTT-ACAGCTTG-TTCTTGCCGTAAAACAATCCAA-----TTTCTAAGGTTGACCTCGGATCAGGTAGGAATACCCGCTGAACTTAAGCATATCAATAAGCGGAGGAAAAGAAACCAACAGGG-ATTGCCCCTAGTAACGGCG-AGTGAAGCGGCAACAGCTCAAATTTGAAATCTGGCTTC--GG-CCCGAGTTGTAATTTGTAGAGG-AAGATTCTGGCAAGGCACCTACTGAGTCCCCTGGAACGGGGCGCCATAGAGGGTGAGAGCCCCGTATAGTAGGACGCTTA-GCCTGTGTGAATCTCCTTCGACGAGTCGAGTAGTTTGGGAATGCTGCTCAAAATGGGAGGTAAATTCCTTCTAAAGCTAAATACTGGCCAGAGACCGATAGCGCACAAGTAGAGTGATCGAAAGATGAAAAGCACTTTGAAAAGAGGGTTAAACAGCACGTGAAATTGTTGAAAGGGAAGCGCTTATGACCAGACTTGCGCCGTGGCGATCATCCGCTGTTTTCAGTGGTGCACTCGCTGCGGCACAGGCCAGCATCGGTTCTCGTGGGGGGATAAAGGTCGCGGGAATGTGGCTCGTCTCGGCGAGTG-TTATAGCCCGTGGCGTAATGCCCCCGTGGGGACCGAGGTTCGCGCATCTGCAAGGATGCTGGCGTAATGGTCATCAGCGACCCGTCTTGAAACACGGACCAAGGAGTCAAGGTTTTGCGCGAGTGTTTGGGTGTTAAACCCGCACGCGTAATGAAAGTGAACGTAGGTGAGAG--CTTCGGCGCATCATCGACCGATCCTGATGTATTCGGATGGATTTGAGTAGGAGCGTTAAGCCTTGGACCCGAAAGATGGTGAACTATGCTTGGATAGGGTGAAGCCAGAGGAAACTCTGGTGGAGGCTCGCAGCGGTT-CTGACGTGCAAATCGATCGTCAAATCTGAGCATGGGGGGCGAAAGACTAATCGAACCATCTAGTAGCTGGTTACCGCC????????????????????????????????????????????????????????????????????????????????????????????????????????????????????????????????????????????????????????????????????????????????????????????????????????????????????????????????????????????????????????????????????????????????????????????????????????????????????????????????????????????????????????????????????????????????????????????????????????????????????????????????????????????????????????????????????????????????????????????????????????????????????????????????????????????????????????????????????????????????????????????????????????????????????????????????????????????????????????????????????????????????????????????????????????????????????????????????????????????????????????????????????????????????????????????????????????????????????????????????????????????????????????????????????????????????????????????????????????????????????????????????????????????????????????????????????????????????????????????????????????????????????????????????????????????????????????????????????????????????????????????????????????????????????????????????????????????????????????????????????????????????????????????????????????????????????????????????????????????????????????????????????????????????????????????????????????????????????????????????????????????????????????????????????????????????????????????????????????????????????????????????????????????????????????????????????????????????????????????????????????????????????????????????????????????????????????????????????????????????????????????????????????????????????????????????????????????????????????????

>*Lasiosphaeris_hispida*_SMH3336

???????????????????????????????????????????????????????????????????????????????????????????????????????????????????????????????????????????????????????????????????????????????????????????????????????????????????????????????????????????????????????????????????????????????????????????????????????????????????????????????????????????????????????????????????????????????????????????????????????????????????????????????????????????????????????????????????????????????????????????????????????????????????????AAAGAAACCAACAGGG-ATTGCCC-TAGTAACGGCG-AGTGAAGCGGCAACAGCTCAAATTTGAAATCTGGCTTC--GG-CCCGAGTTGTAATTTGTAGAGG-AAGATTCTGGCAAGGCACCTACTGAGTCCCCTGGAACGGGGCGCCATAGAGGGTGAGAGCCCCGTATAGTAGGACGCTTA-GCCTGTGTGAATCTCCTTCGACGAGTCGAGTAGTTTGGGAATGCTGCTCAAAATGGGAGGTAAATTCCTTCTAAAGCTAAATACTGGCCAGAGACCGATAGCGCACAAGTAGAGTGATCGAAAGATGAAAAGCACTTTGAAAAGAGGGTTAAACAGCACGTGAAATTGTTGAAAGGGAAGCGCTTATGACCAGACTTGCGCCGTGGTGATCATCCGCTGTTTTCAGTGGTGCACTCGCTGCGGCACAGGCCAGCATCGGTTCTCGTGGGGGGATAAAGGTCGCGGGAATGTGGCTCGTCTCGGCGAGTG-TTATAGCCCGTGGCGTAATGCCCCCGTGGGGACCGAGGTTCGCGCATCTGCAAGGATGCTGGCGTAATGGTCATCAGCGACCCGTCTTGAAACACGGACCAAGGAGTCAAGGTTTTGCGCGAGTGTTTGGGTGTTAAACCCGCACGCGTAATGAAAGTGAACGTAGGTGAGAG--CTTCGGCGCATCATCGACCGATCCTGATGTATTCGGATGGATTTGAGTAGGAGCGTTAAGCCTTGGACCCGAAAGATGGTGAACTATGCTTGGATAGGGTGAAGCCAGAGGAAACTCTGGTGGAGGCTCGCAGCGGTT-CTGACGTGCAAATCGATCGTCAAATCTGAGCAT-GGGGGCGAAAGACTAATCGAACCATCTAGTAGCTGGTTACCGCCGGCCTCAAGTACTCGTTGGCAACCGGTAACTGGGGCGATCAGAAGAAGGCAGCAAGCTCCACGGCTGGTGTTTCCCAGGTGTTGAACAGATACACATTTGCCTCGACCCTTTCGCATTTGCGGCGAACCAATACACCTATTGGTCGTGATGGCAAGCTTGCTAAACCGCGTCAGCTTCACAATACCCATTGGGGCTTGGTTTGTCCCGCAGAAACGCCAGAAGGACAGGCTTGCGGTCTGGTCAAGAATTTGTCGCTCATGTGCTTTGTGAGTGTGGGCTCTGCGCAGGAACCCATCATAGAGTTTATGATTGCTAGGGGCATGGAAGTCCTCGAAGAATATGAGCCTCTGCGATATCCGAATGCCACCAAGGTTTTCGTCAACGGCACTTGGGTCGGTGTGCACCAACAACCCAAAGAGCTGGTCACCGACATCCAGGGTCTCCGGAGAAAGGGATCTCTTACGGCTGAAGTCTCGCTTGTTAGAGACATTCGAGACAGGGAGTTCAAGATTTTCACAGACGCCGGCCGTGTCATGAGACCACTATTTGTGGTTGAGCAGAGCGAAAATGGTGAA---------TGGGGCGTCGAGAAGGGCCAGCTGATCATACAGAAACGACACATCGAGAGGCTGCAGGAAGATAAGGCCTATGCTGGTATCCCGACTGTCGAGGGGGAGGAAAAGCGGGGTTGGGAGTTTTTGCTGGCAGACGGCTGCATCGAGTATCTCGACGCCGAGGAGGAAGAAACGGCCATGATTTGCATGACTCCAGAAGACCTGGACACGTACCGACTCAGCAGATT------------GGGTTTCGACGTCCGGGACGAGGAAACGGCGGAAAACAACAAACGTATCAAGACCAAGATGAACCCGACGACGCACATGTATACGCACTGCGAGATTCACCCCAGCATGCTGCTCGGCATCTGCGCCAGCATCATTCCTTTCCCCGACCACAACCAAGCTTGTTGACCAAGTTCTCGATGTGGTCCGCCGTGAGGCTGAGGGCTGCGACTGCCTCCAGGGCTTCCAGATCACCCACTCTCTCGGTGGTGGTACCGGTGCCGGTATGGGTACCCTCCTGATCTCCAAGATCCGCGAGGAGTTCCCCGACCGCATGATGGCCACCTTCTCCGTTGTGCCTTCGCCCAAGGTCTCGGATACCGTCGTTGAGCCGTACAACGCCACCCTCTCGGTCCATCAGCTTGTCGAGAACTCTGACGAGACCTTCTGCATTGATAACGAGGCTCTCTACGATATCTGCATGCGCACGCTTAAGCTGTCCAACCCCTCGTACGGCGATCTCAACCACCTGGTTTCGGCCGTCATGTCCGGTGTCACCGTCTCGCTGCGCTTCCCCGGCCAGCTAAACTCTGATCTCCGCAAGCTTGCCGTCAACATGGTTCCCTTCCCGCGTCTCCATTTCTTCATGGTTGGCTTCGCGCCTCTTACTAGCCGTGGCGCGCACTCTTTCCGTGCCGTCTCCGTCCCTGAGTTGACTCAGCAAATGTTCGACCCCAAGAACATGATGGCTGCTTCTGATTTCCGCAACGGCCGTTACCTGACTTGCTCTGCCATCTT

>*Lasiosphaeris_hirsuta*_JF02183

?????????????????????????????????????????????????????????????????????????????????????????????????????????????????????????????????????????????????????????????????????????????????????????????????????????????????????????????????????????????????????????????????????????????????????????????????????????????????????????????????????????????????????????????????????????????????????????????????????????????????????????????????????????????????????????????????????????????????????????????ACTTAAGCATATCAATAAGCGGAGGAAAAGAAACCAACAGGG-ATTGCCT-CAGTAACGGCG-AGTGAAGCGGCAACAGCTCAAATTTGAAATCCGGCCTC--GG-CCCGAGTTGTAATTTGCAGAGG-AAGATTCCGGCGAGGTACCTGCTGAGTCTCCTGGAACGGAGCGCCGCAGAGGGTGAGAGCCCCGTATAGCAGGACGCCAA-GCCTTTGTGAATCCCCTTCGACGAGTCGAGTAGTTTGGGAATGCTGCTCAAAATGGGAGGTAAATTCCTTCTAAAGCTAAATACTGGCCAGAGACCGATAGCGCACAAGTAGAGTGATCGAAAGATGAAAAGCACTTTGAAAAGAGGGTTAAACAGCACGTGAAATTGTTGAAAGGGAAGCGCTCGTGACCAGACTTGCGCCGGAGCGATCATCCGCTCTTCTGAGCGGTGCACTCGCCCCGGGGCAGGCCAGCATCGGTTCCGGCGGGGGGACAACAGTTCAGGGAATGTGGCTCGCCTCGGCGAGTG-TTATAGCCCGGTTCACAATGCCCCCGCGGGGACCGAGGTTCGCGCGTCTGCAAGGATGCTGGCGTAATGGTCACCAGCGACCCGTCTTGAACCACGGACCAAGGAGTCAAGGTTTCGCGCGAGTGTCTGGGTGTCAAACCCGCACGCGTAATGAAAGTGAACGTAGGTGAGAG--CTTCGGCGCATCATCGACCGATCCTGATGTCTTCGGATGGATTTGAGTAGGAGCGTCAAGCCTTGGACCCGAAAGATGGTGAACTATGCTTGGATAGGGTGAAGCCAGAGGAAACTCTGGTGGAGGCTCGCAGCGGTT-CTGACGTGCAAATCGATCGTCAAATCTGAGCAT-GGGGGCGAAAGACTAATCGAACCATCTAGTAGCTGGTTACCGCC????????????????????????????????????????????????????????????????????????????????????????????????????????????????????????????????????????????????????????????????????????????????????????????????????????????????????????????????????????????????????????????????????????????????????????????????????????????????????????????????????????????????????????????????????????????????????????????????????????????????????????????????????????????????????????????????????????????????????????????????????????????????????????????????????????????????????????????????????????????????????????????????????????????????????????????????????????????????????????????????????????????????????????????????????????????????????????????????????????????????????????????????????????????????????????????????????????????????????????????????????????????????????????????????????????????????????????????????????????????????????????????????????????????????????????????????????????????????????????????????????????????????????????????????????????????????????????????????????????????????????????????????????????????????????????????????????????????????????????????????????????????????????????????????????????????????????????????????????????????????????????????????????????????????????????????????????????????????????????????????????????????????????????????????????????????????????????????????????????????????????????????????????????????????????????????????????????????????????????????????????????????????????????????????????????????????????????????????????????????????????????????????????????????????????????????????????????????????????????????

>*Amesia_atrobrunnea*_CBS_379.66

ATTACAGAGTT-------GCAAAACTCCC-TAAACCA-TTGTGAACG-TTACCCTTAA-CCG-TTGCTTCGGCGTGGCCACTCTGAGTCT---TCTGTACTGAATAAGTCAAAACTTTCAACAACGGATCTCTTGGTTCTGGCATCGATGAAGAACGCAGCGAAATGCGATAAGTAATGTGAATTGCAGAATTCAGTGAATCATCGAATCTTTGAACGCACATTGCGCCCGCCAGTATTCTGGCGGGCATGCCTGTTCGAGCGTCATTTCAA---CCATCAAG-CCCCC----GGCTTGTGTTGGGG-ACCTGCG-GCT-GCC-CGCAGGCCCT-GAAAAACAGTGGCGGGCTCGCT-GTCAC-ACCGAGCGTAGTAGCATCATTCTCGCTCAGGGCGT-GCTGCGGG-TTCCGGCCGTTAAACGACCTTC---ATAACCCAAGGTTGACCTCGGATCAGGTAGGAAGACCCGCTGAACTTAAGCATATCAATAAGCGGAGGAAAAGAAACCAACAGGG-ATTGCCC-TAGTAACGGCG-AGTGAAGCGGCAACAGCTCAAATTTGAAATCTGGCTTC--GG-CCCGAGTTGTAATTTGCAGAGG-AAGCTTTAGGCGCGGCACCTTCTGAGTCCCCTGGAACGGGGCGCCATAGAGGGTGAGAGCCCCGTATAGTTGGATGCCTA-GCCTGTGTAAAGCTCCTTCGACGAGTCGAGTAGTTTGGGAATGCTGCTCAAAATGGGAGGTAAATTTCTTCTAAAGCTAAATACCGGCCAGAGACCGATAGCGCACAAGTAGAGTGATCGAAAGATGAAAAGCACTTTGAAAAGAGGGTTAAACAGCACGTGAAATTGTTGAAAGGGAAGCGCTTGTGACCAGACTTGCGCCAGGCTGATCATCCGGTGTTCTCACCGGTGCACTCGGCCCGGCACAGGCCAGCATCGGTTCTCGCGGGGGGATAAAGGTCTCGGGAACGTAGCTCC--TCCGGGAGTG-TTATAGCCCGGGGCGTAATGCCCTCGCGGGGACCGAGGTTCGCGCATCTGCAAGGATGCTGGCGTAATGGTCATCAGCGACCCGTCTTGAAACACGGACCAAGGAGTCAAGGTTTTGCGCGAGTGTTTGGGTGTCAAACCCGCACGCGTAATGAAAGTGAACGTAGGTGAGAG--CTTCGGCGCATCATCGACCGATCCTGATGTTTTCGGATGGATTTGAGTAGGAGCGTTAAGCCTTGGACCCGAAAGATGGTGAACTATGCTTGGATAGGGTGAAGCCAGAGGAAACTCTGGTGGAGGCTCGCAGCGGTT-CTGACGTGCAAATCGATCGTCAAATCTGAGCATGGGGGGCGAAAGACTAATCGAACCATCTAGTAGCTGGTTACCGCC????????????????????????????????????????????????????????????????????????????????????????????????????????????????????????????????????????????????????????????????????????????????????????????????????????????????????????????????????????????????????????????????????????????????????????????????????????????????????????????????????????????????????????????????????????????????????????????????????????????????????????????????????CTGGTCACACTGGTGCAGGGTTTGCGGAGGAAGAACGTCATTTCGTTCGAGGTTTCGCTGGTTCGCGACATCCGCGACCGCGAGTTCAAGATCTTCTCAGATGCCGGGCGCGTCATGAGGCCGCTGTTTACCGTCGAGCAGGAGCATGGTTCAGAG---------ACCGGCGCCGAGATGGGCCAGCTCATCCTCAACAAAGAGCATATTACACGGTTAGAAGCCGACAAGGAGCTGGGCAAGTACC------------ATCCCGACTACTGGGGCTGGCAGGGCCTGCTGAAGTCGGGTGCCATCGAGTATCTCGATGCCGAGGAGGAGGAGACAGCCATGATTTGCATGACGCCCGAGGATCTCGACAAGTTCCGGTACAGAAAGAT------------GGGGTTCATCGTCG--AAGACAACT-CGGGTCAAGGTAACAACAGGATCAAGACGAAGCCGAACCCGGCCACCCACATGTACACGCACTGCGAGATCCATCCCAGCATGCTGCTCGGCATCTGCGCAAGCATCATCCCCTTCCCCGACCACAACCAG??????????????????????????????????????????????????????????????????????????????????????????????????????????????????????????????????????????????????????????????????????????????????????????????????????????????????????????????????????????????????????????????????????????????????????????????????????????????????????????????????????????????????????????????????????????????????????????????????????????????????????????????????????????????????????????????????????????????????????????????????????????????????????????????????????????????????????????????????????????????????????????????????????????????????????????????????????????

>*Corynascus_sepedonium*_CBS_111.69

ATTACAGAGTT-------GCAAAACTCCC-TAAACCA-TCGTGAACG-TTACCCAAA--CCG-TTGCTTCGGCGTGGCCACTCTGAGTCT---TCTGTACTGAATAAGTCAAAACTTTCAACAACGGATCTCTTGGTTCTGGCATCGATGAAGAACGCAGCGAAATGCGATAAGTAATGTGAATTGCAGAATTCAGTGAATCATCGAATCTTTGAACGCACATTGCGCCCGCCAGTATCCTGGCGGGCATGCCTGTTCGAGCGTCATTTCAA---CCATCAAG-CCCCC----GGCTTGTGTTGGGG-ACCTGCG-GCT-GTC-CGCAGGCCCT-GAAAACCAGTGGCGGGCTCGCTAGTCAC-ACCGAGCGTAGTAGCATACATCTCGCTCAGGGCGT-GCTGCGGG-TTCCGGCCGTTAAACGACCTTC---ATAACCCAAGGTTGACC????????????????????????????????????????????????????AAAGAAACCAACAGGG-ATTGCCCTTAGTAACGGCGAAGTGAAGCGGCAACAGCTCAAATTTGAAATCTGGCTTC--GG-CCCGAGTTGTAATTTGCAGAGGTAAGCTTTAGGCGCGGCACCTTCTGAGTCCCCTGGAACGGGGCGCCATAGAGGGTGAGAGCCCCGTATAGTTGGATGCCTA-GCCTGTGTAAAGCTCCTTCGACGAGTCGAGTAGTTTGGGAATGCTGCTCAAAATGGGAGGTAAATTTCTTCTAAAGCTAAATACCGGCCAGAGACCGATAGCGCACAAGTAGAGTGATCGAAAGATGAAAAGCACTTTGAAAAGAGGGTTAAATAGCACGTGAAATTGTTGAAAGGGAAGCGCTTGTGACCAGACTTGCGCCGGGCTGATCATCCGGTGTTCTCACCGGTGCACTCTGCCCGGCTCAGGCCAGCATCGGTTCTCGCGGGGGGATAAAGGCCCTGGGAATGTAGCTCC--TCCGGGAGTG-TTATAGCCCGGGGTGTAATGCCCTCGCGGGGACCGAGGTTCGCGCATCTGCAAGGATGCTGGCGTAATGGTCATCAGCGACCCGTCTTGAAACACGGACCAAGGAGTCAAGGTTTTGCGCGAGTGTTTGGGTGTAAAACCCGCACGCGTAATGAAAGTGAACGTAGGTGAGAG--CTTCGGCGCATCATCGACCGATCCTGATGTATTCGGATGGATTTGAGTAGGAGCGTTAAGCCTTGGACCCGAAAGATGGTGAACTATGCTTGGATAGGGTGAAGCCAGAGGAAACTCTGGTGGAGGCTCGCAGCGGTTTCTGACGTGCAAATCGATCGTCAAATCTGAGCATGGGGGGCGAAAGACTAATCGAACCATCTAGTAGCTGGTTACCGCC????????????????????????????????????????????????????????????????????????????????????????????????????????????????????????????????????????????????????????????????????????????????????????????????????????????????????????????????????????????????????????????????????????????????????????????????????????????????????????????????????????????????????????????????????????????????????????????????????????????????????????????????????CTCGTCAATCTGGTTCAGGGGCTGCGGAGAAAGAATGTCATCTCGTTCGAGGTTTCACTCGTCAGGGACATTCGCGACCGCGAGTTCAAGATTTTCTCGGATGCCGGCCGCGTCATGAGACCTCTATTTACCGTGGAGCAAGATCCGAACGGCGAC---------AGCGGCGCCGAGGTGGGCGCGTTGATCCTCAACAAGGAGCACATTGCGCGGTTGGAGGCGGACAAGGAGCTAGGCAAATACC------------ATCCCGATTACTGGGGCTGGCCGGGGCTGTTGAAGTCGGGTGCAATTGAGTACCTCGATGCGGAAGAAGAGGAGACGGTCATGATCTGCATGACGCCTGAGGACCTCGACAATTTCCGTGCTCGGAAGAG-----------AGGGGAGG----AGGTCGAGGACACTTCCGGCGTGGGCAACAACCGTATCAAGACCAAACCGAACCCGACGACGCATATGTACACACACTGCGAGATCCACCCCAGCATGCTGCTTGGCATTTGCGCCAGTATCATTCCATTCCCCGATCACAACCAG??????????????????????????????????????????????????????????????????????????????????????????????????????????????????????????????????????????????????????????????????????????????????????????????????????????????????????????????????????????????????????????????????????????????????????????????????????????????????????????????????????????????????????????????????????????????????????????????????????????????????????????????????????????????????????????????????????????????????????????????????????????????????????????????????????????????????????????????????????????????????????????????????????????????????????????????????????????

>*Chaetomium_globosum*_CBS_160.62

ATTACAGAGTT-------GCAAAACTCCC-TAAACCA-TTGTGAACG-TTACCTATA--CCG-TTGCTTCGGCGTGGCCTCTCTGAGTCT---TCTGTACTGAATAAGTCAAAACTTTCAACAACGGATCTCTTGGTTCTGGCATCGATGAAGAACGCAGCGAAATGCGATAAGTAATGTGAATTGCAGAATTCAGTGAATCATCGAATCTTTGAACGCACATTGCGCCCGCCAGCATTCTGGCGGGCATGCCTGTTCGAGCGTCATTTCAA---CCATCAAG-CCCCC---GGGCTTGTGTTGGGG-ACCTGCG-GCT-GCC--GCAGGCCCT-GAAAAGCAGTGGCGGGCTCGCT-GTCGC-ACCGAGCGTAGTAGCATACATCTCGCTCTGGTCGC-GCCGCGGG-TTCCGGCCGTTAAACCACCTTT----TAACCCAAGGTTGACCTCGGATCAGGTAGGAAGACCCGCTGAACTTAA????????????????????AAAGAAACCAACAGGG-ATTGCCC-TAGTAACGGCG-AGTGAAGCGGCAACAGCTCAAATTTGAAATCTGGCTTC--GG-CCCGAGTTGTAATTTGCAGAGG-AAGCTTTAGGCGCGGCACCTTCTGAGTCCCCTGGAACGGGGCGCCATAGAGGGTGAGAGCCCCGTATAGTTGGATGCCTA-GCCTGTGTAAAGCTCCTTCGACGAGTCGAGTAGTTTGGGAATGCTGCTCAAAATGGGAGGTAAATTTCTTCTAAAGCTAAATACCGGCCAGAGACCGATAGCGCACAAGTAGAGTGATCGAAAGATGAAAAGCACTTTGAAAAGAGGGTTAAATAGCACGTGAAATTGTTGAAAGGGAAGCGCTTGTGACCAGACTTGCGCCGGGCGGATCATCCGGTGTTCTCACCGGTGCACTCCGCCCGGCTCAGGCCAGCATCGGTTCTCGCGGGGGGATAAAGGTCCTGGGAACGTAGCTCC--TCCGGGAGTG-TTATAGCCCGGGGCGTAATGCCCTCGCGGGGACCGAGGTTCGCGCATCTGCAAGGATGCTGGCGTAATGGTCATCAGCGACCCGTCTTGAAACACGGACCAAGGAGTCAAGGTTTTGCGCGAGTGTTTGGGTGTAAAACCCGCACGCGTAATGAAAGTGAACGTAGGTGAGAG--CTTCGGCGCATCATCGACCGATCCTGATGTTTTCGGATGGATTTGAGTAGGAGCGTTAAGCCTTGGACCCGAAAGATGGTGAACTATGCTTGGATAGGGTGAAGCCAGAGGAAACTCTGGTGGAGGCTCGCAGCGGTT-CTGACGTGCAAATCGATCGTCAAATCTGAGCAT-GGGGGCGAAAGACTAATCGAACCATCTAGTAGCTGGTTACCGCC????????????????????????????????????????????????????????????????????????????????????????????????????????????????????????????????????????????????????????????????????????????????????????????????????????????????????????????????????????????????????????????????????????????????????????????????????????????????????????????????????????????????????????????????????????????????????????????????????????????????????????????????????CTTGTCACGCTGGTTCAGGGGCTGCGGAGAAAGAACGTTATCTCGTTTGAGGTTTCGCTCGTTAGAGACATCCGCGACCGTGAGTTCAAGATCTTTTCAGATGCGGGTCGGGTGATGAGGCCGCTGTTCACGGTGGAGCAAGAACCGAATGGCGAG---------AGCGGCGCTGAGATGGGCGCACTGATCCTGAACAAGGATCATATTGGGCGCCTGAAGATGGACGCAGAGCTGGGCAAATACC------------ACCCGGACTACTGGGGCTGGCAAGGCCTGTTGAAGTCGGGCGCTATTGAGTATCTTGATGCTGAGGAGGAGGAGACGGTCATGATCTGCATGACCCCCCAGGATCTTGATCAGTTCCGTGCCCGCAAGAT------------GGGAAGGA--TCGAGCCGGACAACT-CCGGGTTGGGCAATAACCGGATCAAGACGAAACCAAATCCGACAACTCACATGTACACGCACTGCGAGATCCATCCGAGCATGCTCCTCGGCATCTGCGCAAGCATCATCCCCTTTCCTGATCATAACCAA??????????????????????????????????????????????????????????????????????????????????????????????????????????????????????????????????????????????????????????????????????????????????????????????????????????????????????????????????????????????????????????????????????????????????????????????????????????????????????????????????????????????????????????????????????????????????????????????????????????????????????????????????????????????????????????????????????????????????????????????????????????????????????????????????????????????????????????????????????????????????????????????????????????????????????????????????????????

>*Apodospora_gotlandica*_E00204952

???????????????????????????????????????????????????????????????????????????????????????????????????????????????????????????????????????????????????????????????????????????????????????????????????????????????????????????????????????????????????????????????????????????????????????????????????????????????????????????????????????????????????????????????????????????????????????????????????????????????????????????????????????????????????????????????????????????????????????????????????????????????????????AAAGAAACCAACAGGG-ATTGCCC-CAGTAACGGCG-AGTGAAGCGGCAACAGCTCAAATTTGAAATCTGGCTTC--GG-CCCGAGTTGTAATTTGTAGAGG-AAGCTTTTGGCGAGGTGCCTTCTGAGTCCCCTGGAACGGGGCGCCATAGAGGGTGAGAGCCCCGTATAGTTGGACACCTA-GCCTCTGTAAAGCTCCTTCGACGAGTCGAGTAGTTTGGGAATGCTGCTCAAAATGGGAGGTAAATTTCTTCTAAAGCTAAATATTGGCCAGAGACCGATAGCGCACAAGTAGAGTGATCGAAAGATGAAAAGCACTTTGAAAAGAGGGTTAAACAGCACGTGAAATTGTTGAAAGGGAAGCGCTTGTGACCAGACATGCGCCGGGCGGATCATCCGGTGTTCTCACCGGTGCACTCCGCCCGGCTCAGGCCAGCATCGGTTCTCGCGGGGGGATAAAGGCTCGGGGAACGTAGCTCC--TCCGGGAGTG-TTATAGCCCCGGGCGCAACGCCCTCGCGGGGACCGAGGTTCGCGCATCTGCAAGGATGCTGGCGTAATGGTCATCAGCGACCCGTCTTGAAACACGGACCAAGGAGTCAAGGTTTTGCGCGAGTGTTTGGGTGTTAAACCCGCACGCGTAATGAAAGTGAACGTAGGTGAGAG--CTTCGGCGCATCATCGACCGATCCTGATGTATTCGGATGGATTTGAGTAGGAGCGTTAAGCCTTGGACCCGAAAGATGGTGAACTATGCTTGGATAGGGTGAAGCCAGAGGAAACTCTGGTGGAGGCTCGCAGCGGTT-CTGACGTGCAAATCGATCGTCAAATCTGAGCAT-GGGGGCGAAAGACTAATCGAACCATCTAGTAGCTGGTTACCGCC??????????????????????????????????????????????????????????????????????????????????????????????????????????????????????????????????????????????????????????????????????????????????????????????????????????????????????????????????????????????????????????????????????????????????????????????????????????????????????????????????????????????????????????????????????????????????????????????????????????????????????????????????????????????????????????????????????????????????????????????????????????????????????????????????????????????????????????????????????????????????????????????????????????????????????????????????????????????????????????????????????????????????????????????????????????????????????????????????????????????????????????????????????????????????????????????????????????????????????????????????????????????????????????????????????????????????????????????????????????????????????????????????????????????????????????????????????????????????????????????????????????????????GCTTGTTGACCAAGTTCTTGATGTCGTTCGTCGTGAGGCTGAGGGTTGCGACTGCCTCCAGGGCTTCCAGATCACCCACTCGCTCGGTGGTGGTACCGGTGCCGGTATGGGTACTCTCCTCATCTCCAAGATCCGCGAGGAGTTCCCCGACCGCATGATGGCTACCTTCTCCGTCGTGCCCTCCCCCAAGGTCTCGGACACCGTTGTCGAACCCTACAACGCCACTCTCTCGGTGCACCAGCTCGTTGAGAACTCTGACGAGACCTTCTGCATTGACAACGAGGCTCTCTACGACATTTGCATGCGGACCCTCAAGCTGTCCAACCCCTCGTACGGCGACCTTAACCACCTGGTCTCGGCCGTCATGTCGGGTGTCACCGTCTCGCTGCGTTTCCCCGGCCAGCTCAACTCCGATCTCCGCAAGCTTGCTGTCAATATGGTTCCCTTCCCGCGTCTGCACTTCTTCATGGTCGGCTTTGCGCCGCTTACGAGCCGTGGCGCTCACTCTTTCCGTGCTGTTTCGGTTCCCGAGTTGACCCAGCAGATGTTCGACCCCAAGAACATGATGGCTGCTTCTGACTTCCGCAACGGCCGCTACCTGACCTGCTCTGCCATCTT

>*Apodospora_peruviana*_CBS_118394

ATTAAAGAGTT-------GCAAAACTCCCACAAACCA-TCGCGAACCGTACCGATTTA-CCG-TTGCTTCGGCGTGGCCTCTCTGAGTAA----GATTTATAAATAAGTCAAAACTTTCAACAACGGATCTCTTGGTTCTGGCATCGATGAAGAACGCAGCGAAATGCGATAAGTAATGTGAATTGCAGAATTCAGTGAATCATCGAATCTTTGAACGCACATTGCGCCCGCTAGTATTCTGGCGGGCATGCCTGTTCGAGCGTCATTTCAA---CCATCAAG-CTCTC-----GCTTGTGTTGGGG-TCCTGCG-GCT-GTC-CGCAGGCCCT-GAAAACCAGTGGCGGGCTCGCTAGTCAC-TCCGAGCGCAGTAATA-CACTCTCGCTCAGGGCGT-GCGGCGGG-CTCTTGCCGTTAAACCACCACTTTT-ATACCAAAGGTTGACCTCGGATCAGGTAGGAATACCCGCTGAACTTAAGCATATCAATAAGCGG??????????????????????TTGCCC-CAGTAACGGCG-AGTGAAGCGGCAACAGCTCAAATTTGAAATCTGGCTTC--GG-CCCGAGTTGTAATTTGTAGAGG-AAGCTTTTGGCGAGGTGCCTTCTGAGTCCCCTGGAACGGGGCGCCATAGAGGGTGAGAGCCCCGTATAGTTGGACACCTA-GCCTCTGTAAAGCTCCTTCGACGAGTCGAGTAGTTTGGGAATGCTGCTCAAAATGGGAGGTAAATTTCTTCTAAAGCTAAATATTGGCCAGAGACCGATAGCGCACAAGTAGAGTGATCGAAAGATGAAAAGCACTTTGAAAAGAGGGTTAAACAGCACGTGAAATTGTTGAAAGGGAAGCGCTTGTGACCAGACATGCGCCGGGCGGATCATCCGGTGTTCTCACCGGTGCACTCCGCCCGGCTCAGGCCAGCATCGGTTCTCGCGGGGGGATAAAGGCCTGGGGAACGTAGCTCC--TCCGGGAGTG-TTATAGCCCTGGGCGCAACGCCCTCGCGGAGACCGAGGTTCGCGCATCTGCAAGGATGCTGGCGTAATGGTCATCAGCGACCCGTCTTGAAACACGGACCAAGGAGTCAAGGTTTTGCGCGAGTGTTTGGGTGTTAAACCCGCACGCGTAATGAAAGTGAACGTAGGTGAGAG--CTTCGGCGCATCATCGACCGATCCTGATGTATTCGGATGGATTTGAGTAGGAGCGTTAAGCCTTGGACCCGAAAGATGGTGAACTATGCTTGGATAGGGTGAAGCCAGAGGAAACTCTGGTGGAGGCTCGCAGCGGTT-CTGACGTGCAAATCGATCGTCAAATCTGAGCAT-GGGGGCGAAAGACTAATCGAACCATCTAGTAGCTGGTTACCGCC????????????????????????????????????????????????????????????????????????????????????????????????????????????????????????????????????????????????????????????????????????????????????????????????????????????????????????????????????????????????????????????????????????????????????????????????????????????????????????????????????????????????????????????????????????????????????????????????????????????????????????????????????????????????????????????????????????????????????????????????????????????????????????????????????????????????????????????????????????????????????????????????????????????????????????????????????????????????????????????????????????????????????????????????????????????????????????????????????????????????????????????????????????????????????????????????????????????????????????????????????????????????????????????????????????????????????????????????????????????????????????????????????????????????????????????????????????????????????????????????????????????????????????????????????????????????????????????????????????????????????????????????????????????????????????????????????????????????????????????????????????????????????????????????????????????????????????????????????????????????????????????????????????????????????????????????????????????????????????????????????????????????????????????????????????????????????????????????????????????????????????????????????????????????????????????????????????????????????????????????????????????????????????????????????????????????????????????????????????????????????????????????????????????????????????????????????????????????????????????

>*Podospora_fabiformis*_CBS_112043

ATTACAGAGTT-------GCAAAACTCCC-TAAACCA-TCGCGAACG-TACCCCGTA--CAG-TTGCTTCGGCGAGGCCTCTCTGAGTAA------TACTATAATAAGTAAAAACTTTCAACAACGGATCTCTTGGTTCTGGCATCGATGAAGAACGCAGCGAAATGCGATAAGTAATGTGAATTGCAGAATTCAGTGAATCATCGAATCTTTGAACGCACATTGCGCCCGCTAGTATTCTGGCGGGCATGCCTGTTCGAGCGTCATTTCAA---CCATCAAG-CCCA------GCTTGTGTTGGGG-CCCTGCG-TCT------GCAGGCCCT-TAAAACCAGTGGCGGGCTCGCC-TACAC-TCCGAGCGCAGTAATA--CATCTCGTTCTGGTCGT-GTGGCGGG-CGCTTGCCGTAAAACACCCCCC-----TTCTCAAGGTTGACCTCGGATCAGGTAGGAATACCCGCTGAACTTAAGCATATCAATAAGCGGAGGAAAAGAAACCAACAGGG-ATTGCCT-CAGTAACGGCG-AGTGAAGCGGCAACAGCTCAAATTTGAAATCTGGCTTC--GG-CCCGAGTTGTAATTTGTAGAGG-AAGATTCTGGTAAGGCACCATCTGAGTCCCCTGGAACGGGGCGCCATAGAGGGTGAGAGCCCCGTATAGTTGGATGCCGA-GCCTCTGTGAATCTCCTTCGACGAGTCGAGTAGTTTGGGAATGCTGCTCTAAATGGGAGGTAAATTTCTTCTAAAGCTAAATATAGGCCAGAGACCGATAGCGCACAAGTAGAGTGATCGAAAGATGAAAAGCACTTTGAAAAGAGGGTTAAACAGCACGTGAAATTGTTGAAAGGGAAGCGCTTATGACCAGACTTGCGCCGTGCGGATCATCCGGTGTTCTCACCGGTGCACTCCGCGCGGCTCAGGCCAGCATCGGTTCTCGTGGGGGGATAAAGGCTCAGGGAACGTAGCTCC--TCCGGGAGTG-TTATAGCCCTGGGTGCAATGCCCTCGCGGGGACCGAGGTTCGCGCTTCTGCAAGGATGCTGGCGTAATGGTCATCAGCGACCCGTCTTGAAACACGGACCAAGGAGTCAAGGTTTTGCGCGAGTGTTTGGGTGTTAAACCCGCACGCGTAATGAAAGTGAACGTAGGTGAGAG--CTTCGGCGCATCATCGACCGATCCTGATGTATTCGGATGGATTTGAGTAGGAGCGTTAAGCCTTGGACCCGAAAGATGGTGAACTATGCTTGGATAGGGTGAAGCCAGAGGAAACTCTGGTGGAGGCTCGCAGCGGTT-CTGACGTGCAAATCGATCGTCAAATCTGAGCAT-GGGGGCGAAAGAC?????????????????????????????????????????????????????????????????????????????????????????????????????????????????????????????????????????????????CATTTGAGACGAACCAATACTCCAATCGGCCGTGATGGCAAGCTTGCAAAGCCTCGCCAGCTTCATAACACACATTGGGGTCTGGTGTGCCCAGCCGAAACGCCAGAGGGACAAGCTTGTGGTCTCGTCAAGAACTTGTCTCTCATGTGTTTTGTGAGTGTGGGAACACCGGCAGAGCCCATAATAGAGTTCATGATTGCTCGCAACATGGAGGTTCTTGAAGAATATGAACCTCTGCGGTACCCCAATGCTACCAAGGTGTTTGTCAACGGCACGTGGGTCGGTGTCCATCAGGACCCAAAACATTTAGTGACGCTGGTTCAGGGGTTGCGTCGCAAAAACGTTATCTCCTACGAGGTGTCACTGGTTAGAGATATCCGTGACAGAGAGTTCAAGATTTTCTCAGATGCTGGTCGCGTCATGAGGCCACTCTTTGTTGTAGAGCAAGAGGAAAACAGCGAA---------TCTGGAGTTGAAAAGGGACAGTTGATTCTGACCAAGGAACATATTGCCAAGCTGGACGAAGACAAGATGCTTGGCAAATATC------------ATCCGGATTTCTGGGGCTGGGAAGGCCTTCGACGCTCTGGTGCCATCGAATACCTTGATGCTGAAGAGGAAGAAACGTCCATGATATGCATGTCCCCCGAAGATTTGGAGACCTATCGACTGAATAGATT------------GGGTATCGA---AATTGTGAAGGAGGCCGATGGACCCAACCAACGTCTTGCCACCAAGAGAAATGAGAACCTTCACATGTACACACATTGTGAGATCCATCCCAGCATGTTGCTTGGTATTTGCGCCAGTATCATTCCCTTCCCTGATCACAACCAG??????????????????????????????????????????????????????????????????????????????????????????????????????????????????????????????????????????????????????????????????????????????????????????????????????????????????????????????????????????????????????????????????????????????????????????????????????????????????????????????????????????????????????????????????????????????????????????????????????????????????????????????????????????????????????????????????????????????????????????????????????????????????????????????????????????????????????????????????????????????????????????????????????????????????????????????????????????

>*Ramophialophora_vesiculosa*_CBS_110629

ATTACAGAGTT-------GCAAAACTCCC-TAAACCA-TCGCGAACG-TACCCGTA---TCG-TTGCTTCGGCGTGGCCTCTCTGAGTAA------TTTTATAATAAGTAAAAACTTTCAACAACGGATCTCTTGGTTCTGGCATCGATGAAGAACGCAGCGAAATGCGATAAGTAATGTGAATTGCAGAATTCAGTGAATCATCGAATCTTTGAACGCACATTGCGCCCGCTAGTATTCTGGCGGGCATGCCTGTTCGAGCGTCATTTCAA---CCATCAAG-CCCT------GCTTGTGTTGGGG-TCCTGCG-GCT-GTC-CGCAGGCCCT-TAAAACCAGTGGCGGGCTCGCTAGTCAC-TCCGAGCGCAGTAATA--CATCTCGCTCTGGACGT-GCTGCGGG-TGCCGGCCGTAAAACCCCCCCCTTTCTTACGCAAGGTTGACCTCGGATCAGG??????????????????????GCATATCAATAAGCGGAGGAAAAGAAACCAACAGGG-ATTGTCC-TAGTAACGGCG-AGTGAAGCGACAACAGCTCAAATTTGAAATCTGGCTTC--GG-CCCGAGTTGTAATTTGTAGAGG-ATGCTTTTGGTGAGACGCTTTCTGAGTCCCCTGGAACGGGGCGCCATAGAGGGTGAGAGCCCCGTATAGTCAGACGTCAA-GCCTCTGTAAAGCTCCTTCGACGAGTCGAGTAGTTTGGGAATGCTGCTCAAAATGGGAGGTAAATTTCTTCTAAAGCTAAATATAGGCCAGAGACCGATAGCGCACAAGTAGAGTGATCGAAAGATGAAAAGCACTTTGAAAAGAGGGTTAAACAGCACGTGAAATTGTTGAAAGGGAAGCGCTTGTGACCAGACTTGCGCCGGGCTGATCATCCGCTGTTCTCAGCGGTGCACTCTGCCCGGCTCAGGCCAGCATCGGTTCTCGTGGGGGGATAAAAGCCTGGGGAACGTAGCTCC--TCCGGGAGTG-TTATAGCCCTGGGCACAACGCCCTCGCGGGGACCGAGGATCGCGC-TCTGCAAGGATGCTGGCGTAATGGTCACCAGCGACCCGTCTTGAAACACGGACCAAGGAGTCAAGGTTTTGCGCGAGTGTTTGGGTGTAAAACCCGCACGCGTAATGAAAGTGAACGTAGGTGAGAG--CTTCGGCGCATCATCGACCGATCCTGATGTATTCGGATGGATTTGAGTAAGAGCGTTAAGCCTTGGACCCGAAAGATGGTGAACTATGCTTGGATAGGGTGAAGCCAGAGGAAACTCTGGTGGAGGCTCGCAGCGGTT-CTGACGTGCAAATCGATCGTCAAATCTGAGCAT-GGGGGCGAAAGACTAATCGAACCATCTAGTAGCTGGTTACCGCC????????????????????????????????????????????????????????????????????????????????????????????????????????????????????????????????????????????????????????????????????????????????????????????????????????????????????????????????????????????????????????????????????????????????????????????????????????????????????????????????????????????????????????????????????????????????????????????????????????????????????????????????????????????????????????????????????????????????????????????????????????????????????????????????????????????????????????????????????????????????????????????????????????????????????????????????????????????????????????????????????????????????????????????????????????????????????????????????????????????????????????????????????????????????????????????????????????????????????????????????????????????????????????????????????????????????????????????????????????????????????????????????????????????????????????????????????????????????????????????????????????????????????????????????????????????????????????????????????????????????????????????????????????????????????????????????????????????????????????????????????????????????????????????????????????????????????????????????????????????????????????????????????????????????????????????????????????????????????????????????????????????????????????????????????????????????????????????????????????????????????????????????????????????????????????????????????????????????????????????????????????????????????????????????????????????????????????????????????????????????????????????????????????????????????????????????????????????????????????????

>*Zopfiella_attenuata*_CBS_266.77

ATTACAGAGTT-------GCAA-ACTCCCTTAAACCA-TCGCGAACG-TACCCGTAC--ATG-TTGCTTCGGCGGGGCCTCTCTGAGTAA------TACTATAATAAGTAAAAACTTTCAACAACGGATCTCTTGGTTCTGGCATCGATGAAGAACGCAGCGAAATGCGATAAGTAATGTGAATTGCAGAATTCAGTGAATCATCGAATCTTTGAACGCACATTGCGCCCGCCAGTATTCTGGCGGGCATGCCTGTTCGAGCGTCATTTCAA---CCATCAAG-CCCT------GCTTGTGTTGGGG-TCCTGCG-GCT-GTC-CGCAGGCCCT-TAAAACCAGTGGCGGGCTCGCC-TACAC-TCCGAGCGCAGTAATA--CATCTCGTTCTGGTCGT-GTGGTGGG-TTCCAGCCGTTAAACACCCCCC----TTTCGTAAGGTTGACC????????????????????????????????????????????????????AAAGAAACCAACAGGG-ATTGCCC-TAGTAACGGCG-AGTGAAGCGGCAACAGCTCAAATTTGAAATCTGGCTTC--GG-CCCGAGTTGTAATTTGTAGAGG-AAGCTTTTGGCGAGGTACCTTCTGAGTCCACTGGAACGTGGCGCCATAGAGGGTGAGAGCCCCGTATAGTTGGATACCTCTGCCTCTGTAAAGCTCCTTCGACGAGTCGAGTAGTTTGGGAATGCTGCTCTAAATGGGAGGTAAATTTCTTCTAAAGCTAAATACCGGCCAGAGACCGATAGCGCACAAGTAGAGTGATCGAAAGATGAAAAGCACTTTGAAAAGAGGGTTAAACAGCACGTGAAATTGTTGAAAGGGAAGCGCTTGTGATCAGACTTGCGCCGGGCGGATCATCCGGTGTTCTCACCGGTGCACTCCGCCCGGCTCAGGCCAGCATCGGTTCTCGTGGGGGGATAAAGGCTCGGGGAACGTGGCTCC--TCCGGGAGTG-TTATAGCCCCGGGCGCAACGCCCTCGCGGGGACCGAGGTTCGCGCATCTGCAAGGATGCTGGCGTAATGGTCACCAGCGACCCGTCTTGAAACACGGACCAAGGAGTCAAGGTTTTGCGCGAGTGTTTGGGTGTTAAACCCGCACGCGTAATGAAAGTGAACGTAGGTGAGAG--CTTCGGCGCATCATCGACCGATCCTGATGTATTCGGATGGATTTGAGTAGGAGCGTTAAGCCTTGGACCCGAAAGATGGTGAACTATGCTTGGATAGGGTGAAGCCAGAGGAAACTCTGGTGGAGGCTCGCAGCGGTT-CTGACGTGCAAATCGATCGTCATA??????????????????????????????????????????????????????GCCCTCAAGTACTCTTTGGCGACGGGCAACTGGGGTGACCAGAAGAAGGCTGCAAGCTCCACTGCCGGTGTCTCTCAGGTGTTGAACCGTTACACTTTCGCCAGTACACTATCCCATTTGAGGCGCACCAACACCCCGATTGGTCGTGATGGCAAGATTGCAAAGCCTCGTCAGCTCCACAACACTCATTGGGGCCTGGTCTGCCCAGCAGAGACGCCAGAAGGACAAGCTTGCGGTCTTGTCAAGAACCTTTCGCTCATGTGCTATGTCAGTGTGGGCACACCGGCCGATCCGATCGTCGAGTTCATGGTTGCGAGAAATATGGAAGTTTTGGAGGAGTACGAACCGCTACGATATCCCAACGCCACCAAGGTCTTTGTCAATGGCACATGGGTAGGTGTCCACCAAGACGCCAAACATCTCGTGAGCTTGGTACAGGACCTCCGCAGGAAGAACATTATCTCCTTCGAGGTTTCGCTGGTCCGAGATATCCGTGATAGAGAGTTCAAAATTTTCTCTGATGCCGGTCGAGTCATGAGGCCCCTCCTAGCAATCGAGCAAGAGGACGAGAACGAT---------CACAATGTCGCCAAGGGAGGCCTTATTTTGACCCAGAAACACATCAAGAAGTTGCACCGGGACGCGGAACTGGGCAAGTATC------------ACCCCGAGTTTTGGGGTTGGAACGGTCTCCAGCGCTGCGGTGCAGTCGAATACCTCGACGCAGAGGAAGAAGAAACATGCATGATATGCATGACGCCTGACGACTTGATCGATTTC???????????????????????????????????????????????????????????????????????????????????????????????????????????????????????????????????????????????????????????????????????????????????????GCTTGTCGACCAAGTTCTCGATGTCGTCCGTCGCGAGGCTGAGGGCTGCGACTGCCTCCAGGGTTTCCAGATCACCCACTCTCTCGGTGGTGGTACCGGTGCCGGTATGGGTACCCTCCTTATCTCCAAGATCCGCGAGGAGTTTCCCGACCGCATGATGGCCACCTTCTCCGTCGTTCCCTCCCCCAAGGTCTCCGACACCGTTGTCGAACCCTACAACGCCACCCTCTCCGTCCACCAGCTTGTCGAGAACTCGGACGAGACCTTCTGCATTGACAACGAGGCTCTCTACGACATCTGCATGCGCACCCTGAAGCTGTCCAACCCCTCTTACGGCGATCTCAACCACTTGGTTTCCGCTGTCATGTCCGGTGTCACCGTTTCCCTGCGTTTCCCTGGTCAGCTCAACTCTGACCTCCGCAAGCTCGCCGTCAACATGGTTCCCTTCCCCCGTCTCCATTTCTTCATGGTCGGATTCGCCCCCCTTACTAGCCGTGGCGCGCATTCTTTCCGTGCTGTTTCCGTCCCCGAGTTGACCCAGCAGATGTTCGACCCCAAGAACATGATGGCTGCTTCCGACTTCCGCAACGGCCGCTACCTCACCTGCTCTGCCATCTT

>*Zopfiella_pleuropora*_CBS_518.70

ATTACAGAGTT-------GCAAAACTCCCTTAAACCA-TCGCGAACG-TACCCGTAC--ATG-TTGCTTCGGCGGGGCCTCTCTGAGTAA------TACTATAATAAGTAAAAACTTTCAACAACGGATCTCTTGGTTCTGGCATCGATGAAGAACGCAGCGAAATGCGATAAGTAATGTGAATTGCAGAATTCAGTGATTCATCGAATCTTTGAACGCACATTGCGCCCGCCAGTATTCTGGCGGGCATGCCTGTTCGAGCGTCATTTCAA---CCATCAAG-CCCT------GCTTGTGTTGGGG-TCCTGCG-GCT-GTC-CGCAGGCCCT-TAAAACCAGTGGCGGGCTCGCC-TACAC-TCCGAGCGCAGTAATA--CATCTCGTTCTGGTCGT-GTGGTGGG-TTCCAGCCGTTAAACACCCCCC----TTTCGTAAGGTTGACCTCGGATCAGGTAGGAATACCCGCTGAACTTAAGCATATCAATAAGCGGAGGAAAAGAAACCAACAGGG-ATTGCCC-TAGTAACGGCG-AGTGAAGCGGCAACAGCTCAAATTTGAAATCTGGCTTC--GG-CCCGAGTTGTAATTTGTAGAGG-AAGCTTTTGGCGAGGTACCTTCTGAGTCCACTGGAACGTGGCGCCATAGAGGGTGAGAGCCCCGTATAGTTGGATACCTCTGCCTCTGTAAAGCTCCTTCGACGAGTCGAGTAGTTTGGGAATGCTGCTCAAAATGGGAGGTAAATTTCTTCTAAAGCTAAATACTGGCCAGAGACCGATAGCGCACAAGTAGAGTGATCGAAAGATGAAAAGCACTTTGAAAAGAGGGTTAAACAGCACGTGAAATTGTTGAAAGGGAAGCGCTTGTGATCAGACTTGCGCCGGGCGGATCATCCGGTGTTCTCACCGGTGCACTCCGCCCGGCTCAGGCCAGCATCGGTTCTCGTGGGGGGATAAAGGCTCGGGGAACGTGGCTCC--TCCGGGAGTG-TTATAGCCCCGGGCGCAACGCCCTCGCGGGGACCGAGGTTCGCGCATCTGCAAGGATGCTGGCGTAATGGTCACCAGCGACCCGTCTTGAAACACGGACCAAGGAGTCAAGGTTTTGCGCGAGTGTTTGGGTGTTAAACCCGCACGCGTAATGAAAGTGAACGTAGGTGAGAG--CTTCGGCGCATCATCGACCGATCCTGATGTATTCGGATGGATTTGAGTAGGAGCGTTAAGCCTTGGACCCGAAAGATGGTGAACTATGCTTGGATAGGGTGAAGCCAGAGGAAACTCTGGTGGAGGCTCGCAGCGGTT-CTGACGTGCAAATCGATCGTCAAATCTGAGCAT-GGGGGCGAAAGACTAATCGAACCATCTAGTAGCTGGTTACCGCCGCGCTCAAGTACTCTTTGGCGACGGGCAACTGGGGTGACCAGAAGAAGGCTGCAAGCTCCACTGCCGGTGTCTCTCAGGTGTTGAACCGTTACACTTTTGCCAGTACACTATCCCATTTGAGGCGCACCAACACCCCGATTGGCCGTGATGGCAAGATTGCAAAGCCTCGTCAGCTCCACAACACTCATTGGGGCCTGGTCTGTCCAGCAGAGACGCCAGAAGGACAAGCTTGCGGTCTTGTCAAGAACCTTTCGCTCATGTGCTATGTCAGTGTGGGCACACCGGCCCATCCGATCGTCGAGTTCATGGTTGCGAGAAATATGGAAGTTTTGGAGGAGTACGAACCGCTACGATACCCCAACGCCACCAAGGTCTTTGTCAATGGTACATGGGTAGGCGTCCACCAAGACCCCAAACATCTTGTGAGCTTGGTACAGGACCTCCGCAGGAAGAACATTATCTCCTTTGAGGTTTCGCTGGTTCGAGATATCCGTGATAGAGAGTTCAAAATTTTCTCGGATGCTGGTCGAGTCATGAGGCCCCTTCTAGCAATCGAGCAAGAGGACGAGAACGAC---------CACGATGTCGCCAAGGGAGGCCTTATTTTGACCCAGAAACACATCAAGAAGTTGCACCGGGACGCGGAACTGGGCAAGTATC------------ACCCCGAGTTTTGGGGTTGGAACGGTCTCCAGCGCTGCGGTGCGGTCGAATACCTTGACGCGGAGGAAGAAGAAACATCCATGATCTGCATGACGCCTGACGACTTGATCGATTTC???????????????????????????????????????????????????????????????????????????????????????????????????????????????????????????????????????????????????????????????????????????????????????GCTTGTCGACCAAGTTCTCGATGTCGTCCGTCGCGAGGCTGAGGGCTGCGACTGCCTCCAGGGTTTCCAGATCACCCACTCTCTCGGTGGTGGTACCGGTGCCGGTATGGGTACCCTCCTTATCTCCAAGATTCGCGAGGAGTTCCCCGACCGCATGATGGCCACCTTCTCCGTCGTTCCCTCCCCCAAGGTCTCCGACACCGTTGTCGAGCCCTACAACGCCACCCTCTCCGTCCACCAGCTTGTCGAGAACTCGGACGAGACCTTCTGCATTGACAATGAGGCTCTCTACGACATTTGCATGCGCACCCTTAAGCTGTCCAACCCCTCTTATGGCGATCTCAACCACTTGGTTTCGGCTGTCATGTCCGGTGTCACCGTTTCCCTGCGTTTCCCTGGTCAGCTCAACTCTGACCTCCGCAAGCTCGCCGTCAACATGGTTCCCTTCCCCCGTCTCCATTTCTTCATGGTCGGATTCGCCCCCCTTACTAGCCGTGGCGCGCATTCTTTCCGTGCTGTTTCCGTCCCCGAGTTGACCCAGCAGATGTTCGACCCCAAGAACATGATGGCTGCTTCCGATTTCCGCAACGGTCGTTACCTCACCTGCTCTGCCATCTT

>*Podospora_fibrinocaudata*_CBS_315.91

ATTACAGAGTT-------GCAAAACTCCC-TAAACCA-TCGCGAACG-TACCCCGTA--CAG-TTGCTTCGGCGAGGCCTCTCTGAGTAA------TACTATAATAAGTAAAAACTTTCAACAACGGATCTCTTGGTTCTGGCATCGATGAAGAACGCAGCGAAATGCGATAAGTAATGTGAATTGCAGAATTCAGTGAATCATCGAATCTTTGAACGCACATTGCGCCCGCTAGTATTCTGGCGGGCATGCCTGTTCGAGCGTCATTTCAA---CCATCAAG-CCCA------GCTTGTGTTGGGG-CCCTGCG-CCT------GCAGGCCCT-TAAAACCAGTGGCGGGCTCGCC-TACAC-TCCGAGCGCAGTAATA--CATCTCGTTCTGGTCGT-GTGGCGGG-CGCTTGCCGTAAAACACCCCCC-----TTCTCAAGGTTGACCTCGGATCAGGTAGGAATACCCGCTGAACTTAAGCATATCAATAAGCGGAGGAAAAGAAACCAACAGGG-ATTGCCT-CAGTAACGGCG-AGTGAAGCGGCAACAGCTCAAATTTGAAATCTGGCTTC--GG-CCCGAGTTGTAATTTGTAGAGG-AAGATTCTGGTAAGGCACCATCTGAGTCCCCTGGAACGGGGCGCCATAGAGGGTGAGAGCCCCGTATAGTTGGATGCCGA-GCCTCTGTGAATCTCCTTCGACGAGTCGAGTAGTTTGGGAATGCTGCTCTAAATGGGAGGTAAATTTCTTCTAAAGCTAAATATAGGCCAGAGACCGATAGCGCACAAGTAGAGTGATCGAAAGATGAAAAGCACTTTGAAAAGAGGGTTAAACAGCACGTGAAATTGTTGAAAGGGAAGCGCTTATGACCAGACTTGCGCCGTGCGGATCATCCGGTGTTCTCACCGGTGCACTCCGCGCGGCTCAGGCCAGCATCGGTTCTCGTGGGGGGATAAAGGCTCAGGGAACGTAGCTCC--TCCGGGAGTG-TTATAGCCCTGGGTGCAATGCCCTCGTGGGGACCGAGGTTCGCGCTTCTGCAAGGATG??????????????????????????????????????????????????????????????????????????????????????????????????????????????????????????????????????????????????????????????????????????????????????????????????????????????????????????????????????????????????????????????????????????????????????????????????????????????????????????????????????????????????????????????????????????????????????????????????????????????????????????????????????????????????????????TTATCTCATTTGAGACGAACCAATACTCCAATCGGACGTGATGGCAAGCTTGCAAAGCCTCGCCAGCTTCATAACACACATTGGGGTCTGGTGTGCCCGGCCGAAACGCCAGAGGGACAAGCTTGTGGTCTCGTCAAGAATTTGTCTCTCATGTGCTTTGTGAGTGTGGGAACACCGGCAGAGCCCATAATAGAGTTCATGATTGCTCGCAACATGGAGGTGCTTGAAGAGTACGAACCTCTACGGTACCCTAATGCCACCAAGGTGTTTGTCAACGGCACGTGGGTCGGTGTCCATCAGGACCCAAAACATCTAGTGACGCTGGTCCAGGGRTTGCGTCGCAAAAACGTCATCTCGTACGAGGTGTCACTGGTTAGAGATATCCGTGACAGAGAGTTCAAGATTTTCTCAGATGCTGGTCGTGTTATGAGGCCACTCTTTGTTGTGGAGCAAGAGGAAAACAGCGAA---------TCTGGGGTTGAAAAGGGACAGCTGATTCTGACCAAGGAACATATTGCCAAGCTGGACGAAGACAAGATGCTTGGCAAATATC------------ATCCGGATTTCTGGGGCTGGGAAGGCCTTCGACGCTCTGGTGCCATCGAATACCTTGATGCTGAAGAGGAAGAAACGTCCATGATCTGCATGTCCCCCGAAGATTTGGAGACCTACCGACTGAATAGACT------------GGGTATCGA---AATTGTGAAGGAGGCTGATGGACCCAACCAACGTCTTGCCACCAAGAGAAATGAGAACCTACACATGTATACGCATTGCGAGATCCATCCCAGCATGTTGCTTGGTATTTGCGCCAGCATTATTCCCTTCCCCGATCACAACCAG??????????????????????????????????????????????????????????????????????????????????????????????????????????????????????????????????????????????????????????????????????????????????????????????????????????????????????????????????????????????????????????????????????????????????????????????????????????????????????????????????????????????????????????????????????????????????????????????????????????????????????????????????????????????????????????????????????????????????????????????????????????????????????????????????????????????????????????????????????????????????????????????????????????????????????????????????????????

>*Fimetariella_rabenhorstii_Lundqvist*_20410

???????????????????????????????????????????????????????????????????????????????????????????????????????????????????????????????????????????????????????????????????????????????????????????????????????????????????????????????????????????????????????????????????????????????????????????????????????????????????????????????????????????????????????????????????????????????????????????????????????????????????????????????????????????????????????????????????????????????????????????????????????????????????????AAAGAAACCAACAGGG-ATTGCCC-CAGTAACGGCG-AGTGAAGCGGCAACAGCTCAAATTTGAAATCTGGCCTC--GG-CCCGAGTTGTAATTTGTAGAGG-AAGCTTTTGGTGCGGCACTTACTGAGTCCCCTGGAACGGGGCGCCAGAGAGGGTGAGAGCCCCGTATAGTTTGATGCCTA-GCCTTTGTAAAGCTCCTTCGACGAGTCGAGTAGTTTGGGAATGCTGCTCTAAATGGGAGGTAAATTTCTTCTAAAGCTAAATATTGGCCAGAGACCGATAGCGCACAAGTAGAGTGATCGAAAGATGAAAAGCACTTTGAAAAGAGGGTTAAATAGCACGTGAAATTGTTGAAAGGGAAGCGCTTGTGACCAGACTTGCGCCGGGCGGATCATCCGGTGTTCTCACCGGTGCACTCCGCCCGGCTCAGGCCAGCATCGGTTCTCGCGGGGGGATAAAGGTTCAGGGAATGTGGCTCC--TCCGGGAGTG-TTATAGCCCTGGGCGCAACGCCCCCGTGGGGACCGAGGATCGCGC-TCTGCAAGGATGCTGGCGTAATGGTCACCAGCGACCCGTCTTGAAACACGGACCAAGGAGTCAAGGTTTTGCGCGAGTGTTTGGGTGTAAAACCCGCACGCGTAATGAAAGTGAACGTAGGTGAGAG--CTTCGGCGCATCATCGACCGATCCTGATGTATTCGGATGGATTTGAGTAGGAGCGTTAAGCCTTGGACCCGAAAGATGGTGAACTATGCTTGGATAGGGTGAAGCCAGAGGAAACTCTGGTGGAGGCTCGCAGCGGTT-CTGACGTGCAAATCGATCGTCAAATCTGAGCAT-GGGGGCGAAAGACTAATCGAACCATCTAGTAGCTGGTTACCGCC??????????????????????????????????????????????????????????????????????????????????????????????????????????????????????????????????????????????????????????????????????????????????????????????????????????????????????????????????????????????????????????????????????????????????????????????????????????????????????????????????????????????????????????????????????????????????????????????????????????????????????????????????????????????????????????????????????????????????????????????????????????????????????????????????????????????????????????????????????????????????????????????????????????????????????????????????????????????????????????????????????????????????????????????????????????????????????????????????????????????????????????????????????????????????????????????????????????????????????????????????????????????????????????????????????????????????????????????????????????????????????????????????????????????????????????????????????????????????????????????????????????????????GCTTGTTGACCAAGTTCTCGATGTGGTCCGTCGTGAGGCCGAGGGCTGCGACTGCCTCCAGGGCTTCCAGATCACCCACTCTCTCGGTGGTGGTACCGGTGCCGGTATGGGCACCCTGCTTATCTCTAAGATCCGCGAGGAGTTCCCCGACCGCATGATGGCCACCTTCTCCGTCGTGCCCTCGCCCAAGGTTTCGGACACCGTCGTCGAGCCGTACAACGCGACCCTCTCGGTCCACCAGCTGGTCGAGAACTCGGACGAGACCTTCTGCATTGACAACGAGGCGCTCTACGACATCTGCATGCGGACTCTCAAGCTGTCCAACCCGTCGTATGGCGATCTTAACCACCTGGTCTCGGCCGTCATGTCGGGTGTGACCGTCTCCCTCCGCTTCCCTGGCCAGCTCAACTCCGATCTCCGCAAGTTGGCTGTCAACATGGTTCCTTTCCCCCGTCTCCACTTCTTCATGGTCGGATTCGCGCCTCTTACGAGCCGTGGCGCGCATTCGTTCCGTGCCATCTCCGTTCCGGAGTTGACCCAGCAGATGTTCGACCCTAAGAACATGATGGCCGCTTCGGATTTCCGCAACGGTCGCTACCTGACCTGCTCCGCCATCTT

>*Podospora_appendiculata*_CBS_212.97

ATTACAGAGTT-------GCAAAACTCCCCTAAACCA-TCGCGAACG-TACCCGTA---CCG-TTGCTTCGGTG-GGCCTCTCTGAGTAT------TTTTATAATAAGTAAAAACTTTCAACAACGGATCTCTTGGTTCTGGCATCGATGAAGAACGCAGCGAAATGCGATAAGTAATGTGAATTGCAGAATTCAGTGAATCATCGAATCTTTGAACGCACATTGCGCCCGCCAGTATTCTGGCGGGCATGCCTGTTCGAGCGTCATTTCAA---CCATCAAG-CCCT------GCTTGCGTTGGGG-TCCTGCG-GCT-GCC-CGCAGGCCCT-TAAAACCAGTGGCGGGCTCGCT-GTCAC-TCCGAGCGCAGTAATA-CATCTCGCTTTGGACCGT-GCAGCGGG-TACCGGCCGTTAAACACCCCCC----TTTTACAAGGTTGACCTCGGATCAGGTAGGAATACCCGCTGAACTTAAGCATATCAATA?????????AAAGAAACCAACAGGG-ATTGCCC-CAGTAACGGCG-AGTGAAGCGGCAACAGCTCAAATTTGAAATCTGGCTTC--GG-CCCGAGTTGTAATTTGTAGAGG-AAGATTTTGGTGAGGCACCTTCTGAGTCCCCTGGAACGGGGCGCCATAGAGGGTGAGAGCCCCGTATAGCTGGATGCCTA-GCCTGTGTAAATCTCCTTCGACGAGTCGAGTAGTTTGGGAATGCTGCTCTAAATGGGAGGTAAATTTCTTCTAAAGCTAAATATTGGCCAGAGACCGATAGCGCACAAGTAGAGTGATCGAAAGATGAAAAGCACTTTGAAAAGAGGGTTAAACAGCACGTGAAATTGTTGAAAGGGAAGCGCTTGTGACCAGACTTGCGCCGGGCGGATCATCCGGTGTTCTCACCGGTGCACTCCGCCCGGCTCAGGCCAGCATCGGTTTTCGTGGGGGGATAAAGGCCCGGGGAACGTAGCTCC--TCCGGGAGTG-TTATAGCCCTGGGCGCAACGCCCTCGCGGGGACCGAGGTTCGCGCTTCTGCAAGGATGCTGGCGTAATGGTCATCAGCGACCCGTCTTGAAACACGGACCAAGGAGTCAAGGTTTTGCGCGAGTGTTTGGGTGTAAAACCCGCACGCGTAATGAAAGTGAACGTAGGTGAGAG--CTTCGGCGCATCATCGACCGATCCTGATGTATTCGGATGGATTTGAGTAGGAGCGTTAAGCCTTGGACCCGAAAGATGGTGAACTATGCTTGGATAGGGTGAAGCCAGAGGAAACTCTGGTGGAGGCTCGCAGCGGTT-CTGACGTGCAAATCGATCGTCAAATCTGAGCAT-GGGGGCGAAAGACTAATCGAACCATCTAGTAGCTGGTTACCGCCGCCCTGAAATACTCGTTGGCAACAGGCAACTGGGGTGACCAGAAAAAGGCAATGAGCTCGACCGCCGGTGTCTCCCAGGTGTTGAACCGATACACCTTCGCATCCACACTCTCTCATCTGAGACGAACCAATACCCCAATTGGCCGCGATGGCAAGTTGGCCAAGCCACGGCAGTTGCACAACACCCATTGGGGCTTGGTGTGCCCTGCAGAGACCCCCGAAGGACAGGCCTGCGGTCTTGTGAAGAACCTATCGCTCATGTGCTTCGTCAGTGTGGGCACACCTGCGGATCCAATCGTTGAGTTCATGATCGCCAGGAATATGGAAGTCTTGGAAGAGTATGAGCCTCTGAGGTACCCCAACGCCACCAAGGTCTTTGTGAACGGTACCTGGGTCGGCGTGCATCAAGACCCCAAGCATCTCGTCAGCTTGGTCCAGGGGTTGCGCAGAAAGAACATCATCTCGTTCGAAGTGTCGCTGGTACGGGACATTCGCGATAGAGAGTTCAAGATCTTTTCTGATGCTGGGCGAGTTATGCGGCCGCTGTTTGTCGTGGAGCAAGAGGAGAACAGCGAC---------AGCGGCGTCGCGAAGGGACAGCTTGTTCTGACCAAGGACCACATCCGACAGCTAGAGACGGACAAGGAGCTTGGCAAATACC------------ATGAGCAGTACTGGGGCTGGAAGGGACTGAGAGAATCTGGCGCCATCGAATACCTCGACGCCGAAGAGGAAGAAACAACCATGATCTGCATGACGCCGGACGACTTGGATAATTACCGAATAACAAAGCT------------CATGGGCCAGCCTGCCGAAGAGGACGACAGAACTGGCAATGCTCGGATCAAGACAAAGATGAATCCCACAACGCACATGTATACGCATTGCGAGATCCACCCCAGCATGCTGCTTGGCATTTGCGCAAGCATCATTCCTTTCCCCGACCACAATCAAGCTTGTTGACCAAGTTCTCGATGTCGTCCGCCGCGAGGCTGAGGGCTGCGACTGCCTCCAGGGCTTCCAGATCACCCACTCCCTCGGTGGTGGTACCGGTGCCGGTATGGGAACCCTCCTGATCTCCAAGATCCGCGAGGAGTTCCCCGATCGCATGATGGCTACCTTCTCCGTTGTGCCCTCGCCCAAGGTGTCCGACACCGTTGTCGAGCCCTACAACGCCACCCTGTCGGTTCATCAGCTCGTCGAGAACTCGGACGAGACCTTTTGCATTGACAACGAGGCTTTGTACGACATTTGCATGCGTACTCTGAAGCTGTCCAACCCCTCGTACGGCGATCTCAACCACCTGGTTTCCGCCGTCATGTCGGGTGTCACAGTTTCCCTGCGCTTCCCCGGCCAGCTCAACTCTGATCTCCGCAAGTTGGCTGTCAACATGGTTCCCTTCCCCCGTCTCCATTTCTTCATGGTCGGATTTGCCCCTCTTACGAGCCGTGGCGCACATTCTTTCCGTGCTGTCTCGGTTCCCGAGTTGACCCAGCAGATGTTCGACCCCAAGAACATGATGGCTGCTTCCGACTTCCGCAACGGTCGTTACCTGACCTGCTCTGCCATCTT

>*Cercophora_scortea*_GJS_L556

???????????????????????????????????????????????????????????????????????????????????????????????????????????????????????????????????????????????????????????????????????????????????????????????????????????????????????????????????????????????????????????????????????????????????????????????????????????????????????????????????????????????????????????????????????????????????????????????????????????????????????????????????????????????????????????????????????????????????????????????????????????????????????AAAGAAACCAACAGGG-ATTGCCC-CAGTAACGGCG-AGTGAAGCGGCAACAGCTCAAATTTGAAATCTGGCTTC--GG-CCCGAGTTGTAATTTGTAGAGG-AAGATTTTGGTGAGGCACCTTCTGAGTCCCCTGGAACGGGGCGCCATAGAGGGTGAGAGCCCCGTATAGTTGGATGCCTA-GCCTGTGTAAATCTCCTTCGACGAGTCGAGTAGTTTGGGAATGCTGCTCTAAATGGGAGGTAAATTTCTTCTAAAGCTAAATATTGGCCAGAGACCGATAGCGCACAAGTAGAGTGATCGAAAGATGAAAAGCACTTTGAAAAGAGGGTTAAACAGCACGTGAAATTGTTGAAAGGGAAGCGCTTGTGACCAGACTTGCGCCGGGCGGATCATCCGGTGTTCTCACCGGTGCACTCCGCCCGGCTCAGGCCAGCATCGGTTTTCGTGGGGGGATAAAGGCCCGGGGAACGTAGCTCC--TCCGGGAGTG-TTATAGCCCTGGGCGCAACGCCCTCGCGGGGACCGAGGTTCGCGCTTCTGCAAGGATGCTGGCGTAATGGTCATCAGCGACCCGTCTTGAAACACGGACCAAGGAGTCAAGGTTTTGCGCGAGTGTTTGGGTGTAAAACCCGCACGCGTAATGAAAGTGAACGTAGGTGAGAG--CTTCGGCGCATCATCGACCGATCCTGATGTATTCGGATGGATTTGAGTAGGAGCGTTAAGCCTTGGACCCGAAAGATGGTGAACTATGCTTGGATAGGGTGAAGCCAGAGGAAACTCTGGTGGAGGCTCGCAGCGGTT-CTGACGTGCAAATCGATCGTCAAATCTGAGCAT-GGGGGCGAAAGACTAATCGAACCATCTAGTAGCTGGTTACCGCCGCCCTGAAATACTCGTTGGCGACAGGCAACTGGGGTGACCAGAAAAAGGCAATGAGCTCGACCGCTGGTGTCTCCCAGGTGTTGAATCGATACACCTTCGCATCCACACTCTCTCATTTGAGACGAACCAACACCCCTATTGGCCGCGATGGCAAATTGGCCAAGCCACGGCAGCTGCACAACACACATTGGGGCTTGGTGTGCCCTGCAGAGACCCCCGAGGGACAGGCCTGTGGTCTTGTGAAGAACCTTTCGCTCATGTGCTTCGTCAGTGTGGGCACACCTGCCGACCCGATTGTGGAGTTCATGATTGCAAGAAATATGGAAGTGCTGGAAGAGTACGAGCCTCTGCGGTACCCCAACGCCACCAAGGTTTTCGTGAACGGTACCTGGGTCGGCGTGCACCAAGACCCCAAGCACCTCGTTAGCTTGGTCCAGGGGTTGCGCAGAAAGAACATCATCTCGTTCGAAGTGTCACTAGTACGGGACATTCGCGACAGAGAGTTCAAGATCTTTTCTGATGCTGGGCGAGTCATGAGACCGCTGTTTGTCGTGGAGCAAGAGGAAAACAGCGAC---------AGCGGTGTCGCGAAGGGACAGCTTGTTCTGACCAAGGACCACATCCGCCAGCTAGAGACCGACAAAGAGCTTGGCAAATACC------------ACGAGCAGTATTGGGGCTGGAAGGGATTGAGAGAATCTGGCGCCATCGAATACCTCGACGCCGAAGAGGAGGAAACAACCATGATCTGCATGACACCGGACGATTTGGATAATTACCGCATAACAAAGCT------------CATGGGCGGGGCTGCCGAAGAGGACGACAGAACTGGTAATGCCAGGATCAAGACGAAGATGAATCCCACAACACACATGTACACGCATTGCGAGATCCACCCCAGCATGCTGCTTGGTATTTGCGCGAGCATCATTCCCTTCCCCGACCACAACCAAGCTTGTTGATCAAGTTCTCGACGTCGTCCGCCGCGAGGCTGAGGGCTGCGACTGCCTCCAGGGCTTCCAGATCACCCACTCTCTCGGTGGTGGTACCGGTGCCGGTATGGGAACACTCTTGATCTCCAAGATCCGCGAGGAGTTCCCCGACCGCATGATGGCTACCTTCTCGGTTGTGCCCTCGCCCAAGGTCTCCGACACCGTTGTCGAGCCCTACAACGCCACCCTCTCGGTCCATCAGCTCGTTGAGAATTCGGACGAGACCTTCTGCATTGACAACGAGGCTCTGTACGACATCTGCATGCGTACCCTCAAGCTGTCCAACCCCTCGTACGGCGACCTCAACCACCTGGTTTCCGCCGTCATGTCGGGTGTCACCGTGTCTCTGCGCTTCCCCGGCCAGCTCAACTCTGATCTCCGCAAGCTGGCTGTCAACATGGTTCCCTTCCCCCGTCTCCATTTCTTCATGGTCGGATTTGCCCCTCTTACGAGCCGTGGCGCACATTCTTTCCGTGCTGTCTCGGTTCCCGAGTTGACCCAGCAGATGTTCGACCCCAAGAACATGATGGCTGCTTCTGACTTCCGCAACGGCCGTTACCTGACCTGCTCTGCCATCTT

>*Bombardia_bombarda*_SMH3391

???????????????????????????????????????????????????????????????????????????????????????????????????????????????????????????????????????????????????????????????????????????????????????????????????????????????????????????????????????????????????????????????????????????????????????????????????????????????????????????????????????????????????????????????????????????????????????????????????????????????????????????????????????????????????????????????????????????????????????????????????????????????????????AAAGAAACCAACAGGG-ATTGCCC-CAGTAACGGCG-AGTGAAGCGGCAACAGCTCAAATTTGAAATCTGGCTCC--GG-CCCGAGTTGTAATTTGTAGAGG-AAGCTTTTGGTGAGGCACTTTCTGAGTCCCCTGGAACGGGGCGCCATAGAGGGTGAGAGCCCCGTATAGGTAGATGCCGA-GCCTCTGTAAAGCTCCTTCGACGAGTCGAGTAGTTTGGGAATGCTGCTCAAAATGGGAGGTAAATTTCTTCTAAAGCTAAATATTGGCCAGAGACCGATAGCGCACAAGTAGAGTGATCGAAAGATGAAAAGCACTTTGAAAAGAGGGTTAAACAGCACGTGAAATTGTTGAAAGGGAAGCGCTTGTGACCAGACTTGCGTCGGGCTGATCATCCGGTGTTCTCACCGGTGCACTCTGCCCGGCTCAGGCCAGCATCGGTTTTCGCGGGGGGATAAAGGCCCGGGGAACGTAGCTCC--TCCGGGAGTG-TTATAGCCCTGGGCGCAACGCCCCCGCGGGGACCGAGGTTCGCGCATCTGCAAGGATGCTGGCGTAATGGTCACCAGCGACCCGTCTTGAAACACGGACCAAGGAGTCAAGGTTTTGCGCGAGTGTTTGGGTGTAAAACCCGCACGCGTAATGAAAGTGAACGTAGGTGAGAG--CTTCGGCGCATCATCGACCGATCCTGATGTTTTCGGATGGATTTGAGTAGGAGCGTTAAGCCTTGGACCCGAAAGATGGTGAACTATGCTTGGATAGGGTGAAGCCAGAGGAAACTCTGGTGGAGGCTCGCAGCGGTT-CTGACGTGCAAATCGATCGTCAAATCTGAGCAT-GGGGGCGAAAGACTAATCGAACCATCTAGTAGCTGGTTACCGCCGCCCTAAAGTACTCGCTCGCGACGGGTAATTGGGGTGATCAGAAGAAGGCGGCAAGCTCCACAGCTGGTGTGTCTCAGGTGCTCAACCGATACACGTTTGCGTCTACTCTTTCTCATTTGAGACGGACGAACACCCCTATTGGGCGCGATGGCAAACTTGCCAAGCCTCGCCAGCTTCATAACACACATTGGGGCCTGGTGTGTCCAGCAGAGACGCCAGAGGGACAGGCTTGTGGACTGGTCAAGAATCTCTCGCTGATGTGCTTCGTGAGTGTGGGCACTCCCGCAGAGCCTGTCATCGAGTTCATGATCAACCGGAATATGGAGGTGTTGGAAGAGTACGAGCCATTGCGCTATCCCAACGCGACCAAGGTGTTTGTCAACGGTACATGGGTTGGCATTCACCACGAACCCAAGCAGCTTGTCAGCCTGGTTCAAGGCCTCCGACGCTCGGGCATCATTTCTTACGAGGTCTCTCTTGTTCGCGATATTCGCGACAGAGAGTTCAAGATCTTTTCCGACGCTGGTCGCGTTATGAGGCCACTATTTGTCGTGGAGCAAGACGAGAATGCCAGG---------AGTGGGGTCGAAAAGGGACAGTTGATCCTCAACAAGACACATATCCGGCGACTGGAGCAAGATAAAGAGTTGGGCAAGTACC------------ATCCGCAGTATTGGGGTTGGAAGGGTCTTCGTGATACAGGTGCTATTGAGTACCTGGATGCCGAAGAGGAGGAAACGGCCATGATCTGTATGTCACCCGAAGACCTAGACTCCTATCGATTGGCCAAGAT------------GGGTATTGCAGTTGATGAGGAGGATGGGGGGGGTGCGAATGCTCGCATCAGGACCAAGACCAACCCGACAACTCACATGTACACCCACTGTGAAATTCATCCCAGCATGCTGCTCGGTATCTGCGCCAGCATTATCCCCTTCCCCGACCACAACCAGGCTCGTCGACCAGGTCCTCGACGTCGTCCGTCGCGAGGCCGAGGGCTGCGACTGCCTCCAGGGTTTCCAGATCACCCACTCCCTCGGTGGTGGTACCGGTGCCGGTATGGGTACACTCCTGATCTCCAAGATCCGCGAGGAGTTCCCCGACCGCATGATGGCCACCTTCTCCGTTGTGCCTTCCCCCAAGGTCTCGGATACCGTTGTTGAGCCCTACAATGCCACCCTGTCGATCCACCAGCTTGTCGAGAACTCTGACGAGACCTTCTGCATCGACAACGAGGCTCTGTACGATATCTGCTTGCGCACCCTTAAGCTGTCCAACCCTTCGTACGGCGACCTTAACCACCTCGTTTCGGCCGTTATGTCCGGCGTTACCGTCTCGCTGCGTTTCCCTGGCCAGCTCAACTCGGATCTCCGCAAGCTCGCGGTCAACATGGTCCCCTTCCCGCGTCTCCATTTCTTCATGGTTGGATTCGCTCCTCTCACGAGCCGCGGCGCGTACTCTTTCCGTGCCGTCTCGGTTCCTGAGTTGACCCAGCAGATGTTTGACCCCAAGAACATGATGGCTGCTTCTGACTTCCGTAACGGTCGTTACCTGACCTGCTCTGCCATCTT

>*Bombardioidea_anartia*

???????????????????????????????????????????????????????????????????????????????????????????????????????????????????????????????????????????????????????????????????????????????????????????????????????????????????????????????????????????????????????????????????????????????????????????????????????????????????????????????????????????????????????????????????????????????????????????????????????????????????????????????????????????????????????????????????????????????????????????????????????????????????????AAAGAAACCAACAGGG-ATTGCCC-CAGTAACGGCG-AGTGAAGCGGCAACAGCTCAAATTTGAAATCTGGCTCC--GG-CCCGAGTTGTAATTTGTAGAGG-AAGCTTTTGGTGAGGCACTTTCTGAGTCCCCTGGAACGGGGCGCCATAGAGGGTGAGAGCCCCGTATAGTTAGATGCCGA-GCCTCTGTAAAGCTCCTTCGACGAGTCGAGTAGTTTGGGAATGCTGCTCAAAATGGGAGGTAAATTTCTTCTAAAGCTAAATATTGGCCAGAGACCGATAGCGCACAAGTAGAGTGATCGAAAGATGAAAAGCACTTTGAAAAGAGGGTTAAACAGCACGTGAAATTGTTGAAAGGGAAGCGCTTGTGACCAGACTTGCGTCGGGCTGATCATCCGGTGTTCTCACCGGTGCACTCTGCCCGGCTCAGGCCAGCATCGGTTTTCGTGGGGGGATAAAGGCCCGGGGAACGTAGCTCC--TCCGGGAGTG-TTATAGCCCTGGGCGCAACGCCCCCGCGGGGACCGAGGTTCGCGCATCTGCAAGGATGCTGGCGTAATGGTCACCAGCGACCCGTCTTGAAACACGGACCAAGGAGTCAAGGTTTTGCGCGAGTGTTTGGGTGTAAAACCCGCACGCGTAATGAAAGTGAACGTAGGTGAGAG--CTTCGGCGCATCATCGACCGATCCTGATGTTTTCGGATGGATTTGAGTAGGAGCGTTAAGCCTTGGACCCGAAAGATGGTGAACTATGCTTGGATAGGGTGAAGCCAGAGGAAACTCTGGTGGAGGCTCGCAGCGGTT-CTGACGTGCAAATCGATCGTCAAATCTGAGCAT-GGGGGCGAAAGACTAATCGAACCATCTAGTAGCTGGTTACCGCCGCTCTAAAGTACTCGCTGGCCACGGGTAACTGGGGTGATCAGAAGAAAGCGGCAAGCTCCACAGCTGGTGTGTCTCAGGTGCTCAACCGATACACATTTGCATCTACGCTTTCTCATTTGAGACGAACAAACACGCCTATTGGACGCGATGGCAAACTTGCCAAGCCTCGCCAGCTTCACAACACACATTGGGGCTTGGTCTGTCCAGCAGAGACGCCAGAGGGACAGGCCTGTGGACTGGTCAAGAATCTGTCGCTGATGTGCTTCGTCAGTGTGGGCACTCCCGCAGAGCCTGTCATTGAGTTCATGATCAATAGAAACATGGAGGTGTTGGAAGAGTATGAGCCATTGCGTTATCCCAACGCGACCAAGGTGTTTGTCAACGGTACATGGGTCGGCATTCACCACGAGCCCAAGCAGCTTGTCAGCCTGGTTCAAGGTCTCCGACGCTCGGGCGTCATTTCCTACGAGGTCTCTCTTGTTCGCGATATTCGCGACAGAGAGTTCAAGATCTTTTCCGACGCTGGTCGCGTTATGAGGCCACTATTTGTGGTGGAGCAAGACGAGAATGCCAGG---------AGTGGTGTCGAAAAGGGGCAGTTGATTCTCAACAAGACACATATCCGACGACTGGAGCAAGACAAAGAGTTGGGCAAGTACC------------ATCCGCAGTACTGGGGTTGGAAGGGTCTTCGTGACACAGGTGCTATTGAATACCTGGATGCCGAGGAGGAGGAAACGGCCATGATCTGCATGTCACCAGAAGACCTGGATACCTACCGACTGGCAAAGAT------------GGGTATACCGGTTGGTGAGGAGGA---GGGAGGTGCAAATGCTCGTATCAGGACTAAGACGAACCCGACAACTCACATGTACACCCACTGTGAAATCCATCCGAGCATGCTGCTCGGTATCTGCGCCAGCATTATCCCCTTCCCCGACCACAACCAGGCTCGTCGACCAGGTCCTCGACGTCGTCCGTCGCGAGGCCGAGGGCTGCGACTGCCTCCAGGGTTTCCAGATCACCCACTCCCTCGGTGGTGGTACCGGTGCCGGTATGGGCACACTCCTGATCTCCAAGATCCGCGAGGAGTTCCCCGACCGCATGATGGCCACCTTCTCGGTTGTGCCTTCCCCTAAGGTGTCGGATACCGTTGTTGAGCCCTACAACGCCACCCTGTCGATCCACCAGCTTGTCGAGAACTCTGACGAGACCTTCTGCATCGACAACGAGGCTCTCTACGATATCTGCATGCGCACTCTCAAGCTGTCCAACCCTTCGTACGGCGATCTTAACCACCTCGTGTCGGCCGTCATGTCTGGCGTTACCGTCTCGCTCCGTTTCCCTGGCCAGCTCAACTCGGATCTCCGAAAGCTCGCCGTCAACATGGTGCCCTTCCCGCGTCTTCATTTCTTCATGGTTGGATTCGCTCCTCTCACGAGCCGCGGCGCGTACTCTTTCCGTGCCGTCTCGGTTCCTGAGTTGACCCAGCAGATGTTCGACCCCAAGAACATGATGGCTGCTTCCGACTTCCGTAACGGTCGTTACCTTACCTGCTCTGCCATCTT

>*Apodospora_simulans*_Kruys_701

????????????????????????????????????????????????????????????????????????????????????????????????????????????????????????????????????????????????????????????????????????????????????????????????????????????????????????????????????????????????????????????????????????????????????????????????????????????????????????????????????????????????????????????????????????????????????????????????????????????????????????????????????????????????????????????????????????????????????????????????????????????????????????????????????????ATTGCCC-CAGTAACGGCG-AGTGAAGCGGCAACAGCTCAAATTTGAAATCTGGCTTC--GG-CCCGAGTTGTAATTTGTAGAGG-AAGCTTTTGGCGAGGTGCCTTCTGAGTCCCCTGGAACGGGGCGCCATAGAGGGTGAGAGCCCCGTATAGTTGGACACCTA-GCCTCTGTAAAGCTCCTTCGACGAGTCGAGTAGTTTGGGAATGCTGCTCAAAATGGGAGGTAAATTTCTTCTAAAGCTAAATATTGGCCAGAGACCGATAGCGCACAAGTAGAGTGATCGAAAGATGAAAAGCACTTTGAAAAGAGGGTTAAACAGCACGTGAAATTGTTGAAAGGGAAGCGCTTGTGACCAGACATGCGCCGGGCGGATCATCCGGTGTTCTCACCGGTGCACTCCGCCCGGCTCAGGCCAGCATCGGTTCTCGCGGGGGGATAAAGGCCCGGGGAACGTAGCTCC--TCCGGGAGTG-TTATAGCCCTGGGCGCAACGCCCTCGCGGGGACCGAGGTTCGCGCATCTGCAAGGATGCTGGCGTAATGGTCATCAGCGACCCGTCTTGAAACACGGACCAAGGAGTCAAGGTTTTGCGCGAGTGTTTGGGTGTTAAACCCGCACGCGTAATGAAAGTGAACGTAGGTGAGAG--CTTCGGCGCATCATCGACCGATCCTGATGTATTCGGATGGATTTGAGTAGGAGCGTTAAGCCTTGGACCCGAAAGATGGTGAACTATGCTTGGATAGGGTGAAGCCAGAGGAAACTCTGGTGGAGGCTCGCAGCGGTT-CTGACGTGCAAATCGATCGTCAAATCTGAGCAT-GGGGGCGAAAGACTAATCGAACCATCTAGTAGCTGGTTACCGCC??????????????????????????????????????????????????????????????????????????????????????????????????????????????????????????????????????????????????????????????????????????????????????????????????????????????????????????????????????????????????????????????????????????????????????????????????????????????????????????????????????????????????????????????????????????????????????????????????????????????????????????????????????????????????????????????????????????????????????????????????????????????????????????????????????????????????????????????????????????????????????????????????????????????????????????????????????????????????????????????????????????????????????????????????????????????????????????????????????????????????????????????????????????????????????????????????????????????????????????????????????????????????????????????????????????????????????????????????????????????????????????????????????????????????????????????????????????????????????????????????????????????????GCTTGTTGACCAAGTTCTTGATGTCGTCCGTCGCGAGGCCGAGGGCTGCGACTGCCTCCAGGGCTTCCAAATCACCCACTCGCTCGGTGGTGGTACTGGTGCCGGTATGGGTACCCTCTTGATCTCCAAGATCCGCGAGGAGTTCCCGGACCGCATGATGGCTACCTTCTCCGTCGTGCCCTCCCCCAAGGTCTCGGACACCGTTGTCGAGCCCTACAACGCAACTCTCTCCGTGCACCAGCTCGTTGAGAACTCTGACGAGACCTTCTGCATCGACAACGAGGCTCTCTACGACATCTGCATGCGGACTCTCAAGCTGTCCAACCCCTCGTATGGCGACCTGAACCACCTGGTCTCGGCTGTCATGTCCGGCGTCACCGTCTCGCTGCGTTTCCCCGGCCAGCTCAACTCCGATCTCCGCAAGCTTGCTGTCAACATGGTTCCCTTCCCGCGTCTGCACTTCTTCATGGTCGGATTTGCGCCGCTTACGAGCCGTGGTGCTCACTCTTTCCGTGCTGTCTCGGTTCCCGAGCTGACCCAGCAGATGTTCGACCCCAAGAACATGATGGCTGCTTCTGACTTCCGCAACGGTCGCTACCTAACCTGCTCTGCCATCTT

>*Apodus_deciduus*_CBS_506.70

ATTACAGAGTT-------GCAAAACTCCC--AACCCT-TTGTGAACG-AACCTAC----CAG-TTGCTTCGGCGTGGCATCTCTGAGTAG------CTTATAAATAAGTTAAAACTTTCAACAACGGATCTCTTGGTTCTGGCATCGATGAAGAACGCAGCGAAATGCGATAAGTAATGTGAATTGCAGAATTCAGTGAATCATCGAATCTTTGAACGCACATTGCGCCCGCTAGTATTCTGGCGGGCATGCCTGTTCGAGCGTCATTTCAA---CCATCAAG-CCCTA----GGCTTGTGTTGGGG-CCCTGCG-GCC-GTC-CGCAGCCCCC-TAAAAACAGTGGCGGGCTCGCT-AT-AT-ACCGAGTGCAGTAGTTTACTCTTCGCTCAGGACAT-GTAGTGGG-TTCTTGCCGTAAAACCCCCCAT-----TTTTTAAGGTTGACCTCGGATCAGGTAGGAATACCCGCTGAACTTAAGCATATCAATAA????????????????CAACAGGG-ATTGCCC-CAGTAACGGCG-AGTGAAGCGGCAACAGCTCAAATTTGAAATCTGGCTCC--GG-CCCGAGTTGTAATTTGCAGAGG-AAGCTTCTGGTGATATACTGTCTAAGTCCCCTGGAACGGGGCGCCACAGTGGGTGAGAGCCCCATATGACAGATGTAGAT--CCTGTGTGAAGCTCCTTCGACGAGTCGAGTAGTTTGGGAATGCTGCTCTAAATGGGAGGTAAATTCCTTCTAAAGCTAAATATTGGCCAGAGACCGATAGCGCACAAGTAGAGTGATCGAAAGATGAAAAGCACTTTGAAAAGAGGGTTAAACAGCACGTGAAATTGTTGAAAGGGAAGCGCTTATGACCAGACTTGCGCTGGGCTGATCATCCGGTGTTCTCACCGGTGCACTCGGCCCAGCTCAGGCCAGCATCGGTTTTGGTGGGGGGATAAAGGCGTTGGGAACGTAGCTCC--TTCGGGAGTG-TTATAGCCCAGCGTGCAATACCCCCGCTGGGACCGAGGTTCGCGCATCTGCAAGGATGCTGGCGTAATGGTCATCAGCGACCCGTCTTGAAACACGGACCAAGGAGTCAAGGTTTTGCGCGAGTGTTTGGGTGTCAAACCCGCACGCGTAATGAAAGTGAACGTAGGTGAGAG--CTTCGGCGCATCATCGACCGATCCTGATGTATTCGGATGGATTTGAGTAGGAGCGTTAAGCCTTGGACCCGAAAGATGGTGAACTATGCTTGGATAGGGTGAAGCCAGAGGAAACTCTGGTGGAGGCTCGCAGCGGTT-CTGACGTGCAAATCGATCGTCAAATCTGAGCAT-GGGGGCGAAAGACTAATCGA????????????????????????????????????????????????????????????????????????????????????????????????????????????????????????????????????????????????????????????????????????????????????????????????????????????????????????????????????????????????????????????????????????????????????????????????????????????????????????????????????????????????????????????????????????????????????????????????????????????????????????????????????????????????????????????????????????????????????????????????????????????????????????????????????????????????????????????????????????????????????????????????????????????????????????????????????????????????????????????????????????????????????????????????????????????????????????????????????????????????????????????????????????????????????????????????????????????????????????????????????????????????????????????????????????????????????????????????????????????????????????????????????????????????????????????????????????????????????????????????????????????????????????????????????????????????????????????????????????????????????????????????????????????????????????????????????????????????????????????????????????????????????????????????????????????????????????????????????????????????????????????????????????????????????????????????????????????????????????????????????????????????????????????????????????????????????????????????????????????????????????????????????????????????????????????????????????????????????????????????????????????????????????????????????????????????????????????????????????????????????????????????????????????????????????????????????????????????????????????????????????????????????

>*Arnium_cirriferum*_CBS_120041

???????????????????????????????????????????????????????????????????????????????????????????????????????????????????????????????????????????????????????????????????????????????????????????????????????????????????????????????????????????????????????????????????????????????????????????????????????????????????????????????????????????????????????????????????????????????????????????????????????????????????????????????????????????????????????????????????????????????????????????????????????????????????????AAAGAAACCAACAGGG-ATTGCCC-TAGTAACGGCG-AGTGAAGCGGCAACAGCTCAAATTTGAAATCTGGCTCC--GG-CCCGAGTTGTAATTTGCAGAGG-AAGCTTCTGGTGATGCGCTGTCTAAGTCCCCTGGAACGGGGCGCCACAGTGGGTGAGAGCCCCATTTGACAGAGGCAGAT--CCTGTGTGAAGCTCCTTCGACGAGTCGAGTAGTTTGGGAATGCTGCTCAAAATGGGAGGTAAATTCCTTCTAAAGCTAAATATTGGCCAGAGACCGATAGCGCACAAGTAGAGTGATCGAAAGATGAAAAGCACTTTGAAAAGAGGGTTAAACAGCACGTGAAATTGTTGAAAGGGAAGCGCTTGTGACCAGACTTGCGCTGGGCTGATCATCCGGTGTTCTCACCGGTGCACTCGGCCCAGCTCAGGCCAGCATCGGTTTTGGCGGGGGGATAAAGGCGTTAGGAACGTAGCTCC--CTAGGGAGTG-TTATAGCCCAGCGTGCAATGCCCCCGCTGGGACCGAGGTTCGCGCATCTGCAAGGATGCTGGCGTAATGGTCATCAGCGACCCGTCTTGAAACACGGACCAAGGAGTCAAGGTTTTGCGCGAGTGTTTGGGTGTCAAACCCGCACGCGTAATGAAAGTGAACGTAGGTGAGAG--CTTCGGCGCATCATCGACCGATCCTGATGTATTCGGATGGATTTGAGTAGGAGCGTTAAGCCTTGGACCCGAAAGATGGTGAACTATGCTTGGATAGGGTGAAGCCAGAGGAAACTCTGGTGGAGGCTCGCAGCGGTT-CTGACGTGCAAATCGATCGTCAAATCTGAGCAT-GGGGGCGAAAGACTAATCGAACCATCTAGTAGCTGGTTACCGCC??????????????????????????????????????????????????????????????????????????????????????????????????????????????????????????????????????????????????????????????????????????????????????????????????????????????????????????????????????????????????????????????????????????????????????????????????????????????????????????????????????????????????????????????????????????????????????????????????????????????????????????????????????????????????????????????????????????????????????????????????????????????????????????????????????????????????????????????????????????????????????????????????????????????????????????????????????????????????????????????????????????????????????????????????????????????????????????????????????????????????????????????????????????????????????????????????????????????????????????????????????????????????????????????????????????????????????????????????????????????????????????????????????????????????????????????????????????????????????????????????????????????????GCTTGTTGACCAAGTTCTCGATGTCGTTCGTCGCGAGGCCGAGGGCTGCGACTGCCTCCAGGGCTTCCAGATCACCCACTCCCTCGGTGGTGGTACCGGTGCTGGTATGGGTACCCTCCTTATCTCCAAGATTCGCGAGGAGTTCCCCGACCGCATGATGGCGACCTTCTCTGTCGTCCCCTCCCCCAAGGTCTCTGACACCGTCGTCGAGCCCTACAACGCCACCCTGTCGGTGCACCAGCTTGTCGAGAACTCGGACGAGACCTTCTGCATTGACAACGAGGCTCTCTACGACATCTGCATGCGGACGCTCAAGCTGTCCAACCCCTCGTACGGCGACCTCAACCACCTAGTCTCCGCCGTCATGTCGGGTGTCACCGTCTCTCTCCGTTTCCCCGGCCAGCTCAACTCCGATCTCCGCAAGCTCGCCGTCAACATGGTTCCCTTCCCTCGTCTTCACTTCTTCATGGTCGGCTTCGCGCCCCTTACTAGCCGTGGCGCGCACTCTTTCCGTGCTGTCTCGGTTCCTGAGCTCACCCAGCAGATGTTCGACCCCAAGAACATGATGGCCGCCTCTGACTTCCGCAACGGTCGCTACCTCACCTGCTCTGCCATCTT

>*Apiosordaria_microcarpa*_CBS_692.82

ATTACAGAGTT-------GCAAAACTCCC--AACCCT-TTGTGAACG-TACCTA-----ACA-GTGCTTCGGCGTGGCATCTCTGAGCAA------CTATAAAATAAGTTAAAACTTTCAACAACGGATCTCTTGGTTCTGGCATCGATGAAGAACGCAGCGAAATGCGATAAGTAATGTGAATTGCAGAATTCAGTGAATCATCGAATCTTTGAACGCACATTGCGCCCGCTAGTATTCTGGCGGGCATGCCTGTTCGAGCGTCATTTCAA---CCATCAAG-CCTCA----GGCTTGTGTTGGAG-CCCTGCG-GCT-GCC-CGCAGCCTCC-TAAAAGCAGTGGCGGGCTCGCT-ATCAC-ACCGAGTGCAGTAGTTTACTCTTCGCTCAGGGCGT-GTGGCGGG-TTCTAGCCGTAAAACCCCCTAC-----TTTTAAAGGTTGACCTCGGATCAGGTAGGAATACCCGCTGAACTTAAGCATATCAATAAGCGGAGGAAAAGAAACCAACAGGG-ATTGCCC-TAGTAACGGCG-AGTGAAGCGGCAACAGCTCAAATTTGAAATCTGGCTCC--GG-CCCGAGTTGTAATTTGCAGAGG-AAGCTTCTGGTGATATACCGTCTAAGTCCCCTGGAACGGGGCGCCACAGTGGGTGAGAGCCCCATATGACGGATGTAGAT--CCTGTGTGAAGCTCCTTCGACGAGTCGAGTAGTTTGGGAATGCTGCTCAAAATGGGAGGTAAATTCCTTCTAAAGCTAAATATTGGCCAGAGACCGATAGCGCACAAGTAGAGTGATCGAAAGATGAAAAGCACTTTGAAAAGAGGGTTAAACAGCACGTGAAATTGTTGAAAGGGAAGCGCTTATGACCAGACTTGCGCTGGGCTGATCATCCGGTGTTCTCACCGGTGCACTCGGCCCAGCTCAGGCCAGCATCGGTTTTGGTGGGGGGATAAAGGCGCTGGGAACGTAGCTCC--TTCGGGAGTG-TTATAGCCCAGCGTGCAATGCCCCCGCCGGGACCGAGGTTCGCGCATCTGCAAGGATG????????????????????????????????????????????????????????????????????????????????????????????????????????????????????????????????????????????????????????????????????????????????????????????????????????????????????????????????????????????????????????????????????????????????????????????????????????????????????????????????????????????????????????????????????????????????????????????????????????????????????????????????????????TACACCTTTGCCTCGACGCTCTCCCATTTGCGGCGAACAAACACGCCCATCGGTCGCGATGGCAAGTTGGCCAAACCCCGGCAGCTGCACAATACACACTGGGGCCTTGTTTGCCCTGCCGAAACCCCAGAAGGCCAGGCTTGTGGCCTCGTCAAGAACCTCTCGTTAATGTGCTATATCAGCGTGGGCACGAATGCTGAGCCTATCATCGACTTTATGGTGGCCAGGAACATGGAAGTCCTTGAAGAGTACGAGCCGCTCCGCTACCCTAACGCCACCAAGGTCTTTGTCAACGGGACTTGGGTTGGTGTCCACCAAGACCCGAAGCACTTGGTCACATTGGTTCAGAATCTTAGGAGATCCAACGTTATCTCCTTCGAGGTTTCGCTTGTTCGCGACATCCGAGATCGAGAGTTCAAGATCTTCTCTGATGCTGGCCGAGTCATGAGACCGCTCTTCGTTGTTGAGCAGGAAGACGACAACAAG------TTGACCAAGGTCCAGAAGGGCCAGCTAGTCCTGACAAGAGAGCATATGAACCGGCTAGACCGGGACAAGGATATCGGACCAATGG------------ACGAAGACTTCTTCGGCTGGAACGGCCTTCTGAGGGAGGGTTGTGTTGAGTACCTCGATGCCGAGGAAGAGGAGACAGCCATGATTTGCATGACCCCCGACGACCTGGAACACTATCGGTTCACCAGGATGGG---GGCAAAGACCAGACGGAGGAGCAGCCGGGAGACGATGGAGTATAACAAACGCATCACAACGAAGACGAGTCCGACCACGCAAATGTATACCCATTGCGAGATCCATCCCAGCATGTTACTCGGTATTTGCGCCAGCATTATCCCCTTCCCG??????????????????????????????????????????????????????????????????????????????????????????????????????????????????????????????????????????????????????????????????????????????????????????????????????????????????????????????????????????????????????????????????????????????????????????????????????????????????????????????????????????????????????????????????????????????????????????????????????????????????????????????????????????????????????????????????????????????????????????????????????????????????????????????????????????????????????????????????????????????????????????????????????????????????????????????????????????????????????

>*Cercophora_fici*_MFLU_19-2735

ATTAGCGA----------GTATAACTCTCTAAAACCA-TTGTGAACG-AACGAAC----CCG-TTGCTTCGGCGTTGTTTCTCTGAGTA------CTTATTCAATAAGTAAAAACTTTCAACAACGGATCTCTTGGTTCTGGCATCGATGAAGAACGCAGCGAAATGCGATAAGTAATGTGAATTGCAGAATTCAGTGAATCATCGAATCTTTGAACGCACATTGCGCCCGCCAGTATTCTGGCGGGCATGCCTGTTCGAGCGTCATTTCAA---CCATCAAG-CCCTA----GGCTTGTGTTGGGG-ACCTGCA-GCT-GCT--GCAGCCCCC-TAAAAGCAGTGGCGGACTCGCT-GTCAT-TCCGAGCGCAGTAGTT-ACATCTCGCTCTGGGTATGGCGGCGTG-CACTTGCCGTGAAACACTTT----------CTAAGGTTGACCTCGGATCAGGTAGGAATACCCGCTGAACTTAAGCATATCAATAAGCGGAGGAAAAGAAACCAACAGGG-ATTGCCC-TAGTAACGGCG-AGTGAAGCGGCAACAGCTCAAATTTGAAATCTGGCTTC--GG-CCCGAGTTGTAATTTGTAGAGG-AAGCTTCTGGCGCGGTGCCATCCGAGTCCCCTGGAACGGGGCGCCATAGAGGGTGAGAGCCCCGTATGGATGGATGCCTA-GCCTGTGTGAAGCTCCTTCGACGAGTCGAGTAGTTTGGGAATGCTGCTCAAAATGGGAGGTAAATTCCTTCTAAAGCTAAATATTGGCCAGAGACCGATAGCGCACAAGTAGAGTGATCGAAAGATGAAAAGCACTTTGAAAAGAGGGTTAAACAGCACGTGAAATTGTTGAAAGGGAAGCGCTTGTGACCAGACTTGCGCCAGGTTGATCATCCGGTGTTCTCACCGGTGCACTCTGCCTGGCACAGGCCAGCATCAGTTTTGGCGGGGGGATAAAGGGCGCAGGAACGTAGCTCC--TCCGGGAGTG-TTATAGCCTGTGTCGTAATGCCCTCGCCGGGACTGAGGACCGCGCATCTGCAAGGATGCTGGCGTAATGGTCATCAGCGACCCGTCTTGAAACACGGACCAAGGAGTCAAGGTTTTGCGCGAGTGTTTGGGTGTAAAACCCGCACGCGTAATGAAAGTGAACGTAGGTGAGAG--CTTCGGCGCATCATCGACCGATCCTGATGTATTCGGATGGATTTGAGTAAGAGCGTTAAGCCTTGGACCCGAAAGATGGTGAACTATGCTTGGATAGGGTGAAGCCAGAGGAAACTCTGGTGGAGGCTCGCAGCGGTT-CTGACGTGCAAATCGATCGTCAAATCTGAGCAT-GGGGGCGAAAGACTAATCGAA???????????????????????????????????????????????????????????????????????????????????????????????????????????????????????????????????????????????????????????????????????????????????????????????????????????????????????????????????????????????????????????????????????????????????????????????????????????????????????????????????????????????????????????????????????????????????????????????????????????????????????????????????????????????????????????????????????????????????????????????????????????????????????????????????????????????????????????????????????????????????????????????????????????????????????????????????????????????????????????????????????????????????????????????????????????????????????????????????????????????????????????????????????????????????????????????????????????????????????????????????????????????????????????????????????????????????????????????????????????????????????????????????????????????????????????????????????????????????????????????????????????????????????????????????????????????????????????????????????????????????????????????????????????????????????????????????????????????????????????????????????????????????????????????????????????????????????????????????????????????????????????????????????????????????????????????????????????????????????????????????????????????????????????????????????????????????????????????????????????????????????????????????????????????????????????????????????????????????????????????????????????????????????????????????????????????????????????????????????????????????????????????????????????????????????????????????????????????????????????????????????????????????

>*Cercophora_newfieldiana*_SMH3303

?????????????????????????????????????????????????????????????????????????????????????????????????????????????????????????????????????????????????????????????????????????????????????????????????????????????????????????????????????????????????????????????????????????????????????????????????????????????????????????????????????????????????????????????????????????????????????????????????????????????????????????????????????????????????????????????????????????????????????????????????????????????????????????????????????GG-ATTGCCC-TAGTAACGGCG-AGTGAAGCGGCAACAGCTCAAATTTGAAATCTGGCTCC--GG-CCCGAGTTGTAATTTGCAGAGG-AAGCTTCTGGTGATATACTGTCTAAGTCCCCTGGAACGGGGCGCCACAGTGGGTGAGAGCCCCATATGACAGCTGTAGAT--CCTGTGTGAAGCTCCTTCGACGAGTCGAGTAGTTTGGGAATGCTGCTCTAAATGGGAGGTAAATTCCTTCTAAAGCTAAATATTGGCCAGAGACCGATAGCGCACAAGTAGAGTGATCGAAAGATGAAAAGCACTTTGAAAAGAGAGTTAAACAGCACGTGAAATTGTTGAAAGGGAAGCGCTTATGACCAGACTTGCGCTGGGCTAATCATCCGGTGTTCTCACCGGTGCACTTGGCCCAGCTCAGGCCAGCATCGGTTTTGGTGGGGGGATAAAGGCGCTGGGAACGTAGCTCT--TTCGGGAGTG-TTATAGCCCAGTGTGCAATGCCCCCGCTGGGACCGAGGTTCGCGCATTTGCAAGGATGCTGGCGTAATGGTCATCAGCGACCCGTCTTGAAACACGGACCAAGGAGTCAAGGTTTTGCGCGAGTGTTTGGGTGTCAAACCCGCACGCGTAATGAAAGTGAACGTAGGTGAGAG--CTTCGGCGCATCATCGACCGATCCTGATGTATTCGGATGGATTTGAGTAGGAGCGTTAAGCCTTGGACCCGAAAGATGGTGAACTATGCTTGGATAGGGTGAAGCCAGAGGAAACTCTGGTGGAGGCTCGCAGCGGTT-CTGACGTGCAAATCGATCGTCAAATCTGAGCAT-GGGGGCGAAAGACTAATCGAACCATCTAGTAGCTGGTTACCGCCGCCCTCAAGTACTCGCTGGCTACCGGCAACTGGGGTGATCAGAAGAAGGCGATGAGCTCTACGGCTGGTGTGTCGCAGGTGTTGAACCGATATACATTTGCCTCGACGCTCTCCCATTTGCGGCGAACAAACACACCTATCGGTCGTGACGGCAAGCTTGCCAAACCTCGACAGCTGCACAACACTCACTGGGGCCTCGTCTGCCCTGCCGAAACCCCAGAAGGCCAAGCTTGCGGCCTCGTCAAGAACCTCTCTCTGATGTGCTATATTAGCGTGGGCACTAATGCGGAACCTATCGTCGACTTTATGGTGGCTAGGAACATGGAAGTCCTCGAAGAGTACGAGCCGCTCCGCTATCCCAACGCCACGAAGGTCTTCGTCAACGGAACCTGGGTTGGCGTGCACCAAGACCCCAAGCATTTGGTGACCCTGGTCCAGAATCTCAGGAGGTCCAACATCATCTCCTTTGAAGTTTCGCTTGTCCGGGACATTCGAGATCGAGAGTTCAAGATCTTCTCCGACGCAGGCCGTGTCATGAGGCCGCTCTTTGTTGTTGAGCAAGAAGACGAGAACAAG------GTGACCAAGGTCCAGAAGGGCCAGTTGGTCTTGACAAGGTCGCACATCGACCGGCTGGATCGGGACAAGGAGCTCGGGCCATTGG------------ACGAAGGTTTCTTTGGTTGGAACGGCCTCCTCAGAGAGGGTTGTGTCGAGTATCTCGACGCCGAGGAAGAGGAGACGGCCATGATTTGCATGACGCCCGAAGACTTGGATCACTACCGATCTACCAAGCTGGGC---GTTAAGTCGAAAGCCCACCTAGACGATGAAGAGGAAGGGCACAACAAGCGCATCAAAACGAAGGCGAACCCGACCACTCATATGTACACTCATTGCGAGATCCATCCCAGTATGCTGCTTGGTATCTGCGCGAGCATTATCCCCTTCCCGGATCACAATCAGGCTTGTTGACCAAGTTCTCGATGTCGTCCGTCGTGAGGCCGAGGGCTGCGACTGCCTCCAGGGCTTCCAGATCACCCACTCGCTCGGTGGTGGTACCGGTGCCGGTATGGGTACCCTCCTTATCTCCAAGATTCGCGAGGAGTTCCCCGACCGCATGATGGCGACTTTTTCCGTCGTTCCCTCCCCCAAGGTCTCGGACACCGTCGTTGAGCCATACAACGCCACCCTGTCGGTTCACCAGCTTGTCGAGAACTCGGACGAGACTTTCTGCATTGACAACGAGGCTCTGTACGATATCTGCATGCGGACGCTGAAGCTTTCCAACCCCTCGTACGGCGACCTCAACCACCTGGTCTCCGCCGTCATGTCGGGTGTCACCGTTTCCCTGCGTTTCCCCGGCCAGCTCAACTCCGATCTTCGCAAGCTTGCCGTCAACATGGTTCCCTTCCCTCGTCTGCACTTCTTCATGGTCGGCTTCGCGCCTCTTACTAGCCGTGGCGCGTACACTTTCCGTGCCGTCTCGGTGCCCGAGCTCACCCAGCAGATGTTCGACCCCAAGAACATGATGGCTGCCTCCGACTTCCGCAACGGTCGCTATCTGACTTGCTCGGCCATTTT

>*Cercophora_thailandica*_MFLUCC_12-0845

ATTACAGAGTT-------GCAAAACTCCC--AACCCT-TTGTGAACC-AACCTAC----CAG-CTGCTCCGGCGAA-TATCTCTGATTAC------TTATTTAATAAGTTAAAACTTTCAACAACGGATCTCTTGGTTCTGGCATCGATGAAGAACGCAGCGAAATGCGATAAGTAATGTGAATTGCAGAATTCAGTGAATCATCGAATCTTTGAACGCACATTGCGCCCGCCAGTATTCTGGCGGGCATGCCTGTTCGAGCGTCATTTCAA---CCATCAAG-CCCTA----GGCTTGTGTTGGAG-CCCTGCG-GCT-GCC--GCAGCCTCC-CAAAATTAGTGGCGGGCTCGCT-ATTAC-ACCGAGTGCAGTAGTTTACTCTTCGCTCAGGATGT-GTGGCGGG-TGCTAGCCGTGAAACCCCCTAC------TCTCAAGGTTGACCTC???????????????????????????????????????????????????????????????????????CCC-TAGTAACGGCG-AGTGAAGCGGCAACAGCTCAAATTTGAAATCTGGCTCC--GG-CCCGAGTTGTAATTTGCAGAGG-AAGCTTCTGGTGATATACCGTCTAAGTCCCCTGGAACGGGGTGCCACAGTGGGTGAGAGCCCCATGTGACGGATGTAGAT--CCTGTGTGAAGCTCCTTCGACGAGTCGAGTAGTTTGGGAATGCTGCTCAAAATGGGAGGTAAATTCCTTCTAAAGCTAAATATTGGCCAGAGACCGATAGCGCACAAGTAGAGTGATCGAAAGATGAAAAGCACTTTGAAAAGAGGGTTAAACAGCACGTGAAATTGTTGAAAGGGAAGCGCTTATGACCAGACTTGCGCTGGGCTGATCATCCGGTGTTCTCACCGGTGCACTCGGCCCAGCTCAGGCCAGCATCGGTTTTGGCGGGGGGATAAAGGCGTAGGGAATGTAGCTCC--TCCGGGAGTG-TTATAGCCCAACGTGCAATACCCCCGCTGGGACCGAGGTTCGCGC-TCTGCAAGGATGCTGGCGTAATGGTCATCAGCGACCCGTCTTGAAACACGGACCAAGGAGTCAAGGTTTTGCGCGAGTGTTTGGGTGTCAAACCCGCACGCGTAATGAAAGTGAACGTAGGTGAGAG--CTTCGGCGCATCATCGACCGATCCTGATGTATTCGGATGGATTTGAGTAGGAGCGTTAAGCCTTGGACCCGAAAGATGGTGAACTATGCTTGGATAGGGTGAAGCCAGAGGAAACTCTGGTGGAGGCTCGCAGCGGTT-CTGACGTGCAAATCGATCGTCAAATCTGAGCAT-GGGGGCGAAAGACTAATCGAACCATCTAGTAGCTGGTTACCGCCGCTATCAAGTACTCGCTGGCCACCGGCAACTGGCGTGATCAAAAGAAGGCGATGAGCTCCACGGCCGGTGTGTCTCAAGTGTTGAACCGATATACTTTTGCATCGACCCTCTCCCATTTGCGGCGAACCAACACGCCTATCGGTCGCGACGGCAAGCTCGCTAAGCCACGGCAGCTGCACAACACGCACTGGGGCCTTGTCTGCCCTGCCGAGACCCCAGAAGGCCAGGCTTGCGGTCTCGTCAAGAACCTCTCTTTGATGTGTTACATCAGCGTCGGTACCAATGCGGAACCCATCGTCGATTTTATGACAGCCAGGGGGATGGATGTTCTCGAAGAGTACGAGCCGCTGCGGTATCCCAACGCCACCAAGGTCTTCGTCAACGGAACCTGGGTCGGCGTCCACCAAGAGCCTAAACACCTCGTCACCTTGGTCCAGAATCTAAGGCGGACGAGCATCATCTCCTTTGAGGTCTCGCTAGTTAGGGACATTCGTGACCGAGAATTCAAGATCTTCTCGGACGCCGGCCGCGTCATGAGGCCGCTCTTCGTTGTCGAGCAGGAGGAAAAGAGCGAC------ACGTCCAAGGTCCAGAAGGGCCAGCTGGCCCTCACACGGGCACACATGACCCGTCTGGATAGAGACAAGGAGCTCGGGCCCCTTG------------ACGAAGAATACTTTGGTTGGAACGGTCTCCTGAGAGAGGGCTGTGTCGAGTATCTCGACGCCGAGGAAGAGGAGACGGCCATGATTTGCATGACGCCCGAAGATTTGGAACATTATCGCAACACCAAGATGGGCATGTACAAGGACCAGCCCGAGGAGGACCCGGAAAACAAGGAGTATAACAAGCGCATCAAGACGAAGGCGAACCCGACCACGCACATGTACACCCACTGCGAGATCCATCCCAGCATGTTGCTTGGCATCTGCGCGAGCATCATCCCCTCCC????????????????????????????????????????????????????????????????????????????????????????????????????????????????????????????????????????????????????????????????????????????????????????????????????????????????????????????????????????????????????????????????????????????????????????????????????????????????????????????????????????????????????????????????????????????????????????????????????????????????????????????????????????????????????????????????????????????????????????????????????????????????????????????????????????????????????????????????????????????????????????????????????????????????????????????????????????????????????????

>*Echria_gigantospora*_F77-1

???????????????????????????????????????????????????????????????????????????????????????????????????????????????????????????????????????????????????????????????????????????????????????????????????????????????????????????????????????????????????????????????????????????????????????????????????????????????????????????????????????????????????????????????????????????????????????????????????????????????????????????????????????????????????????????????????????????????????????????????????????????????????????AAAGAAACCAACAGGG-ATTGCCC-TAGTAACGGCG-AGTGAAGCGGCAACAGCTCAAATTTGAAATCTGGCCTC--GG-CCCGAGTTGTAATTTGCAGAGG-AAGCTTCTGGCGCGGCGCTGTCCGAGTCCCCTGGAACGGGGCGCCATAGAGGGTGAGAGCCCCGTATGGATGGATGCCTA-GCCTGTGTGAAGCTCCTTCGACGAGTCGAGTAGTTTGGGAATGCTGCTCAAAATGGGAGGTAAATTCCTTCTAAAGCTAAATATTGGCCAGAGACCGATAGCGCACAAGTAGAGTGATCGAAAGATGAAAAGCACTTTGAAAAGAGGGTTAAATAGCACGTGAAATTGTTGAAAGGGAAGCGCTTGTGACCAGACTTGCGCCAGGCTGATCATCCGGTGTTCTCACCGGTGCACTCTGCCTGGCTCAGGCCAGCATCGGTTTCAGCGGGGGGATAAAGGCCTAGGGAACGTAGCTCC--CCCGGGAGTG-TTATAGCCCTGGGTGCAATGCCCCCGCCGGGACCGAGGTTCGCGCATCTGCTAGGATGCTGGCGTAATGGTCATCAGCGACCCGTCTTGAAACACGGACCAAGGAGTCAAGGTTTTGCGCGAGTGTTTGGGTGTTAAACCCGCACGCGTAATGAAAGTGAACGTAGGTGAGAG--CTTCGGCGCATCATCGACCGATCCTGATGTATTCGGATGGATTTGAGTAGGAGCGTTAAGCCTTGGACCCGAAAGATGGTGAACTATGCTTGGATAGGGTGAAGCCAGAGGAAACTCTGGTGGAGGCTCGCAGCGGTT-CTGACGTGCAAATCGATCGTCAAATCTGAGCAT-GGGGGCGAAAGACTAATCGAACCATCTAGTAGCTGGTTACCGCC??????????????????????????????????????????????????????????????????????????????????????????????????????????????????????????????????????????????????????????????????????????????????????????????????????????????????????????????????????????????????????????????????????????????????????????????????????????????????????????????????????????????????????????????????????????????????????????????????????????????????????????????????????????????????????????????????????????????????????????????????????????????????????????????????????????????????????????????????????????????????????????????????????????????????????????????????????????????????????????????????????????????????????????????????????????????????????????????????????????????????????????????????????????????????????????????????????????????????????????????????????????????????????????????????????????????????????????????????????????????????????????????????????????????????????????????????????????????????????????????????????????????????ACTTGTTGACCAAGTTCTCGATGTCGTTCGTCGTGAGGCTGAGGGCTGCGACTGCCTTCAGGGTTTCCAGATCACCCACTCGCTCGGTGGTGGTACCGGTGCCGGTATGGGTACCCTCCTTATCTCCAAGATTCGTGAGGAGTTCCCCGACCGCATGATGGCGACTTTCTCAGTCGTGCCGTCGCCCAAGGTCTCGGATACCGTTGTCGAGCCCTACAATGCCACTCTGTCGGTGCACCAGCTTGTCGAGAACTCGGACGAGACCTTCTGCATTGATAACGAGGCTCTCTACGATATCTGCATGCGGACGCTCAAGCTGTCGAACCCCTCGTACGGCGATCTGAACCACCTCGTTTCGGCCGTCATGTCCGGCGTCACCGTTTCTCTCCGTTTCCCTGGCCAGCTCAACTCTGATCTGCGCAAGCTTGCGGTGAACATGGTTCCCTTCCCTCGTCTCCACTTCTTCATGGTCGGCTTCGCGCCACTGACGAGCCGTGGCGCCCACTCCTTCCGCGCTGTCTCTGTGCCGGAGCTTACGCAGCAGATGTTCGACCCCAAGAACATGATGGCTGCTTCGGACTTCCGCAATGGCCGGTACCTTACTTGCTCGGCCATCTT

>*Echria_macrotheca*_Lundqvist_2311

???????????????????????????????????????????????????????????????????????????????????????????????????????????????????????????????????????????????????????????????????????????????????????????????????????????????????????????????????????????????????????????????????????????????????????????????????????????????????????????????????????????????????????????????????????????????????????????????????????????????????????????????????????????????????????????????????????????????????????????????????????????????????????AAAGAAACCAACAGGG-ATTGCCC-TAGTAACGGCG-AGTGAAGCGGCAACAGCTCAAATTTGAAATCTGGCCCC--GG-CCCGAGTTGTAATTTGCAGAGG-AAGCTTCTGGCGCGGCGCCGTCCGAGTCCCCTGGAACGGGGCGCCATAGAGGGTGAGAGCCCCGTATGGATGGACGCCTA-GCCTGTGTGAAGCTCCTTCGACGAGTCGAGTAGTTTGGGAATGCTGCTCAAAATGGGAGGTAAATTCCTTCTAAAGCTAAATATTGGCCAGAGACCGATAGCGCACAAGTAGAGTGATCGAAAGATGAAAAGCACTTTGAAAAGAGGGTTAAATAGCACGTGAAATTGTTGAAAGGGAAGCGCTTGTGACCAGACTTGCGCCAGGCTGATCATCCGGTGTTCTCACCGGTGCACTCTGCCTGGCTCAGGCCAGCATCGGTTTCAGCGGGGGGATAAAGGCCTAGGGAACGTAGCTCC--CCCGGGAGTG-TTATAGCCCTGGGTGCAATGCCCCCGCCGGGACCGAGGTTCGCGCATCTGCTAGGATGCTGGCGTAATGGTCATCAGCGACCCGTCTTGAAACACGGACCAAGGAGTCAAGGTTTTGCGCGAGTGTTTGGGTGTTAAACCCGCACGCGTAATGAAAGTGAACGTAGGTGAGAG--CTTCGGCGCATCATCGACCGATCCTGATGTATTCGGATGGATTTGAGTAGGAGCGTTAAGCCTTGGACCCGAAAGATGGTGAACTATGCTTGGATAGGGTGAAGCCAGAGGAAACTCTGGTGGAGGCTCGCAGCGGTT-CTGACGTGCAAATCGATCGTCAAATCTGAGCAT-GGGGGCGAAAGACTAATCGAACCATCTAGTAGCTGGTTACCGCC??????????????????????????????????????????????????????????????????????????????????????????????????????????????????????????????????????????????????????????????????????????????????????????????????????????????????????????????????????????????????????????????????????????????????????????????????????????????????????????????????????????????????????????????????????????????????????????????????????????????????????????????????????????????????????????????????????????????????????????????????????????????????????????????????????????????????????????????????????????????????????????????????????????????????????????????????????????????????????????????????????????????????????????????????????????????????????????????????????????????????????????????????????????????????????????????????????????????????????????????????????????????????????????????????????????????????????????????????????????????????????????????????????????????????????????????????????????????????????????????????????????????????GCTTGTTGACCAAGTTCTCGATGTCGTCCGTCGTGAGGCCGAGGGCTGCGACTGCCTCCAGGGTTTCCAGATCACGCACTCGCTCGGTGGAGGTACCGGTGCCGGTATGGGTACCCTCCTTATCTCCAAGATTCGTGAGGAGTTCCCCGACCGCATGATGGCGACTTTCTCGGTCGTGCCGTCGCCCAAGGTCTCGGATACCGTTGTCGAGCCCTACAACGCCACTCTGTCGGTGCACCAGCTTGTCGAGAACTCGGACGAGACGTTCTGCATTGATAACGAGGCTCTCTACGATATCTGCATGCGGACGCTCAAGCTGTCGAACCCCTCATACGGCGACCTGAACCACCTCGTTTCGGCCGTCATGTCGGGCGTCACCGTTTCTCTCCGTTTCCCTGGCCAGCTCAACTCTGATCTCCGCAAGCTTGCGGTGAACATGGTTCCCTTCCCTCGTCTCCACTTCTTCATGGTGGGCTTCGCGCCGCTGACGAGCCGTGGCGCCCACTCTTTCCGCGCTGTCTCTGTGCCGGAGCTTACGCAGCAGATGTTCGACCCCAAGAACATGATGGCTGCTTCGGACTTCCGCAATGGCCGTTACCTTACTTGCTCGGCCATCTT

>*Episternus_onthophagi*_KRAM_F58223

ATTAGCGAGTT-------GCATAACTCCC--AAACCA-TTGCGAACC-TACCTA-----TCG-TTGCCTCGCCGAGGCCTCTCTGAGTAC------ACTTATAACAAGTCAAAACTTTCAACAACGGATCTCTTGGTTCTGGCATCGATGAAGAACGCAGCGAAATGCGATAAGTAATGCGAATTGCAGAATTCCGTGAGTCATCGAATCTTTGAACGCACATTGCGCCCGCCAGTATTCTGGCGGGCATGCCTGTCCGAGCGTCATTTCAA---CCATCAAG-CCCCCG---GGCTTGCGTTGGGG-TCCTGCG-GCT-GCC-CGCAGGCCCC-GAAAGACAGTGGCGGGCTCGCG-AACGCGACCGAGCGCAGTAACATTCTCTTCGCTCTGGGCGC-GCCGCGGGTTACCAGCCGTGAAACCCCCCAT-----TTCTCAAGGTTGACCTCGGAT??????????????????????????????????????????????????????????????????????????????????????????CGGCAACAGCTCAAATTTGAAATCTGGCCTC--GG-CCCGAGTTGTAATTTGTAGAGG-GCGCTTCTGGCGCGGCATCGACTGAGTCCCCTGGAACGGGGCGCCAAAGAGGGTGAGAGCCCCGTAGAGTCGGCTGCTAA-GCCTGTGCGAAGCCCCTTCGACGAGTCGAGTAGTTTGGGAATGCTGCTCAAAATGGGAGGTAAATTCCTTCTAAAGCTAAATACCGGCCAGAGACCGATAGCGCACAAGTAGAGTGATCGAAAGATGAAAAGCACTTTGAAAAGAGGGTTAAACAGCACGTGAAATTGTTGAAAGGGAAGCGCTTGTGGCCAGACTTGCGCCGCGCTGATCATCCGGTGTTCTCACCGGTGCACTCGGCGCGGCTCAGGCCAGCATCGGTTTTCGCGGGGGGATAAAGGCGCCGGGAACGTAGCTCC--TCCGGGAGTG-TTATAGCCCGGCGTGCCATGCCCCCGCGGGGACCGAGGTCCGCGCGTATGCAAGGATGCTGGCGTAATGGCCATCAGCGACCCGTCTTGAAACACGGACCAAGGAGTCAAGGTTTTGCGCGAGTGTTTGGGTGTTAAACCCGCACGCGTAATGAAAGTGAACGTAGGTGAGAG--CTTCGGCGCATCATCGACCGATCCTGATGTATTCGGATGGATTTGAGTAGGAGCGTTAAGCCTTGGACCCGAAAGATGGTGAACTATGCTTGGATAGGGTGAAGCCAGAGGAAACTCTGGTGGAGGCTCGCAGCGGTT-CTGACGTGCAAATCGATCGTCAAATCTGAGCAT-GGGGGCGAAAGACTAAT???????????????????????????????????????????????????????????????????????????????????????????????????????????????????????????????????????????????????????????????????????????????????????????????????????????????????????????????????????????????????????????????????????????????????????????????????????????????????????????????????????????????????????????????????????????????????????????????????????????????????????????????????????????????????????????????????????????????????????????????????????????????????????????????????????????????????????????????????????????????????????????????????????????????????????????????????????????????????????????????????????????????????????????????????????????????????????????????????????????????????????????????????????????????????????????????????????????????????????????????????????????????????????????????????????????????????????????????????????????????????????????????????????????????????????????????????????????????????????????????????????????????????????????????????????????????????????????????????????????????????????????????????????????????????????????????????????????????????????????????????????????????????????????????????????????????????????????????????????????????????????????????????????????????????????????????????????????????????????????????????????????????????????????????????????????????????????????????????????????????????????????????????????????????????????????????????????????????????????????????????????????????????????????????????????????????????????????????????????????????????????????????????????????????????????????????????????????????????????????????????????????????????????

>*Immersiella_caudata*_SMH3298

??????????????????????????????????????????????????????????????????????????????????????????????????????????????????????????????????????????????????????????????????????????????????????????????????????????????????????????????????????????????????????????????????????????????????????????????????????????????????????????????????????????????????????????????????????????????????????????????????????????????????????????????????????????????????????????????????????????????????????????????????????????????????????????????CCAACAGGG-ATTGCCC-TAGTAACGGCG-AGTGAAGCGGCAACAGCTCAAATTTGAAATCTGGCTTC--GG-CCCGAGTTGTAATTTGCAGAGG-AAGATTCTGGCGACGCGCCGTCCGAGTCCCCTGGAACGGGGCGCCATAGAGGGTGAGAGCCCCGTATGGATGGATGCCTA-GCCTGTGTGAATCTCCTTCGACGAGTCGAGTAGTTTGGGAATGCTGCTCAAAATGGGAGGTAAATTCCTTCTAAAGCTAAATATTGGCCAGAGACCGATAGCGCACAAGTAGAGTGATCGAAAGATGAAAAGCACTTTGAAAAGAGGGTTAAACAGCACGTGAAATTGTTGAAAGGGAAGCGCTTGTGACCAGACTTGCGCCTGGCTGATCATCCGGTGTTCTCACCGGTGCACTCTGCCAGGCTCAGGCCAGCATCGGTTTTCGTGGGGGGATAAAGGTCCCGGGAACGTGGCTCC--TCCGGGAGTG-TTATAGCCCGGGGCGTAATGCCCTCGCGGGGACCGAGGTTCGCGCATCTGCAAGGATGCTGGCGTAATGGTCATCAGCGACCCGTCTTGAAACACGGACCAAGGAGTCAAGGTTTTGCGCGAGTGTTTGGGTGTTAAACCCGCACGCGTAATGAAAGTGAACGTAGGTGAGAG--CTTCGGCGCATCATCGACCGATCCTGATGTATTCGGATGGATTTGAGTAGGAGCGTTAAGCCTTGGACCCGAAAGATGGTGAACTATGCTTGGATAGGGTGAAGCCAGAGGAAACTCTGGTGGAGGCTCGCAGCGGTT-CTGACGTGCAAATCGATCGTCAAATCTGAGCAT-GGGGGCGAAAGACTAATCGAACCATCTAGTAGCTGGTTACCGCCGCTCTGAAGTACTCGTTGGCAACCGGCAACTGGGGCGATCAGAAGAAGGCCATGAGCTCCACCGCCGGTGTCTCGCAAGTCTTGAACCGGTACACATTTGCCTCCACACTTTCCCACTTGCGGCGAACCAACACGCCAATCGGTCGAGATGGCAAGCTCGCGAAACCTCGTCAACTCCATAACACGCATTGGGGCCTGGTCTGTCCTGCGGAGACGCCAGAGGGTCAGGCTTGTGGTCTGGTCAAGAACCTCTCCCTCATGTGCTACATCAGCGTTGGCACCAACGCTGATCCAATCGTCGACTTCATGATTGCTAGGAACATGGAAGTCCTCGAAGAGTACGAGCCCTTGCGGTATCCTAATGCCACCAAGGTCTTTGTCAACGGCACGTGGGTTGGCGTCCACCAGGATCCGAAGCATTTGGTCACGCTGGTGCAGAATCTACGGAGATCGAACATCATCTCATTCGAGGTTTCGCTTGTTAGAGACATCCGAGACCGAGAATTCAAGATTTTCTCTGACGCTGGCCGCGTCATGAGACCACTCTTCGTGGTTGAACAGGAAGACGACCACGAGGGCAAGACTAGCAAGGTGGAGAAGGGCCAGCTCGTTCTTACAAAGGCACACATCCATCAATTAGAACGAGACAAGGAGGTTGGGAGATACC------------ACAAGGACTACTTTGGGTGGAATGGCCTTCTCAGGTCCGGGTGCATCGAATACCTCGATGCCGAAGAGGAAGAGACCACCATGATCTGCATGACGCCAGAGGACTTGGACATTTACCGGCTGACTAAGCT------------CGGCTTCACCGTCAACGAGGAGGATCACAGCGAGGGCAACAAGCGCATCAAAACTAGACTGAACCCGACTACCCACATGTACACCCATTGCGAGATCCACCCCAGCATGCTGCTCGGCATCTGTGCCAGCATCATCCCCTTTCCGGATCACAACCAGGCTTGTTGACCAAGTTCTCGATGTCGTCCGTCGCGAGGCCGAGGGCTGCGACTGCCTCCAGGGCTTCCAGATCACCCACTCTCTCGGCGGTGGTACCGGTGCCGGTATGGGTACCCTCCTGATCTCCAAAATTCGCGAGGAGTTCCCCGACCGCATGATGGCCACTTTCTCGGTCGTCCCCTCCCCCAAGGTGTCGGATACCGTCGTTGAGCCGTACAACGCCACCCTTTCTGTCCACCAGCTTGTCGAGAACTCGGACGAGACCTTTTGCATTGACAACGAGGCTCTCTACGACATCTGCATGCGCACGCTCAAGCTGTCGAACCCCTCGTACGGCGACCTCAACCACCTGGTCTCTGCCGTCATGTCGGGTGTCACTGTTTCCCTGCGCTTCCCCGGCCAGCTCAACTCCGACCTTCGCAAGCTTGCCGTCAATATGGTTCCTTTCCCGCGTCTCCATTTCTTCATGGTCGGCTTCGCGCCTCTTACGAGCCGTGGCGCGCACTCTTTCCGTGCCGTCTCCGTCCCTGAGTTGACTCAGCAGATGTTCGACCCCAAGAACATGATGGCTGCTTCCGATTTCCGCAACGGTCGCTACCTGACTTGCTCTGCCATCTT

>*Immersiella_hirta*_E00204487

???????????????????????????????????????????????????????????????????????????????????????????????????????????????????????????????????????????????????????????????????????????????????????????????????????????????????????????????????????????????????????????????????????????????????????????????????????????????????????????????????????????????????????????????????????????????????????????????????????????????????????????????????????????????????????????????????????????????????????????????????????????????????????AAAGAAACCAACAGGG-ATTGCCC-TAGTAACGGCG-AGTGAAGCGGCAACAGCTCAAATTTGAAATCTGGCTTC--GG-CCCGAGTTGTAATTTGCAGAGG-AAGATTCTGGCGACGCGCCGTCCGAGTCCCCTGGAACGGGGCGCCATAGAGGGTGAGAGCCCCGTATGGACGGATGCCTA-GCCTGTGTGAATCTCCTTCGACGAGTCGAGTAGTTTGGGAATGCTGCTCAAAATGGGAGGTAAATTCCTTCTAAAGCTAAATATTGGCCAGAGACCGATAGCGCACAAGTAGAGTGATCGAAAGATGAAAAGCACTTTGAAAAGAGGGTTAAACAGCACGTGAAATTGTTGAAAGGGAAGCGCTTGTGACCAGACTTGCGCCTGGCTGATCATCCGGTGTTCTCACCGGTGCACTCTGCCAGGCTCAGGCCAGCATCGGTTTTCGTGGGGGGATAAAGGTCCCGGGAATGTGGCTCC--TCCGGGAGTG-TTATAGCCCGGGGCGTAATGCCCTCGCGGGGACCGAGGTTCGCGCATCTGCAAGGATGCTGGCGTAATGGTCATCAGCGACCCGTCTTGAAACACGGACCAAGGAGTCAAGGTTTTGCGCGAGTGTTTGGGTGTCAAACCCGCACGCGTAATGAAAGTGAACGTAGGTGAGAG--CTTCGGCGCATCATCGACCGATCCTGATGTATTCGGATGGATTTGAGTAGGAGCGTTAAGCCTTGGACCCGAAAGATGGTGAACTATGCTTGGATAGGGTGAAGCCAGAGGAAACTCTGGTGGAGGCTCGCAGCGGTT-CTGACGTGCAAATCGATCGTCAAATCTGAGCAT-GGGGGCGAAAGACTAATCGAACCATCTAGTAGCTGGTTACCGCC??????????????????????????????????????????????????????????????????????????????????????????????????????????????????????????????????????????????????????????????????????????????????????????????????????????????????????????????????????????????????????????????????????????????????????????????????????????????????????????????????????????????????????????????????????????????????????????????????????????????????????????????????????????????????????????????????????????????????????????????????????????????????????????????????????????????????????????????????????????????????????????????????????????????????????????????????????????????????????????????????????????????????????????????????????????????????????????????????????????????????????????????????????????????????????????????????????????????????????????????????????????????????????????????????????????????????????????????????????????????????????????????????????????????????????????????????????????????????????????????????????????????????GCTTGTTGACCAGGTTCTCGATGTCGTCCGTCGTGAGGCTGAGGGCTGCGACTGCCTCCAGGGCTTCCAGATCACCCACTCTCTCGGTGGTGGTACCGGTGCCGGTATGGGTACCCTCCTGATCTCCAAGATCCGCGAGGAGTTCCCCGACCGCATGATGGCCACTTTCTCGGTCGTCCCCTCTCCCAAGGTGTCGGATACCGTTGTTGAGCCCTACAACGCCACCCTCTCCGTTCATCAGCTCGTCGAGAACTCGGACGAGACCTTCTGCATTGACAACGAGGCTCTCTACGATATCTGCATGCGTACCCTGAAGCTGTCCAACCCCTCGTACGGTGACCTCAACCACCTGGTCTCGGCCGTCATGTCGGGTGTCACGGTTTCCCTGCGCTTCCCCGGCCAGCTCAACTCGGATCTCCGCAAGCTTGCCGTCAACATGGTTCCTTTCCCGCGTCTCCATTTCTTCATGGTCGGCTTCGCGCCTCTTACGAGCCGTGGCGCGCACTCTTTCCGTGCCGTCTCCGTTCCTGAGTTGACCCAGCAGATGTTTGACCCCAAGAACATGATGGCTGCCTCTGATTTCCGCAACGGTCGCTACCTGACTTGCTCTGCCATCTT

>*Immersiella_hirta*_E00204950

??????????????????????????????????????????????????????????????????????????????????????????????????????????????????????????????????????????????????????????????????????????????????????????????????????????????????????????????????????????????????????????????????????????????????????????????????????????????????????????????????????????????????????????????????????????????????????????????????????????????????????????????????????????????????????????????????????????????????????????????????????????????????????????????????????????????????????????????????????????????????????????????????????????????????????????????????????GGCGACGCGCCGTCCGAGTCCCCTGGAACGGGGCGCCATAGAGGGTGAGAGCCCCGTATGGACGGATGCCTA-GCCTGTGTGAATCTCCTTCGACGAGTCGAGTAGTTTGGGAATGCTGCTCAAAATGGGAGGTAAATTCCTTCTAAAGCTAAATATTGGCCAGAGACCGATAGCGCACAAGTAGAGTGATCGAAAGATGAAAAGCACTTTGAAAAGAGGGTTAAACAGCACGTGAAATTGTTGAAAGGGAAGCGCTTGTGACCAGACTTGCGCCTGGCTGATCATCCGGTGTTCTCACCGGTGCACTCTGCCAGGCTCAGGCCAGCATCGGTTTTCGTGGGGGGATAAAGGTCCCGGGAATGTGGCTCC--TCCGGGAGTG-TTATAGCCCGGGGCGTAATGCCCTCGCGGGGACCGAGGTTCGCGCATCTGCAAGGATGCTGGCGTAATGGTCATCAGCGACCCGTCTTGAAACACGGACCAAGGAGTCAAGGTTTTGCGCGAGTGTTTGGGTGTCAAACCCGCACGCGTAATGAAAGTGAACGTAGGTGAGAG--CTTCGGCGCATCATCGACCGATCCTGATGTATTCGGATGGATTTGAGTAGGAGCGTTAAGCCTTGGACCCGAAAGATGGTGAACTATGCTTGGATAGGGTGAAGCCAGAGGAAACTCTGGTGGAGGCTCGCAACGGTT-CTGACGTGCAAATCGATCGTCAAATCTGAGCAT-GGGGGCGAAAGACTAATCGAACCATCTAGTAGCTGGTTACCGCC??????????????????????????????????????????????????????????????????????????????????????????????????????????????????????????????????????????????????????????????????????????????????????????????????????????????????????????????????????????????????????????????????????????????????????????????????????????????????????????????????????????????????????????????????????????????????????????????????????????????????????????????????????????????????????????????????????????????????????????????????????????????????????????????????????????????????????????????????????????????????????????????????????????????????????????????????????????????????????????????????????????????????????????????????????????????????????????????????????????????????????????????????????????????????????????????????????????????????????????????????????????????????????????????????????????????????????????????????????????????????????????????????????????????????????????????????????????????????????????????????????????????????GCTTGTTGACCAGGTTCTCGATGTCGTCCGTCGTGAGGCTGAGGGCTGCGACTGCCTCCAGGGCTTCCAGATCACCCACTCTCTCGGTGGTGGTACCGGTGCCGGTATGGGTACCCTCCTGATCTCCAAGATCCGCGAGGAGTTCCCCGACCGCATGATGGCCACTTTCTCGGTCGTCCCCTCTCCCAAGGTGTCGGATACCGTTGTTGAGCCCTACAACGCCACCCTCTCCGTTCATCAGCTCGTCGAGAACTCGGACGAGACCTTCTGCATTGACAACGAGGCTCTCTACGATATCTGCATGCGTACCCTGAAGCTGTCCAACCCCTCGTACGGTGACCTCAACCACCTGGTCTCGGCCGTCATGTCGGGTGTCACGGTTTCCCTGCGCTTCCCCGGCCAGCTCAACTCGGATCTCCGCAAGCTTGCCGTCAACATGGTTCCTTTCCCGCGTCTCCATTTCTTCATGGTCGGCTTCGCGCCTCTTACGAGCCGTGGCGCGCACTCTTTCCGTGCCGTCTCCGTTCCTGAGTTGACCCAGCAGATGTTTGACCCCAAGAACATGATGGCTGCCTCTGATTTCCGCAACGGTCGCTACCTGACTTGCTCTGCCATCTT

>*Immersiella_immersa*_SMH2589

??????????????????????????????????????????????????????????????????????????????????????????????????????????????????????????????????????????????????????????????????????????????????????????????????????????????????????????????????????????????????????????????????????????????????????????????????????????????????????????????????????????????????????????????????????????????????????????????????????????????????????????????????????????????????????????????????????????????????ATACCCGCTGAACTTAAGCATATCAATAAGCGGAGGAAAAGAAACCAACAGGG-ATTGCCC-CAGTAACGGCG-AGTGAAGCGGCAACAGCTCAAATTTGAAATCTGGCTTC--GG-CCCGAGTTGTAATTTGCAGAGG-AAGATTCTGGTGACGCGCCGTCCGAGTCCCCTGGAACGGGGCGCCATAGAGGGTGAGAGCCCCGTATGGATGGATGCCTA-GCCTGTGTGAATCTCCTTCGACGAGTCGAGTAGTTTGGGAATGCTGCTCAAAATGGGAGGTAAATTCCTTCTAAAGCTAAATATTGGCCAGAGACCGATAGCGCACAAGTAGAGTGATCGAAAGATGAAAAGCACTTTGAAAAGAGGGTTAAACAGCACGTGAAATTGTTGAAAGGGAAGCGCTTGTGACCAGACTTGCGCCTGGCTGATCATCCGGTGTTCTCACCGGTGCACTCTGCCAGGCTCAGGCCAGCATCGGTTTTCGTGGGGGGATAAAGGTCCTGGGAACGTAGCTCC--TCTGGGAGTG-TTATAGCCCGGGGCGTAATGCCCTCGCGGGGACCGAGGTTCGCGCATCTGCAAGGATGCTGGCGTAATGGTCATCAGCGACCCGTCTTGAAACACGGACCAAGGAGTCAAGGTTTTGCGCGAGTGTTTGGGTGTCAAACCCGCACGCGTAATGAAAGTGAACGTAGGTGAGAG--CTTCGGCGCATCATCGACCGATCCTGATGTATTCGGATGGATTTGAGTAGGAGCGTTAAGCCTTGGACCCGAAAGATGGTGAACTATGCTTGGATAGGGTGAAGCCAGAGGAAACTCTGGTGGAGGCTCGCAGCGGTT-CTGACGTGCAAATCGATCGTCAAATCTGAGCAT-GGGGGCGAAAGACTAATCGAACCATCTAGTAGCTGGTTACCGCC????????????????????????????????????????????????????????????????????????????????????????????????????????????????????????????????????????????????????????????????????????????????????????????????????????????????????????????????????????????????????????????????????????????????????????????????????????????????????????????????????????????????????????????????????????????????????????????????????????????????????????????????????????????????????????????????????????????????????????????????????????????????????????????????????????????????????????????????????????????????????????????????????????????????????????????????????????????????????????????????????????????????????????????????????????????????????????????????????????????????????????????????????????????????????????????????????????????????????????????????????????????????????????????????????????????????????????????????????????????????????????????????????????????????????????????????????????????????????????????????????????????????????????????????????????????????????????????????????????????????????????????????????????????????????????????????????????????????????????????????????????????????????????????????????????????????????????????????????????????????????????????????????????????????????????????????????????????????????????????????????????????????????????????????????????????????????????????????????????????????????????????????????????????????????????????????????????????????????????????????????????????????????????????????????????????????????????????????????????????????????????????????????????????????????????????????????????????????????????????

>*Immersiella_immersa*_SMH4104

???????????????????????????????????????????????????????????????????????????????????????????????????????????????????????????????????????????????????????????????????????????????????????????????????????????????????????????????????????????????????????????????????????????????????????????????????????????????????????????????????????????????????????????????????????????????????????????????????????????????????????????????????????????????????????????????????????????????????????????????????????????????????????AAAGAAACCAACAGGG-ATTGCCC-CAGTAACGGCG-AGTGAAGCGGCAACAGCTCAAATTTGAAATCTGGCTTC--GG-CCCGAGTTGTAATTTGCAGAGG-AAGATTCTGGTGACGCGCCGTCCGAGTCCCCTGGAACGGGGCGCCATAGAGGGTGAGAGCCCCGTATGGATGGATGCCTA-GCCTGTGTGAATCTCCTTCGACGAGTCGAGTAGTTTGGGAATGCTGCTCAAAATGGGAGGTAAATTCCTTCTAAAGCTAAATATTGGCCAGAGACCGATAGCGCACAAGTAGAGTGATCGAAAGATGAAAAGCACTTTGAAAAGAGGGTTAAACAGCACGTGAAATTGTTGAAAGGGAAGCGCTTGTGACCAGACTTGCGCCTGGCTGATCATCCGGTGTTCTCACCGGTGCACTCTGCCAGGCTCAGGCCAGCATCGGTTTTCGTGGGGGGATAAAGGTCCTGGGAACGTAGCTCC--TCTGGGAGTG-TTATAGCCCGGGGCGTAATGCCCTCGCGGGGACCGAGGTTCGCGCATCTGCAAGGATGCTGGCGTAATGGTCATCAGCGACCCGTCTTGAAACACGGACCAAGGAGTCAAGGTTTTGCGCGAGTGTTTGGGTGTCAAACCCGCACGCGTAATGAAAGTGAACGTAGGTGAGAG--CTTCGGCGCATCATCGACCGATCCTGATGTATTCGGATGGATTTGAGTAGGAGCGTTAAGCCTTGGACCCGAAAGATGGTGAACTATGCTTGGATAGGGTGAAGCCAGAGGAAACTCTGGTGGAGGCTCGCAGCGGTT-CTGACGTGCAAATCGATCGTCAAATCTGAGCAT-GGGGGCGAAAGACTAATCGAACCATCTAGTAGCTGGTTACCGCCGCTCTAAAGTACTCGTTGGCAACCGGTAACTGGGGCGATCAGAAGAAGGCCATGAGTTCCACCGCCGGTGTCTCGCAAGTCTTGAACCGGTACACATTTGCCTCCACACTTTCCCATTTGCGGCGAACCAACACACCGATCGGTCGAGATGGCAAGCTTGCGAAACCTCGTCAACTCCATAACACACATTGGGGCCTGGTCTGCCCTGCAGAGACGCCAGAGGGTCAGGCCTGTGGCCTGGTCAAGAACCTCTCCCTCATGTGCTACATCAGCGTTGGCACCAACGCTGATCCAATCGTCGACTTCATGATTGCTAGGAACATGGAAGTCCTCGAAGAGTATGAGCCCTTGCGATATCCCAATGCCACCAAGGTCTTTGTCAACGGCACATGGGTTGGCGTCCACCAGGATCCGAAGCATTTGGTCACGCTGGTGCAGAATCTACGGAGATCGAACATCATCTCATTTGAGGTTTCGCTTGTTAGAGACATCCGGGACCGAGAATTCAAGATTTTCTCTGATGCTGGCCGCGTCATGAGACCGCTCTTTGTGGTTGAGCAGGAAGACGACCATGAGGGCAAGACTAGCAAGGTCGATAAGGGCCAGCTCGTTCTCACGAAGGCACACATCCATCAGCTGGAACGAGACAAGGAGGTTGGGAGATACC------------ACAAGGACTACTTTGGGTGGAATGGCCTTCTCAGGTCCGGGTGCATCGAATACCTCGATGCCGAAGAGGAAGAGACTACCATGATCTGTATGACGCCGGAGGACTTGGACATTTACCGGCTGAACAAGCT------------TGGCTTCGGCGTCCACGAGGAGGATCACAGCGAGGGCAACAAGCGCATCAAAACGAGACTGAACCCGACTACTCACATGTACACCCATTGCGAGATCCACCCCAGCATCCTGCTTGGCATCTGTGCCAGCATCATCCCCTTCCCGGATCACAACCAGGCTTGTTGACCAAGTTCTCGATGTCGTCCGTCGCGAGGCCGAGGGCTGCGACTGCCTTCAGGGTTTCCAGATCACCCACTCTCTCGGTGGTGGTACCGGTGCCGGTATGGGTACCCTCCTGATCTCCAAGATTCGCGAAGAGTTCCCCGACCGCATGATGGCCACTTTCTCGGTCGTCCCCTCCCCCAAGGTGTCGGATACCGTCGTTGAGCCGTACAACGCCACCCTTTCCGTCCACCAGCTTGTCGAGAACTCGGACGAGACCTTCTGCATTGACAACGAGGCTCTCTACGACATCTGCATGCGTACGCTCAAGCTGTCGAACCCCTCGTACGGCGATCTCAACCACCTAGTCTCTGCCGTCATGTCGGGTGTCACTGTTTCCCTGCGCTTCCCCGGCCAGCTCAACTCCGACCTTCGCAAGCTTGCCGTCAACATGGTTCCTTTCCCGCGCCTCCATTTCTTCATGGTCGGCTTCGCGCCTCTTACGAGCCGTGGCGCGCATTCTTTCCGTGCCGTCTCCGTCCCTGAGTTGACCCAGCAGATGTTTGACCCCAAGAACATGATGGCTGCCTCCGATTTCCGCAACGGTCGCTACCTAACTTGCTCTGCCATCTT

>*Jugulospora_antarctica*_IMI_381338

???????????????????????????????????????????????????????????????????????????????????????????????????????????????????????????????????????????????????????????????????????????????????????????????????????????????????????????????????????????????????????????????????????????????????????????????????????????????????????????????????????????????????????????????????????????????????????????????????????????????????????????????????????????????????????????????????????????????????????????????????????????????????????AAAGAAACCAACAGGG-ATTGCCC-CAGTAACGGCG-AGTGAAGCGGCAACAGCTCAAATTTGAAATCTGGCCTC--GG-CCCGAGTTGTAATTTGCAGAGG-AAGCTTCTGGTGCGGTCCGTCCCGAGTCCCCTGGAACGGGGCGCCGTAGAGGGTGAGAGCCCCGTACGGACGGATACCAA-TCCTGTGTGAAGCTCCTTCGACGAGTCGAGTAGTTTGGGAATGCTGCTCAAAATGGGAGGTAAATTCCTTCTAAAGCTAAATACCGGCCAGAGACCGATAGCGCACAAGTAGAGTGATCGAAAGATGAAAAGCACTTTGAAAAGAGGGTTAAACAGCACGTGAAATTGTTGAAAGGGAAGCGCTTGTGACCAGACTTGCGCCAGGCTGATCATCCGGTGTTCTCACCGGTGCACTCGGCCTGGCTCAGGCCAGCATCGGTTCCCGCGGGGGGATAAAGGTCCCGGGAATGTAGCTCC--TCCGGGAGTG-TTATAGCCCGGGGCGCAATGCCCCCGTGGGGACCGAGGTTCGCGCATCTGCAAGGATGCTGGCGTAATGGTCATCAGCGACCCGTCTTGAAACACGGACCAAGGAGTCAAGGTTTTGCGCGAGTGTTTGGGTGTCAAACCCGCACGCGTAATGAAAGTGAACGTAGGTGAGAG--CTTCGGCGCATCATCGACCGATCCTGATGTCTTCGGATGGATTTGAGTAGGAGCGTTAAGCCTTGGACCCGAAAGATGGTGAACTATGCTTGGATAGGGTGAAGCCAGAGGAAACTCTGGTGGAGGCTCGCAGCGGTT-CTGACGTGCAAATCGATCGTCAAATCTGAGCAT--GGGGCGAA???????????????????????????????????GCGCTCAAGTACTCGCTGGCGACTGGAAACTGGGGTGATCAAAAGAAAGCCATGAGCTCCACTGCGGGTGTCTCGCAGGTCCTGAACCGATACACATTTGCCTCTACACTTTCTCACTTGCGGCGAACGAACACGCCTATTGGGCGCGATGGCAAGCTCGCAAAACCCCGCCAACTCCATAACACACACTGGGGTCTGGTCTGTCCAGCAGAGACGCCCGAAGGCCAGGCTTGCGGTCTGGTCAAGAATCTCTCGCTGATGTGCTACATTAGCGTTGGTACCAATGCGGATCCTATCATCGAATTCATGATTGCCAGGAACATGGAAGTCCTGGAAGAGTATGAGCCTCTGCGGTATCCGAATGCCACAAAGGTTTTCGTCAATGGCACCTGGGTCGGTGTCCACCAAGACCCAAAGCACCTGGTCAGCTTGGTTCAGAGCCTAAGGAGATCCAACATTATCAGTTTCGAGGTTTCCCTGGTTCGTGACATCCGAGATAGGGAGTTCAAGATCTTCTCCGATGCAGGGCGTGTCATGAGACCGCTATTCGTCGTTGAACAGGAGGATGACGGCGAG---------AGCAAGGTTGAGAAGGGCCAGCTGGTTCTTACAAAGTCGCAAATCCTCAAACTAGAAAAAGACAAGGAGATTGGCAAATACC------------ATCCGGATTACTTCGGCTGGAATGGCCTCCTAAGGGAGGGCTGTGTCGAATACTTGGATGCGGAAGAAGAGGAAACGGCGATGATCTGTATGACGCCCGAGGACCTCGACACGTACCGGCTGGCCAAGCT------------TGGGTTCAACGTCGCCGAGGAGGATCCTAGTGAAGGCAACAAGCGCATCAAGACTAGGCTCAACCCGACGACCCATATGTATACTCACTGCGAGATACACCCCAGCATGCTGCTTGGTATCTGCGCCAGCATCATTCCCTTCCCCGATCACAAC???GCTTGTTGACCAAGTTCTCGATGTCGTCCGTCGCGAGGCCGAGGGCTGCGACTGCCTCCAGGGCTTCCAGATCACCCACTCTCTCGGTGGTGGTACCGGTGCCGGTATGGGTACCCTCTTGATCTCCAAGATTCGCGAGGAGTTCCCCGACCGCATGATGGCGACTTTCTCTGTCGTCCCCTCCCCCAAGGTCTCTGACACCGTCGTTGAGCCCTACAACGCCACGCTCTCCGTCCACCAACTTGTCGAGAACTCGGACGAGACCTTCTGCATTGACAACGAGGCTCTCTACGACATCTGCATGCGGACGCTCAAGCTGTCCAACCCTTCATACGGTGATCTCAACCACCTGGTCTCTGCCGTCATGTCCGGCGTTACCGTTTCCCTGCGCTTCCCCGGCCAGCTCAACTCCGATCTCCGCAAGCTCGCCGTGAACATGGTTCCTTTCCCCCGTCTCCACTTCTTCATGGTCGGCTTCGCGCCGCTTACTAGCCGTGGAGCGCACTCTTTCCGTGCTGTCTCGGTTCCCGAGTTGACCCAGCAGATGTTCGACCCCAAGAACATGATGGCTGCGTCTGACTTCCGCAACGGTCGCTACCTGACCTGCTCTGCCATCTT

>*Jugulospora_carbonaria*_ATCC_34567

???????????????????????????????????????????????????????????????????????????????????????????????????????????????????????????????????????????????????????????????????????????????????????????????????????????????????????????????????????????????????????????????????????????????????????????????????????????????????????????????????????????????????????????????????????????????????????????????????????????????????????????????????????????????????????????????????????????????????????????????????????????????????????AAAGAAACCAACAGGG-ATTGCCC-CAGTAACGGCG-AGTGAAGCGGCAACAGCTCAAATTTGAAATCTGGCCTC--GG-CCCGAGTTGTAATTTGTAGAGG-AAGCTTCTGGTGCGGTCCGTCCCGAGTCCCCTGGAACGGGGCGCCGAAGAGGGTGAGAGCCCCGTACGGACGGATACCAA-TCCTGTGTGAAGCTCCTTCGACGAGTCGAGTAGTTTGGGAATGCTGCTCAAAATGGGAGGTAAATTCCTTCTAAAGCTAAATACCGGCCAGAGACCGATAGCGCACAAGTAGAGTGATCGAAAGATGAAAAGCACTTTGAAAAGAGGGTTAAACAGCACGTGAAATTGTTGAAAGGGAAGCGCTTGTGACCAGACTTGCGCCAGGCTGATCATCCGGTGTTCTCACCGGTGCACTCGGCCTGGCTCAGGCCAGCATCGGTTCCCGCGGGGGGATAAAGGTCCCGGGAATGTAGCTCC--TCCGGGAGTG-TTATAGCCCGGGGCGCAATGCCCCCGTGGGGACCGAGGTTCGCGCATCTGCAAGGATGCTGGCGTAATGGTCATCAGCGACCCGTCTTGAAACACGGACCAAGGAGTCAAGGTTTTGCGCGAGTGTTTGGGTGTCAAACCCGCACGCGTAATGAAAGTGAACGTAGGTGAGAG--CTTCGGCGCATCATCGACCGATCCTGATGTCTTCGGATGGATTTGAGTAGGAGCGTTAAGCCTTGGACCCGAAAGATGGTGAACTATGCTTGGATAGGGTGAAGCCAGAGGAAACTCTGGTGGAGGCTCGCAGCGGTT-CTGACGTGCAAATCGATCGTCAAATCTGAGCAT-GGGGGCGAAAGACTAATCGAACCATCTAGTAGCTGGTTACCGCCGCGCTCAAGTACTCGCTGGCGACTGGAAACTGGGGTGATCAAAAGAAAGCTATGAGCTCCACTGCGGGTGTCTCGCAGGTCCTGAACCGATACACATTTGCCTCTACACTTTCTCACTTGCGGCGAACGAACACGCCTATTGGGCGCGATGGCAAGCTCGCAAAACCCCGCCAACTCCATAACACACACTGGGGTCTGGTCTGTCCAGCAGAGACGCCCGAAGGCCAGGCTTGCGGTCTGGTCAAGAATCTCTCGCTGATGTGCTACATTAGCGTTGGTACCAATGCGGATCCTATCATCGAATTCATGATTGCCAGGAACATGGAAGTCCTGGAAGAGTATGAGCCTCTGCGGTATCCGAATGCCACAAAGGTTTTCGTCAATGGCACCTGGGTCGGTGTCCACCAAGACCCAAAGCACCTGGTCAGCTTGGTTCAGAGCCTAAGGAGATCCAATATTATCAGTTTCGAGGTTTCCCTGGTTCGCGACATCCGAGACAGAGAGTTCAAGATCTTCTCCGATGCAGGGCGTGTCATGAGACCGCTATTCGTCGTTGAACAGGAGGATGACGGTGAG---------AGCAAGGTTGAGAAGGGCCAGCTGGTTCTTACCAAGTCGCAAATCCTCAAACTAGAAAAAGACAAGGAGATTGGCAAATACC------------ATCCGGATTACTTCGGCTGGAATGGCCTCTTGAGGGAAGGCTGTGTCGAATACCTGGATGCGGAAGAAGAGGAAACGGCGATGATCTGTATGACGCCCGAGGACCTCGACACGTACCGGCTGGCCAAGCT------------TGGGTTCAACGTCGCTGAGGAGGATCCTAGTGAAGGCAACAAGCGCATCAAGACTAGGCTCAACCCGACGACCCATATGTATACTCACTGCGAGATACACCCCAGCATGCTGCTTGGTATCTGCGCCAGCATCATTCCCTTCCCAGATCACAACCAGGCTTGTTGACCAAGTTCTCGATGTCGTCCGTCGCGAGGCCGAGGGCTGCGACTGCCTCCAGGGCTTCCAGATCACTCACTCTCTCGGTGGTGGTACCGGTGCCGGTATGGGTACCCTCCTGATCTCCAAGATTCGCGAGGAGTTCCCCGACCGCATGATGGCGACCTTCTCTGTCGTCCCCTCCCCCAAGGTCTCCGACACCGTCGTTGAGCCCTACAACGCCACGCTCTCCGTCCACCAGCTTGTCGAGAACTCGGACGAGACCTTCTGCATTGACAACGAGGCTCTCTACGACATCTGCATGCGGACGCTCAAGCTGTCCAACCCTTCATACGGTGATCTCAACCACCTGGTCTCTGCCGTCATGTCCGGCGTTACCGTTTCCCTGCGCTTCCCCGGCCAGCTCAACTCCGATCTCCGCAAGCTCGCCGTGAACATGGTTCCTTTCCCCCGTCTCCACTTCTTCATGGTCGGCTTCGCGCCGCTTACCAGCCGTGGCGCGCACTCTTTCCGTGCTGTCTCGGTTCCTGAGTTGACCCAGCAGATGTTCGACCCCAAGAACATGATGGCTGCGTCTGACTTCCGCAACGGTCGCTACCTGACCTGCTCTGCCATCTT

>*Jugulospora_rotula*_FMR_12690

ATTACAG-GTT-------GCAAAACTCCC--AAACCG-TTGTGAACG-TCACCGTA---TCG-TTTCTTCGGCGTGGCATCTCTGAGTAA-----CTTACAAAATAAGTTAAAACTTTCAACAACGGATCTCTTGGTTCTGGCATCGATGAAGAACGCAGCGAAATGCGATAAGTAATGTGAATTGCAGAATTCAGTGAATCATCGAATCTTTGAACGCACATTGCGCCCGCTAGTATTCTGGCGGGCATGCCTGTTCGAGCGTCATTTCAA---CCATCAAG-CCCCA---CGGCTTGTGTTGGGG-CCCTGCG-GCC-GTC-CGCAGCCCCC-GGAATGCAGTGGCGGGCTCGTT-GTCAC-CCCGAGTGCAGTAATA--CTCTTCTCTCTGGGCGTGGCGGCGGG-TTCCGGCCGTGAAACAACCAAA----CTTATCAAGGTTGACCTCGGATCAGGTAGGAATACCCGCTGAACTTAAGCATATCAATAAGCGGAGGAAAAGAAACCAACAGGG-ATTGCCC-CAGTAACGGCG-AGTGAAGCGGCAACAGCTCAAATTTGAAATCTGGCCTC--GG-CCCGAGTTGTAATTTGTAGAGG-AAGCTTCTGGTGCGGTCTGTTCCGAGTCCCCTGGAACGGGGCGCCATAGAGGGTGAGAGCCCCGTACGGACGGATACCAA-TCCTGTGTGAAGCTCCTTCGACGAGTCGAGTAGTTTGGGAATGCTGCTCAAAATGGGAGGTAAATTCCTTCTAAAGCTAAATACCGGCCAGAGACCGATAGCGCACAAGTAGAGTGATCGAAAGATGAAAAGCACTTTGAAAAGAGGGTTAAACAGCACGTGAAATTGTTGAAAGGGAAGCGCTTGTGACCAGACTTGCGCCAGGCTGATCATCCGGTGTTCTCACCGGTGCACTCTGCCTGGCTCAGGCCAGCATCGGTTTCCGCGGGGGGATAAAGGTCCCGGGAATGTAGCTCC--TCCGGGAGTG-TTATAGCCCGGGGCGCAATGCCCTCGTGGGGACCGAGGTTCGCGCATCTGCAAGGATGCTGGCGTAATGGTCATCAGCGACCCGTCTTGAAACACGGACCAAGGAGTCAAGGTTTTGCGCGAGTGTTTGGGTGTTAAACCCGCACGCGTAATGAAAGTGAACGTAGGTGAGAG--CTTCGGCGCATCATCGACCGATCCTGATGTATTCGGATGGATTTGAGTAGGAGCGTTAAGCCTTGGACCCGAAAGATGGTGAACTATGCTTGGATAGGGTGAAGCCAGAGGAAACTCTGGTGGAGGCTCGCAGCGGTT-CTGACGTGCAAATCGATCGTCAAATCTGAGCAT-GGGGGCGAAAGACTAATCGAACCATCT?????????????????GCGCTCAAATACTCGCTGGCGACTGGAAACTGGGGTGATCAAAAGAAGGCCATGAGCTCCACCGCGGGTGTCTCGCAGGTCCTGAATCGATACACATTTGCCTCTACGCTTTCTCACTTGCGGCGAACGAACACACCCATTGGGCGCGATGGCAAGCTCGCAAAACCCCGCCAACTCCATAACACACACTGGGGTCTGGTCTGTCCAGCAGAGACGCCCGAAGGCCAGGCTTGCGGTCTGGTCAAGAATCTCTCGCTGATGTGCTACATTAGCGTTGGTACCAATGCGGATCCCATCGTCGAATTTATGATTGCCAGGAACATGGAAGTCCTTGAAGAGTATGAGCCTCTGCGGTATCCCAATGCCACAAAGGTTTTCGTCAACGGCACCTGGGTCGGTGTCCACCAAGACCCAAAGCACCTGGTCAGCTTGGTTCAGAGCCTAAGGAGATCCAACATCATCAGTTTCGAGGTTTCCCTGGTTCGTGACATCCGAGACAGAGAGTTCAAGATCTTCTCCGATGCAGGGCGTGTCATGAGACCGCTATTCGTCGTTGAACAGGAGGACGACGGCGAG---------AGCAAGGTTGAGAAGGGCCAGCTGGTTCTTACAAAGTCGCAAGTCCTCAAACTAGAAAAAGACAAGGAGATTGGCAAATACC------------ATCCGGATTACTTCGGCTGGAATGGCCTCTTGAGGGAGGGCTGTGTCGAATACCTTGATGCGGAAGAAGAGGAAACGGCGATGATCTGTATGACGCCCGAGGACCTCGACACGTACCGGCTGGCCAAGCT------------TGGGTTCAACGTCGCCGAGGAAGATCCTAGTGAAGGCAACAAGCGCATCAAGACTAGGCTTAACCCGACGACCCATATGTATACTCACTGCGAGATACACCCCAGCATGCTGCTTGGTATCTGCGCCAGCATCATTCCCTTCCCCGATCACAACCAGGCTTGTTGACCAGGTTCTCGATGTCGTCCGTCGCGAGGCCGAGGGCTGCGACTGCCTCCAGGGCTTCCAGATCACCCACTCTCTCGGTGGTGGTACCGGTGCCGGTATGGGTACCCTCCTGATCTCCAAGATTCGCGAGGAGTTCCCCGACCGCATGATGGCGACCTTCTCTGTCGTCCCCTCCCCCAAGGTCTCTGATACCGTTGTGGAGCCCTACAACGCCACGCTCTCCGTCCATCAGCTTGTCGAGAACTCCGACGAGACCTTCTGCATTGACAACGAGGCTCTCTACGACATCTGCATGCGGACGCTCAAGCTGTCCAACCCTTCATACGGTGATCTCAACCACCTGGTCTCTGCCGTCATGTCCGGCGTTACCGTTTCCCTGCGCTTCCCCGGCCAGCTCAACTCAGATCTCCGCAAGCTCGCCGTGAACATGGTTCCCTTCCCCCGTCTCCACTTCTTCATGGTCGGCTTCGCGCCGCTTACTAGCCGTGGCGCGCACTCTTTCCGTGCTGTCTCGGTTCCTGAGTTGACCCAGCAGATGTTCGACCCCAAGAACATGATGGCTGCGTCTGACTTCCGCAACGGTCGCTACCTGACTTGCTCTGCCATCTT

>*Jugulospora_vestita*_13575

????????????????????????????C--ACACCA-TCGTGAACG-TCACCGCA---TCG-TTTCTTCGGCGTGGCATCTCTGAGTAG-----CTTACAAAATAAGTCAAAACTTTCAACAACGGATCTCTTGGCTCTGGCATCGATGAAGAACGCAGCGAAATGCGATAAGTAATGCGAATTGCAGAATCCAGTGAGTCATCGAATCTTTGAACGCACATTGCGCCCGCCAGTATTCTGGCGGGCATGCCTGTCCGAGCGTCATTTCCA---CCATCAAG-CCCTG---CGGCTTGTGTTGGGG-CCCTGCG-GCC-GCC-CGCAGCCCCC-GGAATGCAGTGGCGGGCTCGTT-GTCAC-CCCGAGTGCAGTAATG--CTCTTCTCTCGCGGCGTGGCGGCGGG-TTCCGGCCGTGAAACCAACCAA---ACTCATCAAGGTTGACCTCGGATCAGGTAGGAATACCCGCTGAACTTAAGCATATCAATAAGCGGAGGAAAAGAAACCAACAGGG-ATTGCCC-CAGTAACGGCG-AGTGAAGCGGCAACAGCTCAAATTTGAAATCTGGCCTC--GG-CCCGAGTTGTAATTTGCAGAGG-AAGCTTCTGGTGCGGTCTGTCCCGAGTCCCCTGGAACGGGGCGCCGGAGAGGGTGAGAGCCCCGTACGGACGGATACCAA-TCCTGTGTGAAGCTCCTTCGACGAGTCGAGTAGTTTGGGAATGCTGCTCAAAATGGGAGGTAAATTCCTTCTAAAGCTAAATACCGGCCAGAGACCGATAGCGCACAAGTAGAGTGATCGAAAGATGAAAAGCACTTTGAAAAGAGGGTTAAACAGCACGTGAAATTGTTGAAAGGGAAGCGCTTGTGACCAGACTTGCGCCAGGTCGATCATCCGGTGTTCTCACCGGTGCACTCGGCCTGGCTCAGGCCAGCATCGGTTCCCGCGGGGGGATAAAGGCCCAGGGAATGTAGCTCC--TCCGGGAGTG-TTATAGCCCGGGGCGCAATGCCCCCGTGGGGACCGAGGTTCGCGCATCTGCAAGGATGCTGGCGTAATGGTCATCAGCGACCCGTCTTGAAACACGGACCAAGGAGTCAAGGTTTTGCGCGAGTGTTTGGGTGTCAAACCCGCACGCGTAATGAAAGTGAACGTAGGTGAGAG--CTTCGGCGCATCATCGACCGATCCTGATGTCTTCGGATGGATTTGAGTAGGAGCGTTAAGCCTTGGACCCGAAAGATGGTGAACTATGCTTGGATAGGGTGAAGCCAGAGGAAACTCTGGTGGAGGCTCGCAGCGGTT-CTGACGTGCAAATCGATCGTC?????????????????????????????????????????????????????????GCGCTCAAGTACTCGCTGGCGACTGGAAACTGGGGTGATCAAAAGAAAGCCATGAGCTCCACTGCAGGTGTCTCGCAGGTCCTGAACCGATACACATTTGCCTCTACACTTTCTCACTTGCGGCGAACGAACACACCTATTGGGCGCGATGGCAAGCTCGCAAAACCCCGCCAACTCCATAACACACACTGGGGTCTGGTCTGTCCAGCAAAGACGCCCGAAGGCCAGGCTTGTGGTCTGGTCAAGAATCTCTCGCTGATGTGCTACATTAGCGTTGGTACCAATGCGGATCCTATCATCGAATTCATGATTGCCAGGAACATGGAAGTCCTGGAAGAGTATGAGCCTCTGCGGTATCCGAATGCCACAAAGGTTTTCGTCAATGGCACCTGGGTCGGTGTTCACCAAGACCCAAAGCACCTGGTCAGCTTGGTTCAGAGCCTAAGGAGATCCAATATTATCAGTTTCGAGGTTTCCCTGGTTCGCGACATCCGAGACAGAGAGTTCAAGATCTTCTCCGATGCAGGGCGTGTCATGAGACCGCTATTCGTCGTTGAACAGGAGGATGACGGTGAG---------AGCAAGGTTGAGAAGGGCCAGCTGGTTCTTACCAAGTCGCAAATCCTCAAACTAGAAAAAGACAAGGAGATTGGCAAATACC------------ATCCGGATTACTTCGGCTGGAATGGCCTCTTGAGGGAAGGCTGTGTGGAATACCTGGATGCGGAAGAAGAGGAAACGGCGATGATCTGTATGACGCCCGAGGACCTAGACACGTACCGGCTAGCCAAGCT------------TGGGTTCAACGTCGCCGAGGAGGATCCTAGTGAAGGCAACAAGCGCATCAAGACTAGGCTCAACCCGACGACCCATATGTATACTCACTGCGAGATTCACCCCAGCATGCTGCTTGGTATCTGCGCCAGCATCATTCCCTTCCCCGATCACAACCA?GCTTGTTGACCAAGTTCTTGATGTCGTCCGTCGCGAGGCCGAGGGCTGCGACTGCCTCCAGGGCTTCCAGATCACCCACTCTCTCGGTGGTGGTACCGGTGCCGGTATGGGTACCCTCCTGATCTCCAAGATTCGCGAGGAGTTCCCCGACCGCATGATGGCGACCTTCTCTGTCGTCCCCTCGCCCAAGGTCTCTGATACCGTCGTTGAGCCCTACAACGCCACGCTCTCCGTCCACCAGCTTGTTGAGAACTCCGACGAGACCTTCTGCATTGACAACGAGGCTCTCTACGACATCTGCATGCGGACGCTCAAGCTGTCCAACCCTTCATACGGTGATCTCAACCACCTGGTCTCTGCCGTCATGTCCGGCGTTACCGTTTCCCTGCGCTTCCCCGGCCAGCTCAACTCCGATCTCCGCAAGCTCGCCGTGAACATGGTTCCTTTTCCCCGTCTCCACTTCTTCATGGTCGGCTTCGCGCCGCTTACCAGCCGTGGCGCGCACTCTTTCCGTGCTGTCTCGGTTCCTGAGTTGACCCAGCAGATGTTCGACCCCAAGAACATGATGGCTGCGTCCGACTTCCGCAACGGTCGCTATCTGACCTGCTCTGCCATCTT

>*Lundqvistomyces_karachiensis*_CBS_657.74

ATTAGCGA----------GTAATACTCTCTAACACCA-TTGTGAACG-AACCGAATT--TAG-TTGCTTCGGCGTTGTATCTCTGAGTTA-----ACTTTTTAATAAGTTAAAACTTTCAACAACGGATCTCTTGGTTCTGGCATCGATGAAGAACGCAGCGAAATGCGATAAGTAATGTGAATTGCAGAATTCAGTGAATCATCGAATCTTTGAACGCACATTGCGCCCGCCAGCACTCTGGCGGGCATGCCTGTTCGAGCGTCATTTCAA---CCATCAAG-CCCCA----GGCTTGCGTTGGGG-GCCTGCG-GCT-GCC--GCAGCCCCC-TAAAAGCAGTGGCGGGCTCGCT-GTCAT-TCCGAGCGCAGTAGTT-ACATCTCGCTCTGGGCGTGGCGGCGGG-CACCGGCCGTAAAACACTCTTT--------CTAAGGTTGACCTCGGATCAGGTAGGAATACCCGCTGAACTTAAGCATATCAATAAGCGGAGGAAAAGAAACCAACAGGG-ATTGCCC-TAGTAACGGCG-AGTGAAGCGGCAACAGCTCAAATTTGAAATCTGGCTTC--GG-CCCGAGTTGTAATTTGTAGAGG-AAGCTTCTGGCGCGGTGCCGTCCGAGTCCCCTGGAACGGGGCGCCATAGAGGGTGAGAGCCCCGTATGGACGGATGCTAA-GCCTGTGTGAAGCTCCTTCGACGAGTCGAGTAGTTTGGGAATGCTGCTCAAAATGGGAGGTAAATTCCTTCTAAAGCTAAATATTGGCCAGAGACCGATAGCGCACAAGTAGAGTGATCGAAAGATGAAAAGCACTTTGAAAAGAGGGTTAAACAGCACGTGAAATTGTTGAAAGGGAAGCGCTTGTGACCAGACTTGCGCCAGGTTGATCATCCGGTGTTCTCACCGGTGCACTCTGCCTGGCACAGGCCAGCATCAGTTTTGGCGGGGGGATAAAGGGCGCTTGAACGTAGCTCC--TCCGGGAGTG-TTATAGCGTGCGTCGTAATACCCTCGCCGGGACTGAGGACCGCGCATCTGCAAGGATGCTGGCGTAATGGTCACCAGCGACCCGTCTTGAAACACGGACCAAGGAGTCAAGGTTTTGCGCGAGTGTTTGGGTGTTAAACCCGCACGCGTAATGAAAGTGAACGTAGGTGAGAG--CTTCGGCGCATCATCGACCGATCCTGATGTATTCGGATGGATTTGAGTAAGAGCGTTAAGCCTTGGACCCGAAAGATGGTGAACTATGCTTGGATAGGGTGAAGCCAGAGGAAACTCTGGTGGAGGCTCGCAGCGGTT-CTGACGTGCAAATCGATCGTCAAATCTGAGCAT-GGGGGCGAAAGACTAATCGAACCATCTAGTAGCTGGTTACCGCCGGTCTCAAGTACTCGTTGGCCACAGGCAACTGGGGTGACCAGAAGAAGGCGATGAGCTCAACAGCAGGTGTCTCGCAGGTGTTGAACCGTTACACTTTCGCGTCGACCCTCTCGCATTTGCGTCGCACCAATACTCCCATCGGCCGCGATGGCAAGCTCGCTAAGCCTCGACAACTTCACAACACCCATTGGGGTCTGGTCTGCCCTGCAGAAACGCCAGAGGGTCAAGCTTGCGGCCTGGTCAAGAACCTCTCTTTGATGTGCTACATCAGCGTTGGCACGAATGCTGATCCTATTATCGACTTTATGATAGCCAGAAACATGGAAGTCCTCGAGGAGTACGAGCCGCTTCGATATCCCAACGCAACCAAGGTCTTCGTCAACGGAACCTGGGTTGGTGTTGTCCAGGACCCGAAGAATCTCGTTGGCCTTGTCCAGAACCTTAGAAGGTCAAATATCATTTCCTTCGAGGTCTCACTGGTTCGCGATATAAGAGATCGAGAGTTCAAGATTTTCTCGGATGCCGGTCGTGTAATGCGACCGTTGTTCGTAGTGGAGCAAGAAGATGATAACCCG------CAGACAAAGGTCTCGAGAGGGCAGCTGGTGTTGAGAAAGGAGCACATTGAGCGATTGGAGCGCGACAGAGAGATTGGAAGATTCG------------ACCCCAATTACTACGGGTGGGATGGCCTTCTTCGAGACGGATGCGTCGAGTACCTCGACGCCGAGGAAGAAGAAACCACCATGATCTGCATGTCGCCTGAAGATCTCGACCATTTCCGAATGACGAAAGT------TTTGGGCATCACGCCTCACGAAGTGGAAGACCCTAGTGCGGGCAACAAGCGCATCCCTACCAAGCTCAACCCGACCACTCATGCGTACACGCATTGCGAGAT?????????????????????????????????????????????????????????????????????????????????????????????????????????????????????????????TTCCAGATCACCCACTCCCTCGGTGGTGGTACCGGTGCCGGTATGGGTACCCTACTTATCTCCAAGATCCGCGAGGAGTTCCCTGACCGCATGATGGCGACTTTCTCCGTCGTCCCCTCCCCCAAGGTCTCGGATACCGTCGTCGAGCCCTACAACGCCACCCTCTCCGTGCACCAGCTTGTTGAGAACTCGGACGAGACCTTCTGCATTGACAACGAGGCTCTCTACGACATCTGCATGAGGACACTCAAGCTCTCCAACCCCTCGTATGGTGACCTTAACCACCTCGTCTCCGCTGTCATGTCCGGTGTCACCGTCTCCCTCCGTTTCCCCGGTCAGCTCAACTCTGATCTCCGCAAGCTCGCCGTGAACATGGTGCCCTTCCCTCGTCTGCACTTCTTTATGGTTGGCTTTGCCCCTCTTACTAGCCGTGGCGCCCACTCTTTCCGTGCCGTCTCGGTTCCCGAGCTCACCCAGCAGATGTTCGACCCCAAGAACATGATGGCTGCTTCTGATTTCCGCAACGGTCGCTACCTCACTTGCTCCGCCATCTT

>*Lundqvistomyces_tanzaniensis*_TRTC51981

ATTAGCGA----------GTAAAACTCTCTAACACCA-TTGTGAACG-AACCGAATT--TAG-TTGCTTCGGCGTTGTATCTCTGAGTCA----ACTTTTTTAATAAGTTAAAACTTTCAACAACGGATCTCTTGGTTCTGGCATCGATGAAGAACGCAGCGAAATGCGATAAGTAATGTGAATTGCAGAATTCAGTGAATCATCGAATCTTTGAACGCACATTGCGCCCGCCAGCACTCTGGCGGGCATGCCTGTTCGAGCGTCATTTCAA---CCATCAAG-CCCCA----GGCTTGCGTTGGGG-GCCTGCG-GCT-GCC--GCAGCCCCC-TAAAAGCAGTGGCGGGCTCGCT-GTCAT-TCCGAGCGCAGTAGTT-ACATCTCGCTCTGGGCGTGGCGGCGGG-CACCGGCCGTAAAACACTCTTT--------CTAAGGTTGACC????????????????????????????????????????????????????AAAGAAACCAACAGGG-ATTGCCC-TAGTAACGGCG-AGTGAAGCGGCAACAGCTCAAATTTGAAATCTGGCTTC--GG-CCCGAGTTGTAATTTGTAGAGG-AAGCTTCTGGCGCGGTGCCGTCCGAGTCCCCTGGAACGGGGCGCCATAGAGGGTGAGAGCCCCGTATGGACGGATGCTAA-GCCTGTGTGAAGCTCCTTCGACGAGTCGAGTAGTTTGGGAATGCTGCTCAAAATGGGAGGTAAATTCCTTCTAAAGCTAAATATTGGCCAGAGACCGATAGCGCACAAGTAGAGTGATCGAAAGATGAAAAGCACTTTGAAAAGAGGGTTAAACAGCACGTGAAATTGTTGAAAGGGAAGCGCTTGTGACCAGACTTGCGCCAGGTTGATCATCCGGTGTTCTCACCGGTGCACTCTGCCTGGCACAGGCCAGCATCAGTTTTGGCGGGGGGATAAAGGGCGCTTGAAAGTAGCTCC--TCCGGGAGTG-TTATAGCTTGCGTCGTAATACCCTCGCCGGGACTGAGGACCGCGCATCTGCAAGGATGCTGGCGTAATGGTCACCAGCGACCCGTCTTGAAACACGGACCAAGGAGTCAAGGTTTTGCGCGAGTGTTTGGGTGTTAAACCCGCACGCGTAATGAAAGTGAACGTAGGTGAGAG--CTTCGGCGCATCATCGACCGATCCTGATGTATTCGGATGGATTTGAGTAAGAGCGTTAAGCCTTGGACCCGAAAGATGGTGAACTATGCTTGGATAGGGTGAAGCCAGAGGAAACTCTGGTGGAGGCTCGCAGCGGTT-CTGACGTGCAAATCGATCGTCAAATCTGAGCAT-GGGGGCGAAAGACTAATCGAACCATCTAGTAGCTGGTTACCGCCGGTCTCAAGTACTCATTGGCCACAGGCAACTGGGGTGACCAGAAGAAGGCGATGAGCTCGACAGCAGGTGTCTCGCAGGTGTTGAACCGTTACACTTTCGCGTCGACCCTCTCGCATTTGCGTCGCACCAATACTCCCATCGGCCGCGATGGCAAGCTTGCTAAGCCTCGACAACTTCACAACACCCATTGGGGTCTGGTCTGCCCTGCAGAAACGCCAGAGGGTCAAGCTTGCGGCCTGGTCAAGAACCTCTCTTTGATGTGCTACATCAGCGTTGGCACAAATGCTGATCCTATTATTGACTTTATGATCGCCAGAAACATGGAAGTCCTCGAGGAGTACGAGCCGCTTCGATATCCCAACGCAACCAAGGTCTTCGTCAATGGAACCTGGGTTGGTGTTGTCCAGGACCCGAAGAATCTCGTTGGCCTTGTCCAGAACCTTAGAAGGTCAAATATCATTTCCTTCGAGGTCTCCCTGGTTCGCGATATAAGAGATCGAGAGGTCAAGATTTTCTCGGATGCCGGTCGTGTAATGCGACCGTTGTTCGTAGTGGAGCAAGAAGATGATAACCCG------CAGACAAAGGTCTCGAGAGGGCAGCTGGTGTTGAGAAAGGAGCACATTGAGCGATTGGAGCGCGACAGGGAGATTGGAAGATTCG------------ACCCCAATTACTACGGGTGGGATGGCCTTCTTCGAGACGGATGCGTCGAGTACCTCGACGCCGAGGAAGAAGAAACCACCATGATCTGCATGTCGCCTGAAGATCTCGACCATTTCCGAATGACGAAAGT------TTTGGGCATCACGCCTCACGAAGTGGAAGACCCTAGTGCGGGCAACAAGCGCATCCCTACCAAGCTCAACCCGACCACGCATGCGTACACGCATTGCGAGATCCATCCCAGTATGCTGCTCGGCATTTGCGCCAGCATCATTCCGTTCCCGGATCACAACCAGGCTTGTTGACCAAGTCCTCGATGTTGTCCGTCGTGAGGCTGAGGGCTGCGACTGCCTCCAGGGCTTCCAGATCACCCACTCCCTCGGTGGTGGTACCGGTGCCGGTATGGGTACCCTACTTATCTCCAAGATCCGCGAGGAGTTCCCTGACCGCATGATGGCGACTTTCTCCGTCGTCCCCTCCCCCAAGGTCTCGGATACCGTCGTCGAGCCCTACAATGCCACCCTCTCCGTTCACCAGCTTGTTGAGAACTCGGACGAGACCTTCTGCATTGACAACGAGGCTCTCTACGACATCTGCATGAGGACACTCAAGCTCTCCAACCCCTCGTATGGTGACCTTAACCACCTCGTCTCCGCTGTCATGTCCGGTGTCACCGTCTCCCTCCGTTTCCCCGGTCAGCTCAACTCCGATCTCCGCAAGCTCGCCGTGAACATGGTGCCCTTCCCTCGTCTGCACTTCTTCATGGTTGGCTTTGCCCCTCTTACTAGCCGTGGCGCCCACTCTTTCCGTGCCGTCTCGGTTCCCGAGCTCACCCAGCAGATGTTTGACCCCAAGAACATGATGGCTGCTTCTGATTTCCGCAACGGTCGCTACCTCACTTGCTCCGCCATCTT

>*Morinagamyces_vermicularis*_CBS_303.81

ATTACAGAGTT-------GCAAAACTCCC---AACCA-TTGTGAACG-AACCGCT--------GTGCTCAGGCGGTGGATATCTGAGTAG-----CTCATTCAATGAGTCAAAACTTTCAACAACGGATCTCTTGGTTCTGGCATCGATGAAGAACGCAGCGAAATGCGATAAGTAATGTGAATTGCAGAATTCAGTGAATCATCGAATCTTTGAACGCACATTGCGCCCGCTAGTATTCTGGCGGGCATGCCTGTTCGAGCGTCATTTCAA---CCATCAAG-CCCC----GGGCTTGTGTTGGGG-ACCTGCG-GCT-GCC--GCAGCCCCC-TAAAAGCAGTGGCGGTCTCGCT-GTCAC-ACCGAGCGCAGTAGTGTAC-CTCCGCTCGGGGAGTGGCGGCGGGTTGCCTGCCGTGAAACACACCTA-----------AGGTTGACCTCGGATCAGGTAGGAATACCCGCTGAACTTAAGCATATCAATAAGCGGAGGAAAAGAAACCAACAGGG-ATTGCCT-CAGTAACGGCG-AGTGAAGCGGCAACAGCTCAAATTTGAAATCTGGCTTC--GG-CCCGAGTTGTAATTTGCAGAGG-AAGCTTCTGGCGCAGCGCCATCCGAGTCCCCTGGAACGGGGCGCCACAGAGGGTGAGAGCCCCGTATGGATGGACGCCTA-GCCTGTGTGAAGCTCCTTCGACGAGTCGAGTAGTTTGGGAATGCTGCTCAAAATGGGAGGTAAATTCCTTCTAAAGCTAAATACCGGCCAGAGACCGATAGCGCACAAGTAGAGTGATCGAAAGATGAAAAGCACTTTGAAAAGAGGGTTAAATAGCACGTGAAATTGTTGAAAGGGAAGCGCTCATGACCAGACTTGCGCCAGGCTGATCATCCGGTGTTCTCACCGGTGCACTCTGCCTGGCTCAGGCCAGCATCGGTTTCGGCGGGGGGATAAAGGCCTAGGGAACGTAGCTCC--TCCGGGAGTG-TTATAGCCCTGGGTGCAATGCCCCCGCTGGGACCGAGGTTCGCGC-TCTGCAAGGATGCTGGCGTAATGGTCATCAGCGACCCGTCTTGAAACACGGACCAAGGAGTCAAGGTTTTGCGCGAGTGTTTGGGTGTCAAACCCGCACGCGTAATGAAAGTGAACGTAGGTGAGAG--CTTCGGCGCATCATCGACCGATCCTGATGTATTCGGATGGATTTGAGTAGGAGCGTTAAGCCTTGGACCCGAAAGATGGTGAACTATGCTTGGATAGGGTGAAGCCAGAGGAAACTCTGGTGGAGGCTCGCAGCGGTT-CTGACGTGCAAATCGATCGTCA????????????????????????????????????????????????????????GGCCTGAAGTACTCGCTCGCCACTGGCAACTGGGGTGACCAGAAGAAGGCCATGAGCTCCACCGCTGGCGTGTCCCAGGTCTTGAACCGATACACCTTCGCCTCGACCCTCTCTCACTTGCGGCGAACCAACACCCCCATCGGCCGCGACGGGAAGCTGGCGAAACCCCGTCAGCTCCACAACACGCACTGGGGCTTGGTCTGCCCTGCCGAGACTCCCGAAGGCCAGGCCTGCGGTCTGGTCAAGAACCTGTCTCTCATGTGTTACATCAGTGTGGGCACTAATGCTGAACCCATTATCGACTTCATGGTTGCTAGGAACATGGAAGTACTCGAAGAATACGAACCTTTGAGGTACCCCAATGCCACGAAAGTCTTTGTCAATGGAACCTGGGTCGGTATCCATCAAGAGCCTAAGCATCTGGTCAACCTTGTCCAGGGCCTGAGACGACTCAACATTATCTCTTTTGAGGTCTCGCTTGTTAGAGATATCCGAGACCGGGAGTTCCAGATCTTCTCGGACGCCGGCCGTGTCATGCGACCGCTGTTCGTTGTCGCGCAGGAAGCGGATCCCGAG---------CGAAAACTCGAGCAGGGCCAGCTCGTCCTCACAAAGGAACATATCCGCCGTCTGGAACATGACAAGGAGATTGGCCGAGAGC------------ACCCCGACCATTTCGGCTGGGATGGTTTGCTTCGAGAGGGGTGCGTCGAGTATCTCGATGCCGAAGAGGAGGAGACGTCGATGATCTGCATGTCGCCCGAGGACTTGTCCGACTATCGACTCACCAAGCT------------TGGTTTCCACGTTGTCGAGGAAGACTCGAGTGAAGGCAATCGGCGCATCAAGACCAAGATGAACCCCACGACACACATGTACACGCATTGCGAGATCCACCCCAGCATGTTGCTCGGCATTTGTGCTAGCATCATTCCCTTCCCCGACCACAACCAGGCTTGTTGACCAAGTTCTCGATGTCGTCCGTCGCGAGGCTGAGGGTTGCGACTGCCTCCAGGGCTTCCAGATCACCCACTCCCTCGGTGGTGGTACCGGTGCCGGTATGGGTACGCTCCTCATCTCCAAGATCCGCGAGGAGTTCCCCGACCGCATGATGGCCACTTTCTCGGTCGTCCCCTCGCCCAAGGTGTCAGATACCGTCGTCGAGCCTTACAACGCCACTCTTTCGGTCCACCAGCTCGTTGAGAACTCGGACGAGACCTTCTGCATTGACAACGAGGCTCTGTACGACATTTGCATGAGGACTCTGAAGCTGTCCAACCCCTCGTACGGCGACCTTAACCACCTGGTCTCGGCTGTCATGTCGGGCGTCACTGTCTCGCTGCGCTTCCCCGGTCAGCTCAACTCGGACCTCCGCAAGCTTGCTGTCAACATGGTTCCTTTCCCGCGTCTCCACTTCTTCATGGTTGGCTTCGCTCCTCTTACCAGCCGTGGCGCGCACTCTTTCCGTGCCGTTTCGGTTCCTGAGCTCACGCAGCAAATGTTCGACCCCAAGAACATGATGGCTGCTTCTGACTTCCGCAACGGTCGCTACCTTACATGCTCTGCCATCTT

>*Podospora_bullata*_CBS_115576

ATTAGCGAGTT-------GCAAGACTCCC-AAACCCT-CTGTGAATG-CCCTGGA----CCG-CTGCTTCGGCGTGGCATCTCTGAGTAC------TCTTATAATGAGTCAAAACTTTCAACAACGGATCTCTTGGTTCTGGCATCGATGAAGAACGCAGCGAAATGCGATAAGTAATGTGAATTGCAGATTTCAGTGAATCATCGAATCTTTGAACGCACATTGCGCCCGCCAGTATTCTGGCGGGCATGCCTGTTCGAGCGTCATTTCAA--CCCATCAAGCCCCTG----CGCTTGCGTTGGAG-CCCTGCG-GCC-GCC--GCAGCCTCC-CAAAGACAGTGGCGGGCTCGCT-ATCAC-ACCGAGTGCAGTAGATTTCTCCTCGCTCAGGGCGT-GTGGCGGG-TGCCGGCCGTGAAACCCCCCAA---GCTTTCAAAGGTTGACCTCGGATCAGGTAGGAATACCCGCTGAACTTAAGCATATCAATAAGCGG????????????????????????????????????GGCG-AGTGAAGCGGCAACAGCTCAAATTTGAAATCTGGCTCC--GG-CCCGAGTTGTAATTTGCAGAGG-AAGCTTCTGGTGACGCGCCGTCTAAGTCCCCTGGAACGGGGCGCCGCAGCGGGTGAGAGCCCCAT-CTGACGACCGCTGA-CCCAGTGTGAAGCTCCTTCGACGAGTCGAGTAGTTTGGGAATGCTGCTCAAAATGGGAGGTAAATTCCTTCTAAAGCTAAATACCGGCCAGAGACCGATAGCGCACAAGTAGAGTGATCGAAAGATGAAAAGCACTTTGAAAAGAGGGTTAAACAGCACGTGAAATTGTTGAAAGGGAAGCGCTCATGACCAGACTTGCGCTGGGCTGATCATCCGGTGTTCTCACCGGTGCACTCTGCCCAGCTCAGGCCAGCATCGGTTTCGGCGGGGGGATAAAGGCGACGGGAACGTAGCTCC--TCCGGGAGTG-TTATAGCCCGGCGTGCAATACCCCCGCTGGGACCGAGGTCCGCGC-TCTGCAAGGATGCTGGCGTAATGGTCATCAGCGACCCGTCTTGAAACACGGACCAAGGAGTCAAGGTTTTGCGCGAGTGTTTGGGTGTCAAACCCGCACGCGTAATGAAAGTGAACGTAGGTGAGAG--CTTCGGCGCATCATCGACCGATCCTGATGTTCTCGGACGGATTTGAGTAGGAGCGTTAAGCCTTGGACCCGAAAGATGGTGAACTATGCTTGGATAGGGTGAAGCCAGAGGAAACTCTGGTGGAGGCTCGCAGCGGTT-CTGACGTGCAAATCGATCGTCAAATCTGAGCAT?????????????????????????????????????????????????????????????????????????????????????????????????????????????????????????????????????????????????????????????????????????????????????????????????????????????????????????????????????????????????????????????????????????????????????????????????????????????????????????????????????????????????????????????????????????????????????????????????????????????????????????????????????????????????????????????????????????????????????????????????????????????????????????????????????????????????????????????????????????????????????????????????????????????????????????????????????????????????????????????????????????????????????????????????????????????????????????????????????????????????????????????????????????????????????????????????????????????????????????????????????????????????????????????????????????????????????????????????????????????????????????????????????????????????????????????????????????????????????????????????????????????????????????????????????????????????????????????????????????????????????????????????????????????????????????????????????????????????????????????????????????????????????????????????????????????????????????????????????????????????????????????????????????????????????????????????????????????????????????????????????????????????????????????????????????????????????????????????????????????????????????????????????????????????????????????????????????????????????????????????????????????????????????????????????????????????????????????????????????????????????????????????????????????????????????????????????????????????????????????????????????????????????????????????????????

>*Podospora_serotina*_CBS_252.71

ATTACCGAGTT------CTTACAGAACCC--AACCCT-GTGTGATAA-GTTCTCAC---CCGAACTTTTCCACTACAAATATCTGAGCGA----CTTTACATAAAGAGTCAAAACTTTCAACAACGGATCTCTTGGTTCTGGCATCGATGAAGAACGCAGCGAAATGCGATACGTAATGTGAATTGCAGAATTCAGTGAATCATCGAATCTTTGAACGCACATTGCGCCCGCCAGTATTCTGGCGGGCATGCCTGTCCGAGCGTCATTTCAA--CCCATCAAG-CCCAG----CGCTTGTGTTGGAG-CCCTGCG-GCC-GCC--GCAGCCTCC-CAAAATTAGTGGCGGGCTCGCT-ATCAC-GCTGAGTGCAGTAGTATTCTTCTCACTCCTGCGGT-GTAGCGGGTTACCAGCCGTAAAACCCTTTTA-------CCTAAAGTTGACCTCGGATCAGGTAGGAATACCCGCTGAACTTAAGCATATCA?????????????????????????????????????????AACGGCG-AGTGAAGCGGCAACAGCTCAAATTTGAAATCTGGCAAC--AG-CCCGAGTTGTAATTTGCAGAGG-AAGCTTCTGGCGACGCACTGTCTAAGTCCCCTGGAACGGGGCGCCACAGCGGGTGAGAGCCCCATGTGAT-GGCTGCGGA-CCCAGTGTGAAGCTCCTTCGACGAGTCGAGTAGTTTGGGAATGCTGCTCAAAATGGGAGGTAAATTCCTTCTAAAGCTAAATATTGGCCAGAGACCGATAGCGCACAAGTAGAGTGATCGAAAGATGAAAAGCACTTTGAAAAGAGGGTTAAACAGCACGTGAAATTGTTGAAAGGGAAGCGCTCATGACCAGACTTGCGCTGGGCTGATCATCCGGTGTTCTCACCGGTGCACTCTGCCCGGCTCAGGCCAGCATCGGTTTTGGCGGGGGGATAAAGGCGCCGGGAACGTAGCTCC--TCCGGGAGTG-TTATAGCCCGGTGTGCAATACCCCCGCTGGGACCGAGGTCCGCGCATC-GCAAGGATGCTGGCGTAATGGTCATCAGCGACCCGTCTTGAAACACGGACCAAGGAGTCAAGGTTTTGCGCGAGTGTTTGGGTGTCAAACCCGCRCGCGTAATGAAAGTGAACGTAGGTGAGAG--CTTCGGCGCATCATCGACCGATCCTGAAGTTTACGGACGGATTTGAGTAGGAGCGTTAAGCCTTGGACCCGAAAGATGGTGAACTATGCTTGGATAGGGTGAAGCCAGAGGAAACTCTGGTGGAGGCTCGCAGCGGTT-CTGACGTGCAAATCGATCGTCAAATCTGAGCAT-GGG?????????????????????????????????????????????????????????????????????????????????????????????????????????????????????????????????????????????????????????????????????????????????????????????????????????????????????????????????????????????????????????????????????????????????????????????????????????????????????????????????????????????????????????????????????????????????????????????????????????????????????????????????????????????????????????????????????????????????????????????????????????????????????????????????????????????????????????????????????????????????????????????????????????????????????????????????????????????????????????????????????????????????????????????????????????????????????????????????????????????????????????????????????????????????????????????????????????????????????????????????????????????????????????????????????????????????????????????????????????????????????????????????????????????????????????????????????????????????????????????????????????????????????????????????????????????????????????????????????????????????????????????????????????????????????????????????????????????????????????????????????????????????????????????????????????????????????????????????????????????????????????????????????????????????????????????????????????????????????????????????????????????????????????????????????????????????????????????????????????????????????????????????????????????????????????????????????????????????????????????????????????????????????????????????????????????????????????????????????????????????????????????????????????????????????????????????????????????????????????????????????????????????????????????????????

>*Pseudoechria_curvicolla*_CBS_259.69

ATTACCGAGTT------CTTACAGAACCC--AACCCT-GTGTGATAA-GTTCTCAC---CCGAACTTTTCCACTACAAATATCTGAGCGA----CTTTACATAAAGAGTCAAAACTTTCAACAACGGATCTCTTGGTTCTGGCATCGATGAAGAACGCAGCGAAATGCGATACGTAATGTGAATTGCAGAATTCAGTGAATCATCGAATCTTTGAACGCACATTGCGCCCGCCAGTATTCTGGCGGGCATGCCTGTCCGAGCGTCATTTCAA--CCCATCAAG-CCCAG----CGCTTGTGTTGGAG-CCCTGCG-GCC-GCC--GCAGGCTCC-CAAAATTAGTGGCGGGCTCGCT-ATCAC-GCTGAGTGCAGTAGTATTCTTCTCACTCCTGCGGT-GTAGCGGGTAACCAGCCGTAAAACCCTTTTA-------CCTAAAGTTGACCTCGGATCAGGTAGGAATACCCGCTGAACTTAAGCATATCAATAAG???????AAAGAAACCAACAGGG-ATTGCCC-TAGTAACGGCG-AGTGAAGCGGCAACAGCTCAAATTTGAAATCTGGCAAC--AG-CCCGAGTTGTAATTTGCAGAGG-AAGCTTCTGGCGACGCACTGTCTAAGTCCCCTGGAACGGGGCGCCACAGCGGGTGAGAGCCCCATGTGAT-GGCTGCGGA-CCCAGTGTGAAGCTCCTTCGACGAGTCGAGTAGTTTGGGAATGCTGCTCAAAATGGGAGGTAAATTCCTTCTAAAGCTAAATATTGGCCAGAGACCGATAGCGCACAAGTAGAGTGATCGAAAGATGAAAAGCACTTTGAAAAGAGGGTTAAACAGCACGTGAAATTGTTGAAAGGGAAGCGCTCATGACCAGACTTGCGCTGGGCTGATCATCCGGTGTTCTCACCGGTGCACTCTGCCCGGCTCAGGCCAGCATCGGTTTTGGTGGGGGGATAAAGGCGCCGGGAACGTAGCTCC--TCCGGGAGTG-TTATAGCCCGGTGTGCAATACCCCCGCTGGGACCGAGGTCCGCGCATC-GCAAGGATGCTGGCGTAATGGTCATCAGCGACCCGTCTTGAAACACGGACCAAGGAGTCAAGGTTTTGCGCGAGTGTTTGGGTGTCAAACCCGCACGCGTAATGAAAGTGAACGTAGGTGAGAG--CTTCGGCGCATCATCGACCGATCCTGAAGTTTACGGACGGATTTGAGTAGGAGCGTTAAGCCTTGGACCCGAAAGATGGTGAACTATGCTTGGATAGGGTGAAGCCAGAGGAAACTCTGGTGGAGGCTCGCAGCGGTT-CTGACGTGCAA-TCGATCGTCAA-TCTGA?????????????????????????????????????????????????????????????????????????????????????????????????????????????????????????????????????????????????????????????????????????????????????????????????????????????????????????????????????????????????????????????????????????????????????????????????????????????????????????????????????????????????????????????????????????????????????????????????????????????????????????????????????????????????????????????????????????????????????????????????????????????????????????????????????????????????????????????????????????????????????????????????????????????????????????????????????????????????????????????????????????????????????????????????????????????????????????????????????????????????????????????????????????????????????????????????????????????????????????????????????????????????????????????????????????????????????????????????????????????????????????????????????????????????????????????????????????????????????????????????????????????????????????????????????????????????????????????????????????????????????????????????????????????????????????????????????????????????????????????????????????????????????????????????????????????????????????????????????????????????????????????????????????????????????????????????????????????????????????????????????????????????????????????????????????????????????????????????????????????????????????????????????????????????????????????????????????????????????????????????????????????????????????????????????????????????????????????????????????????????????????????????????????????????????????????????????????????????????????????????????????????????????????????????????????????

>*Pseudoechria_decidua*_CBS_254.71

ATTACCGAGTT-------CTTACAGAACC-CAACCCT-GTGTGATAA-GTTCTCAC---CCGAACTTTTCCACTACAAATTTCTGAGCGA----CTTTACATAAAGAGTCAAAACTTTCAACAACGGATCTCTTGGTTCTGGCATCGATGAAGAACGCAGCGAAATGCGATACGTAATGTGAATTGCAGAATTCAGTGAATCATCGAATCTTTGAACGCACATTGCGCCCGCCAGTATTCTGGCGGGCATGCCTGTCCGAGCGTCATTTCAA--CCCATCAAG-CCCAG----CGCTTGTGTTGGAG-CCCTGCG-GCC-GCC--GCAGCCTCC-CAAAATTAGTGGCGGGCTCGCT-ATCAC-GCTGAGTGCAGTAGTATTCTTCTCACTCCTGTGGT-GTAGCGGGTAACCAGCCGTAAAAACCCCCTT----ACTTTAAAAGTTGACCTCGGATCAGGTAGGAATACCCGCTGAACTTAAGCATATCATA??????????AAAGAAACCAACAGGG-ATTGCCC-TAGTAACGGCG-AGTGAAGCGGCAACAGCTCAAATTTGAAATCTGGCAAC--AG-CCCGAGTTGTAATTTGCAGAGG-AAGCTTCTGGCGACGCACTGTCTAAGTCCCCTGGAACGGGGCGCCACAGCGGGTGAGAGCCCCATGTGAT-GGCTGCGGA-CCCAGTGTGAAGCTCCTTCGACGAGTCGAGTAGTTTGGGAATGCTGCTCAAAATGGGAGGTAAATTCCTTCTAAAGCTAAATATTGGCCAGAGACCGATAGCGCACAAGTAGAGTGATCGAAAGATGAAAAGCACTTTGAAAAGAGGGTTAAACAGCACGTGAAATTGTTGAAAGGGAAGCGCTCATGACCAGACTTGCGCTGGGCTGATCATCCGGTGTTCTCACCGGTGCACTCTGCCCGGCTCAGGCCAGCATCGGTTTTGGCGGGGGGATAAAGACGCCGGGAACGTAGCTCC--CCCGGGAGTG-TTATAGCCCGGCGTGCAATACCCCCGCTGGGACCGAGGTCCGCGCATC-GCAAGGATGCTGGCGTAATGGTCATCAGCGACCCGTCTTGAAACACGGACCAAGGAGTCAAGGTTTTGCGCGAGTGTTTGGGTGTCAAACCCGCACGCGTAATGAAAGTGAACGTAGGTGAGAG--CTTCGGCGCATCATCGACCGATCCTGAAGTTTACGGACGGATTTGAGTAGGAGCGTTAAGCCTTGGACCCGAAAGATGGTGAACTATGCTTGGATAGGGTGAAGCCAGAGGAAACTCTGGTGGAGGCTCGCAGCGGTT-CTGACGTGCAAATCGATCGTCAAATCTGAGCAT-GGGGGCGAAAGACTAATCGAACCATCTAGTAGCTGG-TACCGC????????????????????????????????????????????????????????????????????????????????????????????????????????????????TCCCATTTGCGGCGAACAAATACCCCCATCGGCCGCGATGGCAAGCTGGCTAAACCCCGACAGCTGCACAACACCCACTGGGGTCTCGTGTGCCCTGCAGAGACCCCAGAAGGACAAGCCTGCGGTCTCGTCAAGAATCTCTCCTTGATGTGCTATATTAGCGTCGGCACGAACGCAGAACCCATCGTCGACTTTATGACAGCTAGGAACATGGAACTTTTGGAAGAATACGAGCCGCTACGCTATCCCAATGCCACCAAGGTCTTCGTCAACGGAACGTGGGTGGGCGTCCATCAGGATCCAAAGCACCTGGTCAACCTGGTCCAAGGTCTGAGGAGAGCGGGCGTCATTAGTTTCGAGGTTTCGCTCGTCCGAGACATCCGCGACCGAGAGTTCAAGATCTTCTCCGATGCTGGCCGTGTCATGAGGCCGCTGTTTGTTGTCGCGCAGGACGATGACAAAGAG---AAGAATGGCGGCGTCGAAAAGGGACAGCTTGTTCTGACAAGACAACACATTGACCGGCTGGCACGGGACAAGGAAATCGGTAATTTGG------------ACGAGAACTACCTGGGTTGGAATGGACTCCTTAGAGAGGGTTGCGTCGAGTACCTGGACGCCGAGGAGGAGGAGACGACCATGATCTGCATGAGCCCCGAGGACCTGGATGCTTTCCGGCTCACCAAGCT------------TGGTCATAATCCAAACGAGGAGGAGTCTGGCGGGCCCAACAAGCGTATCAAGACGAAGGTCAACCCGACGACCCACATGTATACGCATTGCGAGATTCACCCCAGCATGCTGCTTGGTATCTGCGCCAGCATCATTCCCTTCCCCGACCACAACCAA??????????????????????????????????????????????????????????????????????????????????????????????????????????????????????????????????????????????????????????????????????????????????????????????????????????????????????????????????????????????????????????????????????????????????????????????????????????????????????????????????????????????????????????????????????????????????????????????????????????????????????????????????????????????????????????????????????????????????????????????????????????????????????????????????????????????????????????????????????????????????????????????????????????????????????????????????????????

>*Pseudoechria_longicollis*_CBS_368.52

ATTACCGAGTT------CTTACAGAACCC--AACCCT-GTGTGATAA-GTTCTCAC---CCGAACTTTTCCACTACAAATATCTGAGCGA----CTTTACATAAAGAGTCAAAACTTTCAACAACGGATCTCTTGGTTCTGGCATCGATGAAGAACGCAGCGAAATGCGATACGTAATGTGAATTGCAGAATTCAGTGAATCATCGAATCTTTGAACGCACATTGCGCCCGCCAGTATTCTGGCGGGCATGCCTGTCCGAGCGTCATTTCAA--CCCATCAAG-CCCAG----CGCTTGTGTTGGAG-CCCTGCG-GCC-GCC--GCAGGCTCC-CAAAATTAGTGGCGGGCTCGCT-ATCAC-GCTGAGTGCAGTAGTATTCTTCTCACTCCTGCGGT-GTAGCGGGTAACCAGCCGTAAAACCCTTTTA-------CCTAAAGTTGACCTCGGATCAGGTAGGAATACCCGCTGAACTTAAGCATATCAATAAGCGGAGGAAAAGAAACCAACAGGG-ATTGCCC-TAGTAACGGCG-AGTGAAGCGGCAACAGCTCAAATTTGAAATCTGGCAAC--AG-CCCGAGTTGTAATTTGCAGAGG-AAGCTTCTGGCGACGCACTGTCTAAGTCCCCTGGAACGGGGCGCCACAGCGGGTGAGAGCCCCATGTGAT-GGCTGCGGA-CCCAGTGTGAAGCTCCTTCGACGAGTCGAGTAGTTTGGGAATGCTGCTCAAAATGGGAGGTAAATTCCTTCTAAAGCTAAATATTGGCCAGAGACCGATAGCGCACAAGTAGAGTGATCGAAAGATGAAAAGCACTTTGAAAAGAGGGTTAAACAGCACGTGAAATTGTTGAAAGGGAAGCGCTCATGACCAGACTTGCGCTGGGCTGATCATCCGGTGTTCTCACCGGTGCACTCTGCCCGGCTCAGGCCAGCATCGGTTTTGGTGGGGGGATAAAGGCGCCGGGAACGTAGCTCC--TCCGGGAGTG-TTATAGCCCGGTGTGCAATACCCCCGCTGGGACCGAGGTCCGCGCATC-GCAAGGATGCTGGCGTAATGGTCATCAGCGACCCGTCTTGAAACACGGACCAAGGAGTCAAGGTTTTGCGCGAGTGTTTGGGTGTCAAACCCGCACGCGTAATGAAAGTGAACGTAGGTGAGAG--CTTCGGCGCATCATCGACCGATCCTGAAGTTTACGGACGGATTTGAGTAGGAGCGTTAAGCCTTGGACCCGAAAGATGGTGAACTATGCTTGGATAGGGTGAAGCCAGAGGAAACTCTGGTGGAGGCTCGCAGCGGTT-CTGACGTGCAAATCGATCGTCAAATCTGAGCAT-GGGGGCGAAAGACTAATCGAACCATCTAGTAGCTGGGTACCGCA????????????????????????????????????????????????????????????????????????????????????????????????????????????????????????????????????ACCCCCATCGGCCGCGATGGCAAGCTGGCTAAACCCCGACAGCTGCACAACACCCACTGGGGTCTCGTGTGCCCTGCAGAGACCCCAGAAGGACAAGCTTGCGGTCTCGTCAAAAATCTTTCCTTAATGTGCTACATTAGTGTCGGCACGAACGCAGAACCCATCGTCGACTTCATGACAGCTAGGAACATGGAACTTTTGGAGGAATATGAGCCGCTACGCTATCCCAATGCCACCAAGGTCTTCGTCAACGGAACGTGGGTGGGCGTCCATCAGGACCCCAAGCATCTGGTCACCCTGGTCCAAGGTCTGAGGAGAGCTGGCGTCATTAGTTTCGAGGTTTCGCTCGTCCGAGACATCCGCGACCGAGAGTTCAAGATCTTCTCCGATGCTGGCCGTGTCATGAGGCCGCTGTTTGTTGTCGCGCAGGACGATGACAAAGAG---AAGAATGGCGGCGTGGAAAAGGGACAGCTTGTTCTCACAAGACAACACATTGACCGGCTGGCACGGGACAAGGAAATCGGAAATTTGG------------ACGAGAACTACCTGGGCTGGAATGGTCTTCTGAGAGAGGGTTGCGTCGAGTATCTGGACGCCGAGGAAGAGGAGACGACCATGATCTGCATGAGCCCCGAGGACCTGGATGCTTTCCGGCTCACCAAGCT------------TGGTCATAATCCAAACGACGACGAGTCCGGCGGACCTAACAAGCGTATCAAGACGAAGGTGAACCCGACGACTCACATGTATACGCATTGCGAGATTCACCCCAGCATGCTGCTTGGTATCTGCGCCAGCATCATTCCCTTCCCCGACCACAACCAA??????????????????????????????????????????????????????????????????????????????????????????????????????????????????????????????????????????????????????????????????????????????????????????????????????????????????????????????????????????????????????????????????????????????????????????????????????????????????????????????????????????????????????????????????????????????????????????????????????????????????????????????????????????????????????????????????????????????????????????????????????????????????????????????????????????????????????????????????????????????????????????????????????????????????????????????????????????

>*Pseudoechria_prolifica*_CBS_250.71

ATTACCGAGTT------CTTACAGAACCC--AACCCT-GTGTGATAA-GTTCTCAC---CCGAACTTTTCCACTACAAATATCTGAGCGA----CTTTACATAAAGAGTCAAAACTTTCAACAACGGATCTCTTGGTTCTGGCATCGATGAAGAACGCAGCGAAATGCGATACGTAATGTGAATTGCAGAATTCAGTGAATCATCGAATCTTTGAACGCACATTGCGCCCGCCAGTATTCTGGCGGGCATGCCTGTCCGAGCGTCATTTCAA--CCCATCAAG-CCCAG----CGCTTGTGTTGGAG-CCCTGCG-GCC-GCC--GCAGCCTCC-CAAAATTAGTGGCGGGCTCGCT-ATCAC-GCTGAGTGCAGTAGTATTCTTCTCACTCCTGCGGT-GTAGCGGGTTACCAGCCGTAAAACCCTTTTA-------CCTAAAGTTGACCTCGGATCAGGTAGGAATACCCGCTGAACTTAAGCATATCAATAAGCGGAGGAAAAGAAACCAACAGGG-ATTGCCC-TAGTAACGGCG-AGTGAAGCGGCAACAGCTCAAATTTGAAATCTGGCAAC--AG-CCCGAGTTGTAATTTGCAGAGG-AAGCTTCTGGCGACGCACTGTCTAAGTCCCCTGGAACGGGGCGCCACAGCGGGTGAGAGCCCCATGTGAT-GGCTGCGGA-CCCAGTGTGAAGCTCCTTCGACGAGTCGAGTAGTTTGGGAATGCTGCTCAAAATGGGAGGTAAATTCCTTCTAAAGCTAAATATTGGCCAGAGACCGATAGCGCACAAGTAGAGTGATCGAAAGATGAAAAGCACTTTGAAAAGAGGGTTAAACAGCACGTGAAATTGTTGAAAGGGAAGCGCTCATGACCAGACTTGCGCTGGGCTGATCATCCGGTGTTCTCACCGGTGCACTCTGCCCGGCTCAGGCCAGCATCGGTTTTGGCGGGGGGATAAAGGCGCCGGGAACGTAGCTCC--TCCGGGAGTG-TTATAGCCCGGTGTGCAATACCCCCGCTGGGACCGAGGTCCGCGCATC-GCAAGGATGCTGGCGTAATGGTCATCAGCGACCCGTCTTGAAACACGGACCAAGGAGTCAAGGTTTTGCGCGAGTGTTTGGGTGTCAAACCCGCACGCGTAATGAAAGTGAACGTAGGTGAGAG--CTTCGGCGCATCATCGACCGATCCTGAAGTTTACGGACGGATTTGAGTAGGAGCGTTAAGCCTTGGACCCGAAAGATGGTGAACTATGCTTGGATAGGGTGAAGCCAGAGGAAACTCTGGTGGAGGCTCGCAGCGGTT-CTGACGTGCAAATCGATCGTCAAATCTGAGCAT-GGGGGCGAAAGACTAATCGAACCATCTAGTAGCTGGTTACCGCG?????????????????????????????????????????????????????????????????????????????????????????????????????????ACACTGTCGCATTTGCGGCGAACAAATACCCCCATCGGCCGCGATGGCAAGCTGGCTAAACCCCGACAGCTGCACAACACCCACTGGGGTCTCGTGTGCCCTGCAGAGACCCCGGAAGGACAAGCTTGCGGTCTCGTCAAAAATCTTTCCTTAATGTGCTACATTAGTGTCGGCACGAACGCAGAACCCATCGTCGACTTCATGACAGCTAGGAACATGGAACTTCTGGAGGAATACGAGCCGCTGCGCTATCCCAATGCCACCAAGGTCTTCGTCAACGGAACGTGGGTGGGCGTCCATCAGGACCCCAAGCATCTGGTCACCCTGGTCCAAGGTTTGAGGAGAGCTGGCGTCATTAGTTTCGAGGTTTCGCTCGTCCGAGACATCCGCGACCGAGAGTTCAAGATCTTTTCCGATGCCGGCCGTGTCATGAGGCCGCTGTTTGTTGTCGCGCAGGACGACGACAAAGAG---AAGAATGGCGGCGTCGAAAAGGGACAGCTTGTTCTCACAAGACAACACATTGACCGGCTGGCACGGGACAAGGAAATCGGGAATTTGG------------ACGAGAACTACCTGGGCTGGAATGGTCTTCTGAGAGAGGGTTGCGTCGAGTATCTGGACGCCGAGGAAGAGGAGACGACCATGATCTGCATGAGCCCCGAGGACCTGGATGCTTTCCGGCTCACCAAGCT------------TGGTCATAATCCAAACGACGACGAGTCCGGCGGACCCAACAAGCGTATCAAGACGAAGGTCAACCCGACGACTCACATGTATACGCATTGCGAGATTCACCCCAGCATGCTGCTTGGCATCTGCGCCAGCATCATTCCCTTCCCCGACCACAACCAA??????????????????????????????????????????????????????????????????????????????????????????????????????????????????????????????????????????????????????????????????????????????????????????????????????????????????????????????????????????????????????????????????????????????????????????????????????????????????????????????????????????????????????????????????????????????????????????????????????????????????????????????????????????????????????????????????????????????????????????????????????????????????????????????????????????????????????????????????????????????????????????????????????????????????????????????????????????

>*Pseudoschizothecium_atropurpureum*_SMH2961

???????????????????????????????????????????????????????????????????????????????????????????????????????????????????????????????????????????????????????????????????????????????????????????????????????????????????????????????????????????????????????????????????????????????????????????????????????????????????????????????????????????????????????????????????????????????????????????????????????????????????????????????????????????????????????????????????????????????????????????????????????????????????????AAAGAAACCAACAGGG-ATTGCCC-TAGTAACGGCG-AGTGAAGCGGCAACAGCTCAAATTTGAAATCTGGCTCC--GG-CCCGAGTTGTAATTTGCAGAGG-AAGCATTTGGTGCGGTATTCTCCGAGTCCCCTGGAATGGGGCGCCAGAGAGGGTGAGAGCCCCGTAGGATTGAATACCTA-GCCTGTGTGAAGCTCCTTCGACGAGTCGAGTAGTTTGGGAATGCTGCTCTAAATGGGAGGTAAATTCCTTCTAAAGCTAAATATTGGCCAGAGACCGATAGCGCACAAGTAGAGTGATCGAAAGATGAAAAGCACTTTGAAAAGAGGGTTAAATAGCACGTGAAATTGTTGAAAGGGAAGCGCCTGTGACCAGACTTGCGCCCAGCGGATCATCCGGTGTTCTCACCGGTGCACTCCGCTTGGCTCAGGCCAGCATCGGTTCTGGTGGGGGGATAAAGGTTCCGGGAATGTAGCTCT--TTCGGGAGTG-TTATAGCCCGGGGCGCAATGCCCTCGCCGGGACCGAGGACCGCGCGAAAGCAAGGATGCTGGCGTAATGGTCACCAGCGACCCGTCTTGAAACACGGACCAAGGAGTCAAGGTTTTGCGCGAGTGTTTGGGTGTCAAACCCGCACGCGTAATGAAAGTGAACGTAGGTGAGAG--CTTCGGCGCATCATCGACCGATCCTGATGTACTCGGATGGATTTGAGTAGGAGCGTTAAGCCTTGGACCCGAAAGATGGTGAACTATGCTTGGATAGGGTGAAGCCAGAGGAAACTCTGGTGGAGGCTCGCAGCGGTT-CTGACGTGCAAATCGATCGTCAAATCTGAGCAT-GGGGGCGAAAGACTAATCGAACCATCTAGTAGCTGGTTACCGCC??????????????????????????????????????????????????????????????????????????????????????????????????????????????????????????????????????????????????????????????????????????????????????????????????????????????????????????????????????????????????????????????????????????????????????????????????????????????????????????????????????????????????????????????????????????????????????????????????????????????????????????????????????????????????????????????????????????????????????????????????????????????????????????????????????????????????????????????????????????????????????????????????????????????????????????????????????????????????????????????????????????????????????????????????????????????????????????????????????????????????????????????????????????????????????????????????????????????????????????????????????????????????????????????????????????????????????????????????????????????????????????????????????????????????????????????????????????????????????????????????????????????????GCTTGTTGACCAAGTTCTCGACGTCGTCCGCCGTGAGGCCGAGGGCTGCGACTGCCTCCAGGGTTTCCAGATCACCCACTCGCTCGGTGGTGGTACTGGTGCTGGTATGGGTACCCTTCTCATCTCCAAGATTCGCGAGGAGTTCCCCGACCGCATGATGGCGACTTTCTCCGTCGTCCCCTCGCCCAAGGTGTCCGACACCGTCGTCGAGCCCTACAACGCTACCCTCTCCGTTCACCAGCTCGTTGAGAACTCGGACGAGACCTTCTGTATTGATAACGAGGCTCTGTACGATATCTGCATGCGCACCCTGAAGCTGTCCAACCCCTCGTATGGCGACCTCAACCACCTCGTTTCGGCTGTCATGTCGGGCGTCACGGTCTCGCTCCGTTTCCCCGGCCAGCTTAACTCGGATCTCCGCAAGCTCGCCGTCAACATGGTGCCTTTCCCTCGTCTGCACTTCTTCATGGTTGGCTTTGCGCCCCTGACTAGCCGCGGCGCCTACACTTTCCGTGCCGTCTCGGTTCCGGAGCTCACCCAGCAGATGTTCGACCCCAAGAACATGATGGCTGCTTCTGACTTCCGCAATGGCCGCTACCTCACTTGCTCAGCCATTTT

>*Pseudoschizothecium_atropurpureum*_SMH3073

???????????????????????????????????????????????????????????????????????????????????????????????????????????????????????????????????????????????????????????????????????????????????????????????????????????????????????????????????????????????????????????????????????????????????????????????????????????????????????????????????????????????????????????????????????????????????????????????????????????????????????????????????????????????????????????????????????????????????????????????????????????????????????AAAGAAACCAACAGGG-ATTGCCC-TAGTAACGGCG-AGTGAAGCGGCAACAGCTCAAATTTGAAATCTGGCTCC--GG-CCCGAGTTGTAATTTGCAGAGG-AAGCATTTGGTGCGGTATTCTCCGAGTCCCCTGGAATGGGGCGCCAGAGAGGGTGAGAGCCCCGTAGGATTGAATACCTA-GCCTGTGTGAAGCTCCTTCGACGAGTCGAGTAGTTTGGGAATGCTGCTCTAAATGGGAGGTAAATTCCTTCTAAAGCTAAATATTGGCCAGAGACCGATAGCGCACAAGTAGAGTGATCGAAAGATGAAAAGCACTTTGAAAAGAGGGTTAAATAGCACGTGAAATTGTTGAAAGGGAAGCGCCTGTGACCAGACTTGCGCCCAGCGGATCATCCGGTGTTCTCACCGGTGCACTCCGCTTGGCTCAGGCCAGCATCGGTTCTGGTGGGGGGATAAAGGTTCCGGGAATGTAGCTCT--TTCGGGAGTG-TTATAGCCCGGGGCGCAATGCCCTCGCCGGGACCGAGGACCGCGCGAAAGCAAGGATGCTGGCGTAATGGTCACCAGCGACCCGTCTTGAAACACGGACCAAGGAGTCAAGGTTTTGCGCGAGTGTTTGGGTGTCAAACCCGCACGCGTAATGAAAGTGAACGTAGGTGAGAG--CTTCGGCGCATCATCGACCGATCCTGATGTACTCGGATGGATTTGAGTAGGAGCGTTAAGCCTTGGACCCGAAAGATGGTGAACTATGCTTGGATAGGGTGAAGCCAGAGGAAACTCTGGTGGAGGCTCGCAGCGGTT-CTGACGTGCAAATCGATCGTCAAATCTGAGCAT-GGGGGCGAAAGACTAATCGAACCATCTAGTAGCTGGTTACCGCCGCTCTGAAATACTCACTCGCGACTGGCAATTGGGGAGATCAAAAGAAAGCAATGAACTCGACCGCCGGTGTCTCTCAAGTTTTGAACAGATATACGTTTGCTTCGACCCTCTCTCATTTGCGTCGGACAAATACACCCATCGGACGTGATGGCAAGCTTGCCAAACCCCGCCAGCTTCACAACACGCACTGGGGCTTAGTTTGCCCCGCCGAGACCCCAGAAGGACAGGCTTGCGGTTTGGTCAAGAATCTATCGCTCATGTGTTACGTCAGTGTTGGCACCAATGCCGAACCGATTGTCGACTTCATGGTGCAGAGAGGCATGGAAGTGCTGGAAGAATACGAGCCCTTGCGATATCCAAATTCCACCAAGGTTTTCGTCAACGGGACTTGGGTGGGTGTCCACCCCGATCCAAAGCACCTGGTTACCGCGGTCCAGGGTCTACGGCGTAGCAACCTTATATCATTCGAGGTTTCGCTCGTGAGAGATATCAGGGACCGCGAGTTCCAAATCTTCTCCGATGCTGGCCGTGTCATGAGGCCGTTATTTGTGGTAGAGCAGGAAGACAGTGGCGAA---------CATGGAGTTGAGAAGGGCCAGCTGGTTCTCACGAAATCCATGGTCCGCGAGCTTGAACGAAGCAAGGGGCTGGGCAAGTTCC------------ACCCTGATTACAAGGGCTGGGAGTGGCTCCTCGGGCAGGGAGCTATTGAGTATTTGGATGCCGAAGAGGAGGAAACGGCCATGATCTGCATGACTCCAGAGGATCTCGACAATTATCGGCTCACTCGTCT------------GGGCTTGGAGATGCCCGAAGAAGA---GGAGGGGAACGCCAAGCGTATCAAGACGAGGATGAACCCGACGACGCACATGTATACGCATTGCGAAATCCACCCGGCTATGCTGCTGGGAATTTGCGCCAGCATTATCCCCTTCCCGGATCACAACCAAGCTTGTTGACCAAGTTCTCGACGTCGTCCGCCGTGAGGCCGAGGGCTGCGACTGCCTCCAGGGTTTCCAGATCACCCACTCGCTCGGTGGTGGTACTGGTGCTGGTATGGGTACCCTTCTCATCTCCAAGATTCGCGAGGAGTTCCCCGACCGCATGATGGCGACTTTCTCCGTCGTCCCCTCGCCCAAGGTGTCCGACACCGTCGTCGAGCCCTACAACGCTACCCTCTCCGTTCACCAGCTCGTTGAGAACTCGGACGAGACCTTCTGTATTGATAACGAGGCTCTGTACGATATCTGCATGCGCACCCTGAAGCTGTCCAACCCCTCGTATGGCGACCTCAACCACCTCGTTTCGGCTGTCATGTCGGGCGTCACGGTCTCGCTCCGTTTCCCCGGCCAGCTTAACTCGGATCTCCGCAAGCTCGCCGTCAACATGGTGCCTTTCCCTCGTCTGCACTTCTTCATGGTTGGCTTTGCGCCCCTGACTAGCCGCGGCGCCTACACTTTCCGTGCCGTCTCGGTTCCGGAGCTCACCCAGCAGATGTTCGACCCCAAGAACATGATGGCTGCTTCTGACTTCCGCAATGGCCGCTACCTCACTTGCTCAGCCATTTT

>*Ramophialophora_chlamydospora*

??????????????????????????????????????????????????????????????????????????CGGCCTCTCTGAGTACG----ATTTTTAAACAAGTTAAAACTTTCAACAACGGATCTCTTGGTTCTGGCATCGATGAAGAACGCAGCGAAATGCGATAAGTAATGTGAATTGCAGAATTCAGTGAATCATCGAATCTTTGAACGCACATTGCGCCCGCTAGTATTCTGGCGGGCATGCCTGTCCGAGCGTCATTTCAACCCCCCTCAAGCCCTCTCCCCGGCTTGGTGCTGAGGCCCTGCG-GCT-GTT-CGCAGGCCTC-GAAAACTAGTGGCGGGCTCGCTAGTCAC-CCCGAGCGCAGTAGCA--CATCTCGCTTAGGGCGT-GCAGCGGGTCTTTTGCCGTGAAACACCCCCCCCTTACCACAAAGGTTGACCTCGGATCAGGTAGGAGTACCCGCTGAACTTAAGCATATCAATAAGCGGAGGAA?????????????????????????????????????????????????????????????????????????????????????????????????????????????????????????????????????????????????????????????????????????????????????????????????????????????????????????????????????????????????????????????????????????????????????????????????????????????????????????????????????????????????????????????????????????????????????????????????????????????????????????????????????????????????????????????????????????????????????????????????????????????????????????????????????????????????????????????????????????????????????????????????????????????????????????????????????????????????????????????????????????????????????????????????????????????????????????????????????????????????????????????????????????????????????????????????????????????????????????????????????????????????????????????????????????????????????????????????????????????????????????????????????????????????????????????????????????????????????????????????????????????????????????????????????????????????????????????????????????????????????????????????????????????????????????????????????????????????????????????????????????????????????????????????????????????????????????????????????????????????????????????????????????????????????????????????????????????????????????????????????????????????????????????????????????????????????????????????????????????????????????????????????????????????????????????????????????????????????????????????????????????????????????????????????????????????????????????????????????????????????????????????????????????????????????????????????????????????????????????????????????????????????????????????????????????????????????????????????????????????????????????????????????????????????????????????????????????????????????????????????????????????????????????????????????????????????????????????????????????????????????????????????????????????????????????????????????????????????????????????????????????????????????????????????????????????????????????????????????????????????????????????????????????????????????????????????????????????????????????????????????????????????????????????????????????????????????????????????????????????????????????????????????????????????????????????????????????????????????????????????????????????????????????????????????????????????????????????????????????????????????????????????????????????????????????????????????????????????????????????????????????????????????????????????????????????????????????????????????????????

>*Ramophialophora_petraea*_LC6222

????????????????????????????????A-CCA-TCGCGAACGTTACCCGTAA--CCG-TTGCCTCGGCGAGGCCTCTCTGAGTAA-----CTTATCAAATAAGTCAAAACTTTTAACAACGGATCTCTTGGTTCTGGCATCGATGAAGAACGCAGCGAAATGCGATAAGTAATGTGAATTGCAGAATTCAGTGAATCATCGAATCTTTGAACGCACATTGCGCCCGCCAGTATTCTGGCGGGCATGCCTGTTCGAGCGTCATTTCAA---CCATCAAG-CCCCCG---GGCTTGCGTTGGGG-CCCTGCG-GCT-GCC--GCAGACCCC-TAAAACCAGTGGCGGGCTCGCT-GTCAC-ACCGAGCGCAGTAGCA-ATATCTCGCTCTGGGCGT-GGCGCGGG-TGCTTGCCGTAAAACACACCAC-----TTAACAAGGTTGACCTCGGATCAGGTAGGAATACCCGCTGAACTTAAGCATATCAATAAGCGGAGGAAAAGAAACCAACAGGG-ATTGCCC-CAGTAACGGCG-AGTGAAGCGGCAACAGCTCAAATTTGAAATCTGGCCTC--GG-CCCGAGTTGTAATTTGTAGAGG-AAGCTTCTGGTGCGGCACCGGCTGAGTCCCCTGGAACGGGGCGCCATAGAGGGTGAGAGCCCCGTATAGCCGGCTGCCTA-GCCTGTGTGAAGCTCCTTCGACGAGTCGAGTAGTTTGGGAATGCTGCTCAAAATGGGAGGTAAATTCCTTCTAAAGCTAAATATTGGCCAGAGACCGATAGCGCACAAGTAGAGTGATCGAAAGATGAAAAGCACTTTGAAAAGAGGGTTAAACAGCACGTGAAATTGTTAAAAGGGAAGCGCTTGTGACCAGACTTGCGCCGGGGCGATCATCCGGTGTTCTCACCGGTGCACTCGCCCCGGCTCAGGCCAGCATCGGTTTTCGCGGGGGGATAAAGGCACCGGGAACGTAGCTCC--TCCGGGAGTG-TTATAGCCCGGGGCGTAATGCCCCCGCGGGGACCGAGGACCGCGCATCTGCAAGGATGCTGGCGTAATGGTCACCAGCGACCCGTCTTGAAACACGGACCAAGGAGTCAAGGTTTTGCGCGAGTGTTTGGGTGTAAAACCCGCACGCGTAATGAAAGTAAACGTAGGTGAGAG--CTTCGGCGCATCATCGACCGATCCTGATGTATTCGGATGGATTTGAGTAGGAGCGTTAAGCCTTGGACCCGAAAGATGGTGAACTATGCTTGGATAGGGTGAAGCCAGAGGAAACTCTGGTGGAGGCTCGCAGCGGTT-CTGACGTGCAAATCGATCGTCAAATCTGAGCAT-GGGGGCGAAAGACTAATCGAACCATCTAG???????????????GCGCTCAAGTACTCGCTGGCCACCGGAAACTGGGGAGACCAGAAGAAGGCCGCGAGCTCCACCGCTGGCGTGTCGCAGGTGCTGAACCGGTACACGTTTGCCTCGACCCTATCCCATTTGAGGCGGACAAACACGCCGATTGGGCGCGACGGAAAGCTTGCCAAGCCGCGTCAGCTTCACAATACGCATTGGGGCTTGGTCTGCCCAGCCGAGACGCCCGAGGGCCAAGCTTGCGGTCTGGTCAAGAATCTGTCCCTCATGTGCTACGTGAGCGTGGGGACACCCGCGGAGCCCATCGTCGAATTCATGATTGCGAGAAACATGGAAGTTCTGGAGGAGTACGAGCCGTTGCGGTACCCCAACGCCACCAAGGTATTCGTCAACGGCACCTGGGTCGGCGTCCACCACGACGCCAAGCAGTTGGTGGGTCTGGTGCAAGGTCTGCGGCGAAAGAACGTCATCTCGTTCGAGGTATCGCTCGTCCGGGACATTCGCGACCGCGAATTCAAGGTGTTTTCCGACGCAGGCCGTGTCATGAGGCCGCTTTTCGTTGTCGAGCAGGAAGAGAACAGCGAC---------AGCGGCGTTCAGCCCGGACAGCTTCTGCTCAAGAAGGAGCATGTGCAGCAGCTGGAAGAGGACAAGGCGCTTGGGAAGGGTC------------ATCCCGACTACTGGGGCTGGGATGGTCTTCTCAGGTCTGGTGCGATCGAATACCTGGATGCCGAGGAGGAAGAGACATCCATGATTTGCATGTCTCCCGAGGACCTTGATACCTATCGGATGGCCAAGTT------------GGGATACAACGT---CGAGGAGAGCGATCCCGCCGGAGGCAACAAGCGGATCAAGACCAAGCTGAACCCTACCACGCATCGCTACACGCATTGCGAGATCCACCCCAGCATGCTGCTCGGTATTTGCGCCAGCATC???????????????????????????????????????????????????????????????????????????????????????????????????????????????????????????????????????????????????????????????????????????????????????????????????????????????????????????????????????????????????????????????????????????????????????????????????????????????????????????????????????????????????????????????????????????????????????????????????????????????????????????????????????????????????????????????????????????????????????????????????????????????????????????????????????????????????????????????????????????????????????????????????????????????????????????????????????????????????????????????

>*Rinaldiella_pentagonospora*_CBS_132344

ATTACAGGGTAGCTTTATGCTGTAAACCCCCAA-CCA-TTGTGAACG-AAGCTTATTCGCAG-TTGCCTCAGCGTGTCATCTCTGAGTAA-----CTTTT--AATAAGTCAAAACTTTCAACAACGGATCTCTTGGTTCTGGCATCGATGAAGAACGCAGCGAAATGCGATAAGTAATGTGAATTGCAGAATTCAGTGAATCATCGAATCTTTGAACGCACATTGCGCCCGCTAGTATTCTGGCGGGCATGCCTGTTCGAGCGTCATTTCAA---CCATCAAG-CCCTA----GGCTTGTGTTGGGG-CTCTGCG-GCT-GCC--GCAGTCCCC-TAAAAGCAGTGGCGGACTCGCT-GTCAT-ACCGAGCGCAGTAGCA--TATCTCGCTCTGGGCGTGGCGGCGGG-TACCGGCCGTTAAACAATCCAT---------CAAGGTTGACCTCGGATCAGGTAGGAATACCCGCTGAACTTAAGCATATCAA???????????AAAGAAACCAACAGGG-ATTGCCC-TAGTAACGGCG-AGTGAAGCGGCAACAGCTCAAATTTGAAATCTGGCTTC--GG-CCCGAGTTGTAATTTGCAGAGG-AAGCTTCTGGCGCGGCGCTGTCCGAGTCCCCTGGAACGGGGCGCCATAGAGGGTGAGAGCCCCGTATGGATGGATGCCTA-GCCTGTGTGAAGCTCCTTCGACGAGTCGAGTAGTTTGGGAATGCTGCTCAAAATGGGAGGTAAATTCCTTCTAAAGCTAAATATTGGCCAGAGACCGATAGCGCACAAGTAGAGTGATCGAAAGATGAAAAGCACTTTGAAAAGAGGGTTAAATAGCACGTGAAATTGTTGAAAGGGAAGCGCTTGTGACCAGACTTGCGCCAGGCTGATCATCCGGTGTTCTCACCGGTGCACTCTGCCTGGCTCAGGCCAGCATCGGTTTTGGTGGGGGGATAAAGACCTAGGGAACGTAGCTCT--TTCGGGAGTG-TTATAGCCCTGGGTGTAATGCCCCCGCTGGGACCGAGGTTCGCGCATCTGCAAGGATGCTGGCGTAATGGTCATCAGCGACCCGTCTTGAAACACGGACCAAGGAGTCAAGGTTTTGCGCGAGTGTTTGGGTGTTAAACCCGCACGCGTAATGAAAGTGAACGTAGGTGAGAG--CTTCGGCGCATCATCGACCGATCCTGATGTATTCGGATGGATTTGAGTAGGAGCGTTAAGCCTTGGACCCGAAAGATGGTGAACTATGCTTGGATAGGGTGAAGCCAGAGGAAACTCTGGTGGAGGCTCGCAGCGGTT-CTGACGTGCAA???????????????????????????????????????????????????????????????????GGGCTGAAGTACTCGCTCGCAACTGGTAACTGGGGTGATCAGAAGAAGGCGATGAGCTCTACTGCCGGTGTTTCGCAAGTGTTGAACCGATATACATTTGCTTCCACACTTTCCCACTTGCGGCGTACCAACACACCCATCGGTCGCGACGGGAAACTCGCCAAACCCCGACAACTGCACAACACTCACTGGGGCTTGGTCTGCCCCGCAGAGACCCCCGAAGGCCAAGCATGCGGTCTTGTGAAGAACCTTTCTCTCATGTGCTACGTCAGCGTCGGCACTAATGCCGAGCCGATTGTCGATTTCATGATAGCTCGGAATATGGAAGTCCTCGAGGAGTATGAACCCTTGCGGTACCCGAATGCTACCAAAGTCTTCGTCAACGGCACCTGGGTTGGTGTTCACCAAGACCCGAAGCACTTGGTCGGTTTGGTGCAAGGCCTGAGAAGAACCAACGTCATCTCTTTTGAGGTCTCGCTAGTGAGAGACATTCGAGACAGGGAGTTCAAGATCTTCTCGGACGCCGGCCGAGTTATGCGACCGCTCTTTGTTGTGGAGCAAGAGGACGACAATCCC---------GCCAAGATTGAGAAGGGACAGTTGGTCTTGACGAAATCACACGTACAGCGTCTGGAGAACGATAAGGTAATTGGTCGATACC------------ACAAGGATTACTTCGGTTGGGATGGCCTGGTGAGGGAAGGCTGCGTCGAGTATCTAGATGCCGAGGAGGAGGAGACAACCATGATTTGCATGACCCCTGAGGACCTGGACACCTACCGCTTGACGAAGCT?????????????????????????????????????????????????????????????????????????????????????????????????????????????????????????????????????????????????????????????????????????GCTTGTCGACCAAGTTCTCGATGTCGTCCGTCGCGAGGCCGAGGGCTGCGACTGCCTCCAGGGCTTCCAGATTACCCACTCACTCGGCGGTGGTACCGGTGCTGGTATGGGCACCCTCCTTATCTCCAAGATTCGCGAGGAGTTCCCCGACCGCATGATGGCGACTTTCTCGGTCGTTCCCTCACCCAAGGTGTCGGATACCGTTGTTGAGCCCTACAACGCCACTCTCTCCGTCCACCAACTCGTTGAGAACTCGGATGAGACCTTCTGCATTGACAACGAGGCTCTTTACGACATCTGCATGCGGACGCTCAAGCTGTCCAACCCCTCGTACGGCGACCTCAACCACCTGGTCTCTGCCGTCATGTCGGGCGTCACCGTTTCTCTCCGTTTCCCCGGCCAGCTCAACTCTGACCTCCGCAAGCTTGCCGTCAACATGGTTCCCTTCCCGCGTCTCCACTTCTTCATGGTGGGCTTCGCTCCTCTTACCAGCCGTGGCGCGCACTCTTTCCGTGCCGTCTCGGTGCCCGAGCTTACCCAGCAGATGTTCGACCCCAAGAACATGATGGCCGCTTCTGACTTCCGCAACGGTCGCTACCTTACCTGCTCTGCCATTTT

>*Schizothecium_aloides*_CBS_879.72

ATTACAGAGTT-------GCGAAACTCCC--AA-CCA-TTGTGAACC-TACCTCA----CCG-TTGCTTCGGCGTGGCCTCTCTGAGTAA-----CTTATACAATAAGTCAAAACTTTCAACAACGGATCTCTTGGTTCTGGCATCGATGAAGAACGCAGCGAAATGCGATACGTAATGTGAATTGCAGATTTCAGTGAATCATCGAATCTTTGAACGCACATTGCGCCCGCTAGTATTCTGGCGGGCATGCCTGTTCGAGCGTCATTTCAA---CCATCAAG-CCCCC---GGGCTTGCGTTGGAG-CCCTGCG-GCT-GCC--GCAGGCTCC-CAAATCCAGTGGCGGGCTCGTC-GTCGT-ACCGAGTGCAGTAAAC--ATCCTCGCTCAGGGAAC-GCGTCGGT--TCTTGCCGTGAAACCCCCCCT-----ATATCAAGGTTGACCTCGGATCAGGTAGGAATACCCGCTGAACTTAAGCATATCAATAAGCGGAGGAA???????CAACAGGG-ATTGCCC-CAGTAACGGCG-AGTGAAGCGGCAACAGCTCAAATTTGAAATCTGGCCTC--GG-CCCGAGTTGTAATTTGTAGAGG-AAGCTTCTGGTGCGGCGCCGTCCGAGTCTCCTGGAACGGAGCGCCATAGAGGGTGAGAGCCCCGTATGGTCGGATGCCAA-ACCTGTGTGAAGCTCCTTCGACGAGTCGAGTAGTTTGGGAATGCTGCTCTAAATGGGAGGTAAATTCCTTCTAAAGCTAAATATTGGCCAGAGACCGATAGCGCACAAGTAGAGTGATCGAAAGATGAAAAGCACTTTGAAAAGAGGGTTAAACAGCACGTGAAATTGTTGAAAGGGAAGCGCTTGTGACCAGACTTGAGGGCGGCGGATCATCCGGTGTTCTCACCGGTGCACTCCGCCGCTCCCAGGCCAGCATCGGTTTCCGCGGGGGGACAAAGGTCCCGGGAACGTAGCTCC--TCCGGGAGTG-TTATAGCCCGGGGCGCAATGCCCCCGCGGGGACCGAGGACCGCGCATCTGCAAGGATGCTGGCGTAATGGTCATCAGCGACCCGTCTTGAAACACGGACCAAGGAGTCAAGGTTTTGCGCGAGTGTTTGGGTGTCAAACCCGCACGCGTAATGAAAGTGAACGTAGGTGAGAG--CTTCGGCGCATCATCGACCGATCCTGATGTATTCGGATGGATTTGAGTAGGAGCGTTAAGCCTTGGACCCGAAAGATGGTGAACTATGCTTGGATAGGGTGAAGCCAGAGGAAACTCTGGTGGAGGCTCGCAGCGGTT-CTGACGTGCAAATCGATCGTCAAATCTGAGCAT-GGGGGCGAAAGACTAATCGA????????????????????????????????????????????????????????????????????????????????????????????????????????????????????????????????????????????????????????????????????????????????????????????????????????????????????????????????????????????????????????????????????????????????????????????????????????????????????????????????????????????????????????????????????????????????????????????????????????????????????????????????????????????????????????????????????????????????????????????????????????????????????????????????????????????????????????????????????????????????????????????????????????????????????????????????????????????????????????????????????????????????????????????????????????????????????????????????????????????????????????????????????????????????????????????????????????????????????????????????????????????????????????????????????????????????????????????????????????????????????????????????????????????????????????????????????????????????????????????????????????????????????????????????????????????????????????????????????????????????????????????????????????????????????????????????????????????????????????????????????????????????????????????????????????????????????????????????????????????????????????????????????????????????????????????????????????????????????????????????????????????????????????????????????????????????????????????????????????????????????????????????????????????????????????????????????????????????????????????????????????????????????????????????????????????????????????????????????????????????????????????????????????????????????????????????????????????????????????????????????????????????????

>*Schizothecium_carpinicola*_CBS_228.87

ATTACAGAGTT-------GCAAAACTCCC--AA-CCA-TTGTGAACC-TACCTCA----CCG-TTGCTTCGGCGTGGCCTCTCTGAGTAA-----CTTATACAATAAGTCAAAACTTTCAACAACGGATCTCTTGGTTCTGGCATCGATGAAGAACGCAGCGAAATGCGATACGTAATGTGAATTGCAGATTTCAGTGAATCATCGAATCTTTGAACGCACATTGCGCCCGCTAGTATTCTGGCGGGCATGCCTGTTCGAGCGTCATTTCAA---CCATCAAG-CCCCC----GGCTTGCGTTGGAG-CCCTGCG-GCT-GCC--GCAGGCTCC-CAAATCCAGTGGCGGGCTCGTC-GTCGT-ACCGAGTGCAGTAAAC--ATCCTCGCTCAGGGAAC-GCGTCGGG-TTCTTGCCGTGAAACCCCCCCC-----ACATCAAGGTTGACCTCGGATCAGGTAGGAATACCCGCTGAACTTAAGCATATCAATAAGCGGAGGAA????????????????ATTGCCC-CAGTAACGGCG-AGTGAAGCGGCAACAGCTCAAATTTGAAATCTGGCCTC--GG-CCCGAGTTGTAATTTGTAGAGG-AAGCTTCTGGTGCGGCGCCGTCCGAGTCTCCTGGAACGGAGCGCCATAGAGGGTGAGAGCCCCGTATGGTCGGATGCCAA-ACCTGTGTGAAGCTCCTTCGACGAGTCGAGTAGTTTGGGAATGCTGCTCTAAATGGGAGGTAAATTCCTTCTAAAGCTAAATATTGGCCAGAGACCGATAGCGCACAAGTAGAGTGATCGAAAGATGAAAAGCACTTTGAAAAGAGGGTTAAACAGCACGTGAAATTGTTGAAAGGGAAGCGCTTGTGACCAGACTTGAGCGCGGCGGATCATCCGGTGTTCTCACCGGTGCACTCCGCCGTGCCCAGGCCAGCATCGGTTTCCGCGGGGGGACAAAGGTCCCGGGAACGTAGCTCC--TCCGGGAGTG-TTATAGCCCGGGGCGCAATGCCCCCGCGGGGACCGAGGACCGCGCATCTGCAAGGATGCTGGCGTAATGGTCATCAGCGACCCGTCTTGAAACACGGACCAAGGAGTCAAGGTTTTGCGCGAGTGTTTGGGTGTTAAACCCGCACGCGTAATGAAAGTGAACGTAGGTGAGAG--CTTCGGCGCATCATCGACCGATCCTGATGTATTCGGACGGATTTGAGTAGGAGCGTTAAGCCTTGGACCCGAAAGATGGTGAACTATGCTTGGATAGGGTGAAGCCAGAGGAAACTCTGGTGGAGGCTCGCAGCGGTT-CTGACGTGCAAATCGATCGTCAAATCTGAGCAT-GGGGGCGAAAGACTAATCGAT???????????????????????????????????????????????????????????????????????????????????????????????????????????????????????????????????????????????????????????????????????????????????????????????????????????????????????????????????????????????????????????????????????????????????????????????????????????????????????????????????????????????????????????????????????????????????????????????????????????????????????????????????????????????????????????????????????????????????????????????????????????????????????????????????????????????????????????????????????????????????????????????????????????????????????????????????????????????????????????????????????????????????????????????????????????????????????????????????????????????????????????????????????????????????????????????????????????????????????????????????????????????????????????????????????????????????????????????????????????????????????????????????????????????????????????????????????????????????????????????????????????????????????????????????????????????????????????????????????????????????????????????????????????????????????????????????????????????????????????????????????????????????????????????????????????????????????????????????????????????????????????????????????????????????????????????????????????????????????????????????????????????????????????????????????????????????????????????????????????????????????????????????????????????????????????????????????????????????????????????????????????????????????????????????????????????????????????????????????????????????????????????????????????????????????????????????????????????????????????????????????????????????

>*Schizothecium_conicum*_CBS_434.50

ATTACAGAGTT-------GCAAAACTCCC--AA-CCA-TTGTGAACC-TACCTCA----CCG-TTGCTTCGGCGTGGCCTCTCTGAGTAA-----CTTATACAATAAGTCAAAACTTTCAACAACGGATCTCTTGGTTCTGGCATCGATGAAGAACGCAGCGAAATGCGATACGTAATGTGAATTGCAGATTTCAGTGAATCATCGAATCTTTGAACGCACATTGCGCCCGCCAGTATTCTGGCGGGCATGCCTGTTCGAGCGTCATTTCAA---CCATCAAG-CCCCC----GGCTTGTGTTGGAG-CCCTGCG-GCT-GCC--GCAGGCTCC-CAAATCCAGTGGCGGGCTCGTC-GTCGT-ACCGAGTGCAGTAAAC--ATCCTCGCTCAGGGCAC-GTGACGGG-TTCCGGCCGTGAAACCCCCACT----TATATCAAGGTTGACCTCGGATCAGGTAGGAATACCCGCTGAACTTAAGCATATCAATAAGCGGAGGAAAAGAAACCAACAGGG-ATTGCCC-CAGTAACGGCG-AGTGAAGCGGCAACAGCTCAAATTTGAAATCTGGCCTC--GG-CCCGAGTTGTAATTTGTAGAGG-AAGCTTCTGGTGCGGCGCCGTCCGAGTCTCCTGGAACGGAGCGCCATAGAGGGTGAGAGCCCCGTATGGACGGATGCCAA-ACCTGTGCGAAGCTCCTTCGACGAGTCGAGTAGTTTGGGAATGCTGCTCTAAATGGGAGGTAAATTCCTTCTAAAGCTAAATATTGGCCAGAGACCGATAGCGCACAAGTAGAGTGATCGAAAGATGAAAAGCACTTTGAAAAGAGGGTTAAACAGCACGTGAAATTGTTGAAAGGGAAGCGCTTGTGACCAGACTTGAGCGCGGCGGATCATCCGGTGTTCTCACCGGTGCACTCTGCCGCGCCCAGGCCAGCATCGGTTTCCGCCGGGGGACAAAGGCACCGGGAACGTAGCTCC--TCCGGGAGTG-TTATAGCCCGGGGCGTAATGCCCTAGCGGGGACCGAGGACCGCGCATCTGCAAGGATGCTGGCGTAATGGTCATCAGCGACCCGTCTTGAAACACGGACCAAGGAGTCAAGGTTTTGCGCGAGTGTTTGGGTGTTAAACCCGCACGCGTAATGAAAGTGAACGTAGGTGAGAG--CTTCGGCGCATCATCGACCGATCCTGATGTATTCGGATGGATTTGAGTAGGAGCGTTAAGCCTTGGACCCGAAAGATGGTGAACTATGCTTGGATAGGGTGAAGCCAGAGGAAACTCTGGTGGAGGCTCGCAGCGGTT-CTGACGTGCAAATCGATCGTCAAATCTGAGCAT?????????????????????????????????????????????????????????????????????????????????????????????????????????????????????????????????????????????????????????????????????????????????????????????????????????????????????????????????????????????????????????????????????????????????????????????????????????????????????????????????????????????????????????????????????????????????????????????????????????????????????????????????????????????????????????????????????????????????????????????????????????????????????????????????????????????????????????????????????????????????????????????????????????????????????????????????????????????????????????????????????????????????????????????????????????????????????????????????????????????????????????????????????????????????????????????????????????????????????????????????????????????????????????????????????????????????????????????????????????????????????????????????????????????????????????????????????????????????????????????????????????????????????????????????????????????????????????????????????????????????????????????????????????????????????????????????????????????????????????????????????????????????????????????????????????????????????????????????????????????????????????????????????????????????????????????????????????????????????????????????????????????????????????????????????????????????????????????????????????????????????????????????????????????????????????????????????????????????????????????????????????????????????????????????????????????????????????????????????????????????????????????????????????????????????????????????????????????????????????????????????????????????????????????????????????

>*Schizothecium_curvisporum*_ATCC_36709

???????????????????????????????????????????????????????????????????????????????????????????????????????????????????????????????????????????????????????????????????????????????????????????????????????????????????????????????????????????????????????????????????????????????????????????????????????????????????????????????????????????????????????????????????????????????????????????????????????????????????????????????????????????????????????????????????????????????????????????????????????????????????????AAAGAAACCAACAGGG-ATTGCCC-CAGTAACGGCG-AGTGAAGCGGCAACAGCTCAAATTTGAAATCTGGCCTC--GG-CCCGAGTTGTAATTTGCAGAGG-AAGCTTCTGGTGCGGCGCCGTCCGAGTCTCCTGGAACGGAGCGCCATAGAGGGTGAGAGCCCCGTATGGACGGACGCCAA-ACCTGTGTGAAGCTCCTTCGACGAGTCGAGTAGTTTGGGAATGCTGCTCAAAATGGGAGGTAAATTCCTTCTAAAGCTAAATACCGGCCAGAGACCGATAGCGCACAAGTAGAGTGATCGAAAGATGAAAAGCACTTTGAAAAGAGGGTTAAACAGCACGTGAAATTGTTGAAAGGGAAGCGCTTGTGACCAGACTCGGGCGCGGCGGATCATCCGGTGTTCTCACCGGTGCACTCCGCCGCGCCCGGGCCAGCATCGGCTTCCGCCGGGGGACAAAGGTCCCGGGAACGTAGCTCC--TCCGGGAGTG-TTATAGCCCGGGGCGCAATGCCCCGGCGGGGGCCGAGGACCGCGCAT-TGCAAGGATGCTGGCGTAATGGTCATCAGCGACCCGTCTTGAAACACGGACCAAGGAGTCAAGGTTTTGCGCGAGTGTTTGGGTGTTAAACCCGCACGCGTAATGAAAGTGAACGTAGGTGAGAG--CTTCGGCGCATCATCGACCGATCCTGATGTATTCGGATGGATTTGAGTAGGAGCGTTAAGCCTTGGACCCGAAAGATGGTGAACTATGCTTGGATAGGGTGAAGCCAGAGGAAACTCTGGTGGAGGCTCGCAGCGGTT-CTGACGTGCAAATCGATCGTCAAATCTGAGCAT-GGGGGCGAAAGACTAATCGAACCATCTAGTAGCTGGTTACCGCCGCGCTCAAGTACTCGCTTGCCACGGGTAACTGGGGCGACCAGAAGAAGGCGGCGAGCTCGACAGCCGGTGTGTCCCAGGTGTTGAATCGCTACACGTTTGCCTCGACCCTTTCTCATTTGCGCCGCACCAACACGCCCATCGGCCGCGACGGAAAGCTGGCCAAGCCGCGCCAGCTGCACAACACCCATTGGGGCCTCGTCTGTCCAGCCGAGACGCCCGAGGGCCAGGCCTGCGGGCTGGTCAAGAATCTGTCGCTCATGTGCTACATTAGCGTGGGCACCAACGCGGAGCCCATTATCGAGTTCATGATCGCGCGCAACATGGAGGTCTTGGAAGAGTACGAGCCACTGCGCTCCCCCAACGCCACCAAGATCTTTGTCAACGGCACGTGGGTCGGCGTGCACCACGACGCCAAGCACCTCGTGCACCTTGTCCAGGGTCTCCGGCGATCCAACATTGTGAGCTTCGAGGTGTCGCTGGTCCGGGATATCCGAGACCGCGAGTTCAAGATCATGTCGGATGCCGGCCGCGTCATGAGGCCCCTCTTCGTCGTCGAGACCGAGGACGAGAGCTCC---------ACCGGGGTGGAAAAGGGCGAGCTGGTGCTCACCAAGACCCACGTCCAGAAGCTGGCCAACGACAAGCTGATTGGGAAATACC------------ACAAAGACTACTTTGGGTGGCAAGGCCTCTTGCAATCAGGTGCCGTCGAATACCTCGACGCCGAGGAGGAGGAGACGGCCATGATCTCCATGTCGCCCGAAGACCTCGACCATTTCCGCGACGCCAAGGC------GAGAAACTTTGAGGAACCCGAGGGCAAGGTGGTGACCGAGGGCAACAAGCGCATCCCGACGAGGATCAACCCGACGACCTACATGTATACGCACTGCGAGATCCACCCGAGCATGCTGCTCGGCATCTGCGCGAGCATCATCCCTTTCCCGGACCACAACCAGGCTTGTCGACCAGGTTCTCGATGTCGTCCGTCGCGAGGCCGAGGGCTGCGACTGCCTCCAGGGCTTCCAGATCACCCACTCGCTCGGTGGTGGTACCGGTGCCGGTATGGGTACCCTCCTCATCTCCAAGATCCGCGAGGAGTTCCCCGACCGCATGATGGCGACCTTCTCCGTCGTCCCCTCCCCCAAGGTGTCGGATACCGTCGTGGAGCCCTACAACGCCACCCTCTCCGTCCACCAGCTTGTCGAGAACTCGGACGAGACCTTCTGCATTGACAACGAGGCTCTGTACGACATCTGCATGCGCACCCTCAAGCTGTCGAACCCCTCGTACGGCGACCTCAACCACCTCGTCTCGGCCGTCATGTCGGGTGTCACCGTTTCGCTGCGCTTCCCCGGCCAGCTCAACTCTGATCTCCGCAAGCTCGCCGTGAACATGGTTCCCTTCCCCCGTCTCCACTTCTTCATGGTCGGCTTCGCCCCTCTTACCAGCCGTGGCGCCCACTCTTTCCGTGCCGTCTCGGTCCCCGAGCTCACCCAGCAGATGTTCGACCCCAAGAACATGATGGCTGCCTCGGACTTCCGCAACGGTCGTTACCTTACCTGCTCCGCCATCTT

>*Schizothecium_fimbriatum*_CBS_144.54

ATTACAGAGTT-------GCAAAACTCCC--AA-CCA-TTGTGAACC-TACCTCA----CCG-TTGCTTCGGCGTGGCCTCTCTGAGTAA-----CTTATACAATAAGTCAAAACTTTCAACAACGGATCTCTTGGTTCTGGCATCGATGAAGAACGCAGCGAAATGCGATACGTAATGTGAATTGCAGATTTCAGTGAATCATCGAATCTTTGAACGCACATTGCGCCCGCCAGTATTCTGGCGGGCATGCCTGTTCGAGCGTCATTTCAA---CCATCAAG-CCCCC----GGCTTGTGTTGGGG-CCCTGCG-GCT-GCC--GCAGACCCC-TAAATCCAGTGGCGGGCTCGTC-GTCGT-ACCGAGTGCAGTAAAC--ATCCTCGCTCAGGGAAC-CCGACGGG-TGCCGGCCGTGAAACCCCCCCT------TCTCAAGGTTGACCTCGGATCAGGTAGGAATACCCGCTGAACTTAAGCATATCAATAAGCGGAGGA???????CCAACAGGGCATTGCCC-CAGTAACGGCG-AGTGAAGCGGCAACAGCTCAAATTTGAAATCTGGCCTC--GG-CCCGAGTTGTAATTTGCAGAGG-AAGCTTCTGGTGCGGCGCCGTCCGAGTCTCCTGGAACGGAGCGCCATAGAGGGTGAGAGCCCCGTATGGACGGACGCCAA-ACCTGTGTGAAGCTCCTTCGACGAGTCGAGTAGTTTGGGAATGCTGCTCAAAATGGGAGGTAAATTCCTTCTAAAGCTAAATATTGGCCAGAGACCGATAGCGCACAAGTAGAGTGATCGAAAGATGAAAAGCACTTTGAAAAGAGGGTTAAACAGCACGTGAAATTGTTGAAAGGGAAGCGCTTGTGACCAGACTTGGGCGCGGCGGATCATCCGGTGTTCTCACCGGTGCACTCCGCCGCGCCCGGGCCAGCATCGGCTTCCGCCGGGGGACAAAGGTCCCGGGAACGTAGCTCC--TCCGGGAGTG-TTATAGCCCGGGGCGCAATGCCCCGGCGGGGGCCGAGGACCGCGCAT-TGCAAGGATGCTGGCGTAATGGTCATCAGCGACCCGTCTTGAAACACGGACCAAGGAGTCAAGGTTTTGCGCGAGTGTTTGGGTGTTAAACCCGCACGCGTAATGAAAGTGAACGTAGGTGAGAG--CTTCGGCGCATCATCGACCGATCCTGATGTATTCGGATGGATTTGAGTAGGAGCGTTAAGCCTTGGACCCGAAAGATGGTGAACTATGCTTGGATAGGGTGAAGCCAGAGGAAACTCTGGTGGAGGCTCGCAGCGGTT-CTGACGTGCAAATCGATCGTCAAATCTGAGCAT-GGGGGCGAAAGACTAATCGA????????????????????????GCGCTCAAGTACTCGCTTGCCACGGGCAACTGGGGCGACCAGAAGAAGGCGGCAAGCTCGACGGCCGGCGTGTCGCAGGTGCTGAATCGCTACACGTTCGCCTCGACCCTCTCTCATTTGCGCCGCACCAACACACCCATCGGTCGCGATGGAAAGCTCGCCAAGCCGCGTCAGCTTCACAACACGCATTGGGGCCTCGTCTGTCCGGCCGAGACGCCCGAGGGCCAGGCCTGCGGGCTGGTCAAGAATCTGTCGCTCATGTGCTACATCAGCGTGGGCACCAACGCGGAGCCCATTGTCGAGTTCATGATTGCGCGCAACATGGAGGTGTTGGAAGAGTACGAGCCGCTGCGCTCCCCCAACGCCACCAAGATCTTTGTCAACGGCACATGGGTCGGCGTGCACCACGACGCCAAGCACCTCGTGCACCTTGTCCAGGGCCTCCGGCGATCCAACATTGTGAGCTTCGAGGTGTCGCTGGTCCGGGATATCCGAGACCGCGAGTTCAAGATCATGTCGGATGCCGGCCGCGTCATGAGACCGCTCTTTGTTGTCGAGACCGAGGACGAGAGCTCT---------ACGGGAGTGGAAAAGGGCGAGCTGGTGCTCACCAAGACCCACGTCCAGAAGCTGGCCAACGACAAGCTGATTGGGAAATACC------------ACAAGGACTACTTTGGGTGGCAAGGCCTCTTGCAATCGGGCGCCGTCGAATACCTCGACGCCGAGGAGGAGGAGACGTCCATGATCTCCATGTCGCCCGAGGACCTGGACCATTTCCGCGACGCCAAGGC------GAGAAACTTTGAGGAGCCCGAGGGCAAGGTGGTGACCGAGGGCAACAAGCGCATCCCGACGAGGATCAACCCGACGACCTACATGTACACGCACTGCGAGATCCACCCCAGCATGCTGCTCGGTATCTGCGCGAGCATCATCCCTTTCCCCGACCACAACCAGGCTTGTCGACCAGGTTCTCGATGTCGTCCGTCGCGAGGCCGAGGGCTGCGACTGCCTCCAGGGCTTCCAGATCACCCACTCGCTCGGTGGTGGTACCGGTGCCGGCATGGGTACTCTCCTCATCTCCAAGATCCGCGAGGAGTTCCCCGACCGCATGATGGCGACCTTCTCCGTCGTCCCCTCGCCCAAGGTGTCGGATACCGTCGTGGAACCCTACAACGCCACCCTCTCCGTCCACCAGCTTGTTGAGAACTCGGACGAGACCTTCTGCATTGACAACGAGGCTCTCTACGACATCTGCATGCGCACCCTCAAGCTGTCGAACCCCTCGTACGGCGACCTCAACCACCTCGTCTCGGCCGTCATGTCGGGTGTCACCGTTTCGCTGCGCTTCCCCGGCCAGCTCAACTCCGATCTCCGCAAGCTCGCCGTGAACATGGTTCCCTTCCCCCGTCTCCACTTCTTCATGGTCGGCTTCGCCCCTCTTACCAGCCGTGGCGCCCACTCTTTCCGCGCCGTTTCGGTTCCCGAGCTCACCCAGCAGATGTTCGACCCCAAGAACATGATGGCTGCCTCGGACTTCCGCAACGGTCGTTACCTTACCTGCTCTGCCATCTT

>*Schizothecium_glutinans*_CBS_134.83

ATTACAGAGTT-------GCAAAACTCCC--AA-CCA-TTGTGAACC-TACCTCA----CCG-TTGCTTCGGCGTGGCCTCTCTGAGTAA-----CTTATACAATAAGTCAAAACTTTCAACAACGGATCTCTTGGTTCTGGCATCGATGAAGAACGCAGCGAAATGCGATACGTAATGTGAATTGCAGATTTCAGTGAATCATCGAATCTTTGAACGCACATTGCGCCCGCCAGTATTCTGGCGGGCATGCCTGTTCGAGCGTCATTTCAA---CCATCAAG-CGCCT---GCGCTTGTGTTGTAG-CCCTGCG-GCT-GCC--GCAGGCTCC-CAAACCCAGTGGCGGGCTCGTC-GTCGT-ACCGAGTGCAGTAAAT-TTACCACGCTCAGGGCGC-GCGACGGGTGACCGGCCGTAAAACCCCCCAA---CTTTATCAAGGTTGACCTCGGATCAGGTAGGAATACCCGCTGAACTTAAGCATATCAATAAGCGGAGGA????????CAACAGGG-ATTGCCC-CAGTAACGGCG-AGTGAAGCGGCAACAGCTCAAATTTGAAATCTGGCCTC--GG-CCCGAGTTGTAATTTGCAGAGG-AAGCTTCTGGTGCGGCGCTGTCCGAGTCTCCTGGAACGGAGCGCCATAGAGGGTGAGAGCCCCGTATGGACGGATGCCAA-ACCTGTGTGAAGCTCCTTCGACGAGTCGAGTAGTTTGGGAATGCTGCTCTAAATGGGAGGTAAATTCCTTCTAAAGCTAAATATTGGCCAGAGACCGATAGCGCACAAGTAGAGTGATCGAAAGATGAAAAGCACTTTGAAAAGAGGGTTAAACAGCACGTGAAATTGTTGAAAGGGAAGCGCTTGTGACCAGACTTGAGCGCGGCGGATCATCCAGTGTTCTCACCGGTGCACTCCGCCGCGTCCAGGCCAGCATCGGTTTCCACGGGGGGACAAAGGTCCCGGGAACGTAGCTCT--TTCGAGAGTG-TTATAGCCCGGGGCGCAATGCCCCCGCGGGGACCGAGGACCGCGCATCTGCAAGGATGCTGGCGTAATGGTCATCAGCGACCCGTCTTGAAACACGGACCAAGGAGTCAAGGTTTTGCGCGAGTGTTTGGGTGTTAAACCCGCACGCGTAATGAAAGTGAACGTAGGTGAGAG--CTTCGGCGCATCATCGACCGATCCTGATGTATTCGGATGGATTTGAGTAGGAGCGTTAAGCCTTGGACCCGAAAGATGGTGAACTATGCTTGGATAGGGTGAAGCCAGAGGAAACTCTGGTGGAGGCTCGCAGCGGTT-CTGACGTGCAAATCGATCGTCAAATCTGAGCAT-GGGGGCGAAAGACTAATCGA????????????????????????????????????????????????????????????????????????????????????????????????????????????????????????????????????????????????????????????????????????????????????????????????????????????????????????????????????????????????????????????????????????????????????????????????????????????????????????????????????????????????????????????????????????????????????????????????????????????????????????????????????????????????????????????????????????????????????????????????????????????????????????????????????????????????????????????????????????????????????????????????????????????????????????????????????????????????????????????????????????????????????????????????????????????????????????????????????????????????????????????????????????????????????????????????????????????????????????????????????????????????????????????????????????????????????????????????????????????????????????????????????????????????????????????????????????????????????????????????????????????????????????????????????????????????????????????????????????????????????????????????????????????????????????????????????????????????????????????????????????????????????????????????????????????????????????????????????????????????????????????????????????????????????????????????????????????????????????????????????????????????????????????????????????????????????????????????????????????????????????????????????????????????????????????????????????????????????????????????????????????????????????????????????????????????????????????????????????????????????????????????????????????????????????????????????????????????????????????????????????????????????

>*Schizothecium_inaequale*_CBS_356.49

ATTACAGAGTT-------GCAAAACTCCC--AA-CCA-TTGTGAACC-TACCTCA----CCG-TTGCCTCGGCGTGGCCTCTCTGAGTAA-----CTTATACAATAAGTCAAAACTTTCAACAACGGATCTCTTGGTTCTGGCATCGATGAAGAACGCAGCGAAATGCGATACGTAATGTGAATTGCAGATTTCAGTGAATCATCGAATCTTTGAACGCACATTGCGCCCGCCAGTATTCTGGCGGGCATGCCTGTTCGAGCGTCATTTCAA---CCATCAAG-CGCCC----CGCTTGTGTTGGGG-CCCTGCG-GCT-GCC--GCAGACCCC-TAAATCCAGTGGCGGGCTCGTC-GTCGT-ACCGAGTGCAGTAAAC--ATCCTCGCTCAGGGCAC-GCGCCGGGTCTCTTGCCGTGAAACACCCCAC-----ATATCAAGGTTGACCTCGGATCAGGTAGGAATACCCGCTGAACTTAAGCATATCAATAAGCGGAGGAAAAGAAACCAACAGGG-ATTGCCC-CAGTAACGGCG-AGTGAAGCGGCAACAGCTCAAATTTGAAATCTGGCCTC--GG-CCCGAGTTGTAATTTGCAGAGG-AAGCTTCTGGTGCGGCGCCGTCCGAGTCTCCTGGAACGGAGCGCCATAGAGGGTGAGAGCCCCGTATGGACGGATGCCAA-ACCTGTGTGAAGCTCCTTCGACGAGTCGAGTAGTTTGGGAATGCTGCTCTAAATGGGAGGTAAATTCCTTCTAAAGCTAAATATTGGCCAGAGACCGATAGCGCACAAGTAGAGTGATCGAAAGATGAAAAGCACTTTGAAAAGAGGGTTAAACAGCACGTGAAATTGTTGAAAGGGAAGCGCTTGTGACCAGACTTGAGCGCGGCGGATCATCCGGTGTTCTCACCGGTGCACTCCGCCGCGCCCAGGCCAGCATCGGCTTCCGCGGGGGGACAAAGGTCCCGGGAACGTAGCTCC--TCCGGGAGTG-TTATAGCCCGGGGCGCAATGCCCCCGCGGGGGCCGAGGACCGCGCATCTGCAAGGATG??????????????????????????????????????????????????????????????????????????????????????????????????????????????????????????????????????????????????????????????????????????????????????????????????????????????????????????????????????????????????????????????????????????????????????????????????????????????????????????????????????????????????????????????????????????????????????????????????????????????????????????????????????????????TTTGCTTCGACCCTCTCTCATTTGCGCCGCACCAACACCCCCATCGGCCGCGACGGAAAGCTGGCCAAGCCGCGCCAGCTGCACAACACCCATTGGGGCCTCGTCTGTCCGGCCGAGACGCCCGAGGGCCAGGCCTGCGGGCTGGTCAAGAATTTGTCCCTCATGTGCTACATCAGCGTGGGCACGAACGCGGAGCCCATTGTCGAGTTCATGATTGCGCGCAACATGGAGGTCTTGGAAGAGTACGAGCCACTGCGCTCCCCCAACGCCACCAAGATCTTTGTAAACGGTACGTGGGTCGGCGTGCACCACGACGCCAAGCATCTCGTGCACCTTGTCCAGGGCCTCCGGCGATCCAACATTGTGAGCTTCGAAGTGTCGCTGGTCCGGGATATCCGAGACCGCGAGTTCAAGATCATGTCGGATGCCGGCCGCGTCATGAGGCCCCTCTTCGTTGTCGAGACCGAGGACGAGAGCTCC---------ACGGGAGTAGAAAAGGGCGAGCTGGTGCTCACCAAGACCCACGTCCAGAAGCTGGCCAACGACAAGCTGATTGGGAAATACC------------ACAAGGACTACTTCGGGTGGCAAGGCCTCTTGCAATCGGGCGCCGTCGAATACCTCGACGCCGAGGAGGAGGAGACGTCCATGATCTCCATGTCGCCCGAGGACCTCGACCATTTCCGCGACGCCAAGGC------GAGAAACTTTGAGGAACCCGAGGGCAAGGTGGTAACCGAGGGCAACAAGCGCATCCCGACGAGGATCAACCCGACGACCTACATGTATACGCACTGCGAGATCCACCCCAGCATGCTGCTCGGCATCTGCGCGAGCATCATCCCTTTCCCCGACCACAACCAG??????????????????????????????????????????????????????????????????????????????????????????????????????????????????????????????????????????????????????????????????????????????????????????????????????????????????????????????????????????????????????????????????????????????????????????????????????????????????????????????????????????????????????????????????????????????????????????????????????????????????????????????????????????????????????????????????????????????????????????????????????????????????????????????????????????????????????????????????????????????????????????????????????????????????????????????????????????

>*Schizothecium_minicauda*_CBS_227.87

ATTACAGAGTT-------GCAAAACTCCC--AA-CCA-TTGTGAACC-TACCTCA----CCG-TTGCTTCGGCGTGGCCTCTCTGAGTAA-----CTTATACAATAAGTCAAAACTTTCAACAACGGATCTCTTGGTTCTGGCATCGATGAAGAACGCAGCGAAATGCGATACGTAATGTGAATTGCAGATTTCAGTGAATCATCGAATCTTTGAACGCACATTGCGCCCGCCAGTATTCTGGCGGGCATGCCTGTTCGAGCGTCATTTCAA---CCATCAAG-CGCCC----CGCTTGTGTTGGGG-CCCTGCG-GCT-GCC--GCAGACCCC-TAAATCCAGTGGCGGGCTCGTC-GTCGT-ACCGAGTGCAGTAAAC--ATCCTCGCTCAGGGCAC-GCGGCGGGTTCCTTGCCGTGAAACACCCCAC-----ATATCAAGGTTGACCTCGGATCAGGTAGGAATACCCGCTGAACTTAAGCATATCAATAAGCGGAGGAAAAGAAACCAACAGGG-ATTGCCC-CAGTAACGGCG-AGTGAAGCGGCAACAGCTCAAATTTGAAATCTGGCCTC--GG-CCCGAGTTGTAATTTGCAGAGG-AAGCTTCTGGTGCGGCGCCGTCCGAGTCTCCTGGAACGGAGCGCCATAGAGGGTGAGAGCCCCGTATGGACGGATGCCAA-ACCTGTGTGAAGCTCCTTCGACGAGTCGAGTAGTTTGGGAATGCTGCTCTAAATGGGAGGTAAATTCCTTCTAAAGCTAAATATTGGCCAGAGACCGATAGCGCACAAGTAGAGTGATCGAAAGATGAAAAGCACTTTGAAAAGAGGGTTAAACAGCACGTGAAATTGTTGAAAGGGAAGCGCTTGTGACCAGACTTGAGCGCGGCGGATCATCCGGTGTTCTCACCGGTGCACTCCGCCGCGCCCAGGCCAGCATCGGTTTCCGCGGGGGGACAAAGGTCCCGGGAACGTAGCTCC--TCCGGGAGTG-TTATAGCCCGGGGCGCAATGCCCCCGCGGGGACCGAGGACCGCGCATCTGCAAGGATGCTGGCGTAATGGTCATCAGCGACCCGTCTTGAAACACGGACCAAGGAGTCAAGGTTTTGCGCGAGTGTTTGGGTGTTAAACCCGCACGCGTAATGAAAGTGAACGTAGGTGAGAG--CTTCGGCGCATCATCGACCGATCCTGATGTATTCGGATGGATTTGAGTAGGAGCGTTAAGCCTTGGACCCGAAAGATGGTGAACTATGCTTGGATAGGGTGAAGCCAGAGGAAACTCTGGTGGAGGCTCGCAGCGGTT-CTGACGTGCAAATCGATCGTCAAATCTGAGCAT-GGGGGCGAAAGACTAATCGAACCATCTAGTAGCTGGTTACCGCC????????????????????????????????????????????????????????????????????????????????????????????????????????????????????????????????????????????????????????????????????????????????????????????????????????????????????????????????????????????????????????????????????????????????????????????????????????????????????????????????????????????????????????????????????????????????????????????????????????????????????????????????????????????????????????????????????????????????????????????????????????????????????????????????????????????????????????????????????????????????????????????????????????????????????????????????????????????????????????????????????????????????????????????????????????????????????????????????????????????????????????????????????????????????????????????????????????????????????????????????????????????????????????????????????????????????????????????????????????????????????????????????????????????????????????????????????????????????????????????????????????????????????????????????????????????????????????????????????????????????????????????????????????????????????????????????????????????????????????????????????????????????????????????????????????????????????????????????????????????????????????????????????????????????????????????????????????????????????????????????????????????????????????????????????????????????????????????????????????????????????????????????????????????????????????????????????????????????????????????????????????????????????????????????????????????????????????????????????????????????????????????????????????????????????????????????????????????????????????????

>*Schizothecium_selenosporum*_CBS_109403

ATTACAGAGTT-------GCAAAACTCCC--AA-CCA-TTGTGAACC-TACCTCA----CCG-TTGCTTCGGCGTGGCCTCTCTGAGTAA-----CTTATACAATAAGTCAAAACTTTCAACAACGGATCTCTTGGTTCTGGCATCGATGAAGAACGCAGCGAAATGCGATACGTAATGTGAATTGCAGATTTCAGTGAATCATCGAATCTTTGAACGCACATTGCGCCCGCCAGTATTCTGGCGGGCATGCCTGTTCGAGCGTCATTTCAA---CCATCAAG-CCCCC----GGCTTGTGTTGGGG-CCCTGCG-GCT-GCC--GCAGGCCCC-TAAATCCAGTGGCGGGCTCGTC-GTCGT-ACCGAGCGCAGTAAAC--ATCCTCGCTCGGGGACC-CCGACGGG-TGCCGGCCGTGAAACCCCCCCT------TCTCAAGGTTGACCTCGGATCAGGTAGGAATACCCGCTGAACTTAAGCATATCAATAAGCGGAGGAAAAGAAACCAACAGGG-ATTGCCC-CAGTAACGGCG-AGTGAAGCGGCAACAGCTCAAATTTGAAATCTGGCCTC--GG-CCCGAGTTGTAATTTGCAGAGG-AAGCTTCTGGTGCGGCGCCGTCCGAGTCTCCTGGAACGGAGCGCCATAGAGGGTGAGAGCCCCGTATGGACGGACGCCAA-ACCTGTGTGAAGCTCCTTCGACGAGTCGAGTAGTTTGGGAATGCTGCTCAAAATGGGAGGTAAATTCCTTCTAAAGCTAAATACCGGCCAGAGACCGATAGCGCACAAGTAGAGTGATCGAAAGATGAAAAGCACTTTGAAAAGAGGGTTAAACAGCACGTGAAATTGTTGAAAGGGAAGCGCTTGTG--CAGACTCGGGCGCGGCGGATCATCCGGTGTTCTCACCGGTGCACTCCGCCGCGCCCGGGCCAGCATCGGCTTCCGCCGGGGGATAAAGGTCCCGGGAACGTAGCTCC--TCCGGGAGTG-TTATAGCCCGGGGCGCAATGCCCCGGCGGGGGCCGAGGACCGCGCAT-TGCAAGGATG????????????????????????????????????????????????????????????????????????????????????????????????????????????????????????????????????????????????????????????????????????????????????????????????????????????????????????????????????????????????????????????????????????????????????????????????????????????????????????????????????????????????????????????????????????????????????????????????????????????????????????????????????????????????????TCGACCCTCTCTCATTTGCGCCGCACCAACACGCCCATCGGCCGCGACGGAAAGCTCGCCAAGCCGCGCCAGCTTCACAACACGCATTGGGGCCTCGTCTGTCCAGCCGAGACGCCCGAGGGCCAGGCCTGCGGGCTGGTCAAGAATCTGTCGCTCATGTGCTACATCAGCGTGGGCACCAACGCGGAGCCCATTATCGAGTTCATGATCGCACGCAACATGGAGGTCTTGGAAGAGTACGAGCCACTGCGCTCCCCCAATGCCACCAAGATCTTTGTCAACGGCACGTGGGTCGGCGTGCACCACGACGCCAAGCACCTCGTGCACCTTGTCCAGGGCCTCCGGCGATCCAACATTGTGAGCTTCGAGGTGTCGCTGGTCCGGGATATCCGAGACCGCGAGTTCAAGATCATGTCGGATGCCGGCCGCGTCATGAGGCCCCTCTTCGTCGTCGAGACCGAGGACGAGAGCTCC---------ACGGGGGTGGAAAAGGGCGAGCTGGTGCTCACCAAGACCCACGTCCAGAAGCTGGCCAACGACAAGCTGATTGGGAAATACC------------ACAAGGACTACTTTGGGTGGCAAGGCCTCTTGCAATCAGGCGCCGTCGAATACCTCGACGCCGAGGAGGAGGAGACGGCCATGATCTCCATGTCGCCCGAGGACCTCGACCATTTCCGCGACGCCAAGGC------GAGAAACTTTGAGGAACCCGAGGGCAAGGTGGTGACCGAGGGCAACAAGCGCATCCCGACGAGGATCAACCCGACGACCTACATGTATACGCACTGCGAGATCCACCCCAGCATGCTGCTCGGCATCTGTGCGAGCATCATCCCTTTCCCGGACCACAACCAG??????????????????????????????????????????????????????????????????????????????????????????????????????????????????????????????????????????????????????????????????????????????????????????????????????????????????????????????????????????????????????????????????????????????????????????????????????????????????????????????????????????????????????????????????????????????????????????????????????????????????????????????????????????????????????????????????????????????????????????????????????????????????????????????????????????????????????????????????????????????????????????????????????????????????????????????????????????

>*Schizothecium_tetrasporum*_CBS_394.87

ATTACAGAGTT-------GCAAAACTCCC--AA-CCA-TTGTGAACC-TACCTCA----CCG-TTGCCTCGGCGTGGCCTCTCTGAGTAA-----CTTATACAATAAGTCAAAACTTTCAACAACGGATCTCTTGGTTCTGGCATCGATGAAGAACGCAGCGAAATGCGATACGTAATGTGAATTGCAGATTTCAGTGAATCATCGAATCTTTGAACGCACATTGCGCCCGCCAGTATTCTGGCGGGCATGCCTGTTCGAGCGTCATTTCAA---CCATCAAG-CGCAC----CGCTTGTGTTGGGG-CCCTGCG-GCT-GCCG-CAGACCCCT--AAATCCAGTGGCGGGCTCGTC-GTCGT-ACCGAGTGCAGTAAAC--ATCCTCGCTCAGGGCAC-GCGCCGGGTCTCTTGCCGTGAAACACCCCAC-----ATATCAAGGTTGACCTCGGATCAGGTAGGAATACCCGCTGAACTTAAGCATATCAATAAGCGGAGGAAAAGAAACCAACAGGG-ATTGCCC-CAGTAACGGCG-AGTGAAGCGGCAACAGCTCAAATTTGAAATCTGGCCTC--GG-CCCGAGTTGTAATTTGCAGAGG-AAGCTTCTGGTGCGGCGCCGTCCGAGTCTCCTGGAACGGAGCGCCATAGAGGGTGAGAGCCCCGTATGGACGGATGCCAA-ACCTGTGTGAAGCTCCTTCGACGAGTCGAGTAGTTTGGGAATGCTGCTCTAAATGGGAGGTAAATTCCTTCTAAAGCTAAATATTGGCCAGAGACCGATAGCGCACAAGTAGAGTGATCGAAAGATGAAAAGCACTTTGAAAAGAGGGTTAAACAGCACGTGAAATTGTTGAAAGGGAAGCGCTTGTGACCAGACTTGAGCGCGGCGGATCATCCGGTGTTCTCACCGGTGCACTCCGCCGTGCCCAGGCCAGCATCGGCTTCCGCGGGGGGACAAAGGTCCCGGGAACGTAGCTCC--TCCGGGAGTG-TTATAGCCCGGGGCGCAATGCCCCCGCGGGGGCCGAGGACCGCGCATCTGCAAGGATGCTGGCGTAATGGTCATCAGCGACCCGTCTTGAAACACGGACCAAGGAGTCAAGGTTTTGCGCGAGTGTTTGGGTGTTAAACCCGCACGCGTAATGAAAGTGAACGTAGGTGAGAG--CTTCGGCGCATCATCGACCGATCCTGATGTATTCGGATGGATTTGAGTAGGAGCGTTAAGCCTTGGACCCGAAAGATGGTGAACTATGCTTGGATAGGGTGAAGCCAGAGGAAACTCTGGTGGAGGCTCGCAGCGGTT-CTGACGTGCAAATCGATCGTCAAATCTGAGCAT-GGGGGCGAAAGACTAATCGAACCATCTAGTAGCTGGTTACCGCC????????????????????????????????????????????????????????????????????????????????????????????????????????????????????????????????????????????????????????????????????????????????????????????????????????????????????????????????????????????????????????????????????????????????????????????????????????????????????????????????????????????????????????????????????????????????????????????????????????????????????????????????????????????????????????????????????????????????????????????????????????????????????????????????????????????????????????????????????????????????????????????????????????????????????????????????????????????????????????????????????????????????????????????????????????????????????????????????????????????????????????????????????????????????????????????????????????????????????????????????????????????????????????????????????????????????????????????????????????????????????????????????????????????????????????????????????????????????????????????????????????????????????????????????????????????????????????????????????????????????????????????????????????????????????????????????????????????????????????????????????????????????????????????????????????????????????????????????????????????????????????????????????????????????????????????????????????????????????????????????????????????????????????????????????????????????????????????????????????????????????????????????????????????????????????????????????????????????????????????????????????????????????????????????????????????????????????????????????????????????????????????????????????????????????????????????????????????????????????????

>*Zopfiella_erostrata*_CBS_255.71

ATTACAGAGTT-------GCAAAACTCCC--AACCCT-TTGTGAATC-AACTAGA----CAG-TTGCCTCGGCGTGGCCTCTCTGAGTAA------TTCTAAAATGAATCAAAACTTTCAACAACGGATCTCTTGGTTCTGGCATCGATGAAGAACGCAGCGAAATGCGATACGTAATGTGAATTGCAGAATTCAGTGAATCATCGAATCTTTGAACGCACATTGCGCCCGCCAGCACTCTGGCGGGCATGCCTGTTCGAGCGTCATTTCAA---CCATCAAG-CCCCA----GGCTTGTGTTGGAG-CCCTGCG-GCT-GCC--GCAGCCTCC-TAAAAGCAGTGGCGGGCTCGCT-ATCAC-ACCGAGTGCAGTAGATTTCTCTTCGCTCAGGACGT-GTGGCGGG-TTCCGGCCGTGAAACCCCCTAC-----TCTTTAAGGTTGACCTCGGATCAGGTAGGAATACCCGCTGAACTTAAGCATATCAATAAGCGGAGGAA???????CAACAGGG-ATTGCCC-TAGTAACGGCG-AGTGAAGCGGCAACAGCTCAAATTTGAAATCTGGCTCC--GG-CCCGAGTTGTAATTTGCAGAGG-AAGCTTCTGGTGATGCACTGTCTAAGTCCCCTGGAACGGGGCGCCACAGTGGGTGAGAGCCCCATTTG-ACAGAGGCAGA-TCCTGTGTGAAGCTCCTTCGACGAGTCGAGTAGTTTGGGAATGCTGCTCAAAATGGGAGGTAAATTCCTTCTAAAGCTAAATATTGGCCAGAGACCGATAGCGCACAAGTAGAGTGATCGAAAGATGAAAAGCACTTTGAAAAGAGGGTTAAACAGCACGTGAAATTGTTGAAAGGGAAGCGCTTGTGACCAGACTTGCGCTGGGCTGATCATCCGGTGTTCTCACCGGTGCACTCGGCCCAGCTCAGGCCAGCATCGGTTTTGGTGGGGGGATAAAGGCGCTGGGAACGTAGCTCC--TTCGGGAGTG-TTATAGCCCAGCGTGCAATGCCCCCGCTGGGACCGAGGTTCGCGCATCTGCAAGGATGCTGGCGTAATGGTCATCAGCGACCCGTCTTGAAACACGGACCAAGGAGTCAAGGTTTTGCGCGAGTGTTTGGGTGTCAAACCCGCACGCGTAATGAAAGTGAACGTAGGTGAGAG--CTTCGGCGCATCATCGACCGATCCTGATGTATTCGGATGGATTTGAGTAGGAGCGTTAAGCCTTGGACCCGAAAGATGGTGAACTATGCTTGGATAGGGTGAAGCCAGAGGAAACTCTGGTGGAGGCTCGCAGCGGTT-CTGACGTGCAAATCGATCGTCAAATCTGAGCAT-GGGGGCGAAAGACTAATCGA????????????????????????????????????????????????????????????????????????????????????????????????????????????????????????????????????????????????????????????????????????????????????????????????????????????????????????????????????????????????????????????????????????????????????????????????????????????????????????????????????????????????????????????????????????????????????????????????????????????????????????????????????????????????????????????????????????????????????????????????????????????????????????????????????????????????????????????????????????????????????????????????????????????????????????????????????????????????????????????????????????????????????????????????????????????????????????????????????????????????????????????????????????????????????????????????????????????????????????????????????????????????????????????????????????????????????????????????????????????????????????????????????????????????????????????????????????????????????????????????????????????????????????????????????????????????????????????????????????????????????????????????????????????????????????????????????????????????????????????????????????????????????????????????????????????????????????????????????????????????????????????????????????????????????????????????????????????????????????????????????????????????????????????????????????????????????????????????????????????????????????????????????????????????????????????????????????????????????????????????????????????????????????????????????????????????????????????????????????????????????????????????????????????????????????????????????????????????????????????????????????????????????

>*Zopfiella_tardifaciens*_CBS_670.82

ATTACAGAGTT-------GCAAAACTCCC--AACCCT-TTGTGAACG-CACCTAG----CAG-TTGCTTCGGCGTGGCATCTCTGAGTAG-----CTTATAAAATAAGTTAAAACTTTCAACAACGGATCTCTTGGTTCTGGCATCGATGAAGAACGCAGCGAAATGCGATAAGTAATGTGAATTGCAGAATTCAGTGAATCATCGAATCTTTGAACGCACATTGCGCCCGCCAGTATTCTGGCGGGCATGCCTGTTCGAGCGTCATTTCAA---CCATCAAG-CCCCA----GGCTTGTGTTGGAG-CCCTGCG-GCC-GCC-CGCAGCCTCC-CAAAATCAGTGGCGGGCTTGCA-AACAC-CCCGAGCGCAGTAGTTTACTCTTCTCTCTGGCGT--GTAGCAGG-TTCCGGCCGTGAAACCCCCCCA----TATTCTAAGGTTGACCTCGGATCAGGTAGGAATACCCGCTGAACTTAAGCATATCAATAAGCGGAGGAAAAGAAACCAACAGGG-ATTGCCC-TAGTAACGGCG-AGTGAAGCGGCAACAGCTCAAATTTGAAATCTGGCTCC--GG-CCCGAGTTGTAATTTGCAGAGG-AAGCTTCTGGTGATATACTGTCTAAGTCCCCTGGAACGGGGCGCCACAGTGGGTGAGAGCCCCATATG-ACAGCTGTAGA-TCCTGTGTGAAGCTCCTTCGACGAGTCGAGTAGTTTGGGAATGCTGCTCAAAATGGGAGGTAAATTCCTTCTAAAGCTAAATATTGGCCAGAGACCGATAGCGCACAAGTAGAGTGATCGAAAGATGAAAAGCACTTTGAAAAGAGAGTTAAACAGCACGTGAAATTGTTGAAAGGGAAGCGCTTGTGACCAGACTTGCGCTGGGCTGATCATCCGGTGTTCTCACCGGTGCACTCGGCCCAGCTCAGGCCAGCATCGGTTTTGGTGGGGGGATAAAGGCGCTGGGAACGTAGCTCC--CCCGGGAGTG-TTATAGCCCAGCGTGCAATACCCCCGCTGGGACCGAGGTTCGCGCATCTGCAAGGATGCTGGCGTAATGGTCATCAGCGACCCGTCTTGAAACACGGACCAAGGAGTCAAGGTTTTGCGCGAGTGTTTGGGTGTCAAACCCGCACGCGTAATGAAAGTGAACGTAGGTGAGAG--CTTCGGCGCATCATCGACCGATCCTGATGTATTCGGATGGATTTGAGTAGGAGCGTTAAGCCTTGGACCCGAAAGATGGTGAACTATGCTTGGATAGGGTGAAGCCAGAGGAAACTCTGGTGGAGGCTCGCAGCGGTT-CTGACGTGCAAATCGATCGTCAAATCTGAGCAT-GGGGGCGAAAGACTAATCGA??????????????????????????????????????????????????????????????????????????????????????????????????????????????????????????????????????????CATTTGCGGCGAACCAACACGCCTATCGGTCGCGACGGCAAGCTTGCCAAACCCCGACAGCTGCACAATACGCACTGGGGCCTCGTGTGCCCTGCCGAGACTCCAGAAGGCCAGGCTTGCGGCCTCGTCAAGAACCTCTCCCTGATGTGCTATATTAGCGTGGGCACTAATGCTGAACCCATCGTCGACTTCATGGTTGCTAGAAACATGGAAGTCCTGGAAGAGTATGAGCCCCTCCGCTATCCCAACGCCACCAAGGTCTTCGTCAACGGAACCTGGGTCGGCGTGCACCAAGACCCGAAGCACTTGGTCACCCTAGTCCAGAATCTCAGGCGGTCCAACGTCATCTCCTTTGAAGTTTCCTTGGTGCGGGATATCCGAGACCGAGAGTTCAAGATCTTCTCTGATGCTGGCCGCGTCATGAGACCGCTTTTTGTTGTCGAGCAGGAGGACGAGAACAAG------CACACCAAGGTCCAAAAGGGCCAGCTAGTCCTAACTAGGGAACACATCAACCGGCTGGATCGGGACAAGGACCTCACGCAACTCG------------ACGAGGACTTCTTTGGCTGGAACGGCCTCCTGAGGGAAGGCTGTGTCGAGTACCTGGACGCCGAGGAAGAGGAGACGGCCATGATCTGCATGACCCCCGAAGACCTCGACCACTACCGGTCTACCAAGTTGGGC---ATTAAACCAAAAAAGAAAACAGACGAGGAGGAGGAGGGGCACAACCAGCGCATCAAAACGAAGGCGAACCCGACCACCCACATGTATACTCATTGCGAGATTCATCCTAGTATGTTGCTCGGCATCTGCGCGAGCATCATCCCCTTCCCAGATCACAACCAG??????????????????????????????????????????????????????????????????????????????????????????????????????????????????????????????????????????????????????????????????????????????????????????????????????????????????????????????????????????????????????????????????????????????????????????????????????????????????????????????????????????????????????????????????????????????????????????????????????????????????????????????????????????????????????????????????????????????????????????????????????????????????????????????????????????????????????????????????????????????????????????????????????????????????????????????????????????

>*Zygopleurage_zygospora*_SMH4219

???????????????????????????????????????????????????????????????????????????????????????????????????????????????????????????????????????????????????????????????????????????????????????????????????????????????????????????????????????????????????????????????????????????????????????????????????????????????????????????????????????????????????????????????????????????????????????????????????????????????????????????????????????????????????????????????????????????????????????????????????????????????????????AAAGAAACCAACCGGG-ATTGCCC-TAGTAACGGCG-AGTGAAGCGGCAACAGCTCAAATTTGAAATCTGGCTCC--GG-CCCGAGTTGTAATTTGCAGAGG-AAGCTTCTGGCGAAACACCTTCTAAGTCCCCTGGAACGGGGCGCCACAGTGGGTGAGAGCCCCATGTG-ATGGATGTTGA-ACCTGTGTGAAGCTCCTTCGACGAGTCGAGTAGTTTGGGAATGCTGCTCAAAATGGGAGGTAAATTCCTTCTAAAGCTAAATATTGGCCAGAGACCGATAGCGCACAAGTAGAGTGATCGAAAGATGAAAAGCACTTTGAAAAGAGGGTTAAACAGCACGTGAAATTGTTGAAAGGGAAGCGCTTGTGACCAGACTTGCGCTGGGCTGATCATCCGGTGTTCTCACCGGTGCACTCGGCCCAGCTCAGGCCAGCATCGGTTTCGGCGGGGGGATAAAGGCGCCAGGAACGTAGCTCC--CCCGGGAGTG-TTATAGCCTGGCGTGCAATGCCCCCGCTGGGACCGAGGTTCGCGCATCTGCAAGGATGCTGGCGTAATGGTCATCAGCGACCCGTCTTGAAACACGGACCAAGGAGTCAAGGTTTTGCGCGAGTGTTTGGGTGTCAAACCCGCACGCGTAATGAAAGTGAACGTAGGTGAGAG--CTTCGGCGCATCATCGACCGATCCTGATGTATTCGGATGGATTTGAGTAGGAGCGTTAAGCCTTGGACCCGAAAGATGGTGAACTATGCTTGGATAGGGTGAAGCCAGAGGAAACTCTGGTGGAGGCTCGCAGCGGTT-CTGACGTGCAAATCGATCGTCAAATCTGAGCAT-GGGGGCGAAAGACTAATCGAACCATCTAGTAGCTGGTTACCGCC??????????????????????????????????????????????????????????????????????????????????????????????????????????????????????????????????????????????????????????????????????????????????????????????????????????????????????????????????????????????????????????????????????????????????????????????????????????????????????????????????????????????????????????????????????????????????????????????????????????????????????????????????????????????????????????????????????????????????????????????????????????????????????????????????????????????????????????????????????????????????????????????????????????????????????????????????????????????????????????????????????????????????????????????????????????????????????????????????????????????????????????????????????????????????????????????????????????????????????????????????????????????????????????????????????????????????????????????????????????????????????????????????????????????????????????????????????????????????????????????????????????????????GCTTGTTGACCAAGTTCTCGATGTCGTCCGTCGTGAGGCTGAGGGCTGCGACTGCCTCCAGGGCTTCCAGATCACCCACTCCCTCGGTGGTGGTACCGGTGCCGGTATGGGTACCCTCCTTATCTCCAAGATTCGCGAGGAGTTCCCCGACCGCATGATGGCGACCTTCTCCGTCGTTCCCTCCCCTAAGGTCTCGGATACCGTCGTCGAGCCCTACAACGCCACCCTGTCGGTGCACCAGCTTGTCGAGAACTCGGATGAGACCTTCTGCATTGACAACGAGGCTCTCTACGACATCTGCATGAGGACGCTCAAGCTCTCCAACCCCTCTTATGGCGACCTTAACCACCTCGTCTCCGCTGTCATGTCGGGTGTCACCGTTTCGCTCCGTTTCCCTGGCCAGCTTAACTCCGACCTGCGCAAGCTTGCTGTCAACATGGTYCCCTTCCCTCGTCTGCACTTCTTCATGGTCGGCTTCGCGCCTCTTACTAGCCGTGGCGCGCACTCTTTCCGTGCTGTCTCGGTTCCTGAGCTCACCCAGCAGATGTTCGACCCCAAGAACATGATGGCCGCCTCCGACTTCCGCAACGGTCGCTACCTCACTTGCTCTGCCATCTT

>*Zygospermella_insignis*_E00204312

???????????????????????????????????????????????????????????????????????????????????????????????????????????????????????????????????????????????????????????????????????????????????????????????????????????????????????????????????????????????????????????????????????????????????????????????????????????????????????????????????????????????????????????????????????????????????????????????????????????????????????????????????????????????????????????????????????????????????????????????????????????????????????AAAGAAACCAACAGGG-ATTGCCC-TAGTAACGGCG-AGTGAAGCGGCAACAGCTCAAATTTGAAATCTGGCTTC--GG-CCCGAGTTGTAATTTGTAGAGG-AAGCTTCTGGTGCGGCACCGGCTGAGTCCCCTGGAACGGGGCGCCATAGAGGGTGAGAGCCCCGTATAGCCGGCTGCCTA-GCCTGTGTGAAGCTCCTTCGACGAGTCGAGTAGTTTGGGAATGCTGCTCAAAATGGGAGGTAAATTCCTTCTAAAGCTAAATATTGGCCAGAGACCGATAGCGCACAAGTAGAGTGATCGAAAGATGAAAAGCACTTTGAAAAGAGGGTTAAACAGCACGTGAAATTGTTAAAAGGGAAGCGCTTGTGACCAGACTTGCGCCGGGGCGATCATCCGGTGTTCTCACCGGTGCACTCGCCCCGGCTCAGGCCAGCATCGGTTTTCGCGGGGGGATAAAGGCACCGGGAACGTAGCTCC--TTCGGGAGTG-TTATAGCCCGGGGTGTAATGCCCCCGCGGGGACCGAGGACCGCGCATCTGCAAGGATGCTGGCGTAATGGTCACCAGCGACCCGTCTTGAAACACGGACCAAGGAGTCAAGGTTTTGCGCGAGTGTTTGGGTGTAAAACCCGCACGCGTAATGAAAGTGAACGTAGGTGAGAG--CTTCGGCGCATCATCGACCGATCCTGATGTATTCGGATGGATTTGAGTAGGAGCGTTAAGCCTTGGACCCGAAAGATGGTGAACTATGCTTGGATAGGGTGAAGCCAGAGGAAACTCTGGTGGAGGCTCGCAGCGGTT-CTGACGTGCAAATCGATCGTCAAATCTGAGCAT-GGGGGCGAAAGACTAATCGAACCATCTAGTAGCTGGTTACCGCC??????????????????????????????????????????????????????????????????????????????????????????????????????????????????????????????????????????????????????????????????????????????????????????????????????????????????????????????????????????????????????????????????????????????????????????????????????????????????????????????????????????????????????????????????????????????????????????????????????????????????????????????????????????????????????????????????????????????????????????????????????????????????????????????????????????????????????????????????????????????????????????????????????????????????????????????????????????????????????????????????????????????????????????????????????????????????????????????????????????????????????????????????????????????????????????????????????????????????????????????????????????????????????????????????????????????????????????????????????????????????????????????????????????????????????????????????????????????????????????????????????????????????GCTTGTTGACCAAGTTCTCGATGTCGTTCGTCGCGAGGCTGAGGGCTGCGACTGCCTCCAGGGCTTCCAGATCACCCACTCCCTCGGCGGTGGTACCGGTGCTGGTATGGGTACGCTCCTTATCTCCAAGATCCGCGAGGAGTTCCCCGACCGCATGATGGCTACCTTCTCGGTTGTGCCTTCCCCCAAGGTCTCGGACACCGTCGTCGAGCCCTACAACGCCACCCTCTCGGTCCATCAGCTGGTCGAGAACTCGGACGAGACCTTCTGCATTGACAACGAGGCTCTCTACGACATCTGCATGCGGACTCTCAAGCTGTCCAACCCCTCGTACGGCGACCTGAACCACCTGGTCTCGGCCGTCATGTCGGGTGTCACCGTTTCGCTGCGCTTCCCCGGCCAGCTCAACTCGGATCTCCGCAAGCTCGCCGTCAACATGGTTCCTTTCCCGCGTCTCCATTTCTTCATGGTTGGCTTCGCGCCCCTCACCAGCCGTGGAGCTTACTCTTTCCGCTCCGTCTCGGTCCCCGAGTTGACCCAGCAGATGTTCGACCCCAAGAACATGATGGCTGCTTCCGACTTCCGCAACGGTCGCTACCTGACCTGCTCCGCCATCTT

>*Zygospermella_insignis*_Lundqvist_2444-h

?????????????????????????????????????????????????????????????????????????????????????????????????????????????????????????????????????????????????????????????????????????????????????????????????????????????????????????????????????????????????????????????????????????????????????????????????????????????????????????????????????????????????????????????????????????????????????????????????????????????????????????????????????????????????????????????????????????????????????????????????????????????????????????????????????????????????????ACGGCG-AGTGAAGCGGCAACAGCTCAAATTTGAAATCTGGCTTC--GG-CCCGAGTTGTAATTTGTAGAGG-AAGCTTCTGGTGCGGCACCGGCTGAGTCCCCTGGAACGGGGCGCCATAGAGGGTGAGAGCCCCGTATAGCCGGCTGCCTA-GCCTGTGTGAAGCTCCTTCGACGAGTCGAGTAGTTTGGGAATGCTGCTCAAAATGGGAGGTAAATTCCTTCTAAAGCTAAATATTGGCCAGAGACCGATAGCGCACAAGTAGAGTGATCGAAAGATGAAAAGCACTTTGAAAAGAGGGTTAAACAGCACGTGAAATTGTTAAAAGGGAAGCGCTTGTGACCAGACTTGCGCCGGGGCGATCATCCGGTGTTCTCACCGGTGCACTCGCCCCGGCTCAGGCCAGCATCGGTTTTCGCGGGGGGATAAAGGCACCGGGAACGTAGCTCC--TTCGGGAGTG-TTATAGCCCGGGGTGTAATGCCCCCGCGGGGACCGAGGACCGCGCATCTGCAAGGATGCTGGCGTAATGGTCACCAGCGACCCGTCTTGAAACACGGACCAAGGAGTCAAGGTTTTGCGCGAGTGTTTGGGTGTAAAACCCGCACGCGTAATGAAAGTGAACGTAGGTGAGAG--CTTCGGCGCATCATCGACCGATCCTGATGTATTCGGATGGATTTGAGTAGGAGCGTTAAGCCTTGGACCCGAAAGATGGTGAACTATGCTTGGATAGGGTGAAGCCAGAGGAAACTCTGGTGGAGGCTCGCAGCGGTT-CTGACGTGCAAATCGATCGTCAAATCTGAGCAT-GGGGGCGAAAGACTAATCGAACCATCTAGTAGCTGGTTACCGCC??????????????????????????????????????????????????????????????????????????????????????????????????????????????????????????????????????????????????????????????????????????????????????????????????????????????????????????????????????????????????????????????????????????????????????????????????????????????????????????????????????????????????????????????????????????????????????????????????????????????????????????????????????????????????????????????????????????????????????????????????????????????????????????????????????????????????????????????????????????????????????????????????????????????????????????????????????????????????????????????????????????????????????????????????????????????????????????????????????????????????????????????????????????????????????????????????????????????????????????????????????????????????????????????????????????????????????????????????????????????????????????????????????????????????????????????????????????????????????????????????????????????????GCTTGTTGACCAAGTTCTCGATGTCGTTCGTCGCGAGGCTGAGGGCTGCGACTGCCTCCAGGGCTTCCAGATCACCCACTCCCTCGGCGGTGGTACCGGTGCTGGTATGGGTACGCTCCTTATCTCCAAGATCCGCGAGGAGTTCCCCGACCGCATGATGGCTACCTTCTCGGTTGTGCCTTCCCCCAAGGTCTCGGACACCGTCGTCGAGCCCTACAACGCCACCCTCTCGGTCCATCAGCTGGTCGAGAACTCGGACGAGACCTTCTGCATTGACAACGAGGCTCTCTACGACATCTGCATGCGGACTCTCAAGCTGTCCAACCCCTCGTACGGCGACCTGAACCACCTGGTCTCGGCCGTCATGTCGGGTGTCACCGTTTCGCTGCGCTTCCCCGGCCAGCTCAACTCGGATCTCCGCAAGCTCGCCGTCAACATGGTTCCTTTCCCGCGTCTCCATTTCTTCATGGTTGGCTTCGCGCCCCTCACCAGCCGTGGAGCTTACTCTTTCCGCTCCGTCTCGGTCCCCGAGTTGACCCAGCAGATGTTCGACCCCAAGAACATGATGGCTGCTTCCGACTTCCGCAACGGTCGCTACCTGACCTGCTCCGCCATCTT

>*Mammaria_echinobotryoides*_CBS_277.63

ATTACAGAGTT-------GCAAAACTCCC--AAACCA-TTGTGAACGTTACCCGTAA--CCG-TTGCTTCGGCGTGGCCTCTCTGAGTAT-----TTATACAAATAAGTTAAAACTTTCAACAACGGATCTCTTGGTTCTGGCATCGATGAAGAACGCAGCGAAATGCGATAAGTAATGTGAATTGCAGAATTCAGTGAATCATCGAATCTTTGAACGCACATTGCGCCCGCTAGTATTCTGGCGGGCATGCCTGTTCGAGCGTCATTTCAA---CCATCAAG-CCCCC---GGGCTTGCGTTGGGG-CCCTGCG-GCT-GCC--GCAGGCCCT-GAAAAACAGTGGCGGGCTCGCT-GTCAC-ACCGAGCGTAGTATTG--TATCTCGCTTTGGCCGT-GCGGCGGG-TGCCAGCCGTTAAACACCCCCC----TTCCACAAGGTTGACCTCGGATCAGGTAGGAATACCCGCTGAACTTAAGCATATCAATAAGCGGAGGAAAAGAAACCAACAGGG-ATTGCCCTTAGTAACGGCGAAGTGAAGCGGCAACAGCTCAAATTTGAAATCTGGCCTC--GG-CCCGAGTTGTAATTTGTAGAGG-AAGCTTTTGGTGCGGCACCTACTGAGTCCCCTGGAACGGGGCGCCATAGAGGGTGAGAGCCCCGTATAGTAGGACGCCTA-TCCTTTGTAAAGCTCCTTCGACGAGTCGAGTAGTTTGGGAATGCTGCTCAAAATGGGAGGTAAATTTCTTCTAAAGCTAAATATTGGCCAGAGACCGATAGCGCACAAGTAGAGTGATCGAAAGATGAAAAGCACTTTGAAAAGAGGGTTAAACAGCACGTGAAATTGTTGAAAGGGAAGCGCTTGTGACCAGACATGCGGCGGGCTGATCATCCGGTGTTCTCACCGGTGCACTCTGCCCGCCTCAGGCCAGCATCGGTTCTCGCGGGGGGATAAAGGCCTTGGGAACGTAGCTCC--TCCGGGAGTG-TTATAGCCCAGGGTGCAATGCCCTCGCGGGGACCGAGGTTCGCGC-TCTGCAAGGATGCTGGCGTAATGGTCACCAGCGACCCGTCTTGAAACACGGACCAAGGAGTCAAGGTTTTGCGCGAGTGTTTGGGTGTAAAACCCGCACGCGTAATGAAAGTGAACGTAGGTGAGAGCCCTCGGGCGCATCATCGACCGATCCTGATGTATTCGGATGGATTTGAGTAGGAGCGTTAAGCCTTGGACCCGAAAGATGGTGAACTATGCTTGGATAGGGTGAAGCCAGAGGAAACTCTGGTGGAGGCTCGCAGCGGTT-CTGACGTGCAAATCGATCGTCAAATCTGAGCATGGGGGGCGAAAGACTAATCGAACCATCTAGTAGCTGGTTACTCGC????????????????????????????????????????????????????????????????????????????????????????????????????????????????????????????????????????????????????????????????????????????????????????????????????????????????????????????????????????????????????????????????????????????????????????????????????????????????????????????????????????????????????????????????????????????????????????????????????????????????????????????????????????????????????????????????????????????????????????????????????????????????????????????????????????????????????????????????????????????????????????????????????????????????????????????????????????????????????????????????????????????????????????????????????????????????????????????????????????????????????????????????????????????????????????????????????????????????????????????????????????????????????????????????????????????????????????????????????????????????????????????????????????????????????????????????????????????????????????????????????????????????????????????????????????????????????????????????????????????????????????????????????????????????????????????????????????????????????????????????????????????????????????????????????????????????????????????????????????????????????????????????????????????????????????????????????????????????????????????????????????????????????????????????????????????????????????????????????????????????????????????????????????????????????????????????????????????????????????????????????????????????????????????????????????????????????????????????????????????????????????????????????????????????????????????????????????????????????????????

>*Mammaria_echinobotryoides*_CBS_458.65

ATTACAGAGTT-------GCAAAACTCCC--AAACCA-TTGTGAACGTTACCCGTAA--CCG-TTGCTTCGGCGTGGCCTCTCTGAGTAT-----TTATACAAATAAGTTAAAACTTTCAACAACGGATCTCTTGGTTCTGGCATCGATGAAGAACGCAGCGAAATGCGATAAGTAATGTGAATTGCAGAATTCAGTGAATCATCGAATCTTTGAACGCACATTGCGCCCGCTAGTATTCTGGCGGGCATGCCTGTTCGAGCGTCATTTCAA---CCATCAAG-CCCCC---GGGCTTGCGTTGGGG-CCCTGCG-GCT-GCC--GCAGGCCCT-GAAAAACAGTGGCGGGCTCGCT-GTCAC-ACCGAGCGTAGTATTG--TATCTCGCTTTGGCCGT-GCGGCGGG-TGCCAGCCGTTAAACACCCCCC----TTCCACAAGGTTGACCTCGGATCAGGTAGGAATACCCGCTGAACTTAAGCATATCAAAAGCGGAGGAAAAAGAAACCAACAGGG-ATTGCCC-TAGTAACGGCG-AGTGAAGCGGCAACAGCTCAAATTTGAAATCTGGCCTC--GG-CCCGAGTTGTAATTTGTAGAGG-AAGCTTTTGGTGCGGCACCTACTGAGTCCCCTGGAACGGGGCGCCATAGAGGGTGAGAGCCCCGTATAGTAGGACGCCTA-TCCTTTGTAAAGCTCCTTCGACGAGTCGAGTAGTTTGGGAATGCTGCTCAAAATGGGAGGTAAATTTCTTCTAAAGCTAAATATTGGCCAGAGACCGATAGCGCACAAGTAGAGTGATCGAAAGATGAAAAGCACTTTGAAAAGAGGGTTAAACAGCACGTGAAATTGTTGAAAGGGAAGCGCTTGTGACCAGACATGCGGCGGGCTGATCATCCGGTGTTCTCACCGGTGCACTCTGCCCGCCTCAGGCCAGCATCGGTTCTCGCGGGGGGATAAAGGCCTTGGGAACGTAGCTCC--TCCGGGAGTG-TTATAGCCCAGGGTGCAATGCCCTCGCGGGGACCGAGGTTCGCGC-TCTGCAAGGATGCTGGCGTAATGGTCACCAGCGACCCGTCTTGAAACACGGACCAAGGAGTCAAGGTTTTGCGCGAGTGTTTGGGTGTAAAACCCGCACGCGTAATGAAAGTGAACGTAGGTGAGAGCCCTCGGGCGCATCATCGACCGATCCTGATGTATTCGGATGGATTTGAGTAGGAGCGTTAAGCCTTGGACCCGAAAGATGGTGAACTATGCTTGGATAGGGTGAAGCCAGAGGAAACTCTGGTGGAGGCTCGCAGCGGTT-CTGACGTGCAAATCGATCGTCAAATCTGAGCATGGGGGGCGAAAGACTAATCGAAACCATCTAGTAGCTGGGTTACCG????????????????????????????????????????????????????????????????????????????????????????????????????????????????????????????????????????????????????????????????????????????????????????????????????????????????????????????????????????????????????????????????????????????????????????????????????????????????????????????????????????????????????????????????????????????????????????????????????????????????????????????????????????????????????????????????????????????????????????????????????????????????????????????????????????????????????????????????????????????????????????????????????????????????????????????????????????????????????????????????????????????????????????????????????????????????????????????????????????????????????????????????????????????????????????????????????????????????????????????????????????????????????????????????????????????????????????????????????????????????????????????????????????????????????????????????????????????????????????????????????????????????????????????????????????????????????????????????????????????????????????????????????????????????????????????????????????????????????????????????????????????????????????????????????????????????????????????????????????????????????????????????????????????????????????????????????????????????????????????????????????????????????????????????????????????????????????????????????????????????????????????????????????????????????????????????????????????????????????????????????????????????????????????????????????????????????????????????????????????????????????????????????????????????????????????????????????????????????????????

>*Schizochlamydosporiella_marina*_strain_FMR_20114

TTTACAGAGTT-------GCAAAACTTCC-CAACCCTTTTTGTGAACGTACT-GAA---AAG-TTGCTTCGGCGTGGCATCTCTGAGTAG-----CTTATA-AATAAGTTAAAACTTTCAACAACGGATCTCTTGGTTCTGGCATCGATGAAGAACGCAGCGAAATGCGATACGTAATGTGAATTGCAGAATTCAGTGAATCATCGAATCTTTGAACGCACATTGCGCCCGCCAGTATTCTGGCGGGCATGCCTGTTCGAGCGTCATTTCAA---CCATCAAG-CCCCA----GGCTTGTGTTGGAG-CCCTGCG-GCT-GCC--GCAGCCTCC-TAAAAGCAGTGGCGGGCTCGCT-ATCAC-ACCGAGTGCAGTAGTTTACTCTTCGCTCAGGGCGT-GTGGCGGG-TTCCAGCCGTTAAACCCCCTAC----TTTTACAAGGTTGACCTCGGATCAGGTAGGAATACCCGCTGAACTTAAGCATATCAATAAGCGGAGGAAAAGAAACCAACAGGG-ATTGCCC-TAGTAACGGCG-AGTGAAGCGGCAACAGCTCAAATTTGAAATCTGGCTCC--GG-CCCGAGTTGTAATTTGCAGAGG-AAGCTTCTGGTGATATACTGTCTAAGTCCCCTGGAACGGGGCGCCACAGTGGGTGAGAGCCCCATATG-ACGGATGTAGA-TCCTGTGTGAAGCTCCTTCGACGAGTCGAGTAGTTTGGGAATGCTGCTCAAAATGGGAGGTAAATTCCTTCTAAAGCTAAATATTGGCCAGAGACCGATAGCGCACAAGTAGAGTGATCGAAAGATGAAAAGCACTTTGAAAAGAGGGTTAAACAGCACGTGAAATTGTTGAAAGGGAAGCGCTTATGACCAGACTTGCGCTGGGCTGATCATCCGGTGTTCTCACCGGTGCACTCGGCCCAGCTCAGGCCAGCATCGGTTTTGGTGGGTGGATAAAGGCTCTGGGAACGTAGCTCC--TCCGGGAGTG-TTATAGCCCAGGGTGCAATACACCCGCTGGGACCGAGGTTCGCGCATCTGCAAGGATGCTGGCGTAATGGTCATCAGCGACCCGTCTTGAAACACGGACCAAGGAGTCAAGGTTTTGCGCGAGTGTTTGGGTGTCAAACCCGCACGCGTAATGAAAGTGAACGTAGGTGAGAG--CTTCGGCGCATCATCGACCGATCCTGATGTATTCGGATGGATTTGAGTAGGAGCGTTAAGCCTTGGACCCGAAAGATGGTGAACTATGCTTGGATAGGGTGAAGCCAGAGGAAACTCTGGTGGAGGCTCGCAGCGGTT-CTGACGTGCAAATCGATCGTCAAATCTGAGCAT???????????????????????????????????????????????????????????????????????????????????????????????????????????????????????????????????????TACACCTTTGCCTCGACGCTCTCCCATCTGCGGCGAACAAATACACCCATTGGTCGCGACGGCAAACTTGCCAAGCCTCGCCAGCTGCACAACACACATTGGGGTCTCGTCTGCCCTGCCGAAACCCCAGAAGGCCAGGCTTGTGGTCTCGTCAAGAACCTTTCCTTGATGTGCTACATCAGCGTGGGCACGGACGCCGAACCCATCGTCGACTTCATGGTTGCTAGAAACATGGAAGTCCTCGAAGAGTATGAGCCGCTCCGCTACCCCAACGCTACCAAGGTCTTTGTCAATGGAACCTGGGTTGGCGTCCATCAAGACCCGAAGCACTTGGTCACTTTGGTCCAGAATCTCAGAAGATCAAACGTCATTAGTTTTGAGGTTTCTCTTGTTCGAGATATCCGAGACCGAGAGTTCAAGATCTTCTCTGACGCTGGCCGTGTTATGAGGCCACTCTTTGTTGTCGAGCAGGAAGAAGAGAACAAG------GACACTGGGGTTCAAAAGGGCCAGCTGACCCTAACAAGAGCACACCTGAACAGGCTGGATCGGGACAAGGATATCGGGCCACTGG------------ACGACGATTTCTTTGGCTGGAACGGCCTCTTGAGAGAAGGTTGCGTGGAGTACCTCGACGCTGAGGAAGAGGAGACCGCCATGATTTGCATGACCCCCGAAGACCTGGAACACTACCGCGATGTCAAGCTGGGTATCATCAAAGAGAATGAGGAGGACCCAGAAGAACTC---GAGTCTAACAAGCGCATCAAAACCAAGGCGAACCCCACGACGCACATGTATACCCATTGCGAGATTCACCCCAGCATGCTGCTTGGCATCTGCGCGAGCATTATCCCCTTCCC???????????????????????????????????????????????????????????????????????????????????????????????????????????????????????????????????????????????????????????????????????????????????????????????????????????????????????????????????????????????????????????????????????????????????????????????????????????????????????????????????????????????????????????????????????????????????????????????????????????????????????????????????????????????????????????????????????????????????????????????????????????????????????????????????????????????????????????????????????????????????????????????????????????????????????????????????????????????????????

>*Lasiosphaeria_lanuginosa*_SMH3819

ATTACAGAGTT-------GCAAAACTCCC--AAACCA-TCGCGAACT-TACC-GTA---CCG-TTGCTTCGGCGTGGTATCTCTGAGTAT----AACATACAAATAAGTTAAAACTTTCAACAACGGATCTCTTGGTTCTGGCATCGATGAAGAACGCAGCGAAATGCGATAAGTAATGTGAATTGCAGAATTCAGTGAATCATCGAATCTTTGAACGCACATTGCGCCCGCCAGTATTCTGGCGGGCATGCCTGTTCGAGCGTCATTTCAA---CCATCAGG-CCCTC---GGGCCCGTGTTGGGG-CGCTGCGCGCT-GCCGCGCAGGCCCT-CAAAACCAGTGGCGGGCTCGCT-GTCGC-ACCGAGCGTAGTAACA--TATCTCGCTTTGGACGC-GCGGCGGG-CGCTTGCCGTAAAACACCCCCC-----TTCTCAAGGTTGACCTCGGATCAGGTAGGAATACCCGCTGAACTTAAGCATATCAATAAGCGGAGGAAAAGAAACCAACAGGG-ATTGCCC-TAGTAACGGCG-AGTGAAGCGGCAACAGCTCAAATTTGAAATCTGGCCTCCCGG-CCCGAGTTGTAATTTGTAGAGG-AAGCTTCTGGCGAGGTACCTGCTGAGTCCCCTGGAACGGGGCGCCATAGAGGGTGAGAGCCCCGTATAGCAGAGTGCCGA-CCCTGTGTGAAGCTCCTTCGACGAGTCGAGTAGTTTGGGAATGCTGCTCTAAATGGGAGGTAAATTTCTTCTAAAGCTAAATATTGGCCAGAGACCGATAGCGCACAAGTAGAGTGATCGAAAGATGAAAAGCACTTTGAAAAGAGGGTTAAATAGCACGTGAAATTGTTGAAAGGGAAGCGCTTGTGATCAGACTTGCGCCCGGCGGATCATCCGGCGTTCTCGCCGGTGCACTCCGCCGGGCTCAGGCCAGCATCGGTTCTCGCGGGGGGATAAAGGCTCGGGGAACGTGGCTCC--TCCGGGAGTG-TTATAGCCCCTTGTGCAATGCCCTCGCGGGGACCGAGGTTCGCGCATCTGCAAGGATGCTGGCGTAATGGTCATCAGCGACCCGTCTTGAAACACGGACCAAGGAGTCAAGGTTTTGCGCGAGTGTTTGGGTGTAAAACCCGCACGCGTAATGAAAGTGAACGTAGGTGAGAG--CTTCGGCGCATCATCGACCGATCCTGATGTATTCGGATGGATTTGAGTAAGAGCGTTAAGCCTTGGACCCGAAAGATGGTGAACTATGCTTGGATAGGGTGAAGCCAGAGGAAACTCTGGTGGAGGCTCGCAGCGGTT-CTGACGTGCAAATCGATCGTCAAATCTGAGCAT-GGGGGCGAAAGACTAATCGAACCATCTAGTAGCTGGTTACCGCCGCCCTGAAGTACTCTCTTGCGACTGGAAATTGGGGTGATCAGAAGAAGGCCATGAGCTCCACAGCCGGTGTCTCGCAGGTTTTGAACAGATACACCTTTGCTTCGACCTTGTCACATTTGAGGCGCACCAATACACCTATTGGCCGTGATGGCAAACTCGCCAAGCCACGGCAGCTGCACAACACACATTGGGGCCTGGTCTGTCCGGCTGAGACACCAGAAGGACAGGCTTGTGGGCTTGTCAAGAATCTGTCATTGATGTGTTATGTCAGTGTGGGCTCGGCCGCGGATCCGATCATCGAGTTTATGACCTCTCGTAACATGGAGATCCTGGAAGAGTACGAACCGCTGCGCTACCCGAACGCCACCAAGGTGTTTGTCAACGGCTCCTGGGTCGGTGTTCATCAAGATCCGAAGCACTTGGTGAACATGGTGCAGAGTCTACGGAGAAAGAACGTGATTTCCTATGAGGTATCGCTCGTCCGTGACATCCGTGACCGGGAGTTCAAAATCTTTTCCGACGCTGGTCGCGTCATGAGGCCGCTCTTTGTTGTGGAGACAGAAGAGGTTGGGGAG---------ACCGGAGTTGAGAAGGGCCAACTCATCTTGAGCAAAGAACACATTGGGAAGCTCGAGGCAGACAAGGAGTTGGGCAAGTACC------------ACCCTGACTACTGGGGCTGGAATGGTCTCCGAGCGTCAGGTGCTATTGAATATCTCGATGCTGAGGAAGAAGAATCGGCCATGATCTGCATGACACCCGAAGATCTCGAATACTTCCGCCGGACTAAGAT---------AAAGGGGAAGCCCAAGCCGAAGGAGCAGGTCCATCTAGGCAATGCGCGCATCAGGACACAGTACAATCCGACAACGCATATGTACACGCATTGCGAGATTCACCCCAGTATGCTTCTCGGCATTTGTGCAAGCATCATCCCCTTCCCCGACCACAACCAAGCTTGTTGACCAAGTTCTTGATGTTGTCCGTCGCGAGGCCGAGGGCTGCGACTGCCTCCAGGGTTTCCAGATCACCCACTCTCTTGGTGGTGGAACTGGTGCCGGTATGGGTACCCTCCTGATCTCCAAGATTCGCGAGGAGTTCCCCGACCGCATGATGGCTACTTTCTCCGTCGTGCCCTCCCCCAAGGTCTCCGACACTGTCGTCGAGCCCTACAACGCCACCCTCTCTGTCCATCAGCTGGTTGAGAACTCCGATGAGACCTTCTGCATTGACAACGAGGCTCTTTACGACATTTGCATGCGGACCCTGAAGCTGTCCAACCCTTCGTACGGCGATCTCAACTACCTTGTTTCTGCCGTCATGTCGGGTGTCACTGTTTCTCTGCGCTTCCCTGGCCAGCTTAACTCTGATCTCCGCAAGCTCGCCGTCAACATGGTTCCTTTCCCGCGTCTCCATTTCTTCATGGTCGGCTTTGCGCCTCTCACCAGCCGCGGTGCCCACTCCTTCCGCGCCGTCTCGGTTCCCGAGTTGACCCAGCAGATGTTCGACCCCAAGAACATGATGGCTGCCTCCGACTTCCGCAACGGTCGCTACCTGACCTGCTCTGCCATCTT

>*Lasiosphaeria_glabrata*_SMH4617

ATTACAGAGTT-------GCAAAACTCCC-TAAACCA-TCGCGAACG-TACCTGTA---CCG-TTGCTTCGGCGTATTATCTCTGAGTAT----AACATACAAATAAGTTAAAACTTTCAACAACGGATCTCTTGGTTCTGGCATCGATGAAGAACGCAGCGAAATGCGATAAGTAATGTGAATTGCAGAATTCAGTGAATCATCGAATCTTTGAACGCACATTGCGCCCGCTAGTATTCTGGCGGGCATGCCTGTTCGAGCGTCATTTCAA---CCATCAAG-CCCCC---GGGCCTGTGTTGGGG-CTCTGCGCGCT-GTCGCGCAGGCTCT-TAAAAATAGTGGCGGGCTCGCT-GTCGC-ACCGAGCGTAGTAATCT-TATCTCGCTTCGGACGC-GTGGCTTG-CGCTTGCCGTTAAACACCCCCT-----TTCTTAAGGTTGACCTCGGATCAGGTAGGAATACCCGCTGAACTTAAGCATATCAATAAGCGGAGGAAAAGAAACCAACAGGG-ATTGCCC-TAGTAACGGCG-AGTGAAGCGGCAACAGCTCAAATTTGAAATCTGGCCCCCCGG-CCCGAGTTGTAATTTGTAGAGG-AAGATTCTGGTGAGGTACCTGCTGAGTCCCCTGGAACGGGGCGCCATAGAGGGTGAGAGCCCCGTATAGCAGGATGCCGA-CCCTGTGTGAATCTCCTTCGACGAGTCGAGTAGTTTGGGAATGCTGCTCTAAATGGGAGGTAAATTTCTTCTAAAGCTAAATATTGGCCAGAGACCGATAGCGCACAAGTAGAGTGATCGAAAGATGAAAAGCACTTTGAAAAGAGGGTTAAATAGCACGTGAAATTGTTGAAAGGGAAGCGCTTGTGATCAGACTTGCGCCTGGCGGATCATCCGGCGTTCTCGCCGGTGCACTCCGCCTGGCACAGGCCAGCATCGGTTCTCGCGGGGAGATAAAGGCTCGGGGAACGTGGCTCCTTTCCGGGAGTGTTTATAGCCCCGGGTGCAATGCCCTCGCGGGGACCGAGGTTCGCGCATCTGCAAGGATGCTGGCGTAATGGTCATCAGCGACCCGTCTTGAAACACGGACCAAGGAGTCAAGGTTTTGCGCGAGTGTTTGGGTGTAAAACCCGCACGCGTAATGAAAGTGAACGTAGGTGAGAG--CTTCGGCGCATCATCGACCGATCCTGATGTATTCGGATGGATTTGAGTAAGAGCGTTAAGCCTTGGACCCGAAAGATGGTGAACTATGCTTGGATAGGGTGAAGCCAGAGGAAACTCTGGTGGAGGCTCGCAGCGGTT-CTGACGTGCAAATCGATCGTCAAATCTGAGCAT-GGGGGCGAAAGACTAATCGAACCATCTAGTAGCTGGTTACCGCCGCCTTGAAGTACTCGCTTGCGACTGGAAATTGGGGCGACCAGAAGAAGGCCATGAGCTCTACAGCTGGCGTCTCGCAGGTTTTGAACCGATACACATTTGCTTCGACCTTGTCGCATCTGAGGCGTACCAACACGCCCATTGGTCGCGATGGCAAACTTGCCAAGCCACGGCAGCTGCACAACACACATTGGGGTCTCGTTTGTCCGGCAGAGACACCAGAAGGACAGGCTTGTGGCCTTGTCAAGAACCTGTCTTTGATGTGTTATGTCAGTGTGGGCTCGGCTGCAGATCCCATTATTGAGTTTATGACCTCGAGAAATATGGAGATTCTGGAAGAGTACGAACCCCTGCGCTACCCGAATGCCACCAAGGTGTTTGTCAACGGCTCTTGGGTTGGTGTTCATCAAGACCCGAAGCACTTGGTGAACATGGTGCAGAGTCTACGGAGAAAAAACGTCATTTCCTATGAGGTATCGCTCGTTAGAGACATCCGTGACCGGGAGTTCAAAATATTCTCCGACGCGGGTCGCGTCATGAGGCCGCTTTTTGTTGTGGAGACGGAAGATATTGGCGAG---------ACCGGAGTCGAAAAGGGCCAACTTATCCTGAGCAAGGAACACATTGCGCGGCTGGAGAGAGACAGAGAGTTGGGTAAATACC------------ACCCCGACTACTGGGGCTGGAATGGCCTGCGAATGTCAGGTGCCATCGAATACCTCGATGCTGAGGAGGAGGAGTCTGCAATGATCTGCATGACGCCCGGAGATCTCGAATACTTCTGGCGAACCAAGAT---------AAAGGGAAAGCCCAAAAGGAAGGAGCAGGTTCATCTTGGGAATGGACGAATTAAGACGCGGTATAACCCGACCACGCACATGTACACGCATTGCGAGATTCACCCCAGTATGCTTCTTGGCATCTGTGCAAGCATCATCCCCTTCCCCGATCACAATCAGGCTGGTTGACCAAGTCCTTGACGTGGTCCGCCGCGAGGCTGAGGGCTGCGACTGCCTCCAGGGTTTCCAGATCACCCACTCTCTCGGTGGTGGAACTGGTGCCGGCATGGGTACCCTCCTTATCTCCAAGATTCGCGAAGAGTTCCCCGACCGCATGATGGCTACCTTCTCCGTCGTACCCTCCCCCAAGGTCTCGGACACTGTCGTAGAGCCGTACAATGCCACCCTCTCCGTCCACCAGCTGGTCGAGAACTCTGATGAGACTTTCTGCATTGACAACGAGGCTCTCTACGACATTTGTATGCGCACCTTGAAGCTGTCCAACCCTTCGTACGGCGACCTCAACTACCTGGTTTCCGCCGTCATGTCGGGCGTTACCGTTTCTCTGCGCTTCCCTGGACAGCTTAACTCTGATCTCCGCAAGCTCGCCGTCAACATGGTTCCTTTCCCGCGTCTCCATTTCTTCATGGTCGGCTTTGCGCCCCTTACCAGCCGCGGTGCCCACTCCTTCCGTGCCGTCTCGGTCCCCGAGTTGACCCAGCAGATGTTCGACCCCAAGAACATGATGGCTGCCTCCGACTTCCGCAATGGTCGTTACCTGACGTGCTCTGCCATCTT

>*Lasiosphaeria_ovina*_SMH1538

ATTACAGAGTT-------GCAAAACTCCC--AAACCA-TCGCGAACT-TACC-GTA---CCG-TTGCTTCGGCGTGGTATCTCTGAGTAT----AACATACAAATAAGTTAAAACTTTCAACAACGGATCTCTTGGTTCTGGCATCGATGAAGAACGCAGCGAAATGCGATAAGTAATGTGAATTGCAGAATTCAGTGAATCATCGAATCTTTGAACGCACATTGCGCCCGCTAGTATTCTGGCGGGCATGCCTGTTCGAGCGTCATTTCAA---CCATCAGG-CCCCC---GGGCCCGTGTTGGGG-CACTGCGCGCC-GCCGCGCAGGCCCT-CAAAACCAGTGGCGGGCTCGCT-GTCGC-ACCGAGCGTAGTAACA--TATCTCGCTTAGGACGC-GTAGCGGG-CGCTTGCCGTAAAACACTCCCT------TCTCAAGGTTGACCTCGGATCAGGTAGGAATACCCGCTGAACTTAAGCATATCAATAAGCGGAGGAAAAGAAACCAACAGGG-ATTGCCC-TAGTAACGGCG-AGTGAAGCGGCAACAGCTCAAATTTGAAATCTGGCCCCCAGG-CCCGAGTTGTAATTTGTAGAGG-AAGCTTCTGGTGAGGTACCTGCTGAGTCCCCTGGAACGGGGCGCCATAGAGGGTGAGAGCCCCGTATAGCAGAGTACCGA-CCCTATGTGAAGCTCCTTCGACGAGTCGAGTAGTTTGGGAATGCTGCTCTAAATGGGAGGTAAATTTCTTCTAAAGCTAAATATTGGCCAGAGACCGATAGCGCACAAGTAGAGTGATCGAAAGATGAAAAGCACTTTGAAAAGAGGGTTAAATAGCACGTGAAATTGTTGAAAGGGAAGCGCTTGTGATCAGACTTGCGCCCGGCGGATCATCCGGCGTTCTCGCCGGTGCACTCCGCCGGGCTCAGGCCAGCATCGGTTCTCGCGGGGGGATAAAGGCTCGGGGAACGTAGCTCC--TCCGGGAGTG-TTATAGCCCCTTGTGCAATGCCCTCGCGGGGACCGAGGTTCGCGCATCTGCAAGGATGCTGGCGTAATGGTCATCAGCGACCCGTCTTGAAACACGGACCAAGGAGTCAAGGTTTTGCGCGAGTGTTTGGGTGTAAAACCCGCACGCGTAATGAAAGTGAACGTAGGTGAGAG--CTTCGGCGCATCATCGACCGATCCTGATGTATTCGGATGGATTTGAGTAAGAGCGTTAAGCCTTGGACCCGAAAGATGGTGAACTATGCTTGGATAGGGTGAAGCCAGAGGAAACTCTGGTGGAGGCTCGCAGCGGTT-CTGACGTGCAAATCGATCGTCAAATCTGAGCAT-GGGGGCGAAAGACTAATCGAACCATCTAGTAGCTGGTTACCGCCGCCCTGAAGTACTCTCTTGCGACTGGAAATTGGGGCGATCAGAAGAAGGCTATGAGCTCTACAGCTGGCGTCTCGCAGGTTTTGAACAGATATACTTTTGCCTCGACCTTGTCACATTTGAGGCGCACCAATACGCCCATTGGCCGTGACGGCAAACTCGCCAAGCCACGGCAGCTGCACAACACACATTGGGGCCTGGTCTGTCCGGCAGAGACACCAGAAGGACAGGCTTGTGGGCTTGTCAAGAATCTGTCATTGATGTGTTATGTCAGTGTGGGCTCGGCTGCAGATCCGATCATCGAGTTCATGACCTCTCGCAACATGGAGATCCTGGAAGAGTACGAACCCCTGCGCTACCCGAACGCCACCAAGGTGTTCGTCAACGGCTCCTGGGTCGGTGTTCATCAAGACCCAAAGCACTTGGTGAACATGGTGCAGAGTTTACGGAGAAAGAACGTGATTTCCTATGAGGTATCGCTCGTCCGTGACATTCGTGACCGGGAGTTCAAAATCTTTTCCGACGCTGGTCGCGTCATGAGGCCGCTCTTTGTCGTGGAGACAGAAGATGTTGGGGAG---------ACTGGAGTTGAAAAGGGCCAACTCATTTTGAGCAAAGCACACATTGGGAAGCTTGAGGCAGACAAGGAGTTGGGCAAGTATC------------ACCCTGACTACTGGGGCTGGAATGGTCTTCGAGCGTCAGGTGCTATCGAGTATCTCGATGCTGAAGAGGAAGAATCGGCCATGATCTGCATGACACCCGAAGATCTCGAATACTTCCGCCGGACCAAGAT---------AAAGGGGAAGCCCAAGACGAAGGAGCAGCACCATCTCGGCAATGCGCGCATCAGGACGCAGTACAATCCGACAACTCACATGTACACGCATTGCGAGATTCACCCCAGCATGCTTCTCGGCATCTGTGCAAGCATCATCCCCTTCCCCGACCACAACCAAGCTTGTTGACCAAGTTCTTGATGTCGTCCGTCGCGAGGCCGAGGGCTGCGACTGCCTTCAGGGTTTCCAGATCACCCACTCTCTCGGTGGTGGAACTGGTGCTGGTATGGGTACCCTCCTGATCTCCAAGATTCGCGAGGAGTTCCCCGACCGCATGATGGCTACTTTCTCCGTCGTGCCCTCCCCCAAGGTCTCGGACACTGTCGTCGAGCCCTACAACGCCACCCTCTCTGTCCATCAGCTGGTTGAGAACTCCGATGAGACCTTCTGCATTGACAACGAGGCTCTCTACGACATTTGCATGCGGACCCTGAAGCTGTCCAACCCTTCGTACGGCGATCTCAACTACCTTGTTTCCGCCGTCATGTCGGGTGTCACTGTTTCTCTGCGCTTTCCTGGCCAGCTTAACTCTGATCTCCGCAAGCTCGCTGTGAACATGGTTCCTTTCCCGCGTCTCCATTTCTTCATGGTCGGCTTTGCGCCTCTCACCAGCCGTGGTGCCCACTCCTTCCGCGCCGTCTCGGTTCCCGAGTTGACCCAGCAGATGTTCGACCCCAAGAACATGATGGCTGCCTCCGACTTCCGCAACGGTCGCTACCTGACCTGCTCTGCCATCTT

>*Lasiosphaeria_rugulosa*_SMH1518

ATAACAGAGTT-------GCAAGACTCC--TAAACCA-TCGCGAACC-TACCCGTAC--CCG-TTGCTTCGGCGTGGTATCTCTGAGTAA----AAAATACAAATAAGTTAAAACTTTCAACAACGGATCTCTTGGTTCTGGCATCGATGAAGAACGCAGCGAAATGCGATAAGTAATGTGAATTGCAGAATTCAGTGAATCATCGAATCTTTGAACGCACATTGCGCCCGCTAGTATTCTGGCGGGCATGCCTGTTCGAGCGTCATTTCAA---CCATCAGG-CCTCT---GGGCCCGTGTTGGGG-CTCTGTGCGCT-GCTGTGCAGGCCCT-CAAAACTAGTGGCGGGCTCGCT-GTCGC-ACCGAGCGTAGTAATATAAATCTCGCTCAGGTCGC-GTGGCGGG-CGCTAGCCGTAAAACACCCTAC-----TCCACAAGGTTGACCTCGGATCAGGTAGGAATACCCGCTGAACTTAAGCATATCAATAAGCGGAGGAAAAGAAACCAACAGGG-ATTGCCC-TAGTAACGGCG-AGTGAAGCGGCAACAGCTCAAATTTGAAATCTGGCCCCCCGG-CCCGAGTTGTAATTTGTAGAGG-AAGCTTCAGGTGAGGTCCCTGCTGAGTCCCCTGGAACGGGGCGCCATAGAGGGTGAGAGCCCCGTATAGCAGGATGCCGA-CCCTGTGTGAAGCTCCTTCGACGAGTCGAGTAGTTTGGGAATGCTGCTCTAAATGGGAGGTAAATTTCTTCTAAAGCTAAATATTGGCCAGAGACCGATAGCGCACAAGTAGAGTGATCGAAAGATGAAAAGCACTTTGAAAAGAGGGTTAAATAGCACGTGAAATTGTTGAAAGGGAAGCGCTTGTGATCAGACTTGCGCCCGGCGGATCATCCGGCGTTCTCGCCGGTGCACTCTGCCAGGCTCAGGCCAGCATCGGTTCTCGCGGGGGGACAAAGGTTCGGGGAACGTGGCTCC--TTCGGGAGTG-TTATAGCCCCGGGCGTAATGCCCTCGCGGGGACCGAGGACCGCGCATCTGCAAGGATGCTGGCATAATGGTCATTAGCGACCCGTCTTGAAACACGGACCAAGGAGTCAAGGTTTTGCGCGAGTGTTTGGGTGTAAAACCCGCACGCGTAATGAAAGTGAACGTAGGTGAGAG--CTTCGGCGCATCATCGACCGATCCTGATGTATTCGGATGGATTTGAGTAAGAGCGTTAAGCCTTGGACCCGAAAGATGGTGAACTATGCTTGGATAGGGTGAAGCCAGAGGAAACTCTGGTGGAGGCTCGCAGCGGTT-CTGACGTGCAAATCGATCGTCAAATCTGAGCAT-GGGGGCGAAAGACTAATCGAACCATCTAGTAGCTGGTTACCGCCGCTTTGAAGTATTCGCTTGCGACCGGAAACTGGGGCGATCAGAAGAAGGCTATGAGCTCTACAGCTGGCGTCTCGCAGGTGCTAAACCGGTACACATTTGCTTCGACCCTGTCCCATTTGAGGCGAACCAACACACCTATCGGTCGTGACGGCAAACTTGCCAAGCCCAGACAGCTGCACAACACCCACTGGGGGCTAGTTTGCCCAGCAGAGACACCAGAAGGGCAGGCTTGCGGTCTTGTCAAGAATCTGTCGCTGATGTGCTATGTCAGCGTGGGCTCGGCCGCCGAACCCATTATCGAATTTATGACGTCCAGGAACATGGAGATTCTGGAGGAGTACGAGCCTCTACGCTACCCCAACGCCACCAAGGTGTTCGTCAATGGCTCTTGGGTCGGTGTCCACCAGGACCCTAAGCATCTGGTGAACATGGTTCAGAGCCTGCGCCGAAAGAACGTGATTTCTTATGAGGTATCACTGGTTAGAGACATCCGTGACCGAGAATTCAAGATATTTTCCGACGCAGGTCGCGTTATGAGGCCACTCTTTGTTGTGGAAACAGAAGATATCGGTGAA---------ACGGGTGTCGAGAGGGGCCAGCTTATTTTGAGCAAGGACCACATCGAGAGGCTTGAGAGAGACAAGGAATTGGGAAAATACC------------ATCCCGACTACTGGGGTTGGAACGGCCTCCGAGCGTCAGGTGCCATCGAATACCTCGATGCCGAGGAGGAAGAGTCGGCCATGATCTGCATGACTCCCGAGGATCTCGAATACTTCCGGCGGACTAAGAT---------CAAGGGCAAGCCTAAGTCTAAGGAGCAGGTACACCTTGGCAATGCACGAATCAAGACACAGTACAACCCGACAACGCACATGTACACGCATTGCGAGATTCACCCCAGCATGCTTCTTGGTATCTGCGCAAGCATCATCCCGTTCCCCGATCACAACCAAGCTTGTTGACCAAGTTCTTGATGTCGTGCGTCGCGAGGCCGAGGGCTGTGACTGCCTCCAGGGTTTCCAGATCACCCACTCTCTCGGTGGTGGAACCGGCGCCGGCATGGGTACCCTCCTGATCTCCAAGATTCGTGAGGAGTTCCCTGACCGTATGATGGCTACCTTCTCTGTCGTGCCCTCCCCCAAGGTCTCGGACACTGTCGTCGAGCCGTACAACGCCACTCTCTCCGTCCATCAGCTGGTCGAGAATTCCGATGAGACCTTCTGTATTGACAACGAAGCTCTCTACGACATCTGCATGCGGACTCTGAAGCTGTCCAACCCGTCGTACGGTGACCTCAACTACCTAGTCTCCGCCGTCATGTCCGGCGTCACCGTTTCGCTGCGCTTCCCCGGCCAGCTTAACTCTGATCTCCGCAAGCTCGCCGTCAACATGGTTCCTTTCCCGCGTCTTCACTTCTTCATGGTCGGCTTTGCGCCTCTTACCAGCCGCGGTGCCCACTCCTTCCGCGCCGTGTCGGTCCCTGAGTTGACGCAGCAGATGTTCGACCCCAAGAACATGATGGCCGCCTCCGACTTCCGCAATGGCCGTTACCTGACGTGCTCCGCCATCTT

>*Lasiosphaeria_miniovina*_SMH_2392

ATTACAGAGTT-------GCAAAACTCCC--AAACCA-TTGTGAACT-TACC-GTA---CCG-TTGCTTCGGCGTGGTATCTCTGAGTAT----AACATATAAATAAGTTAAAACTTTCAACAACGGATCTCTTGGTTCTGGCATCGATGAAGAACGCAGCGAAATGCGATAAGTAATGTGAATTGCAGAATTCAGTGAATCATCGAATCTTTGAACGCACATTGCGCCCGCCAGTATTCTGGCGGGCATGCCTGTTCGAGCGTCATTTCAA---CCATCAGG-CCCCC---GGGCCCGCGTTGGGG-CACTGCGCGCC-GCCGCGCAGGCCCT-CAAAACTAGTGGCGGGCTCGCT-GTCGC-ACCGAGCGTAGTAACA--TATCTCGCTTTGGACGC-GCGGCGGG-CGCTTGCCGTAAAACACCTCCC-----TTCTCAAGGTTGACCTCGGATCAGGTAGGAATACCCGCTGAACTTAAGCATATCAATAAGCGGAGGAAAAGAAACCAACAGGG-ATTGCCC-TAGTAACGGCG-AGTGAAGCGGCAACAGCTCAAATTTGAAATCTGGCCCCCAGG-CCCGAGTTGTAATTTGTAGAGG-AAGCTTCTGGCGAGGTACCTGCTGAGTCCCCTGGAACGGGGCGCCATAGAGGGTGAGAGCCCCGTATAGCAGAGTGCCGA-CCCTATGTGAAGCTCCTTCGACGAGTCGAGTAGTTTGGGAATGCTGCTCTAAATGGGAGGTAAATTTCTTCTAAAGCTAAATATTGGCCAGAGACCGATAGCGCACAAGTAGAGTGATCGAAAGATGAAAAGCACTTTGAAAAGAGGGTTAAATAGCACGTGAAATTGTTGAAAGGGAAGCGCTTGTGATCAGACTTGCGCCTGGCGGATCATCCGGCGTTCTCGCCGGTGCACTCCGCTAGGCTCAGGCCAGCATCGGTTCTCGCGGGGGGATAAAGGCTCGGGGAACGTGGCTCC--TCCAGGAGTG-TTATAGCCCCTTGTGCAATGCCCTCGCGGGGACCGAGGTTCGCGCATCTGCAAGGATGCTGGCGTAATGGTCATCAGCGACCCGTCTTGAAACACGGACCAAGGAGTCAAGGTTTTGCGCGAGTGTTTGGGTGTAAAACCCGCACGCGTAATGAAAGTGAACGTAGGTGAGAG--CTTCGGCGCATCATCGACCGATCCTGATGTATTCGGATGGATTTGAGTAAGAGCGTTAAGCCTTGGACCCGAAAGATGGTGAACTATGCTTGGATAGGGTGAAGCCAGAGGAAACTCTGGTGGAGGCTCGCAGCGGTT-CTGACGTGCAAATCGATCGTCAAATCTGAGCAT-GGGGGCGAAAGACTAATCGAACCATCTAGTAGCTGGTTACCGCC????????????????????????????????????????????????????????????????????????????????????????????????????????????????????????????????????????????????????????????????????????????????????????????????????????????????????????????????????????????????????????????????????????????????????????????????????????????????????????????????????????????????????????????????????????????????????????????????????????????????????????????????????????????????????????????????????????????????????????????????????????????????????????????????????????????????????????????????????????????????????????????????????????????????????????????????????????????????????????????????????????????????????????????????????????????????????????????????????????????????????????????????????????????????????????????????????????????????????????????????????????????????????????????????????????????????????????????????????????????????????????????????????????????????????????????????????????????????????????????????????????????????????????????????????????????????????????????????????????????????????????????????????????????????????????????????????????????????????????????????????????????????????????????????????????????????????????????????????????????????????????????????????????????????????????????????????????????????????????????????????????????????????????????????????????????????????????????????????????????????????????????????????????????????????????????????????????????????????????????????????????????????????????????????????????????????????????????????????????????????????????????????????????????????????????????????????????????????????????????

>*Lasiosphaeria_sorbina*_CBS_885.85

?????????TT-------GCAAGACTCCC--AAACCA-TCGCGAACT-TACC-GTA---CCG-TTGCTTCGGCGTGGCATCTCTGAGTGTGTGGCATGCAAAATGAAGTCAAAACTTTCAACAACGGATCTCTTGGTTCTGGCATCGATGAAGAACGCAGCGAAATGCGATAAGTAATGTGAATTGCAGAATTCAGTGAATCATCGAATCTTTGAACGCACATTGCGCCCGCCAGTATTCTGGCGGGCATGCCTGTCCGAGCGTCATTTCAA---CCAGTCGGGCCCCG---GCGCCCGTGTTGGGG-CACTGCGCGCCAGCCGCGCAGGCCCTCTGAAACCAGTGGCGGGCTCGCC-GTCGC-ACCGAGCGCAGTAACG--CATCTCGCTCAGGGCGC-GCGGCGGG-CGCTTGCCGTAAAACGACCCTC--TCCTCCTCAAGGTTGACCTCGGATCAGGTAGGAATACCCGCTGAACTTAAGCATATCAAT??????????AAAGAAACCAACAGGG-ATTGCCC-TAGTAACGGCG-AGTGAAGCGGCAACAGCTCAAATTTGAAATCCGGCCCCCCGGGCCCGAGTTGTAATTTGCAGAGG-AAGCTTCCGGCGAGGTGCCTGCTGAGTCCCCTGGAACGGGGCGCCATAGAGGGTGAGAGCCCCGTATAGCAGAGCGCCGA-CCCTGTGTGAAGCTCCTTCGACGAGTCGAGTAGTTTGGGAATGCTGCTCTAAATGGGAGGTAAATTTCTTCTAAAGCTAAATACTGGCCAGAGACCGATAGCGCACAAGTAGAGTGATCGAAAGATGAAAAGCACTTTGAAAAGAGGGTTAAATAGCACGTGAAATTGTTGAAAGGGAAGCGCTTGTGATCAGACTTGCGCCCGGCGGATCATCCGGCGTTCTCGCCGGTGCACTCCGCCGGGCTCAGGCCAGCATCGGTTCTCGTGGGGGGACAAAGGTCCGGGGAACGTGGCTCC--TCCGGGAGTG-TTATAGCCCCGGGCGCAATGCCCTCGTGGGGACCGAGGTTCGCGCGTCCGCAAGGATGCTGGCGTAATGGTCATCAGCGACCCGTCTTGAAACACGGACCAAGGAGTCAAGGTTTTGCGCGAGTGTTTGGGTGTCAAACCCGCACGCGTAATGAAAGTGAACGTAGGTGAGAG--CTTCGGCGCATCATCGACCGATCCTGATGTTCTCGGATGGATTTGAGTAAGAGCGTCAAGCCTTGGACCCGAAAGATGGTGAACTATGCTTGGATAGGGTGAAGCCAGAGGAAACTCTGGTGGAGGCTCGCAGCGGTT-CTGACGTGCAAATCGATCGTCAAATCTGAGCAT-GGGGGCGAAAGACTAATCGAACCATCTAGTAGCTGGTTACCGCCGCCCTGAAGTACTCTCTTGCGACTGGAAATTGGGGCGATCAGAAGAAGGCCATGAGCTCCACAGCCGGCGTCTCGCAGGTTCTGAATCGATATACTTTTGCTTCGACCTTGTCACATTTGAGGCGCACCAACACACCAATTGGCCGTGACGGCAAACTCGCCAAGCCCCGACAGCTGCACAACACACATTGGGGCCTGGTCTGTCCGGCAGAGACACCAGAAGGGCAGGCTTGTGGCCTTGTCAAGAATCTGTCACTAATGTGTTATGTCAGTGTGGGTTCTGCTGCAGATCCGATCATCGAGTTTATGACCTCTCGTAACATGGAGATCCTGGAAGAGTACGAACCCCTGCGCTACCCGAACGCCACCAAAGTGTTCGTGAACGGCTCCTGGGTCGGTGTTCATCAAGACCCGAAGCACTTGGTGAACATGGTGCAGAGTTTACGGAGAAAGAACGTGATTTCCTATGAGGTATCGCTCGTTCGAGACATTCGTGACCGGGAGTTCAAGATCTTTTCCGATGCTGGTCGCGTCATGAGGCCGCTCTTCGTTGTGGAAACAGAAGATGTTGGGGAG---------ACCGGAGTTGAGAAGGGCCAACTCATCTTGAGCAAGGGACACATTGAGAAGCTTGAGGGAGACAAGGAGTTGGGCAAGTACC------------ATCCCGACTACTGGGGCTGGAATGGTCTTCGAGCGTCAGGTGCTATTGAATATCTCGACGCCGAAGAAGAAGAATCGGCCATGATTTGCATGACACCCGAAGATCTCGAATACTTCCGCCGGACTAAGAT---------AAAGGGGAAGCCCAAGTCGAAGGAGCAGGTCCATCTCGGCAACGCGCGAATCCGGACGCAGTACAATCCGACAACGCACATGTACACGCATTGCGAGATTCACCCCAGCATGCTTCTCGGCATCTGCGCAAGCATCATCCCCTTCCCCGACCACAACCAAGCTCGTTGATCAAGTTCTTGATGTCGTCCGTCGCGAGGCCGAAGGCTGCGACTGCCTCCAGGGTTTCCAGATCACCCACTCTCTCGGTGGTGGAACTGGTGCCGGTATGGGCACCCTCCTGATCTCCAAGATTCGCGAGGAGTTCCCCGACCGCATGATGGCTACTTTCTCCGTCGTGCCCTCCCCCAAGGTCTCGGACACTGTCGTCGAGCCGTACAACGCCACCCTCTCTATCCATCAGCTGGTTGAGAACTCCGATGAGACCTTCTGCATTGACAACGAAGCTCTCTACGACATTTGCATGCGGACTCTGAAGCTGTCCAACCCTTCGTACGGCGACCTCAACTACCTTGTTTCCACCGTCATGTCGGGCGTCACTGTTTCTCTGCGCTTCCCTGGCCAGCTTAACTCTGATCTCCGCAAGCTCGCCGTCAACATGGTTCCTTTCCCGCGTCTCCATTTCTTCATGGTCGGCTTTGCGCCCCTTACTAGCCGCGGCGCCCACTCCTTCCGTGCTGTCTCGGTTCCCGAGTTGACGCAGCAGATGTTCGACCCCAAGAACATGATGGCTGCCTCCGATTTTCGCAACGGTCGCTACCTGACCTGCTCTGCCATCTT

>*Lasiosphaeria_similisorbina*_AR_1884

ATTACAGAGTT-------GCAAAACTCCC--AAACCA-TCGCGAACT-TACC-GTA---CCG-TTGCTTCGGCGTGGTATCTCTGAGTAT----AACATACAAATAAGTTAAAACTTTCAACAACGGATCTCTTGGTTCTGGCATCGATGAAGAACGCAGCGAAATGCGATAAGTAATGTGAATTGCAGAATTCAGTGAATCATCGAATCTTTGAACGCACATTGCGCCCGCCAGTATTCTGGCGGGCATGCCTGTTCGAGCGTCATTTCAA---CCATCAGG-CCCCC--TGGGCCCGTGTTGGGG-CACTGCGCGCC-GCCGCGCAGGCCCT-CAAAACCAGTGGCGGGCTCGCT-GTCGC-ACCGAGCGTAGTAACA--TATCTCGCTTTGGACGC-GCGGCGGG-CGCCTGCCGTGAAACACCCCCC-----TTCTCAAGGTTGACCTCGGATCAGGTAGGAATACCCGCTGAACTTAAGCATATCAATAAGCGGAGGAAAAGAAAC-AACAGGG-ATTGCCC-TAGTAACGGCG-AGTGAAGCGGCAACAGCTCAAATTTGAAATCTGGCCCCCAGG-CCCGAGTTGTAATTTGTAGAGG-AAGCTTCTGGCGAGGTACCTGCTGAGTCCCCTGGAACGGGGCGCCATAGAGGGTGAGAGCCCCGTATAGCAGAGTGCCGA-CCCTGTGTGAAGCTCCTTCGACGAGTCGAGTAGTTTGGGAATGCTGCTCTAAATGGGAGGTAAATTTCTTCTAAAGCTAAATATTGGCCAGAGACCGATAGCGCACAAGTAGAGTGATCGAAAGATGAAAAGCACTTTGAAAAGAGGGTTAAATAGCACGTGAAATTGTTGAAAGGGAAGCGCTTGTGATCAGACTTGCGCCCGGCGGATCATCCGGCGTTCTCGCCGGTGCACTCCGCCGGGCTCAGGCCAGCATCGGTTCTCGCGGGGGGATAAAGGCTCGGGGAACGTGGCTCCTCTCGGGGAGTG-TTATAGCCCCTTGTGCAATGCCCTCGCGGGGACCGAGGTTCGCGCATCTGCAAGGATGCTGGCGTAATGGTCATCAGCGACCCGTCTTGAAACACGGACCAAGGAGTCAAGGTTTTGCGCGAGTGTTTGGGTGTAAAACCCGCACGCGTAATGAAAGTGAACGTAGGTGAGAG--CTTCGGCGCATCATCGACCGATCCTGATGTATTCGGATGGATTTGAGTAAGAGCGTTAAGCCTTGGACCCGAAAGATGGTGAACTATGCTTGGATAGGGTGAAGCCAGAGGAAACTCTGGTGGAGGCTCGCAGCGGTT-CTGACGTGCAAATCGATCGTCAAATCTGAGCAT-GGGGGCGAAAGACTAATCGAACCATCTAGTAGCTGGTTACCGCC????????????????????????????????????????????????????????????????????????????????????????????????????????????????????????????????????????????????????????????????????????????????????????????????????????????????????????????????????????????????????????????????????????????????????????????????????????????????????????????????????????????????????????????????????????????????????????????????????????????????????????????????????????????????????????????????????????????????????????????????????????????????????????????????????????????????????????????????????????????????????????????????????????????????????????????????????????????????????????????????????????????????????????????????????????????????????????????????????????????????????????????????????????????????????????????????????????????????????????????????????????????????????????????????????????????????????????????????????????????????????????????????????????????????????????????????????????????????????????????????????????????????????????????????????????????????????????????????????????????????????????????????????????????????????????????????????????????????????????????????????????????????????????????????????????????????????????????????????????????????????????????????????????????????????????????????????????????????????????????????????????????????????????????????????????????????????????????????????????????????????????????????????????????????????????????????????????????????????????????????????????????????????????????????????????????????????????????????????????????????????????????????????????????????????????????????????????????????????????????

>*Zopfiella_tabulata*_CBS_230.78

ATTACAGAGTT-------GCAAAACTCCC-TAAACCA-TCGCGAACG-TTACCCCTA--CCG-TTGCTTCGGCGAGGCCTCTCTGAGTAA----TTTATACAAATAAGTTAAAACTTTCAACAACGGATCTCTTGGTTCTGGCATCGATGAAGAACGCAGCGAAATGCGATAAGTAATGTGAATTGCAGAATTCAGTGAATCATCGAATCTTTGAACGCACATTGCGCCCGCCAGTATTCTGGCGGGCATGCCTGTTCGAGCGTCATTTCAA---CCATCAAG-CCCCC----GGCTTGTGTTGGGG-CCCTGCG-GCT-GCC--GCAGGCCCT-TAAAAACAGTGGCGGGCTCGCT-GTCAC-ACCGAGCGTAGTAATA--CATCTCGCTTTGGACGT-GCAGCGGG-TTCTTGCCGTGAAACACCCCCC-----TTCTCAAGGTTGACCTCGGATCAGGTAGGAATACCCGCTGAACTTAAGCATATCAATAAGCGGAGGA?????????AACAGGG-ATTGCCC-CAGTAACGGCG-AGTGAAGCGGCAACAGCTCAAATTTGAAATCTGGCTTC--GG-CCCGAGTTGTAATTTGTAGAGG-AAGCTTTTGGTGCGGCACCTACTGAGTCCCCTGGAACGGGGCGCCATAGAGGGTGAGAGCCCCGTATAGTAGGACGCCTA-GCCTGTGTAAAGCTCCTTCGACGAGTCGAGTAGTTTGGGAATGCTGCTCTAAATGGGAGGTAAATTTCTTCTAAAGCTAAATATTGGCCAGAGACCGATAGCGCACAAGTAGAGTGATCGAAAGATGAAAAGCACTTTGAAAAGAGGGTTAAACAGCACGTGAAATTGTTGAAAGGGAAGCGCTTGTGACCAGACTTGCGCCGGGCGGATCATCCGGTGTTCTCACCGGTGCACTCCGCCCGGCTCAGGCCAGCATCGGTTCTCGTGGGGGGATAAAGGCTCTGGGAACGTGGCTCC--TCCGGGAGTG-TTATAGCCCAGGGTGCAATGCCCTCGTGGGGACCGAGGTTCGCGCATCTGCAAGGATGCTGGCGTAATGGTCATCAGCGACCCGTCTTGAAACACGGACCAAGGAGTCAAGGTTTTGCGCGAGTGTTTGGGTGTAAAACCCGCACGCGTAATGAAAGTGAACGTAGGTGAGAG--CTTCGGCGCATCATCGACCGATCCTGATGTATTCGGATGGATTTGAGTAGGAGCGTTAAGCCTTGGACCCGAAAGATGGTGAACTATGCTTGGATAGGGTGAAGCCAGAGGAAACTCTGGTGGAGGCTCGCAGCGGTT-CTGACGTGCAAATCGATCGTCAAATCTGAGCAT-GGGGGCGAAAGACTAATCGAACCATC?????????????????????????????????????????????????????????????????????????????????????????????????????????????????????????????????????????????CGTACAAGCACGCCTGTTGGACGTGACGGCAAGCTTGCCAAGCCCCGCCAGCTACACAATACACATTGGGGTCTCGTCTGCCCGGCAGAGACACCTGAAGGACAGGCCTGTGGCCTCGTGAAGAACCTGTCGCTCATGTGCTTCGTCAGCGTGGGCACCGCCGCTGAGCCCATTATAGAGTTCATGATTGCTCGAAATATGGAGGTGCTTGAAGAGTACGAACCCCTGCGGTATCCCAACGCCACCAAGGTCTTCGTCAACGGCACGTGGGTTGGCGTCCACCAAGACCCCAAGACTCTGGTCGGCTTGGTGCAGCGACTCCGGCGGAAGAATATCATCTCCTATGAGGTGTCCCTGGTTAGAGATATCCGCGATAGAGAGTTCAAGATCTTTTCCGACGCGGGCCGCGTTATGAGGCCACTTTTCGTGGTCGAGACGGATGACAACAGCGAC---------AGCGGTGCCGAGAAGGGCCAGCTGATATTGAAGAAGGACCACATTCGCCGGTTGGAAAACGACAAGATGCTGAACAAATGGG------------ATCCCGACTACTGGGGTTGGCAAGGATTGCGGTCCTCGGGCGCTATCGAGTATCTCGATGCCGAGGAAGAGGAGTCGGCCATGATCTGCATGACGCCCGAAGATCTCGACACGTACCGTCTTAGCAGGAT------------GGGCTACGAGATGGACAGCGGTGGCGGCAACCTCACCAATGCGCGCATCAAGACCAAGCTGAATCCAACTACGCACATGTACACGCATTGCGAGATTCACCCGAGCATGCTGCTCGGTATCTGCGCCAGCATCATCCCCTTCCCCGACCACAACCAG??????????????????????????????????????????????????????????????????????????????????????????????????????????????????????????????????????????????????????????????????????????????????????????????????????????????????????????????????????????????????????????????????????????????????????????????????????????????????????????????????????????????????????????????????????????????????????????????????????????????????????????????????????????????????????????????????????????????????????????????????????????????????????????????????????????????????????????????????????????????????????????????????????????????????????????????????????????

>*Cercophora_sulphurella*_SMH2531

ATTACAGAGTT-------GCAAAACTCCC-TAAACCA-TCGCGAACG-TACCTTTA---CCG-TTGCTTCGGCGAGGCCTCTCTGAGTAT-----TTATACAAATAAGTTAAAACTTTCAACAACGGATCTCTTGGTTCTGGCATCGATGAAGAACGCAGCGAAATGCGATAAGTAATGTGAATTGCAGAATTCAGTGAATCATCGAATCTTTGAACGCACATTGCGCCCGCTAGTATTCTGGCGGGCATGCCTGTTCGAGCGTCATTTCAA---CCATCAAG-CCCCG----GGCTTGTGTTGGGG-CCCTGCG-GTT-GCC--GCAGGCCCT-TAAAAACAGTGGCGGGCTCGCT-GTCAC-ACCGAGCGTAGTAATA--CATCTCGCTTTGGACGT-GCGGCGTGTTTCTTGCCGTTAAACACCCCCC----CTTCTCAAGGTTGACCTCGAATCAGGTAGGAATACCCGCTGAACTTAAGCATATCAATAAGCGGAGGAAAAGAAACCAACAGGG-ATTGCCC-TAGTAACGGCG-AGTGAAGCGGCAACAGCTCAAATTTGAAATCTGGCTTC--GG-CCCGAGTTGTAATTTGTAGAGG-AAGCTTTTGGTGCGGCATCTACTGAGTCCCCTGGAACGGGGCGCCATAGAGGGTGAGAGCCCCGTATAGTAGGACGCCTA-GCCTCTGTAAAGCTCCTTCGACGAGTCGAGTAGTTTGGGAATGCTGCTCTAAATGGGAGGTAAATTTCTTCTAAAGCTAAATATTGGCCAGAGACCGATAGCGCACAAGTAGAGTGATCGAAAGATGAAAAGCACTTTGAAAAGAGGGTTAAACAGCACGTGAAATTGTTGAAAGGGAAGCGCTTGTGACCAGACTTGCGCCGGGCGGATCATCCGGTGTTCTCACCGGTGCACTCCGCCCGGCTCAGGCCAGCATCGGTTCTCGTGGGGGGATAAAGGCTCTGGGAACGTGGCTCC--TCCGGGAGTG-TTATAGCCCAGTGTGCAATGCCCTCGTGGGGACCGAGGTTCGCGCATCTGCAAGGATGCTGGCGTAATGGTCATCAGCGACCCGTCTTGAAACACGGACCAAGGAGTCAAGGTTTTGCGCGAGTGTTTGGGTGTAAAACCCGCACGCGTAATGAAAGTGAACGTAGGTGAGAG--CTTCGGCGCATCATCGACCGATCCTGATGTATTCGGATGGATTTGAGTAGGAGCGTTAAGCCTTGGACCCGAAAGATGGTGAACTATGCTTGGATAGGGTGAAGCCAGAGGAAACTCTGGTGGAGGCTCGCAGCGGTT-CTGACGTGCAAATCGATCGTCAAATCTGAGCAT-GGGGGCGAAAGACTAATCGAACCATCTAGTAGCTGGTTACCGCCGCACTGAAGTACTCGTTAGCGACTGGCAATTGGGGCGACCAGAAGAAGGCAATGAGTTCTACCGCCGGTGTCTCGCAGGTCTTGAACAGATATACCTTTGCCTCGACCCTGTCTCATTTAAGAAGAACGAATACCCCTGTTGGCCGCGACGGCAAGCTTGCCAAACCCCGCCAGCTGCACAACACCCATTGGGGCCTCGTCTGCCCGGCAGAGACGCCAGAGGGACAGGCTTGCGGTCTGGTGAAGAATTTGTCGCTGATGTGCTTCGTGAGCGTGGGCACTGCCGCCGAGCCCATCATAGAGTTTATGGTTGCTCGAAATATGGAGGTCCTCGAAGAATACGAGCCCCTGCGATATCCCAACGCCACCAAGGTGTTCGTCAATGGCACCTGGGTTGGGGTCCACCAGGACCCAAAGCACTTAGTGAGCTTGGTGCAGCGTCTCCGGCGGAAGAACATCATTTCGTACGAGGTTTCTCTGGTGAGAGATATCCGCGACCGAGAGTTCAAGATCTTCTCCGATGCTGGTCGTGTCATGAGGCCGCTTTTCGTAGTCGAGACCGAAGACAACAGCGAT---------AGCGGAGCTGAGAAGGGCCAGTTGATATTGAAGAAGGAGCACATCCACAAGTTGGAGAACGATAAAATGCTGAACAAATGGG------------ATCCCGACTACTGGGGCTGGCAGGGGTTGCGATCGTCGGGCGCTATCGAGTACCTTGATGCCGAGGAAGAAGAGTCGGCCATGATATGCATGACACCAGAAGACCTCGACACCTACCGCTTGAGTAGAAT------------GGGCTTTGACGTGGACGGCGATGGTGGTAACCAGAACAACGCACGCATCAAGACCAAACTGAACCCAACAACGCACATGTACACGCATTGCGAGATTCATCCAAGCATGCTGCTTGGTATCTGCGCAAGCATTATCCCTTTCCCCGATCACAACCAAGCTTGTTGACCAAGTACTTGATGTCGTCCGTCGCGAGGCTGAGGGCTGCGACTGCCTCCAGGGCTTTCAGATCACCCACTCCCTTGGTGGTGGCACTGGTGCCGGTATGGGTACTCTTCTGATCTCCAAGATCCGTGAAGAGTTCCCCGATCGCATGATGGCCACCTTCTCGGTTGTGCCCTCTCCCAAGGTCTCGGATACCGTCGTGGAGCCTTACAACGCCACCCTCTCCGTCCACCAGCTGGTCGAGAACTCTGACGAGACTTTCTGCATTGACAACGAGGCTCTCTACGATATTTGTATCCGTACCCTGAAGCTCTCCAACCCTTCGTACGGTGATCTTAACCACCTGGTTTCGGCCGTCATGTCGGGTGTTACCGTTTCTCTGCGCTTCCCCGGCCAGCTGAACTCGGATCTCCGCAAGCTCGCCGTCAACATGGTTCCTTTCCCCCGTCTCCATTTCTTCATGGTCGGCTTTGCGCCCCTTACCAGCCGTGGCGCATACTCTTTCCGTGCTGTTTCCGTTCCCGAGTTGACCCAGCAGATGTTCGACCCCAAGAACATGATGGCTGCTTCCGACTTCCGCAACGGTCGCTACCTGACCTGCTCCGCCATCTT

>*Cercophora_sparsa*_JF00229

ATTACAGAGTT-------GCAAAACTCCC-TAAACCA-TCGCGAACG-TACCTTTA---CCG-TTGCTTCGGCGAGGCCTCTCTGAGTAT-----TTATACAAATAAGTTAAAACTTTCAACAACGGATCTCTTGGTTCTGGCATCGATGAAGAACGCAGCGAAATGCGATAAGTAATGTGAATTGCAGAATTCAGTGAATCATCGAATCTTTGAACGCACATTGCGCCCGCCAGTATTCTGGCGGGCATGCCTGTTCGAGCGTCATTTCAA---CCATCAAG-CCCCA----GGCTTGCGTTGGGG-TCCTGCG-GCT-GCC--GCAGGCCCT-GAAAAACAGTGGCGGGCTCGCT-GTCAC-ACCGAGCGTAGTAATA--CATCTCGCTTTGGACGT-GCGGCGGG-TTCTTGCCGTTAAACACCCCCC-----TTCTCAAGGTTGACCTCGGATCAGGTAGGAATACCCGCTGAACTTAAGCATATCAATAAGCGGAGGAAAAGAAACCAACAGGG-ATTGCCC-TAGTAACGGCG-AGTGAAGCGGCAACAGCTCAAATTTGAAATCTGGCCTC--GG-CCCGAGTTGTAATTTGTAGAGG-CGGCTTTTGGTGCGGCACCTACTGAGTCCCCTGGAACGGGGCGCCATAGAGGGTGAGAGCCCCGTATAGTAGGATGCCTA-GCCTGTGTAAAGCCCCTTCGACGAGTCGAGTAGTTTGGGAATGCTGCTCTAAATGGGAGGTAAATTTCTTCTAAAGCTAAATATTGGCCAGAGACCGATAGCGCACAAGTAGAGTGATCGAAAGATGAAAAGCACTTTGAAAAGAGGGTTAAACAGCACGTGAAATTGTTGAAAGGGAAGCGCTTGTGACCAGACTTGCGCCGGGCGGATCATCCGGTGTTCTCACCGGTGCACTCCGCCCGGCTCAGGCCAGCATCGGTTCTCGTGGGGGGATAAAGGCTCTGGGAACGTGGCTCC--TTCGGGAGTG-TTATAGCCCAGGGTGCAATGCCCTCGTGGGGACCGAGGTTCGCGCATCTGCAAGGATGCTGGCGTAATGGTCATCAGCGACCCGTCTTGAAACACGGACCAAGGAGTCAAGGTTTTGCGCGAGTGTTTGGGTGTCAAACCCGCACGCGTAATGAAAGTGAACGTAGGTGAGAG--CTTCGGCGCATCATCGACCGATCCTGATGTATTCGGATGGATTTGAGTAGGAGCGTTAAGCCTTGGACCCGAAAGATGGTGAACTATGCTTGGATAGGGTGAAGCCAGAGGAAACTCTGGTGGAGGCTCGCAGCGGTT-CTGACGTGCAAATCGATCGTCAAATCTGAGCAT-GGGGGCGAAAGACTAATCGAACCATCTAGTAGCTGGTTACCGCC??????????????????????????????????????????????????????????????????????????????????????????????????????????????????????????????????????????????????????????????????????????????????????????????????????????????????????????????????????????????????????????????????????????????????????????????????????????????????????????????????????????????????????????????????????????????????????????????????????????????????????????????????????????????????????????????????????????????????????????????????????????????????????????????????????????????????????????????????????????????????????????????????????????????????????????????????????????????????????????????????????????????????????????????????????????????????????????????????????????????????????????????????????????????????????????????????????????????????????????????????????????????????????????????????????????????????????????????????????????????????????????????????????????????????????????????????????????????????????????????????????????????????GCTTGTTGATCAAGTTCTTGACGTCGTCCGTCGCGAGGCTGAGGGCTGTGACTGCCTCCAGGGTTTCCAGATCACCCACTCCCTTGGTGGTGGTACAGGTGCCGGTATGGGTACTCTCCTGATCTCCAAGATCCGCGAGGAGTTCCCCGACCGCATGATGGCCACCTTCTCCGTTGTGCCCTCCCCCAAGGTCTCGGATACCGTTGTCGAACCTTACAACGCCACCCTCTCGGTCCACCAGCTGGTCGAGAACTCTGACGAGACCTTCTGCATTGATAACGAGGCTCTCTACGATATTTGCATGCGCACCCTGAAGCTGTCCAACCCTTCGTACGGTGATCTGAACCACCTGGTTTCGGCCGTCATGTCGGGCGTCACTGTTTCGCTGCGCTTCCCCGGTCAGCTCAACTCGGATCTCCGCAAGCTGGCTGTCAACATGGTCCCCTTCCCCCGTCTCCATTTCTTCATGGTCGGCTTTGCGCCCCTTACCAGCCGTGGCGCTCACTCTTTCCGTGCTGTTTCCGTTCCCGAGTTGACCCAGCAGATGTTCGACCCCAAGAACATGATGGCTGCTTCTGACTTCCGCAATGGTCGCTACCTGACCTGCTCTGCCATCTT

>*Anopodium_ampullaceum*_E00218015

???????????????????????????????????????????????????????????????????????????????????????????????????????????????????????????????????????????????????????????????????????????????????????????????????????????????????????????????????????????????????????????????????????????????????????????????????????????????????????????????????????????????????????????????????????????????????????????????????????????????????????????????????????????????????????????????????????????????????????????????????????????????????????AAAGAAACCAACAGGG-ATTGCCC-CAGTAACGGCG-AGTGAAGCGGCAACAGCTCAAATTTGAAATCTGGCCTC--GG-CCCGAGTTGTAATTTGTAGAGG-AAGCTTTTGGTGCGGCACCTACTGAGTCCCCTGGAACGGGGCGCCATAGAGGGTGAGAGCCCCGTATAGTAGGACGCCTA-GCCTCTGTAAAGCTCCTTCGACGAGTCGAGTAGTTTGGGAATGCTGCTCTAAATGGGAGGTAAATTTCTTCTAAAGCTAAATATTGGCCAGAGACCGATAGCGCACAAGTAGAGTGATCGAAAGATGAAAAGCACTTTGAAAAGAGGGTTAAACAGCACGTGAAATTGTTGAAAGGGAAGCGCTTGTGACCAGACTTGCGCCGGGCGGATCATCCGGTGTTCTCACCGGTGCACTCCGCCCGGCTCAGGCCAGCATCGGTTCTCGTGGGGGGATAAAGGCTCTGGGAACGTGGCTCC--TCCGGGAGTG-TTATAGCCCAGGGTGCAATGCCCTCGTGGGGACCGAGGTTCGCGCATCTGCAAGGATGCTGGCGTAATGGTCATCAGCGACCCGTCTTGAAACACGGACCAAGGAGTCAAGGTTTTGCGCGAGTGTTTGGGTGTAAAACCCGCACGCGTAATGAAAGTGAACGTAGGTGAGAG--CTTCGGCGCATCATCGACCGATCCTGATGTATTCGGATGGATTTGAGTAGGAGCGTTAAGCCTTGGACCCGAAAGATGGTGAACTATGCTTGGATAGGGTGAAGCCAGAGGAAACTCTGGTGGAGGCTCGCAGCGGTT-CTGACGTGCAAATCGATCGTCAAATCTGAGCAT-GGGGGCGAAAGACTAATCGAACCATCTAGTAGCTGGTTACCGCC??????????????????????????????????????????????????????????????????????????????????????????????????????????????????????????????????????????????????????????????????????????????????????????????????????????????????????????????????????????????????????????????????????????????????????????????????????????????????????????????????????????????????????????????????????????????????????????????????????????????????????????????????????????????????????????????????????????????????????????????????????????????????????????????????????????????????????????????????????????????????????????????????????????????????????????????????????????????????????????????????????????????????????????????????????????????????????????????????????????????????????????????????????????????????????????????????????????????????????????????????????????????????????????????????????????????????????????????????????????????????????????????????????????????????????????????????????????????????????????????????????????????????GCTTGTTGACCAAGTTCTTGATGTCGTCCGTCGCGAGGCCGAGGGCTGCGACTGCCTGCAGGGTTTCCAGATCACCCACTCCCTTGGTGGTGGTACTGGTGCCGGTATGGGTACTCTCCTGATCTCCAAGATCCGTGAGGAGTTCCCCGACCGTATGATGGCCACCTTCTCGGTTGTGCCCTCTCCCAAGGTCTCCGATACCGTTGTCGAGCCCTACAACGCCACCCTCTCCGTCCACCAGCTGGTCGAGAACTCGGACGAGACCTTCTGCATTGACAACGAGGCTCTCTACGATATTTGCATCCGTACCCTGAAGCTCTCCAACCCTTCGTACGGTGATCTTAACCACCTGGTCTCGGCCGTCATGTCGGGTGTCACCGTTTCTCTGCGCTTCCCCGGCCAGCTCAACTCGGATCTCCGCAAGCTCGCCGTCAACATGGTTCCCTTCCCCCGTCTCCATTTCTTCATGGTCGGCTTTGCGCCCCTTACCAGCCGCGGCGCATACTCTTTCCGTGCTGTTTCTGTTCCCGAGTTGACCCAGCAGATGTTCGACCCCAAGAACATGATGGCTGCTTCCGACTTCCGCAACGGTCGCTACCTGACCTGCTCTGCCATCTT

>*Anopodium_ampullaceum*_MJR_40/07

???????????????????????????????????????????????????????????????????????????????????????????????????????????????????????????????????????????????????????????????????????????????????????????????????????????????????????????????????????????????????????????????????????????????????????????????????????????????????????????????????????????????????????????????????????????????????????????????????????????????????????????????????????????????????????????????????????????????????????????????????????????????????????????????????????????????????TAACGGCG-AGTGAAGCGGCAACAGCTCAAATTTGAAATCTGGCCTC--GG-CCCGAGTTGTAATTTGTAGAGG-AAGCTTTTGGTGCGGCACCTACTGAGTCCCCTGGAACGGGGCGCCATAGAGGGTGAGAGCCCCGTATAGTAGGACGCCTA-GCCTCTGTAAAGCTCCTTCGACGAGTCGAGTAGTTTGGGAATGCTGCTCTAAATGGGAGGTAAATTTCTTCTAAAGCTAAATATTGGCCAGAGACCGATAGCGCACAAGTAGAGTGATCGAAAGATGAAAAGCACTTTGAAAAGAGGGTTAAACAGCACGTGAAATTGTTGAAAGGGAAGCGCTTGTGACCAGACTTGCGCCGGGCGGATCATCCGGTGTTCTCACCGGTGCACTCCGCCCGGCTCAGGCCAGCATCGGTTCTCGTGGGGGGATAAAGGCTCTGGGAACGTGGCTCC--TCCGGGAGTG-TTATAGCCCAGGGTGCAATGCCCTCGTGGGGACCGAGGTTCGCGCATCTGCAAGGATGCTGGCGTAATGGTCATCAGCGACCCGTCTTGAAACACGGACCAAGGAGTCAAGGTTTTGCGCGAGTGTTTGGGTGTAAAACCCGCACGCGTAATGAAAGTGAACGTAGGTGAGAG--CTTCGGCGCATCATCGACCGATCCTGATGTATTCGGATGGATTTGAGTAGGAGCGTTAAGCCTTGGACCCGAAAGATGGTGAACTATGCTTGGATAGGGTGAAGCCAGAGGAAACTCTGGTGGAGGCTCGCAGCGGTT-CTGACGTGCAAATCGATCGTCAAATCTGAGCAT-GGGGGCGAAAGACTAATCGAACCATCTAGTAGCTGGTTACCGCC??????????????????????????????????????????????????????????????????????????????????????????????????????????????????????????????????????????????????????????????????????????????????????????????????????????????????????????????????????????????????????????????????????????????????????????????????????????????????????????????????????????????????????????????????????????????????????????????????????????????????????????????????????????????????????????????????????????????????????????????????????????????????????????????????????????????????????????????????????????????????????????????????????????????????????????????????????????????????????????????????????????????????????????????????????????????????????????????????????????????????????????????????????????????????????????????????????????????????????????????????????????????????????????????????????????????????????????????????????????????????????????????????????????????????????????????????????????????????????????????????????????????????GCTTGTTGACCAAGTTCTTGATGTCGTCCGTCGCGAGGCCGAGGGCTGCGACTGCCTGCAGGGTTTCCAGATCACCCACTCCCTTGGTGGTGGTACTGGTGCCGGTATGGGTACTCTCCTGATCTCCAAGATCCGTGAGGAGTTCCCCGACCGTATGATGGCCACCTTCTCGGTTGTGCCCTCTCCCAAGGTCTCCGATACCGTTGTCGAGCCCTACAACGCCACCCTCTCCGTCCACCAGCTGGTCGAGAACTCGGACGAGACCTTCTGCATTGACAACGAGGCTCTCTACGATATTTGCATCCGTACCCTGAAGCTCTCCAACCCTTCGTACGGTGATCTTAACCACCTGGTCTCGGCCGTCATGTCGGGTGTCACCGTTTCTCTGCGCTTCCCCGGCCAGCTCAACTCGGATCTCCGCAAGCTCGCCGTCAACATGGTTCCCTTCCCCCGTCTCCATTTCTTCATGGTCGGCTTTGCGCCCCTTACCAGCCGCGGCGCATACTCTTTCCGTGCTGTTTCTGTTCCCGAGTTGACCCAGCAGATGTTCGACCCCAAGAACATGATGGCTGCTTCCGACTTCCGCAACGGTCGCTACCTGACCTGCTCTGCCATCTT

>*Bellojisia_rhynchostoma*

???????????????????????????????????????????????????????????????????????????????????????????????????????????????????????????????????????????????????????????????????????????????????????????????????????????????????????????????????????????????????????????????????????????????????????????????????????????????????????????????????????????????????????????????????????????????????????????????????????????????????????????????????????????????????????????????????????????????????????????????????????????????????????AAAGAAACCAACAGGG-ATTGCCC-TAGTAACGGCG-AGTGAAGCGGCAACAGCTCAAATTTGAAATCTGGCTTC--GG-CCCGAGTTGTAATTTGTAGAGG-AAGCTTTTGGTGCGGCACCTACTGAGTCCCCTGGAACGGGGCGCCATAGAGGGTGAGAGCCCCGTATAGTAGGACGCCTA-GCCTGTGTAAAGCTCCTTCGACGAGTCGAGTAGTTTGGGAATGCTGCTCTAAATGGGAGGTAAATTTCTTCTAAAGCTAAATATTGGCCAGAGACCGATAGCGCACAAGTAGAGTGATCGAAAGATGAAAAGCACTTTGAAAAGAGGGTTAAACAGCACGTGAAATTGTTGAAAGGGAAGCGCTTGTGACCAGACTTGCGCCGGGCGGATCATCCGGTGTTCTCACCGGTGCACTCCGCCCGGCTCAGGCCAGCATCGGTTCTCGTGGGGGGATAAAGGCTCTGGGAACGTGGCTCC--TCCGGGAGTG-TTATAGCCCAGGGTGCAATGCCCTCGTGGGGACCGAGGTTCGCGCATCTGCAAGGATGCTGGCGTAATGGTCATCAGCGACCCGTCTTGAAACACGGACCAAGGAGTCAAGGTTTTGCGCGAGTGTTTGGGTGTAAAACCCGCACGCGTAATGAAAGTGAACGTAGGTGAGAG--CTTCGGCGCATCATCGACCGATCCTGATGTATTCGGATGGATTTGAGTAGGAGCGTTAAGCCTTGGACCCGAAAGATGGTGAACTATGCTTGGATAGGGTGAAGCCAGAGGAAACTCTGGTGGAGGCTCGCAGCGGTT-CTGACGTGCAAATCGATCGTCAAATCTGAGCAT-GGGGGCGAAAGACTAATCGAACCATCTAGTAGCTGGTTACCGCC????????????????????????????????????????????????????????????????????????????????????????????????????????????????????????????????????????????????????????????????????????????????????????????????????????????????????????????????????????????????????????????????????????????????????????????????????????????????????????????????????????????????????????????????????????????????????????????????????????????????????????????????????????????????????????????????????????????????????????????????????????????????????????????????????????????????????????????????????????????????????????????????????????????????????????????????????????????????????????????????????????????????????????????????????????????????????????????????????????????????????????????????????????????????????????????????????????????????????????????????????????????????????????????????????????????????????????????????????????????????????????????????????????????????????????????????????????????????????????????????????????????????????????????????????????????????????????????????????????????????????????????????????????????????????????????????????????????????????????????????????????????????????????????????????????????????????????????????????????????????????????????????????????????????????????????????????????????????????????????????????????????????????????????????????????????????????????????????????????????????????????????????????????????????????????????????????????????????????????????????????????????????????????????????????????????????????????????????????????????????????????????????????????????????????????????????????????????????????????????

>*Ramophialophora_humicola*_FMR_9523

ATTACCGA-TT-------GCAAAA-TCTC-TAAACCA-TCGCGAACGTTACCCCACG--CCG-TTGCCTCGGCGAGGCCTCTCTGAGTAT-----TTATAAAAATAAGTTAAAACTTTCAACAACGGATCTCTTGGTTCTGGCATCGATGAAGAACGCAGCGAAATGCGATAAGTAATGTGAATTGCAGAATTCAGTGAATCATCGAATCTTTGAACGCACATTGCGCCCGCCAGTATTCTGGCGGGCATGCCTGTTCGAGCGTCATTTCAA---CCATCAAG-CCCTA----GGCTTGTGTTGGGG-CCCTGCG-GCT-GCC--GCAGGCCCT-GAAAAACAGTGGCGGGCTCGCT-GTCAC-ACCGAGCGTAGTAAAA--CATCTCGCTTTGGACGT-GCGGCGGG-TTCTTGCCGTTAAACACCCCCC-----TTCTCAAGGTTGACCTCGGATCAGGTAGGAATACCCGCTGAACTTAAGCATATCAATAGCGGGAG????????????????????????????????????????????????????????????ATTTGAAATCTGGCCTC--GG-CCCGAGTTGTAATTTGTAGAGG-AAGCTTTTGGCGCGGCACCTACTGAGTCCCCTGGAACGGGGCGCCATAGAGGGTGAGAGCCCCGTATAGTAGGACGCCTA-GCCTGTGTAAAGCTCCTTCGACGAGTCGAGTAGTTTGGGAATGCTGCTCTAAATGGGAGGTAAATTTCTTCTAAAGCTAAATATTGGCCAGAGACCGATAGCGCACAAGTAGAGTGATCGAAAGATGAAAAGCACTTTGAAAAGAGGGTTAAACAGCACGTGAAATTGTTGAAAGGGAAGCGCTTGTGACCAGACTTGCGCCGGGCGGATCATCCGGTGTTCTCACCGGTGTACTCCGCCCGGCTCAGGCCAGCATCGGTTCTCGTGGGGGGATAAAGGCTCTGGGAACGTGGCTCC--TCCGGGAGTG-TTATAGCCCAGGGTGCAATGCCCTCGTGGGGACCGAGGTTCGCGCATCTGCAAGGATGCTGGCG????????????????????????????????????????????????????????????????????????????????????????????????????????????????????????????????????????????????????????????????????????????????????????????????????????????????????????????????????????????????????????????????????????????????????????????????????????????????????????????????????????????????????????????????????????????????????????????????????????????????????????????????????????????????????????????????????????????????????????????????????????????????????????????????????????????????????????????????????????????????????????????????????????????????????????????????????????????????????????????????????????????????????????????????????????????????????????????????????????????????????????????????????????????????????????????????????????????????????????????????????????????????????????????????????????????????????????????????????????????????????????????????????????????????????????????????????????????????????????????????????????????????????????????????????????????????????????????????????????????????????????????????????????????????????????????????????????????????????????????????????????????????????????????????????????????????????????????????????????????????????????????????????????????????????????????????????????????????????????????????????????????????????????????????????????????????????????????????????????????????????????????????????????????????????????????????????????????????????????????????????????????????????????????????????????????????????????????????????????????????????????????????????????????????????????????????????????????????????????????????????????????????????????????????????????????????????????????????????????????????????????????????????????????????????????????????????????????????????????????????????????????????????????????????????????????????????????????????????????????????????????????????????????????????????????????????????????????????????????????????????

>*Podospora_didyma*_CBS_232.78

ATTACAGAGTT-------GCAAAACTCCC-TAAACCA-TCGCGAACGTTACCCCTA---CCG-TTGCTTCGGCGAGGCCTCTCTGAGTAA----TTTATACAAATAAGTTAAAACTTTCAACAACGGATCTCTTGGTTCTGGCATCGATGAAGAACGCAGCGAAATGCGATAAGTAATGTGAATTGCAGAATTCAGTGAATCATCGAATCTTTGAACGCACATTGCGCCCGCCAGTATTCTGGCGGGCATGCCTGTTCGAGCGTCATTTCAA---CCATCAAG-CCCCC----GGCTTGTGTTGGGG-CCCTGCG-GCT-GCC--GCAGGCCCT-TAAAAACAGTGGCGGGCTCGCT-GTCAC-ACCGAGCGTAGTAATA--CATCTCGCTTTGGACGT-GCAGCGGG-TTCTTGCCGTGAAACACCCCCC-----TTCTCAAGGTTGACCTCGGATCAGGTAGGAATACCCGCTGAACTTAAGCATATCAATAAGCGGAGGA??????????ACAGGG-ATTGCCC-CAGTAACGGCG-AGTGAAGCGGCAACAGCTCAAATTTGAAATCTGGCTTC--GG-CCCGAGTTGTAATTTGTAGAGG-AAGCTTTTGGTGCGGCACCTACTGAGTCCCCTGGAACGGGGCGCCATAGAGGGTGAGAGCCCCGTATAGTAGGACGCCTA-GCCTGTGTAAAGCTCCTTCGACGAGTCGAGTAGTTTGGGAATGCTGCTCTAAATGGGAGGTAAATTTCTTCTAAAGCTAAATATTGGCCAGAGACCGATAGCGCACAAGTAGAGTGATCGAAAGATGAAAAGCACTTTGAAAAGAGGGTTAAACAGCACGTGAAATTGTTGAAAGGGAAGCGCTTGTGACCAGACTTGCGCCGGGCGGATCATCCGGTGTTCTCACCGGTGCACTCCGCCCGGCTCAGGCCAGCATCGGTTCTCGCGGGGGGATAAAGGCTCTGGGAACGTGGCTCC--TCCGGGAGTG-TTATAGCCCAGGGTGCAATGCCCTCGTGGGGACCGAGGTTCGCGCATCTGCAAGGATGCTGGCGTAATGGTCATCAGCGACCCGTCTTGAAACACGGACCAAGGAGTCAAGGTTTTGCGCGAGTGTTTGGGTGTAAAACCCGCACGCGTAATGAAAGTGAACGTAGGTGAGAG--CTTCGGCGCATCATCGACCGATCCTGATGTATTCGGATGGATTTGAGTAGGAGCGTTAAGCCTTGGACCCGAAAGATGGTGAACTATGCTTGGATAGGGTGAAGCCAGAGGAAACTCTGGTGGAGGCTCGCAGCGGTT-CTGACGTGCAAATCGATCGTCAAATCTGAGCAT-GGGGGCGAAAGACTAATCGAACCATC??????????????????????????????????????????????????????????????????????????????????????????????????????????????????????????????????????????????????????????????????????????????????????????????????????????????????????????????????????????????????????????????????????????????????????????????????????????????????????????????????????????????????????????????????????????????????????????????????????????????????????????????????????????????????????????????????????????????????????????????????????????????????????????????????????????????????????????????????????????????????????????????????????????????????????????????????????????????????????????????????????????????????????????????????????????????????????????????????????????????????????????????????????????????????????????????????????????????????????????????????????????????????????????????????????????????????????????????????????????????????????????????????????????????????????????????????????????????????????????????????????????????????????????????????????????????????????????????????????????????????????????????????????????????????????????????????????????????????????????????????????????????????????????????????????????????????????????????????????????????????????????????????????????????????????????????????????????????????????????????????????????????????????????????????????????????????????????????????????????????????????????????????????????????????????????????????????????????????????????????????????????????????????????????????????????????????????????????????????????????????????????????????????????????????????????????????????????????????????????????????????????????

>*Corylomyces_selenosporus*_FMR_8279

ATTACTGAGTT--------TTCAACTCCC-TAAACCA-TCGCGAACG-TACCTTTA---CCG-TTGCTTCGGCGAGGCCTCTCTGAGTAA-----TCATACAAATAAGTTAAAACTTTCAACAACGGATCTCTTGGTTCTGGCATCGATGAAGAACGCAGCGAAATGCGATAAGTAATGTGAATTGCAGAATTCAGTGAATCATCGAATCTTTGAACGCACATTGCGCCCGCCAGTATTCTGGCGGGCATGCCTGTTCGAGCGTCATTTCAA---CCATCAAG-CCCCA----GGCTTGTGTTGGGG-TCCTGCG-GCT-GCC--GCAGGCCCT-GAAAAACAGTGGCGGGCTCGCT-GTCAC-ACCGAGCGTAGTAATA--CATCTCGCTTTGGACGT-GCGGCGGG-TTCTTGCCGTTAAACACCCCCC-----TTCTCAAGGTTGACCTCGGATCAGGTAGGAATACCCGCTGAACTTAAGCATATCAATAAGCGGAGG?AAAGAAACCAACAGGG-ATTGCCC-TAGTAACGGCG-AGTGAAGCGGCAACAGCTCAAATTTGAAATCTGGCTTC--GG-CCCGAGTTGTAATTTGTAGAGG-AAGCTTTTGGTGCGGCACCTACTGAGTCCCCTGGAACGGGGCGCCATAGAGGGTGAGAGCCCCGTATAGTAGGACGCCTA-GCCTGTGTAAAGCTCCTTCGACGAGTCGAGTAGTTTGGGAATGCTGCTCTAAATGGGAGGTAAATTTCTTCTAAAGCTAAATATTGGCCAGAGACCGATAGCGCACAAGTAGAGTGATCGAAAGATGAAAAGCACTTTGAAAAGAGGGTTAAACAGCACGTGAAATTGTTGAAAGGGAAGCGCTTGTGACCAGACTTGCGCCGGGCGGATCATCCGGTGTTCTCACCGGTGCACTCCGCCCGGCTCAGGCCAGCATCGGTTCTCGTGGGGGGATAAAGGCCCTGGGAACGTGGCTCC--TTCGGGAGTG-TTATAGCCCAGGGTGCAATGCCCTCGTGGGGACCGAGGTTCGCGCATCTGCAAGGATGCTGGCGTAATGGTCATCAGCGACCCGTCTTGAAACACGGACCAAGGAGTCAAGGTTTTGCGCGAGTGTTTGGGTGTAAAACCCGCACGCGTAATGAAAGTGAACGTAGGTGAGAG--CTTCGGCGCATCATCGACCGATCCTGATGTATTCGGATGGATTTGAGTAGGAGCGTTAAGCCTTGGACCCGAAAGATGGTGAACTATGCTTGGATAGGGTGAAGCCAGAGGAAACTCTGGTGGAGGCTCGCAGCGGTT-CTGACGTGCAAATCGATCGTCAAATCTGAGCAT-GGGGGCGAAAGACTAATCGAACCATCTAGTAGCTGGTTACCGCCGCGTTGAAGTACTCGCTCGCAACTGGCAATTGGGGCGACCAGAAGAAGGCAATGAGCTCCACCGCTGGTGTGTCGCAGGTGCTGAACAGATATACATTCGCCTCGACCCTCTCTCATTTGAGACGAACCAACACGCCTATTGGCCGTGATGGCAAGCTCGCCAAACCCCGCCAGCTGCACAACACCCATTGGGGCTTGGTTTGCCCAGCAGAGACCCCCGAGGGACAGGCTTGTGGTCTCGTGAAGAACCTGTCGCTCATGTGTTTCGTGAGCGTAGGCACGGCCGCTGAGCCTATAATAGAGTTCATGATTGCTCGAAACATGGAAGTACTGGAAGAGTACGAACCCCTGCGGTATCCTAACGCGACCAAAGTGTTCGTCAACGGCACTTGGGTTGGCGTCCACCAAGACCCGAAGCATTTGGTCAGGCTGGTGCAGAGTCTCCGGCGCAAGAACGTCATTTCGTACGAGGTGTCGCTGGTGAGGGATATCCGCGACCGAGAGTTCAAGATCTTTTCCGACGCCGGCCGCGTTATGAGGCCACTTTTCGTGGTTGAGACCGAAGACAACAGCGAC---------AGTGGGGCCGAGAAGGGCCAGTTGATATTGAAGAAAGAGCACATTCGCAGGTTGGAGAATGACAAACAACTTCAAAAGTGGG------------ACCCGGATTACTGGGGCTGGAAAGGATTGCGGTCGTCCGGCGCCATCGAGTACCTCGATGCCGAGGAGGAAGAGTCGGCCATGATCTGCATGACGCCAGAAGACCTCGACACATACCGATTGAGCAGAAT------------GGGTTTCGACGTGGACGGCGATGGCAGTGGCCAAGGTAACGCACGTATCAAGACCAAGTTGAACCCTACCACCCACATGTACACACATTGCGAGATCCATCCAAGCATGCTGCTCGGTATCTGCGCTAGCATTATCC????????????????????GCTTGTTGACCAAGTTCTTGATGTGGTCCGCCGCGAGGCTGAGGGCTGCGACTGCCTCCAGGGTTTCCAGATCACCCACTCCCTTGGTGGTGGTACCGGTGCTGGTATGGGTACTCTCCTGATCTCCAAGATCCGTGAGGAGTTCCCCGACCGCATGATGGCCACCTTCTCGGTCGTGCCCTCCCCCAAGGTCTCGGATACTGTTGTCGAGCCCTACAACGCCACCCTCTCTGTCCACCAGCTGGTCGAGAACTCTGACGAGACCTTCTGCATTGACAATGAGGCCCTCTACGACATTTGCATGCGCACCCTGAAGCTGTCCAACCCTTCGTACGGTGATCTTAACCACCTGGTTTCCGCCGTCATGTCGGGCGTCACCGTTTCGCTGCGCTTCCCCGGTCAGCTCAACTCGGATCTCCGCAAGCTGGCTGTCAACATGGTTCCCTTCCCCCGTCTCCATTTCTTCATGGTCGGCTTTGCGCCCCTTACCAGCCGTGGCGCACACTCTTTCCGTGCTGTTTCCGTTCCCGAGTTGACCCAGCAGATGTTCGACCCCAAGAACATGATGGCTGCTTCTGACTTCCGCAATGGTCGCTATCTGACCTGCTCTGCCATCTT

>*Camarops_amorpha*_SMH1450

??????????????????????????????????????????????????????????????????????????????????????????????????????????????????????????????????????????????????????????????????????????????????????????????????????????????????????????????????????????????????????????????????????????????????????????????????????????????????????????????????????????????????????????????????????????????????????????????????????????????????????????????????????????????????????????????????????????????????????????????????????????????????????????????????CAGGG-ATTGCCC-CAGTAACGGCG-AGTGAAGCGGCAACAGCTCAAATTTGAAATCTGGCCTC--GG-CCCGAGTTGTAATTTGCAGAGG-ATGCTTTCGGCGCGGCGCCTCCCGAGTCCCCTGGAACGGGGCGCCACAGAGGGTGAGAGCCCCGTATGGCAGGACGCCCC-GCCCGTGTAAAGCTCCTTCGACGAGTCGAGTAGTTTGGGAATGCTGCTCAAAATGGGAGGTAAATTCCTTCTAAAGCTAAATACCGGCCAGAGACCGATAGCGCACAAGTAGAGTGATCGAAAGATGAAAAGCACTTTGAAAAGAGGGTCAAACAGCACGTGAAATTGTTGAAAGGGAAGCGCTCGTGACCAGACTTGCGCCGGGCCGATCATCCGGCGTTCTCGCCGGTGCACTCGGCCCGGCTCAGGCCAGCATCGGTTCTCGCGGGGGGACAAAAGCGTCGGGAACGTGGCTCC--TCCGGGAGTG-TTATAGCCCGGCGCACAATGCCCCCGCGGGGACCGAGGCCCGCGC-TCCGCAAGGATGCTGGCGTAATGGTCACCGGCGACCCGTCTTGAAACACGGACCAAGGAGTCAAGGTTTTGCGCGAGTGTTCGGGTGTCAAACCCGCACGCGTAATGAAAGTGAACGTAGGTGAGAG--CTTCGGCGCATCATCGACCGATCCTGATGTTCTCGGATGGATTTGAGTAAGAGCGTGATGCCTTGGACCCGAAAGATGGTGAACTATGCCTGGATAGGGTGAAGCCAGAGGAAACTCTGGTGGAGGCTCGCAGCGGTT-CTGACGTGCAAATCGATCGTCAAATCAGGGCAT-GGGGGCGAAAGACTAATCGAACCATCTAGTAGCTGGTTACCGCCGCGCTAAAGTACTCTCTTGCCACGGGAAACTGGGGTGAACAGAAGAAAGCCATGAGTTCAACGGCTGGCGTGTCCCAGGTCCTGAACAGATACACCTTCGCGTCCACTCTATCTCACCTGCGACGCACGAACACGCCCATTGGACGCGACGGCAAGATTGCCAAGCCGCGGCAGCTGCATAACACCCATTGGGGCCTGGTTTGTCCGGCGGAGACCCCTGAGGGTCAGGCCTGTGGTCTCGTCAAGAATTTGTCTTTGATGTGCTATGTGAGCGTGGGTACACCTGCCGAGCCCATCATCGATTTCATGATCGCGAGAAACATGGAAGTCTTGGAGGAGTATGAGCCGCTGCGGTATCCCAACGCGACCAAGATTTTCGTCAACGGAACTTGGGTCGGAGTCCATCAGGACCCGAAGCATTTGGTCAGCCTCGTGCAGGGTCTTCGTCGCAAAAACGTCATTTCATTCGAGGTGTCTCTTGTCCGCGACATACGCGACCGCGAGTTCAAGATATTCTCCGATGCTGGCCGTGTCATGAGGCCCCTTTTCGTGGTGGAGCAGGATGATAATGCGGAG---------AGCGGCATCGAGAAGGGCAGGCTCATCCTGAACAAGGATCACATTCGCCGGCTGGAAGACGACAAAGAGCTCGGCAAATACC------------ACCCTGACTACTGGGGCTGGAAGGGCCTACAGGAATCCGGCGTTATCGAGTACCTCGATGCCGAAGAGGAGGAAACAGCTATGATCTGTATGACTCCCGAGGATCTGGACAATTACCGCCTCAGGAAGTT------------GGGCGTCGACATTGCGGAGGACGACAATAACAATCCGAATGCGCGTATTAAGACGAAGATCAACCCTACCACCCACATGTATACCCACTGCGAGATCCATCCGGCCATGCTTCTGGGCATCTGTGCCAGTATCATTCCCTTCCCCGACCACAACCAGGCTCGTCGACCAGGTCCTGGACGTCGTCCGCCGCGAGGCCGAGGGCTGCGACTGCTTGCAGGGCTTCCAGATCACCCACTCTCTCGGCGGTGGTACCGGCGCCGGCATGGGCACCCTGCTGATCTCCAAGATCCGCGAGGAGTTCCCCGACCGCATGATGGCCACCTTCTCGGTCGTGCCGTCCCCCAAGGTCTCCGACACCGTCGTCGAGCCCTACAACGCGACCCTGTCGGTGCACCAGCTGGTCGAGAACTCGGACGAGACCTTTTGCATCGACAACGAGGCCCTGTACGACATCTGCATGCGCACCCTGAAGCTATCGAACCCATCCTACGGCGACCTCAACCACCTCGTCTCCGCCGTCATGTCCGGCGTCACCACCTGTCTGCGCTTCCCCGGCCAGCTGAACTCGGACCTTCGCAAGCTCGCTGTCAACATGGTCCCCTTCCCACGCCTGCACTTCTTCATGGTCGGCTTCTCGCCGCTCACGAGCCGCGGCGCCTACTCATTCCGTGCCGTCACCGTGCCGGAGTTGACGCAGCAGATGTTCGACCCCAAGAACATGATGGCCGCCTCCGACTTCCGCAACGGCCGCTACCTAACCTGCTCCGCCATCTT
